# The critical role of pore size on depth-dependent microbial cell counts in sediments

**DOI:** 10.1038/s41598-020-78714-3

**Published:** 2020-12-10

**Authors:** Junghee Park, J. Carlos Santamarina

**Affiliations:** Earth Science and Engineering, King Abdullah University of Science and Technology (KAUST), Building 5, Thuwal, 23955-6900 Saudi Arabia

**Keywords:** Marine biology, Physical oceanography

## Abstract

Cell counts decrease with sediment depth. Typical explanations consider limiting factors such as water availability and chemistry, carbon source, nutrients, energy and temperature, and overlook the role of pore size. Our analyses consider sediment self-compaction, the evolution of pore size with depth, and the probability of pores larger than the microbial size to compute the volume fraction of life-compatible pores. We evaluate cell counts vs. depth profiles gathered at 116 sites worldwide. Results confirm the critical role of pore size on cell counts in the subsurface and explain much of the data spread (from ~ 9 orders of magnitude range in cell counts to ~ 2 orders). Cells colonize pores often forming dense biofilms, thus, cell counts in pores are orders of magnitude higher than in the water column. Similar arguments apply to rocks.

## Introduction

Bioactivity has been found at sediment depths in excess of 1000 m^1–3^. Overall, cell counts decrease with depth^2,4–7^; typical explanations include limiting factors such as water availability and chemistry, carbon source, nutrients, energy and temperature^8–15^. Some studies have also recognized the critical role of pore size on microbial life and the need for interconnected and traversable habitable spaces^16–18^. A previous experimental study and related analyses identified three distinct regions defined by particle size and sediment depth: “active and motile” for coarse silts and sands at all depths, and either “trapped” or “membrane punctured” for clay-controlled sediments^19^. Still, the role of pore size as a limiting factor to microbial activity in sediments remains unnoticed or overlooked.

Here, we analyze cell counts versus depth data gathered at 116 sites as part of the global Ocean Drilling Program ODP, Integrated Ocean Drilling Program IODP, and other expeditions. Our goal is to assess the role of pore size on microbial cell counts in marine sediments.

## Analyses

A detailed description of the analytical approach follows. Additional details, the complete dataset and references can be found in the Supplementary Information associated to this manuscript.

### From sediment self-compaction to cell counts

The equilibrium analysis of a sediment slice of thickness *dz* at depth *z* predicts that the effective stress gradient *dσ'*_*z*_/*dz* is a function of the local void ratio *e*_*z*_ (Note: the void ratio *e*_z_ is the volume of voids *V*_*v*_ normalized by the volume of the solid mineral phase *V*_*m*_):

1 $$ \frac{{d\sigma_{z}^{^{\prime}} }}{dz} = \rho_{w} \left[ {\frac{{G_{s} - 1}}{{1 + e_{z} }}} \right]g $$

where gravitational acceleration is *g* = 9.81 m/s^2^ and the specific gravity *G*_*s*_ = *ρ*_*m*_/*ρ*_*w*_ is the ratio between the mineral density *ρ*_*m*_ and water density *ρ*_*w*_. The sediment void ratio *e*_*z*_ decreases with depth *z* as the vertical effective stress *σ'*_*z*_ increases. We adopt an asymptotically-correct exponential compaction model, where the void ratio decreases from the asymptotic maximum value *e*_*L*_ at the water-seabed interface where *σ'*_*z*_ = 0, to the limiting void ratio *e*_*H*_ at very high effective stress^20^:

2 $$ e_{z} = e_{H} + \left( {e_{L} - e_{H} } \right)\exp \left[ { - \left( {\frac{{\sigma_{z}^{^{\prime}} }}{{\sigma_{c}^{^{\prime}} }}} \right)^{\eta } } \right] $$

The characteristic effective stress is typically between *σ'*_*c*_ = 500 and 3000 kPa, and the model parameter *η* reflects the sensitivity of the void ratio to effective stress (Note: most sediments exhibit *η* = 1/3—See related analysis^21^). The solution of the differential Eq. (1) with the constitutive model in Eq. (2) results in the following implicit equation that relates the void ratio *e*_*z*_ and the effective stress *σ'*_*z*_ at depth *z* (for *η* = 1/3—See related example^21,22^):

3 $$ z = \frac{{\left( {1 + e_{H} } \right)}}{{\left( {G_{s} - 1} \right)\rho_{w} g}}\sigma_{z}^{^{\prime}} + 3\frac{{\left( {e_{L} - e_{H} } \right)}}{{\left( {G_{s} - 1} \right)\rho_{w} g}}\sigma_{c}^{^{\prime}} \left\{ {\left[ {\left( {\frac{{\sigma_{z}^{^{\prime}} }}{{\sigma_{c}^{^{\prime}} }}} \right)^{\frac{2}{3}} + 2\left( {\frac{{\sigma_{z}^{^{\prime}} }}{{\sigma_{c}^{^{\prime}} }}} \right)^{\frac{1}{3}} + 2} \right] \cdot \exp \left[ { - \left( {\frac{{\sigma_{z}^{^{\prime}} }}{{\sigma_{c}^{^{\prime}} }}} \right)^{\frac{1}{3}} } \right] - 2} \right\} $$

Finally, we obtain the void ratio profile *e*_*z*_ = *f* (*z*) with depth *z* by replacing a selected effective stress *σ'*_*z*_ in both Eqs. (3) and (2).

### Mean pore size

The geometrical analysis of various sediment fabrics shows that the mean pore size *μ*_*d*_ [m] is a function of the void ratio *e*_*z*_ and the specific surface *S*_*s*_ defined as the ratio between the surface area of particles *A*_*s*_ and their mass, *S*_*s*_ = *A*_*s*_/(*ρ*_*m*_*V*_*m*_) [m^2^/g]^18,23^,

4 $$ \mu_{d} = k\frac{{e_{z} }}{{S_{s} \rho_{m} }} $$

where the *k-*factor reflects the soil fabric (see geometric analyses and values for various fabrics in the Supplementary Table S1). Furthermore, our database shows a strong correlation between the specific surface *S*_*s*_ and the asymptotic void ratio *e*_*L*_ (see Supplementary Fig. S1):

5 $$ e_{L} = 0.9 + 0.03\frac{{S_{s} }}{{[{\text{m}}^{2} /{\text{g}}]}} $$

For large rotund particles, the specific surface *S*_*s*_ → 0 while the asymptotic void ratio tends to *e*_*L*_ → 0.9 which corresponds to the loose, simple cubic packing of monosize spherical particles.

### Pore size distribution (Soils and rocks)

**Figure 1:**
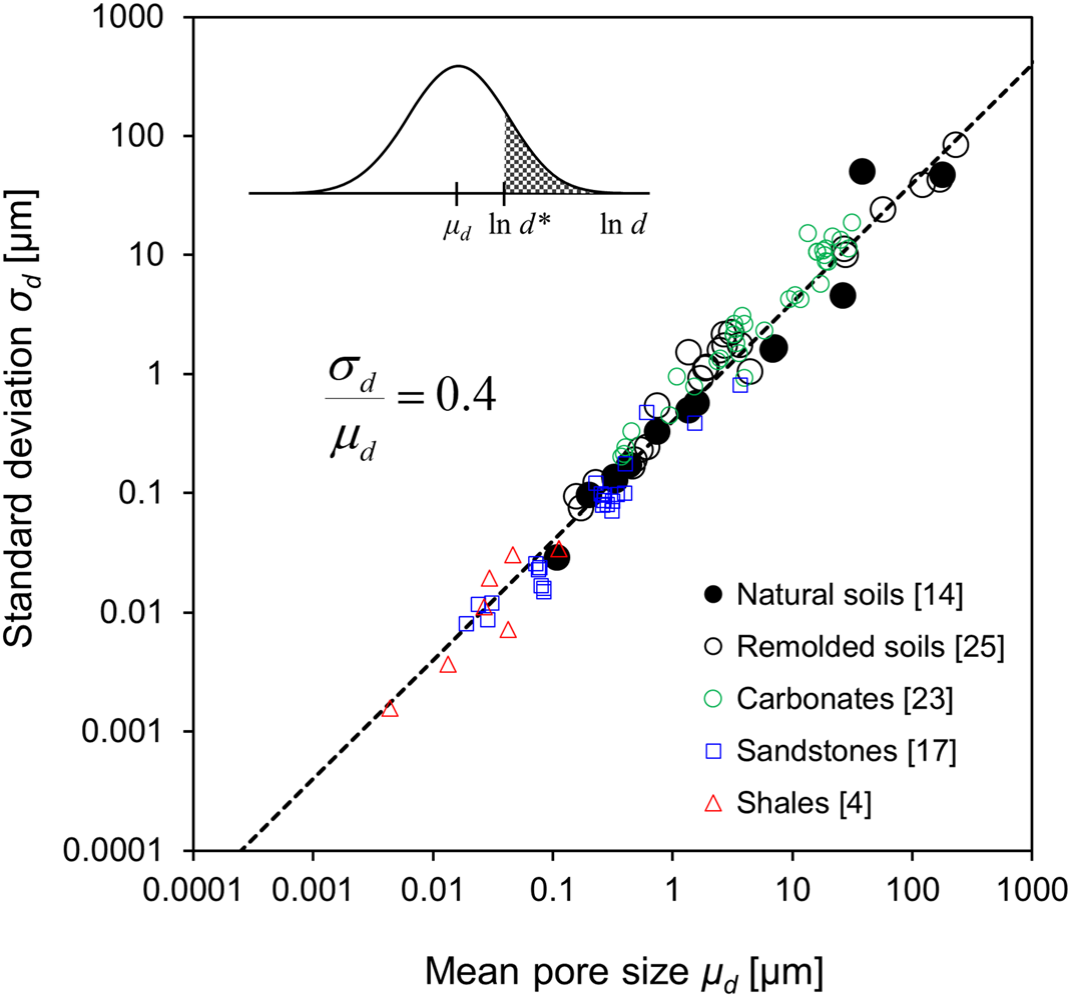
Pore size distribution for soils and rocks: standard deviation vs. mean. The number in square brackets indicates the number of datasets in each case (See Supplementary Table S2 and Figs. S2–S6). The hatched area in the inset indicates the probability of pores larger than a critical size d*.

We compiled a large database of pore size distributions measured from a wide range of soils (39 specimens) and intact rocks (44 specimens), and fitted each dataset with a log-normal distribution (Supplementary Table S2 and Figs. S2–S6). Figure 1 shows the standard deviation *σ*_*d*_ [ln(*d*/*µ*m)] plotted against the mean pore size *μ*_*d*_ [ln(*d*/*µ*m)]. The trend reveals a surprisingly strong relationship across all sediments and intact rocks: *σ*_*d*_/*μ*_*d*_ ≈ 0.4, from nm-size pores in shales to mm-size pores in sandy sediments (previously observed for a small set of sediments^18^).

### Cell count in sediments

The pore size *d* must be larger than the cell size *b* [m]. Consequently, cell counts per sediment unit volume must relate to the probability of pores *P*(*d* ≥ *b*). The probability *P*(*d* ≥ *b*) for a log-normal distribution assuming *σ*_*d*_/*μ*_*d*_ ≈ 0.4 simplifies to (see inset in Fig. 1):

6 $$ P(d \ge b) = \int\limits_{\ln b}^{\infty } {\exp \left\{ { - \frac{2.5}{4}\left[ {\ln \left( {\frac{d}{{\mu_{d} }}} \right) + 0.05} \right]^{2} } \right\}{\text{d}} d} $$

Finally, the cell count *c* [cells/cm^3^] in a sediment at depth *z* depends on: (1) the cell concentration *c*_*fl*_ [cells/cm^3^] in the pore fluid within the pores, (2) the sediment void ratio *e*_*z*_ (Eqs. 2 and 3), and (3) the probability *P*(*d* ≥ *b*) (Eq. 6) for a given mean pore size *μ*_*d*_ (Eq. 4) and standard deviation *σ*_*d*_/*μ*_*d*_ = 0.4 (Fig. 1):

7 $$ c = c_{fl} \cdot \left( {\frac{{e_{z} }}{{1 + e_{z} }}} \right) \cdot P(d \ge b) $$

where porosity *n* = *e*/(1 + *e*). We adopt a nominal cell size *b* = 1 µm for all analyses presented in this manuscript.

### Implementation

We use the effective stress dependent, asymptotically correct self-compaction model to match the reported void ratio versus depth *e*_*z*_-*z* profiles (Eqs. 1–3). The fitted asymptotic void ratio *e*_*L*_ allows the estimation of the specific surface *S*_*s*_ (Eq. 5). Then, the mean pore size *μ*_*d*_(*z*) at depth *z* is computed from the local void ratio *e*_*z*_ and the sediment specific surface *S*_*s*_ (Eq. 4, Supplementary Table S1). We complete the probabilistic pore size analysis by invoking the strong correlation *σ*_*d*_/*μ*_*d*_ = 0.4 between the mean pore size *μ*_*d*_ and the standard deviation *σ*_*d*_ (Fig. 1, Supplementary Table S2 and Figs. S2–S6). While there is variability, we adopt a single *σ*_*d*_/*μ*_*d*_ ≈ 0.4 for all analyses to avoid additional degrees of freedom (The Supplementary Fig. S7 shows the effect of *σ*_*d*_/*μ*_*d*_ on predicted cell counts—all other parameters are kept constant). Finally, we estimate cell count profiles *c(z)* [cells/cm^3^] using a single value of cell concentration in the pore fluid *c*_*fl*_ for the full profile at each site (Eq. 7).

## Results

**Figure 2:**
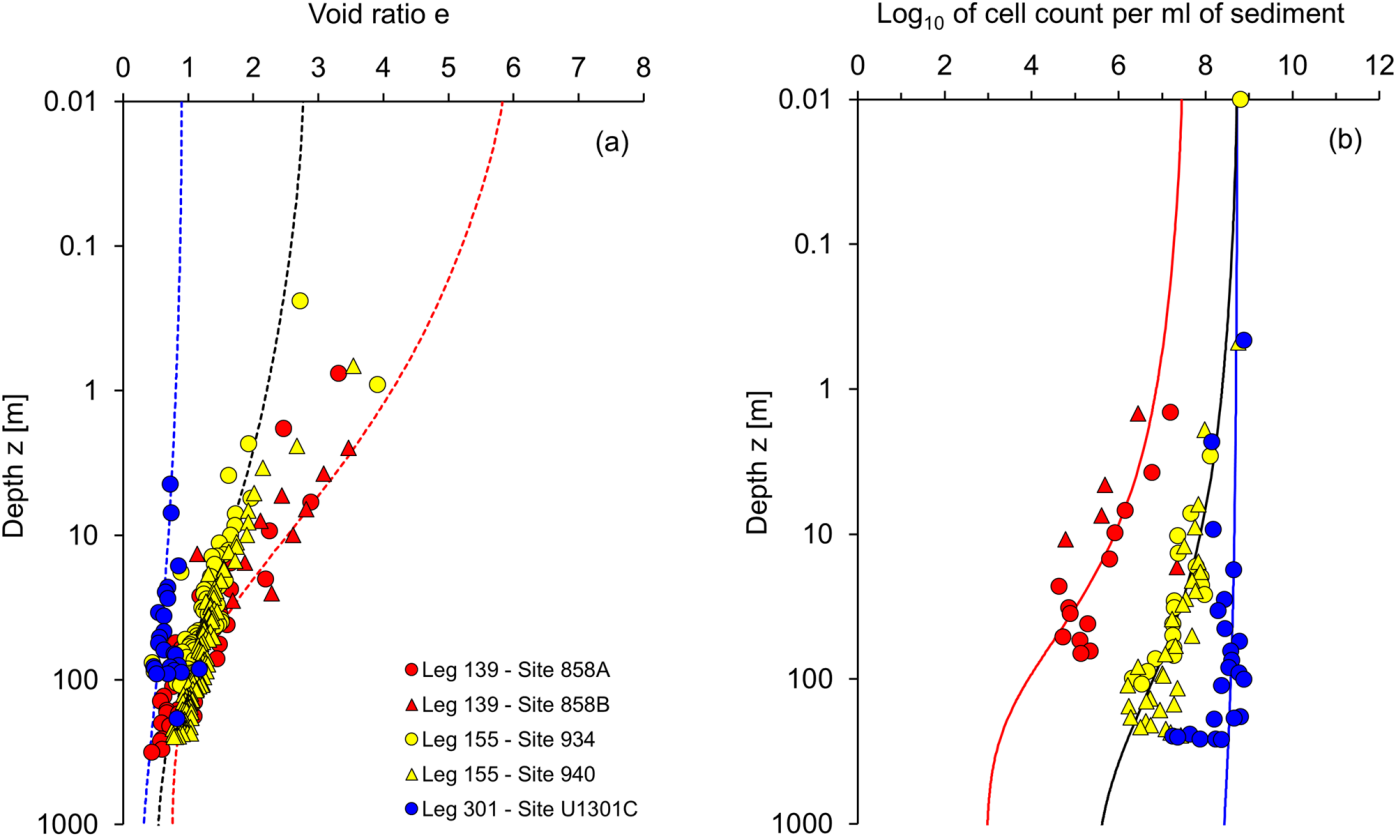
Cell count and void ratio profiles—Data and prediction models. (**a**) Void ratio depth profile. (**b**) Cell count profile. Data from Leg 139—Site 858A/B at Juan de Fuca Ridge (model parameters: *e*_*L*_ = 6.0, the estimated cell concentration of the pore fluid *c*_*fl*_ = 10^10.2^ cell counts/cm^3^), Leg 155—Site 934/940 at Amazon Fan (model parameters: *e*_*L*_ = 2.8, the estimated cell concentration of the pore fluid *c*_*fl*_ = 10^9.5^ cell counts/cm^3^), and Leg 301 Site U1301C at Juan de Fuca Ridge (model parameters: *e*_*L*_ = 0.9, the estimated cell concentration of the pore fluid *c*_*fl*_ = 10^9^ cell counts/cm^3^). See Supplementary Table S3 and Figs. S8–S121 for the complete database.

Results in Fig. 2 show the fitted compaction model and predicted cell counts for various marine sediments. Computed trends fit the compiled data well. In particular, there is a significant reduction in cell count with depth for high specific surface sediments (yellow and red data points). By contrast, pore size is not the limiting factor for microbial cell counts in silty or sandy sediments (low specific surface—blue data points). In fact, the cell count in the pore fluid *c*_*fl*_ and the sediment porosity *n* determine the cell counts in coarse-grained sediments *c* = *c*_*fl*_·[*e*_*z*_/(1 + *e*_*z*_)]  = *c*_*fl*_·*n*_*z*_ [refer to Eqs. (6) and (7)].

**Figure 3:**
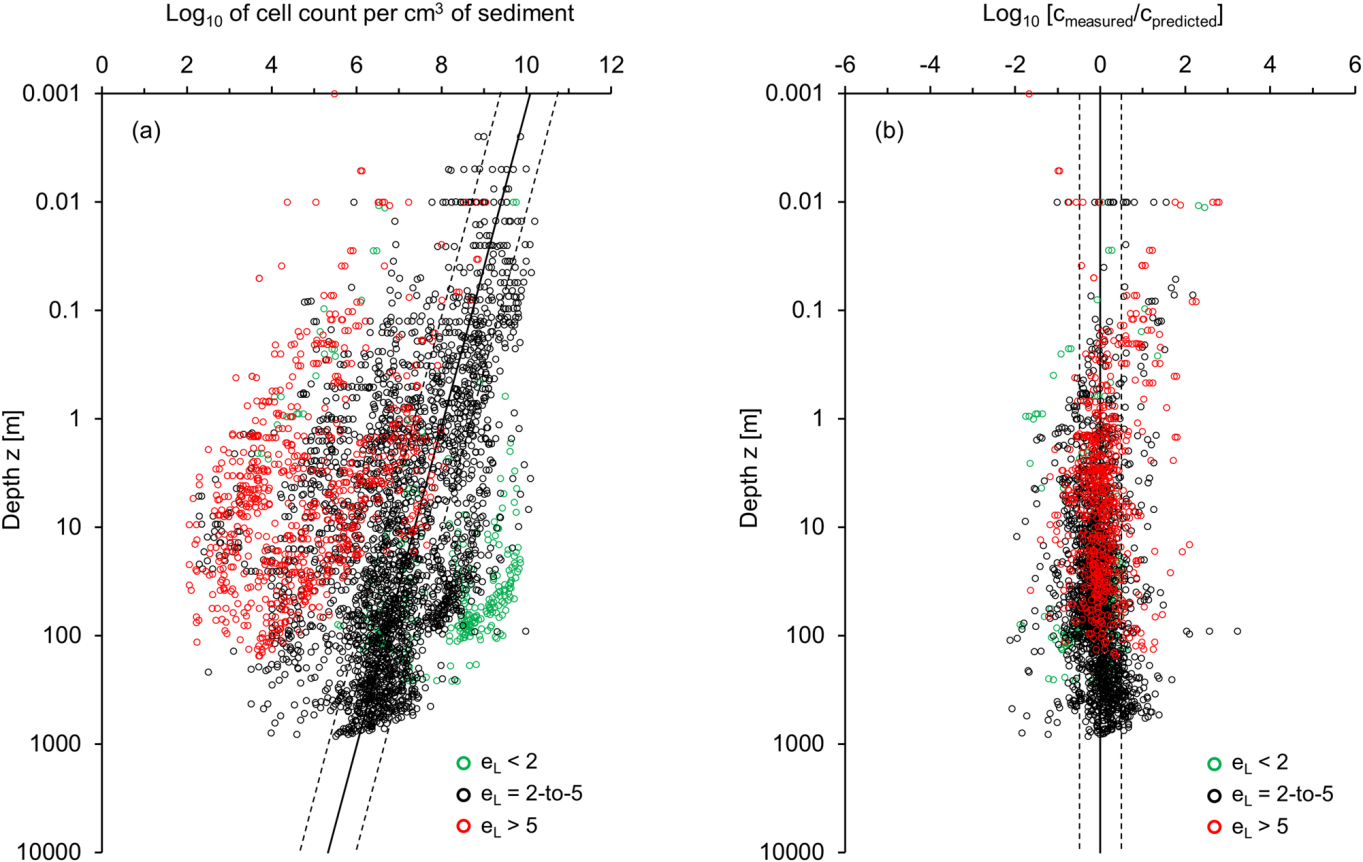
Cell count profiles for all 116 sites. (**a**) Cell counts versus depth. The black lines show a previously suggested trend^24^ log_10_ [cell counts] = 8.05–0.68·log_10_ [depth/m] based on a smaller dataset than the one considered for this study, where the dotted lines indicate 95% lower and upper prediction bounds. (**b**) Measured cell counts normalized by the predicted cell counts using Eq. (7) plotted versus sediment depth (note: dotted lines indicate standard deviation *σ* =  ± 0.52). See Supplementary Table S3 and Figs. S8–S121 for the complete database. For clarity, the cell count data in (**a**) are replotted in Supplementary Fig. S122 for the different void ratio categories.

We followed the same methodology to analyze all 116 profiles in the database (a total of 2696 measurements—Supplementary Table S3 and Figs. S8–S121). Figure 3a presents the complete dataset. We use the fitted asymptotic void ratio *e*_*L*_ to discriminate cell count profiles and cluster bio-habitats into three distinct groups: (1) green data points correspond to sandy and silty sediments (*e*_*L*_ < 2, S_s_ < 1.1 m^2^/g, and *LL* < 30), (2) black data points show intermediate plasticity sediments (*e*_*L*_ = 2–5, *S*_*s*_ = 10–70 m^2^/g, and *LL* ≈ 50-to-120), and (3) red data points represent very high plasticity clayey sediments (*e*_*L*_ > 5, S_s_ > 120 m^2^/g, and *LL* > 140). For completeness, the values in parentheses include the estimated specific surface *S*_*s*_ and liquid limit *LL*, where the liquid limit *LL* is a gravimetric water content of a water–sediment mixture at the paste-slurry transition. Data clustering by sediment type highlights the role of sediment texture and effective stress-dependent pore size on microbial cell counts in the subsurface (For clarity, cell count data for the different void ratio categories are presented in the Supplementary Fig. S122).

Cell counts in sediments vary across > 8 orders of magnitude (Fig. 3a), and the overall depth distribution deviates from previously suggested trends^4,5,24^ (black line^24^: log_10_[cell counts]  = 8.05–0.68·log_10_[depth/m]).

Figure 3b shows the ratio between measured and predicted (Eq. 7) cell counts versus depth. Data points collapse onto a single trend within ± one log cycle (standard deviation *σ* = 0.52). The contraction in the spread from Fig. 3a to Fig. 3b reflects the extent to which observed cell counts can be justified by pore size as a limiting factor. The remaining spread reflects physical factors (e.g., sediment layering and heterogeneity, non-constant cell concentration in the pore fluid *c*_*fl*_ with depth due to nutrient availability and environmental conditions such as temperature), experimental difficulties (e.g., cell counts and void ratio measurements), inherent uncertainties in the analysis and material parameters (e.g., validity of correlations, adopted nominal cell size, and correlation between *e*_*L*_ and *S*_*s*_—Eq. 5).

**Figure 4:**
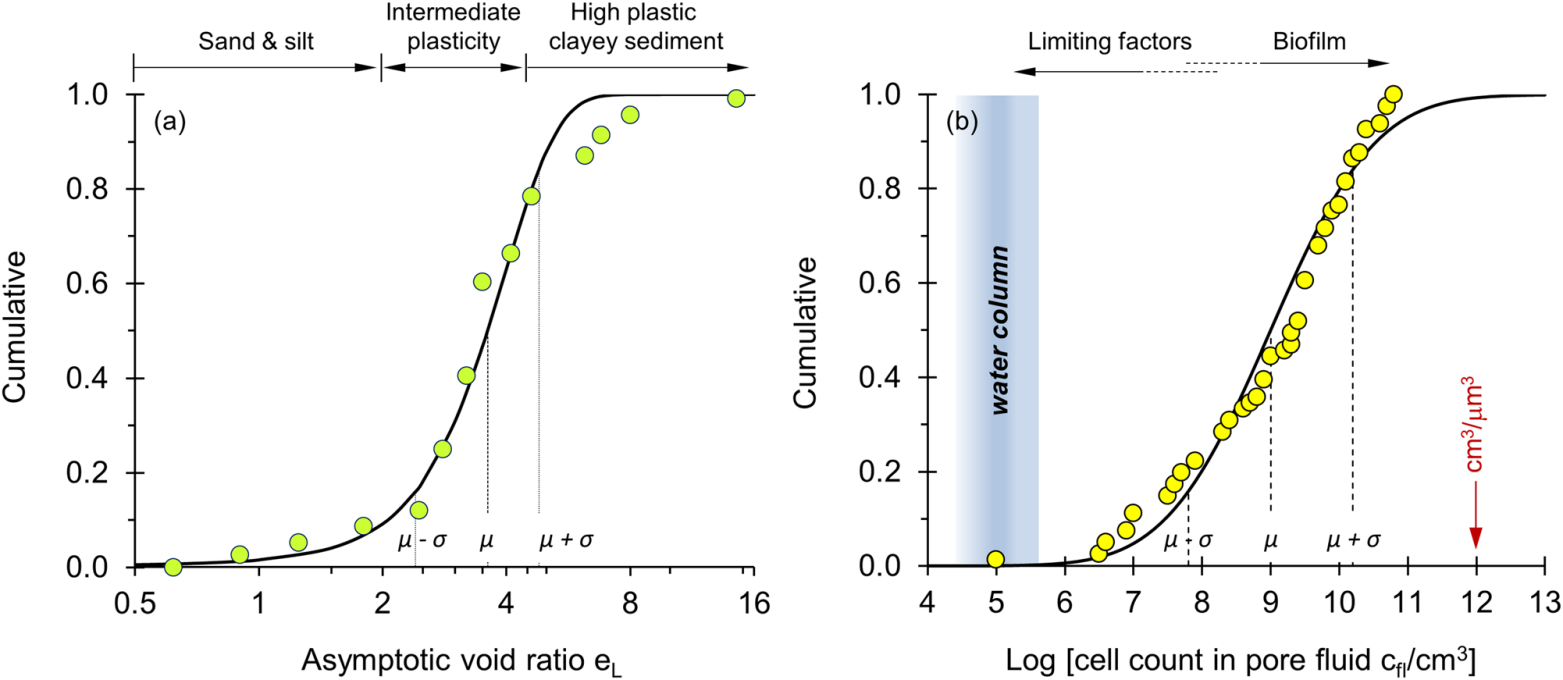
Cumulative distributions of the two key parameters fitted to the 116 depth profiles collected from ODP and IODP sites. The trend line is the Gaussian function. (**a**) Asymptotic void ratio *e*_*L*_ (mean value *µ* = 3.6 and standard deviation *σ* = 1.2). The number of sediment profiles for different ranges of the asymptotic void ratio are: 11 for *e*_*L*_ < 2, 81 for 2 < *e*_*L*_ < 5, and 24 for *e*_*L*_ > 5. (**b**) Cell concentration in the pore fluid *c*_*fl*_ for intermediate plasticity sediments (N = 81 profiles—mean value *µ* = 9.0 and standard deviation *σ* = 1.2).

Our depth dependent cell count analysis identifies two parameters of particular significance: the cell concentration in the pore fluid *c*_*fl*_ and the asymptotic void ratio *e*_*L*_, i.e., sediment type. Figure 4 presents cumulative distributions for the asymptotic void ratio *e*_*L*_ and the cell concentration in the pore fluid *c*_*fl*_ obtained by fitting the analytical model to void ratio and cell count profiles at each of the 116 sites. The fitted asymptotic void ratio values *e*_*L*_ fall between 2.4 ≤ *e*_*L*_ ≤ 4.8 for 68% of data (mean value *e*_*L*_ = 3.6—Fig. 4a). This suggests a prevalence of intermediate plasticity sediments at the studied sites.

Intermediate plasticity sediments with asymptotic void ratios in the range of 2 ≤ *e*_*L*_ ≤ 5 tend to host life with the highest cell volume density *c*_*fl*_. In these sediments, the inferred cell concentrations in the pore fluid varies between *c*_*fl*_ = 10^7.8^ and 10^10.2^ cells/cm^3^ for 68% of the data (mean value *c*_*fl*_ = 10^9^ cells/cm^3^; Fig. 4b), and can reach volume saturation levels found in dense biofilms at ~ 10^11^ cells/cm^3^. (Note: biofilm concentrations are typically reported in areal density; a high biofilm density of 10^7^ cells/cm^2^ corresponds to 10^11^ cells/cm^3^ for biofilm layers separated at 1 μm; for comparison, the packing of micron-size spheres in simple cubic configuration corresponds to the ratio cm^3^/μm^3^ that is 10^12^ cells/cm^3^). These cell counts are orders of magnitude higher than in the water column in most oceans, which ranges between 2 × 10^4^ and 5 × 10^5^ [cells/cm^3^]^9,25–27^.

## Discussion and implications

Active microorganisms require traversable pore throats larger than the nominal *b* ≈ 1 μm size^28^. Pores and pore throats also limit the advective nutrient transport. In fact, the 1 μm size correlates with a hydraulic conductivity *k*_*h*_ ≈ 0.1-to-10 cm/day^29^. We can anticipate low flow velocities *v* = *k*_*h*_·*i* given the typically low hydraulic gradients in nature *i* < 1.0; therefore, the ensuing advective-reactive regime hinders nutrient transport and bio-activity, as reported by others^2,30^.

A small fraction of fines can be sufficient to fill the pore space between coarse grains, control the pore size and eventually limit microbial cell counts. The Revised Soil Classification System RSCS recognizes the critical role of fines on mechanical and fluid flow properties in sediments^31–34^. For example, the fines fraction required to fill the pores in a sandy sediment is within the range of 12% for kaolinite, 7% for illite, and 2% for bentonite. Consequently, the detailed analysis of bioactivity in sediments must carefully consider the presence of fines and their mineralogy.

Data compiled in this study and the mecho-geometrical probabilistic analyses provide strong evidence for the critical role of pore size on microbial cell counts in sediments (together with other limiting factors such as water, carbon source, nutrients and temperature). The sediment type and effective-stress dependent pore size analysis adequately capture the decreasing cell counts with depth, and highlight the controlling role of the sediment specific surface *S*_*s*_. Similarly, pore size emerges as a critical limiting factor for life in rocks as well; for example, it is unlikely that active life will take place in the small pores of intact shales (Fig. 1), however, life may indeed thrive in large carbonate vugs. Furthermore, we expect to find microbial activity in most fractures, even at depth^35,36^. In fact, fractures can be active bio-reactors within rock masses.

## Supplementary information

Supplementary Information 1.

## References

[1] Moore, G. F., Taira, A. & Klaus, A. Shipboard scientific party. In *Proceedings**of**the**Ocean**Drilling**Program*, Initial Reports, Vol. **190** (2001).

[2] Wellsbury P, Mather I, Parkes RJ (2002). Geomicrobiology of deep, low organic carbon sediments in the Woodlark Basin, Pacific Ocean. FEMS. Microbiol. Ecol..

[3] Inagaki F, Hinrichs KU, Kubo Y, Bowles MW, Heuer VB, Hong WL, Hoshino T, Ijiri A, Imachi H, Ito M, Kaneko M (2015). Exploring deep microbial life in coal-bearing sediment down to~ 2.5 km below the ocean floor. Science.

[4] Parkes RJ, Cragg BA, Bale SJ, Getlifff JM, Goodman K, Rochelle PA, Fry JC, Weightman AJ, Harvey SM (1994). Deep bacterial biosphere in Pacific Ocean sediments. Nature.

[5] Parkes RJ, Cragg BA, Wellsbury P (2000). Recent studies on bacterial populations and processes in subseafloor sediments: a review. Hydrogeol. J..

[6] Schippers A, Neretin LN, Kallmeyer J, Ferdelman TG, Cragg BA, Parkes RJ, Jørgensen BB (2005). Prokaryotic cells of the deep sub-seafloor biosphere identified as living bacteria. Nature.

[7] Jørgensen BB, D'Hondt S (2006). A starving majority deep beneath the seafloor. Science.

[8] Jannasch HW, Wirsen CO (1973). Deep-sea microorganisms: in situ response to nutrient enrichment. Science.

[9] Whitman WB, Coleman DC, Wiebe WJ (1998). Prokaryotes: the unseen majority. Proc. Natl. Acad. Sci. USA.

[10] D’Hondt S, Jørgensen BB, Miller DJ, Batzke A, Blake R, Cragg BA, Cypionka H, Dickens GR, Ferdelman T, Hinrichs KU, Holm NG (2004). Distributions of microbial activities in deep subseafloor sediments. Science.

[11] Mitchell JK, Santamarina JC (2005). Biological considerations in geotechnical engineering. J. Geotech. Geoenviron..

[12] Pernthaler J (2005). Predation on prokaryotes in the water column and its ecological implications. Nat. Rev. Microbiol..

[13] Jørgensen BB, Boetius A (2007). Feast and famine—microbial life in the deep-sea bed. Nat. Rev. Microbiol..

[14] Fry JC, Parkes RJ, Cragg BA, Weightman AJ, Webster G (2008). Prokaryotic biodiversity and activity in the deep subseafloor biosphere. FEMS. Microbiol. Ecol..

[15] Røy H, Kallmeyer J, Adhikari RR, Pockalny R, Jørgensen BB, D’Hondt S (2012). Aerobic microbial respiration in 86-million-year-old deep-sea red clay. Science.

[16] Fredrickson JK, McKinley JP, Bjornstad BN, Long PE, Ringelberg DB, White DC, Krumholz LR, Suflita JM, Colwell FS, Lehman RM, Phelps TJ (1997). Pore size constraints on the activity and survival of subsurface bacteria in a late cretaceous shale sandstone sequence, northwestern New Mexico. Geomicrobiol. J..

[17] Bartlett R, Bottrell SH, Sinclair K, Thornton S, Fielding ID, Hatfield D (2010). Lithological controls on biological activity and groundwater chemistry in Quaternary sediments. Hydrol. Process..

[18] Phadnis HS, Santamarina JC (2011). Bacteria in sediments: pore size effects. Geotech. Lett..

[19] Rebata-Landa V, Santamarina JC (2006). Mechanical limits to microbial activity in deep sediments. Geochem. Geophys. Geosyst..

[20] Chong SH, Santamarina JC (2016). Soil compressibility models for a wide stress range. J. Geotech. Geoenviron. Eng..

[21] Lyu C, Park J, Santamarina JC (2021). Depth-dependent seabed properties: geoacoustic assessment. J. Geotech. Geoenviron. Eng..

[22] Terzariol M, Park J, Castro GM, Santamarina JC (2020). Methane hydrate-bearing sediments: Pore habit and implications. Mar. Petrol. Geol..

[23] Santamarina JC, Klein KA, Fam MA (2001). Characterization of Particles and Particulate Media in Soils and waves: Particulate Materials Behavior, Characterization and Process Monitoring.

[24] Parkes RJ, Cragg B, Roussel E, Webster G, Weightman A, Sass H (2014). A review of prokaryotic populations and processes in sub-seafloor sediments, including biosphere: geosphere interactions. Mar. Geol..

[25] Arístegui J, Gasol JM, Duarte CM, Herndld GJ (2009). Microbial oceanography of the dark ocean's pelagic realm. Limnol. Oceanogr..

[26] De Corte D, Sintes E, Yokokawa T, Reinthaler T, Herndl GJ (2012). Links between viruses and prokaryotes throughout the water column along a North Atlantic latitudinal transect. ISME. J..

[27] Mapelli F, Varela MM, Barbato M, Alvariño R, Fusi M, Álvarez M, Merlino G, Daffonchio D, Borin S (2013). Biogeography of planktonic bacterial communities across the whole Mediterranean Sea. Ocean Sci..

[28] NAE (National Academy of Engineering), "Flow and transport - Underlying processes" in *Characterization,**Modeling,**Monitoring,**and**Remediation**of**Fractured**Rock* 31–52 (The National Academies Press, Washington, DC, 2015).

[29] Ren XW, Santamarina JC (2018). The hydraulic conductivity of sediments: a pore size perspective. Eng. Geol..

[30] Boivin-Jahns V, Ruimy R, Bianchi A, Daumas S, Christen R (1996). Bacterial diversity in a deep-subsurface clay environment. Appl. Environ. Microbiol..

[31] Jang J, Santamarina JC (2016). Fines classification based on sensitivity to pore-fluid chemistry. J. Geotech. Geoenviron. Eng..

[32] Jang J, Santamarina JC (2017). Closure to “Fines classification based on sensitivity to pore-fluid chemistry” by Junbong Jang and J. Carlos Santamarina. J. Geotech. Geoenviron. Eng..

[33] Park J, Santamarina JC (2017). Revised soil classification system for coarse-fine mixtures. J. Geotech. Geoenviron. Eng..

[34] Park J, Castro GM, Santamarina JC (2018). Closure to “Revised soil classification system for coarse-fine mixtures” by Junghee Park and J. Carlos Santamarina. J. Geotech. Geoenviron. Eng..

[35] Brodsky EE, Kirkpatrick JD, Candela T (2016). Constraints from fault roughness on the scale-dependent strength of rocks. Geology.

[36] Al-Fahmi MM, Ozkaya SI, Cartwright JA (2018). New insights on fracture roughness and wall mismatch in carbonate reservoir rocks. Geosphere..

