# Supplementary material for: The critical role of pore size on depth-dependent microbial cell counts in sediments
Source: Sci Rep. 2020 Dec 10;10:21692. doi: 10.1038/s41598-020-78714-3 (PMC7729949; doi:10.1038/s41598-020-78714-3)
Supplement: Supplementary file 1 — Supplementary Information 1. [file 41598_2020_78714_MOESM1_ESM.pdf]

Supplementary Information for

The critical role of pore size on depth-dependent microbial cell counts in sediments

Junghee Park, J. Carlos Santamarina*

*Corresponding author (Email: carlos.santamarina@kaust.edu.sa)

Table of Contents

Notation

Supplementary Tables S1 to S3

Supplementary Figures S1 to S122

References

Notation.

e_L	void ratio at low effective stress $\sigma_z'\rightarrow 0$
e_H	void ratio at high effective stress $\sigma_z'\rightarrow\infty$
σ'_c	characteristic effective stress
η	model parameter (= 1/3 in this study)
g	gravity g = 9.81 m/s^2
ρ_w	water density (this study: ρ_w= 1000 kg/m^3)
ρ_m	mineral density (this study: ρ_m= 2650 kg/m^3)
G_s	specific gravity $G_s=\rho_m/\rho_w$
d	pore size
D	grain diameter
b	cell size
μ_d	mean value of pore size distribution
k	fabric factor
c	cell count per cm^3 of sediment
c_{fl}	cell count per cm^3 in the bulk pore fluid
n	porosity
$P(d \geq b)$	probability of fining pore sizes larger than the cell size

Supplementary Table S1. Mean pore size as a function of void ratio e and specific surface S_s (Refs. 1, 2).

		Fabric	Void ratio e	Specific surface S_s	Mean pore size μ_d
Mean pore size as a function of void ratio and specific surface	Clay	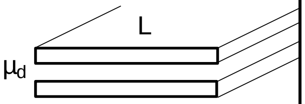	$e_z = \frac{V_{void}}{V_{soil}} = \frac{\mu_d \cdot L \cdot \infty}{V_{soil}}$	$S_s = \frac{2 \cdot L \cdot \infty}{V_{soil}\rho_m}$	$\mu_d = \frac{2e_z}{S_s\rho_m}$ $(k = 2)$
		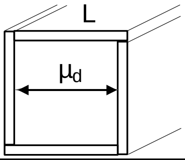	$e_z = \frac{V_{void}}{V_{soil}} = \frac{\mu_d{}^2 \infty}{V_{soil}}$	$S_s = \frac{4 \cdot \mu_d \cdot \infty}{V_{soil}\rho_m}$	$\mu_d = \frac{4e_z}{S_s\rho_m}$ $(k = 4)$
		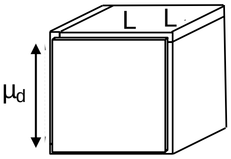	$e_z = \frac{V_{void}}{V_{soil}} = \frac{\mu_d{}^3}{V_{soil}}$	$S_s = \frac{6\mu_d{}^2}{V_{soil}\rho_m}$	$\mu_d = \frac{6e_z}{S_s\rho_m}$ $(k = 6)$
	Sand	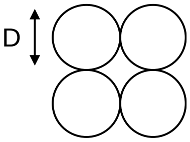	$e_z = \frac{V_{void}}{V_{soil}} = \frac{6}{\pi} - 1$	$S_s = \frac{6}{D\rho_m}$	$\mu_d = (\sqrt{2} - 1)D \approx \frac{2.4}{S_s\rho_m}$
		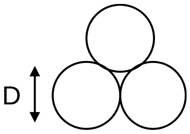	$e_z = \frac{V_{void}}{V_{soil}} = \frac{3\sqrt{2}}{\pi} - 1$	$S_s = \frac{6}{D\rho_m}$	$\mu_d = \left(\frac{2}{\sqrt{3}} - 1\right)D \approx \frac{0.92}{S_s\rho_m}$

Note: Consider parallel platelets of thickness t separated by a gap μ_d. The void ratio e_z is the volume of voids V_{void} normalized by the volume of solids V_{solid}. Thus, the void ratio is $e_z = \mu_d/t$. On the other hand, the specific surface S_s is the ratio between the surface area of particles A_s and their mass m; then, the specific surface is $S_s = 2/(t\rho_m)$. Finally, the separation between platelets is $\mu_d = 2e_z/(S_s\rho_m)$. Similar analysis can be extended to other fabrics. In all cases, $d = k \cdot e_z/(S_s\rho_m)$ where $k = 4$ is the structure factor in this study.

Supplementary Table S2. Pore size distribution for soils and rocks – Data and sources. Note: The number in [ ] indicates the size of datasets.

Type	No.	Descriptions	Mean	Standardard. dev.	References
			μ_d [μm]	σ_d [μm]	
Natural soils [14]	1	Bothkennar clay	1.58	0.57	(Ref. 3)
	2	Osaka clay (Ma11)	0.43	0.17	(Ref. 3)
	3	Kyoto clay (Ma4)	0.33	0.13	(Ref. 3)
	4	Osaka clay (Ma13)	1.36	0.49	(Ref. 3)
	5	Soil A	178.4	47.2	(Ref. 4)
	6	GB-0.2	38.5	50.4	(Ref. 5)
	7	Pusan clay 1	0.33	0.14	(Ref. 6)
	8	Pusan clay 5	0.75	0.33	(Ref. 6)
	9	Fontainebleau	6.87	1.60	(Ref. 7)
	10	Brown Agra	7.14	1.67	(Ref. 7)
	11	green Agra	0.11	0.03	(Ref. 7)
	12	Roche fine	26.4	4.53	(Ref. 7)
	13	Liais	0.20	0.10	(Ref. 7)
	14	St Guillaume clay	0.14	0.2	(Ref. 8)
Remolded soils [25]	1	Soil B	121.3	38.5	(Ref. 4)
	2	Soil C	27.7	10.0	(Ref. 4)
	3	Soil A - High	169.2	42.9	(Ref. 4)
	4	Soil A - Low	233.0	84.1	(Ref. 4)
	5	Soil C – Low	27.1	11.3	(Ref. 4)
	6	Soil C - High	57.4	23.9	(Ref. 4)
	7	Pusan clay 2	0.48	0.19	(Ref. 6)
	8	Pusan clay 3	0.61	0.24	(Ref. 6)
	9	Pusan clay 4	0.54	0.23	(Ref. 6)
	10	St Guillaume clay	0.40	0.40	(Ref. 8)
	11	Silty clay 1	2.50	1.58	(Ref. 9)
	12	Silty clay 2	2.74	1.73	(Ref. 9)
	13	Silty clay 3	3.67	1.74	(Ref. 9)
	14	Silty clay 4	4.46	1.04	(Ref. 9)
	15	Glacial till 17%	2.70	2.15	(Ref. 10)
	16	Glacial till 8%	1.91	1.14	(Ref. 10)
	17	Glacial 17% (After SWCC)	0.16	0.09	(Ref. 10)
	18	Glacial 17% (Before SWCC)	1.36	1.52	(Ref. 10)
	19	Sample mta-1	1.88	1.12	(Ref. 11)
	20	Kaolin	0.17	0.08	(Ref. 12)
	21	Soil 1 (30kPa)	0.46	0.17	(Ref. 13)
	22	Soil 1 (LL)	1.72	0.92	(Ref. 13)
	23	Soil 2 (LL)	0.23	0.12	(Ref. 13)
	24	Soil 4 (LL)	3.09	2.25	(Ref. 13)
	25	Soil 4 (120kP)	0.75	0.54	(Ref. 13)

Supplementary Table S2. Pore size distribution for soils and rocks – Data and sources (Continued). Note: The number in [ ] indicates the size of datasets.

Type	No.	Descriptions	Mean	Standardard. dev.	References
			μ_d [μm]	σ_d [μm]	
Carbonates [23]	1	Winterset	1.53	0.77	(Ref. 14)
	2	Austin Chalk	1.08	0.94	(Ref. 15)
	3	Desert Pink	5.88	2.31	(Ref. 16)
	4	Edwards White	0.95	0.45	(Ref. 17)
	5	Edward Yellow	3.96	0.92	(Ref. 17)
	6	Indiana 2-4	0.45	0.33	(Ref. 18)
	7	Indiana 60 (1)	0.39	0.21	(Ref. 18)
		Indiana 60 (2)	13.5	15.1	(Ref. 18)
	8	Indiana 70 (1)	25.1	13.4	(Ref. 18)
		Indiana 70 (2)	0.37	0.20	(Ref. 18)
	9	Indiana 200 (1)	31.5	18.7	(Ref. 18)
		Indiana 200 (2)	0.41	0.24	(Ref. 18)
	10	Mount Gambier	29.6	11.3	(Ref. 19)
	11	Silurian Dolomite	11.7	4.23	(Ref. 20)
	12	Carbonate-1 (1)	19.0	8.80	(Ref. 21)
		Carbonate-1 (2)	3.59	1.49	(Ref. 21)
	13	Carbonate-2	9.41	4.24	(Ref. 21)
	14	Carbonate-3 (1)	10.6	4.54	(Ref. 21)
		Carbonate-3 (2)	2.52	1.34	(Ref. 21)
	15	Carbonate-4	19.1	11.3	(Ref. 21)
	16	Carbonate-5 (1)	16.1	10.6	(Ref. 21)
		Carbonate-5 (2)	2.39	1.27	(Ref. 21)
	17	Carbonate-6 (1)	16.1	10.6	(Ref. 21)
		Carbonate-6 (2)	3.40	1.81	(Ref. 21)
	18	Carbonate-7 (1)	19.8	8.71	(Ref. 21)
		Carbonate-7 (2)	3.30	2.62	(Ref. 21)
	19	Carbonate-8 (1)	16.1	10.6	(Ref. 21)
		Carbonate-8 (2)	3.83	3.05	(Ref. 21)
	20	Carbonate-9 (1)	21.7	14.3	(Ref. 21)
		Carbonate-9 (2)	3.25	2.14	(Ref. 21)
	21	Carbonate-10 (1)	18.1	10.8	(Ref. 21)
		Carbonate-10 (2)	3.25	2.14	(Ref. 21)
	22	Carbonate-11 (1)	17.3	5.68	(Ref. 21)
		Carbonate-11 (2)	3.33	2.37	(Ref. 21)
	23	Carbonate-12 (1)	18.6	9.93	(Ref. 21)
		Carbonate-12 (2)	3.97	2.61	(Ref. 21)

Supplementary Table S2. Pore size distribution for soils and rocks – Data and sources (Continued). Note: The number in [ ] indicates the size of datasets.

Type	No.	Descriptions	Mean	Standardard. dev.	References
			μ_d [μm]	σ_d [μm]	
Sandstones [17]	1	C11	0.603	0.479	(Ref. 22)
	2	C29	0.228	0.121	(Ref. 22)
	3	No.1	0.023	0.011	(Ref. 23)
	4	No.2	0.267	0.096	(Ref. 23)
	5	No.3	0.030	0.012	(Ref. 23)
	6	No.4 (1)	0.264	0.087	(Ref. 23)
		No.4 (2)	0.071	0.025	(Ref. 23)
	7	No.5 (1)	0.283	0.080	(Ref. 23)
		No.5 (2)	0.075	0.023	(Ref. 23)
	8	No.6 (1)	0.342	0.097	(Ref. 23)
		No.6 (2)	0.077	0.023	(Ref. 23)
	9	No.7	0.395	0.100	(Ref. 23)
	10	No.8	0.019	0.008	(Ref. 23)
	11	No.9	0.252	0.099	(Ref. 23)
	12	No.10	0.028	0.008	(Ref. 23)
	13	No.11	0.312	0.085	(Ref. 23)
	14	No.12 (1)	1.523	0.387	(Ref. 23)
		No.12 (2)	3.648	0.812	(Ref. 23)
	15	No.13 (1)	0.257	0.079	(Ref. 23)
		No.13 (2)	0.083	0.015	(Ref. 23)
	16	No.14 (1)	0.309	0.072	(Ref. 23)
		No.14 (2)	0.079	0.016	(Ref. 23)
	17	No.15 (1)	0.401	0.176	(Ref. 23)
		No.15 (2)	0.083	0.016	(Ref. 23)
Shales [4]	1	North Sea shale (1)	0.042	0.007	(Ref. 24)
		North Sea shale (2)	0.013	0.003	(Ref. 24)
	2	Mancos B (1)	0.046	0.030	(Ref. 24)
		Mancos B (2)	0.112	0.034	(Ref. 24)
	3	Middle Bakken (1)	0.029	0.019	(Ref. 24)
		Middle Bakken (2)	0.112	0.034	(Ref. 24)
	4	Woodford shale (1)	0.004	0.001	(Ref. 24)
		Woodford shale (2)	0.026	0.011	(Ref. 24)

Supplementary Table S3. Summary – ODP, IODP and other expeditions.

No.	Location	Leg	Void ratio	Cell counts	References
1	Deep Peru Margin	112	680A/B	680C	(Refs. 25, 26)
2	Deep Peru Margin	112	681A/B	681C	(Refs. 26, 27)
3	Japan Sea	128	798A/B	798B	(Refs. 28, 29)
4	Lau Basin	135	834A	834A	(Refs. 30, 31)
5	Eastern Equatorial Pacific	138	851A	851A	(Refs. 32, 33)
6	Eastern Equatorial Pacific	138	851B	851B	(Refs. 32, 33)
7	Juan de Fuca Ridge	139	857A/C	857A	(Refs. 34, 35)
8	Juan de Fuca Ridge	139	858A	858A	(Refs. 35, 36)
9	Juan de Fuca Ridge	139	858B	858B	(Refs. 35, 36)
10	Juan de Fuca Ridge	139	858C	858C	(Refs. 35, 36)
11	Cascadia Margin (Part 1)	146	888B	888B	(Refs. 37, 38)
12	Cascadia Margin (Part 1)	146	889A	889A	(Refs. 38, 39)
13	Cascadia Margin (Part 1)	146	890B	890B	(Refs. 38, 39)
14	Cascadia Margin (Part 1)	146	891A	891A	(Refs. 38, 40)
15	Cascadia Margin (Part 1)	146	891B	891B	(Refs. 38, 40)
16	Cascadia Margin (Part 1)	146	892A	892A	(Refs. 38, 41)
17	Cascadia Margin (Part 1)	146	892D	892D	(Refs. 38, 41)
18	Santa Barbara Basin (Part 2)	146	893	893	(Refs. 42, 43)
19	Amazon Fan	155	934	934	(Refs. 44, 45)
20	Amazon Fan	155	940	940	(Refs. 45, 46)
21	Eastern Mediterranean	160	969A	969A	(Refs. 47, 48)
22	Alboran Sea	161	976B	976B	(Refs. 49, 50)
23	Alboran Sea	161	977A	977A	(Refs. 50, 51)
24	Alboran Sea	161	978A	978A	(Refs. 50, 52)
25	Blake Ridge	164	994C	994	(Refs. 53, 54)
26	Blake Ridge	164	995A	995	(Refs. 54, 55)
27	Blake Ridge	164	997	997	(Refs. 54, 56)
28	Juan de Fuca Ridge	168	1026A/C	1026	(Refs. 57, 58)
29	Juan de Fuca Ridge	168	1027B	1027	(Refs. 57, 58)
30	Northeast Pacific	169	1035A	1035A	(Refs. 59, 60)
31	Northeast Pacific	169	1036A	1036A	(Refs. 60, 61)
32	Northeast Pacific	169	1036B	1036B	(Refs. 60, 61)
33	Northeast Pacific	169	1036C	1036C	(Refs. 60, 61)
34	Saanich Inlet, BC	169 [S]	1033B	1033B	(Refs. 62, 63)
35	Saanich Inlet, BC	169 [S]	1034B	1034B	(Refs. 62, 63)
36	Sourthern Ocean	177	1089	1088	(Refs. 64, 65)
37	Sourthern Ocean	177	1090	1093	(Refs. 65, 66)
38	Woodlark Basin	180	1108B	1108B	(Ref.67)
39	Woodlark Basin	180	1109B/C/D	1109B/C/D	(Ref. 68)
40	Woodlark Basin	180	1110B	1110B	(Ref. 69)
41	Woodlark Basin	180	1111A	1111A	(Ref. 69)
42	Woodlark Basin	180	1112A	1112A	(Ref. 69)
43	Woodlark Basin	180	1115A/B/C	1115	(Ref. 70)
44	Woodlark Basin	180	1118	1118	(Ref. 71)

Supplementary Table S3. Summary – ODP, IODP and small-scale expeditions (Continued).

No.	Location	Leg	Void ratio	Cell counts	References
45	Nankai Trough	190	1173A	1173A	(Ref. 72)
46	Nankai Trough	190	1174	1174	(Ref. 73)
47	Nankai Trough	190	1175	1175	(Ref. 74)
48	Nankai Trough	190	1176	1176	(Ref. 75)
49	Nankai Trough	190	1177	1177	(Ref. 76)
50	Nankai Trough	190	1178	1178	(Ref. 77)
51	Peru margin	201	1225A	1225A	(Ref. 78)
52	Peru margin	201	1226B	1226B	(Ref. 79)
53	Peru margin	201	1227A	1227A	(Ref. 80)
54	Peru margin	201	1229A	1229A	(Ref. 81)
55	Peru margin	201	1230	1230	(Ref. 82)
56	Peru margin	201	1231B	1231B	(Ref. 83)
57	Cascadia Margin	204	1244A/B/C/E	1244/1245	(Refs. 84, 85)
58	Cascadia Margin	204	1245B/C/E	1244/1245	(Refs. 85, 86)
59	Cascadia Margin	204	1251B	1251B	(Refs. 85, 87)
60	Juan de Fuca Ridge	301	U1301C	U1301C	(Ref. 88)
61	Lomonosov Ridge (Arctic)	302	M0002A	M0002A	(Refs. 89, 90)
62	Porcupine Seamount	307	U1316A	U1316A	(Ref. 91)
63	Porcupine Seamount	307	U1317	U1317	(Ref. 92)
64	Porcupine Seamount	307	U1318	U1318	(Ref. 93)
65	Gulf of Mexico (Brazos)	308	U1319	U1319	(Ref. 94)
66	Gulf of Mexico (Brazos)	308	U1320	U1320	(Ref. 95)
67	Gulf of Mexico (Brazos)	308	U1322B	U1322B	(Ref. 96)
68	Gulf of Mexico (Brazos)	308	U1324B	U1324B	(Ref. 97)
69	Pacific Equatorial	320/321	U1331	U1331	(Refs. 98, 99)
70	Pacific Equatorial	320/321	U1332	U1332	(Refs. 99, 100)
71	Pacific Equatorial	320/321	U1334	U1334	(Refs. 99, 101)
72	Pacific Equatorial	320/321	U1335	U1335	(Refs. 99, 102)
73	Pacific Equatorial	320/321	U1337	U1337	(Refs. 99, 103)
74	Pacific Equatorial	320/321	U1338	U1338	(Refs. 99, 104)
75	Bering Sea	323	U1342C	U1342	(Refs. 105, 106)
76	Bering Sea	323	U1343A/E	U1343	(Refs. 106, 107)
77	South Pacific Gyre	329	SPG1 M1	SPG1	(Ref. 108)
78	South Pacific Gyre	329	SPG2 P1	SPG2	(Ref. 108)
79	South Pacific Gyre	329	SPG3 P1	SPG3	(Ref. 108)
80	South Pacific Gyre	329	SPG4 M1/P1	SPG4	(Ref. 108)
81	South Pacific Gyre	329	SPG5 M1/P1	SPG5	(Ref. 108)
82	South Pacific Gyre	329	SPG6 P2	SPG6	(Ref. 108)
83	South Pacific Gyre	329	SPG7 M1	SPG7	(Ref. 108)
84	South Pacific Gyre	329	SPG9 P1	SPG9	(Ref. 108)
85	South Pacific Gyre	329	SPG10 P2	SPG10	(Ref. 108)
86	South Pacific Gyre	329	SPG11 TG2	SPG11	(Ref. 108)
87	South Pacific Gyre	329	SPG12 TG2	SPG12	(Ref. 108)
88	South Pacific Gyre	329	U1365	U1365	(Ref. 109)

Supplementary Table S3. Summary – ODP, IODP and small-scale expeditions (Continued).

No.	Location	Leg	Void ratio	Cell counts	References
89	South Pacific Gyre	329	U1366	U1366	(Ref. 110)
90	South Pacific Gyre	329	U1367	U1367	(Ref. 111)
91	South Pacific Gyre	329	U1368	U1368	(Ref. 112)
92	South Pacific Gyre	329	U1369	U1369	(Ref. 113)
93	South Pacific Gyre	329	U1370	U1370	(Ref. 114)
94	South Pacific Gyre	329	U1371	U1371	(Ref. 115)
95	Mid-Okinawa Trough	331	C0013C-to-F	C0013	(Ref. 116)
96	Mid-Okinawa Trough	331	C0014A-to-G	C0014B/D/E/G	(Ref. 117)
97	Mid-Okinawa Trough	331	C0015	C0015B/C	(Ref. 118)
98	Mid-Okinawa Trough	331	C0017	C0017A/B/C/D	(Ref. 119)
99	Mid-Atlantic Ridge	336	1704A/1382/1383	Geob 13501	(Refs.120-122, 123)
100	Mid-Atlantic Ridge	336	1704A/1382/1383	Geob 13502	(Refs.120-122, 123)
101	Mid-Atlantic Ridge	336	1704A/1382/1383	Geob 13504	(Refs.120-122, 123)
102	Mid-Atlantic Ridge	336	1704A/1382/1383	Geob 13507	(Refs.120-122, 123)
103	Mid-Atlantic Ridge	336	1704A/1382/1383	Geob 13510	(Refs.120-122, 123)
104	Mid-Atlantic Ridge	336	1704A/1382/1383	Geob 13512	(Refs.120-122, 123)
105	Shimokita	337	C0020	C0020	(Ref. 124)
106	Baltic Sea Basin	347	M0059D	M0059C	(Ref. 125)
107	Baltic Sea Basin	347	M0059D	M0059E	(Ref. 125)
108	Baltic Sea Basin	347	M0060B	M0060B	(Ref. 126)
109	Baltic Sea Basin	347	N/A	M0061K	(Ref. 127)
110	Baltic Sea Basin	347	M0063E	M0063E	(Ref. 128)
111	Baltic Sea Basin	347	N/A	M0065C	(Ref. 129)
112	Sea of Okhotsk		MD 01-2412	MD 01-2412	(Ref. 130)
113	Chesapeake Bay		N/A	Chesapeake Bay	(Ref. 131)
114	New Caledonia		N/A	MD 06-3019	(Ref. 132)
115	North Sea tidal flat		N/A	North Sea	(Ref. 133)
116	SO 189 Forearc off Sumatra		N/A	6KL	(Ref. 134)

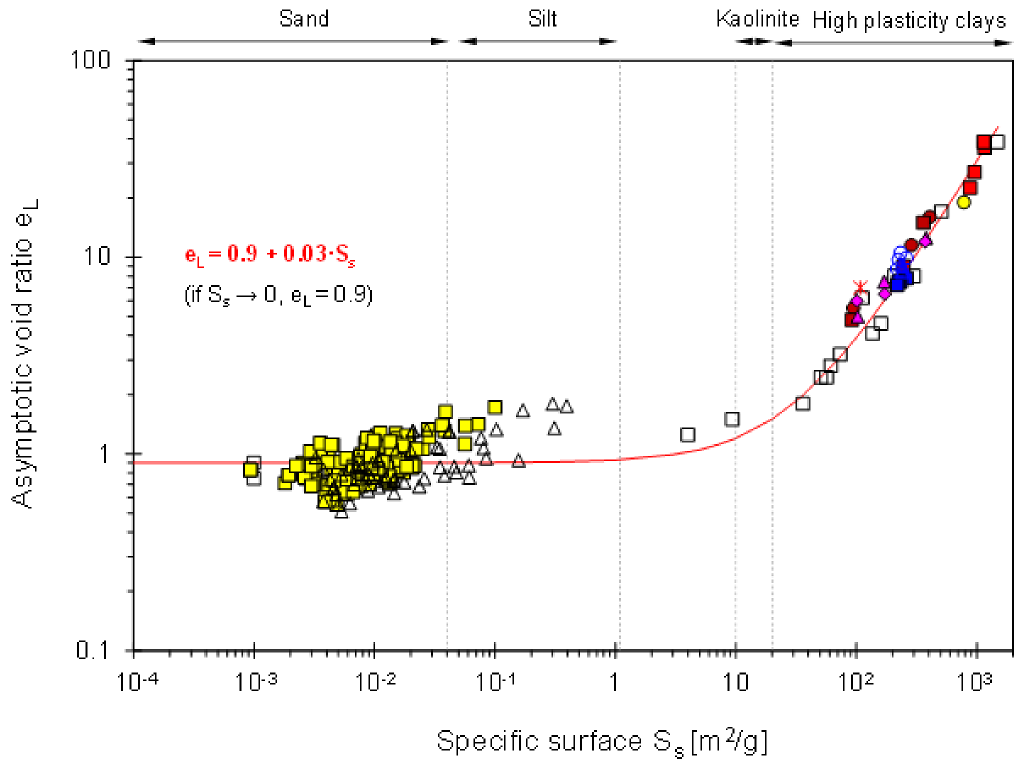

Supplementary Figure S1. Asymptotic void ratio e_L for coarse- and fine-grained soils as a function of specific surface S_s. Asymptotic void ratio e_L is determined by using the compaction model for clayey sediments (Ref. 135) and maximum void ratio e_{max} for sand and silt sediments (Data source - Ref. 136).

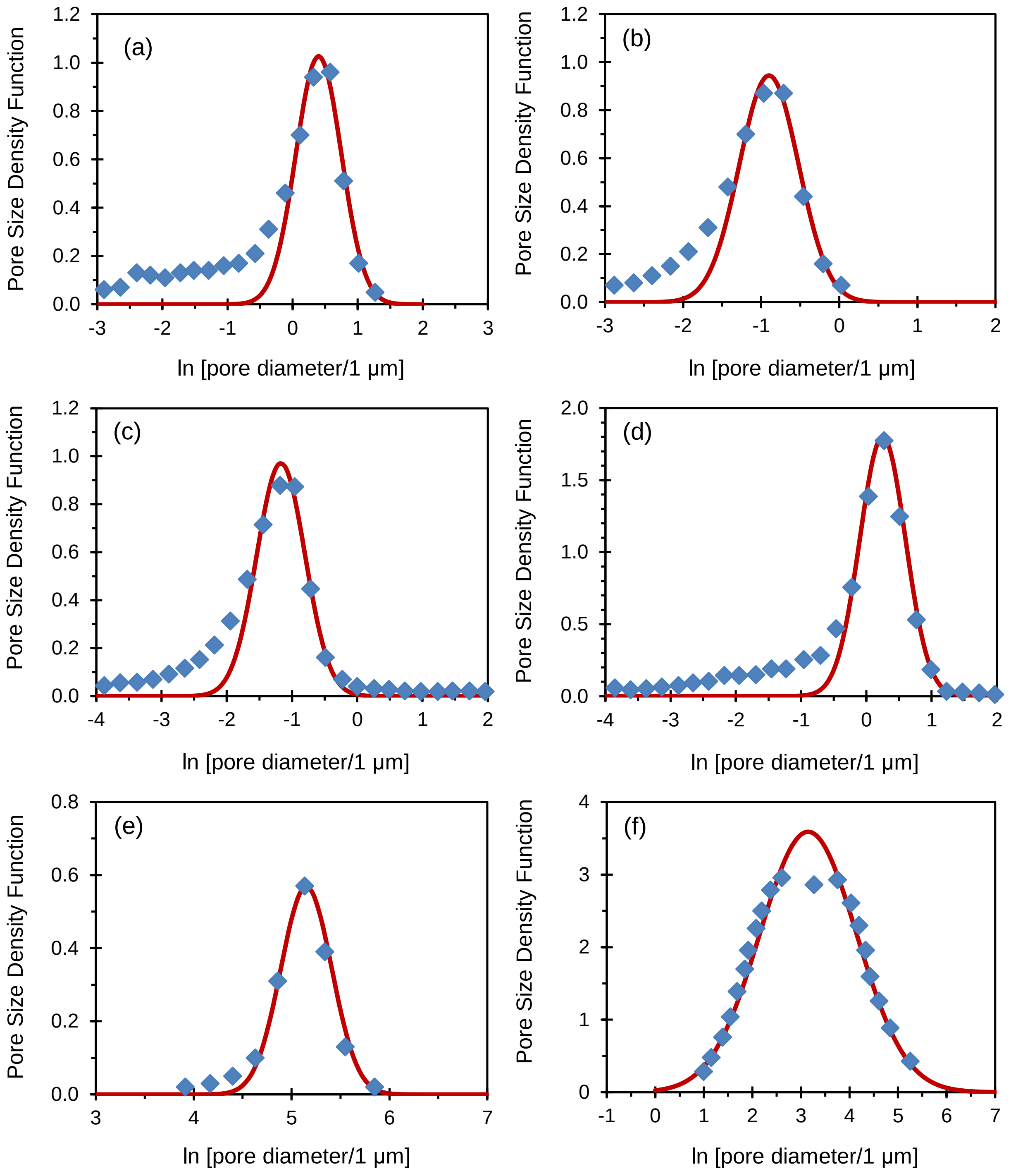

Supplementary Figure S2. Pore size distribution – Natural soils. (a) Bothkennar clay, (b) Osaka bay Pieistocene clay (Ma11), (c) Kyoto clay (Ma4), (d) Osaka clay (Ma 13), (e) Soil A, and (f) GB-0.2 (Supplementary Table 2 - Refs. 3-5).

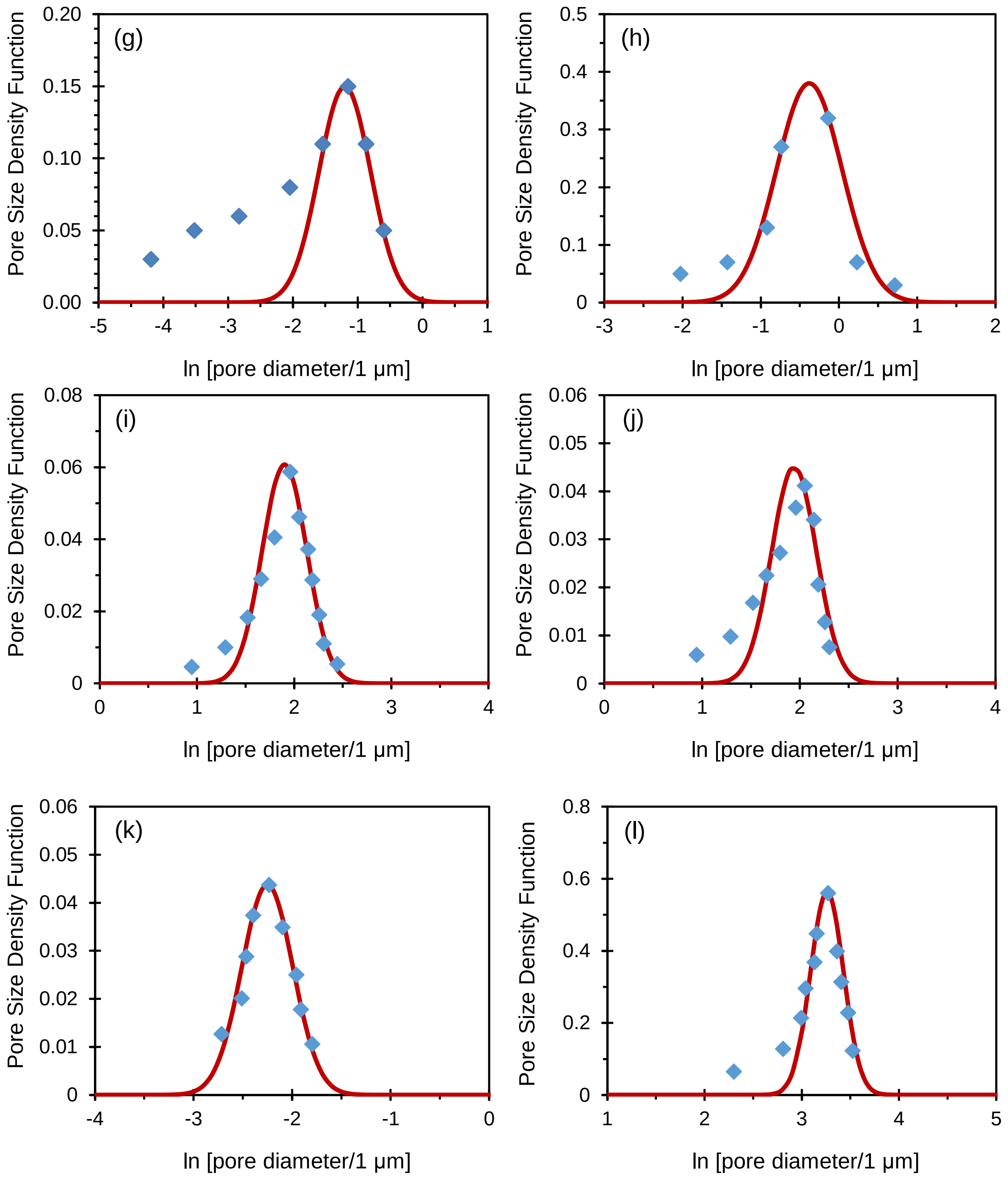

Supplementary Figure S2. Pore size distribution – Natural soils (Continued). (g) Pusan clay 1, (h), Pusan clay 5, (i) Fontainebleau, (j), Brown Agra, (k), Green Agra, and (l) Roche fine (Supplementary Table 2 - Refs. 6, 7).

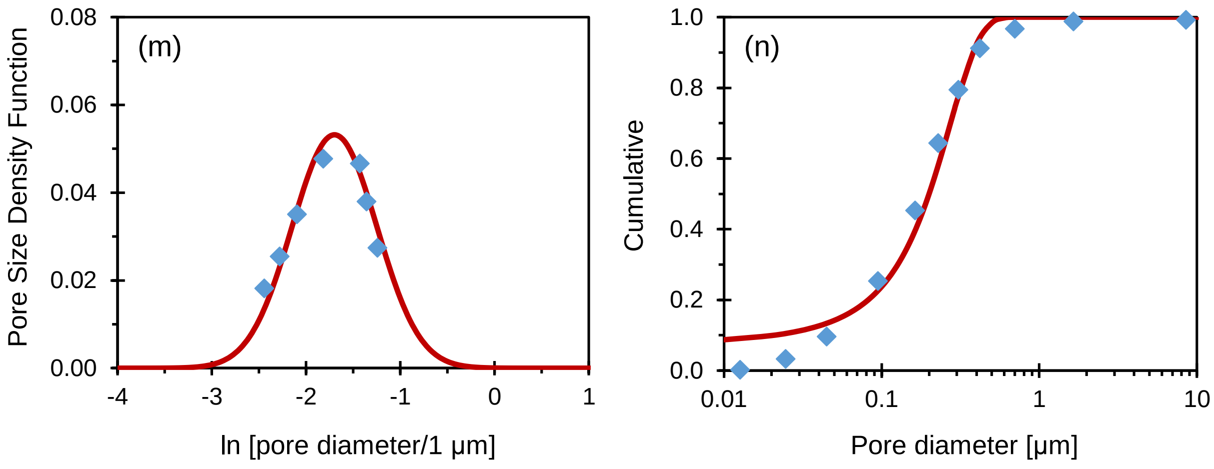

Supplementary Figure S2. Pore size distribution – Natural soils (Continued). (m) Liais, (n) St Guillaume clay (Supplementary Table 2 - Refs. 7, 8).

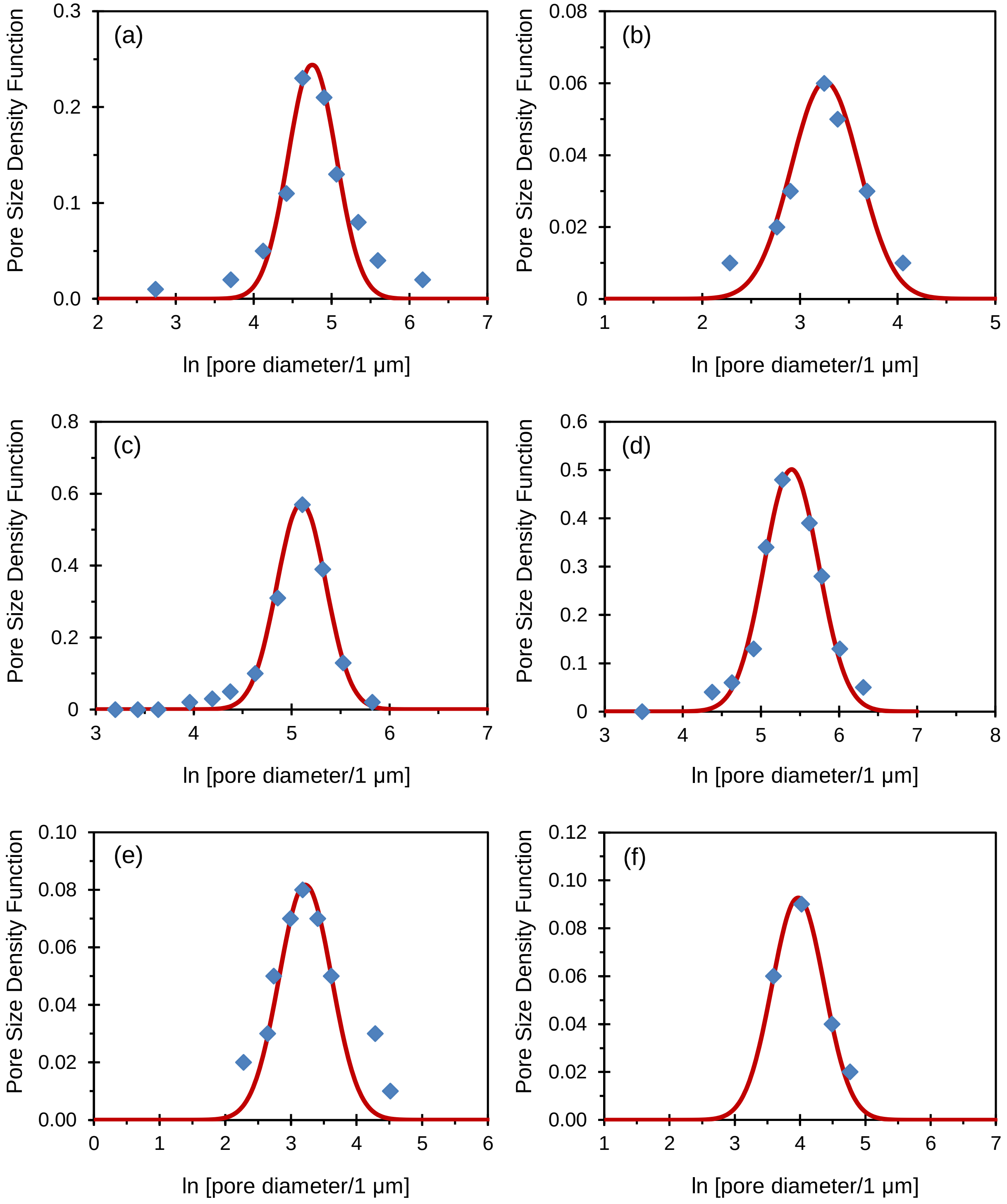

Supplementary Figure S3. Pore size distribution – Remolded soils. (a) Soil B, (b) Soil C, (c) Soil A - High, (d) Soil A - Low, (e) Soil C - Low, and (f) Soil C – High (Supplementary Table 2 - Ref. 4).

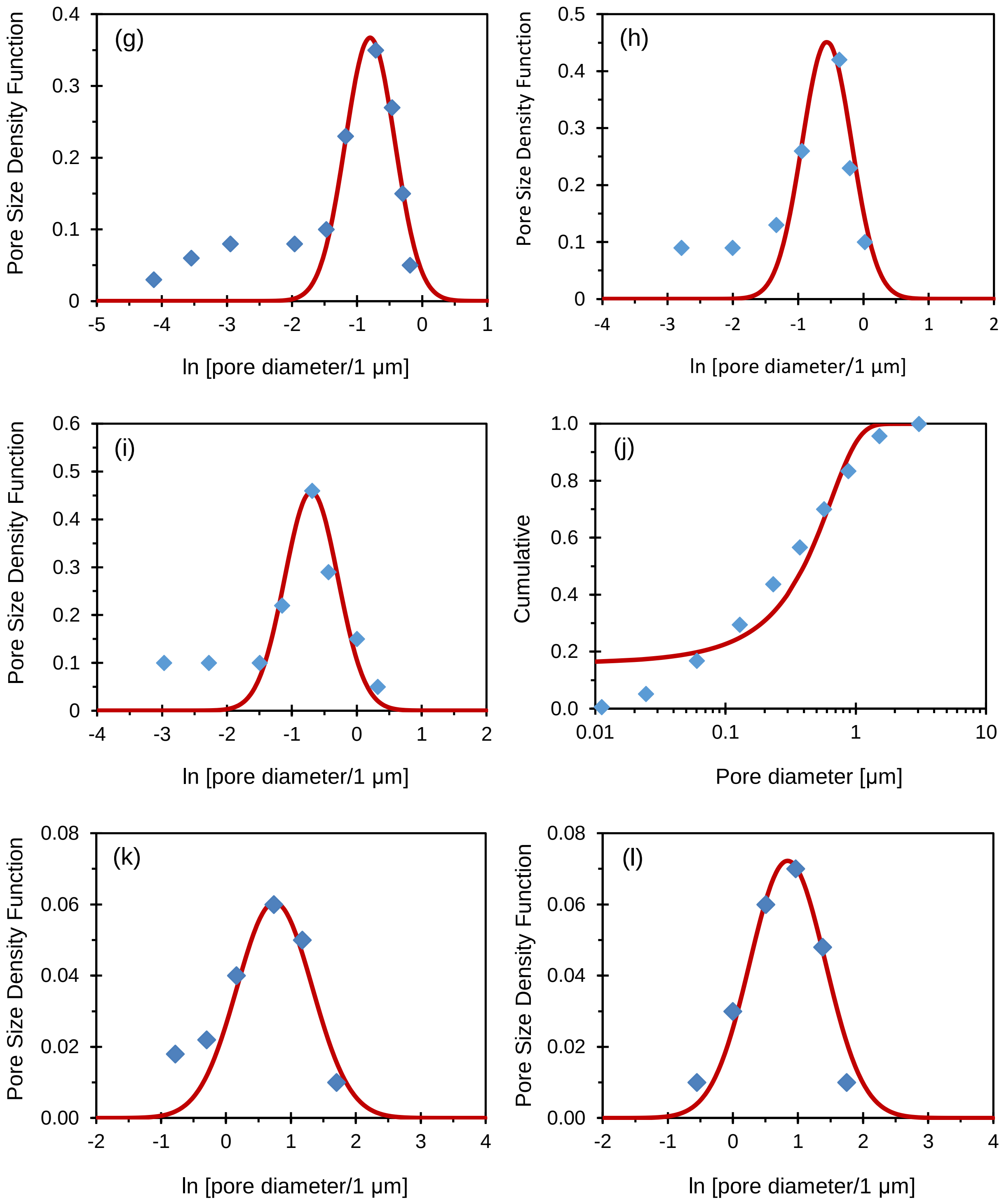

Supplementary Figure S3. Pore size distribution – Remolded soils (Continued). (g) Pusan clay 2, (h) Pusan clay 3, (i) Pusan clay 4, (j) St Guillaume clay, (k) Silty clay 1, and (l) Silty clay 2 (Supplementary Table 2 - Refs. 6, 8, 9).

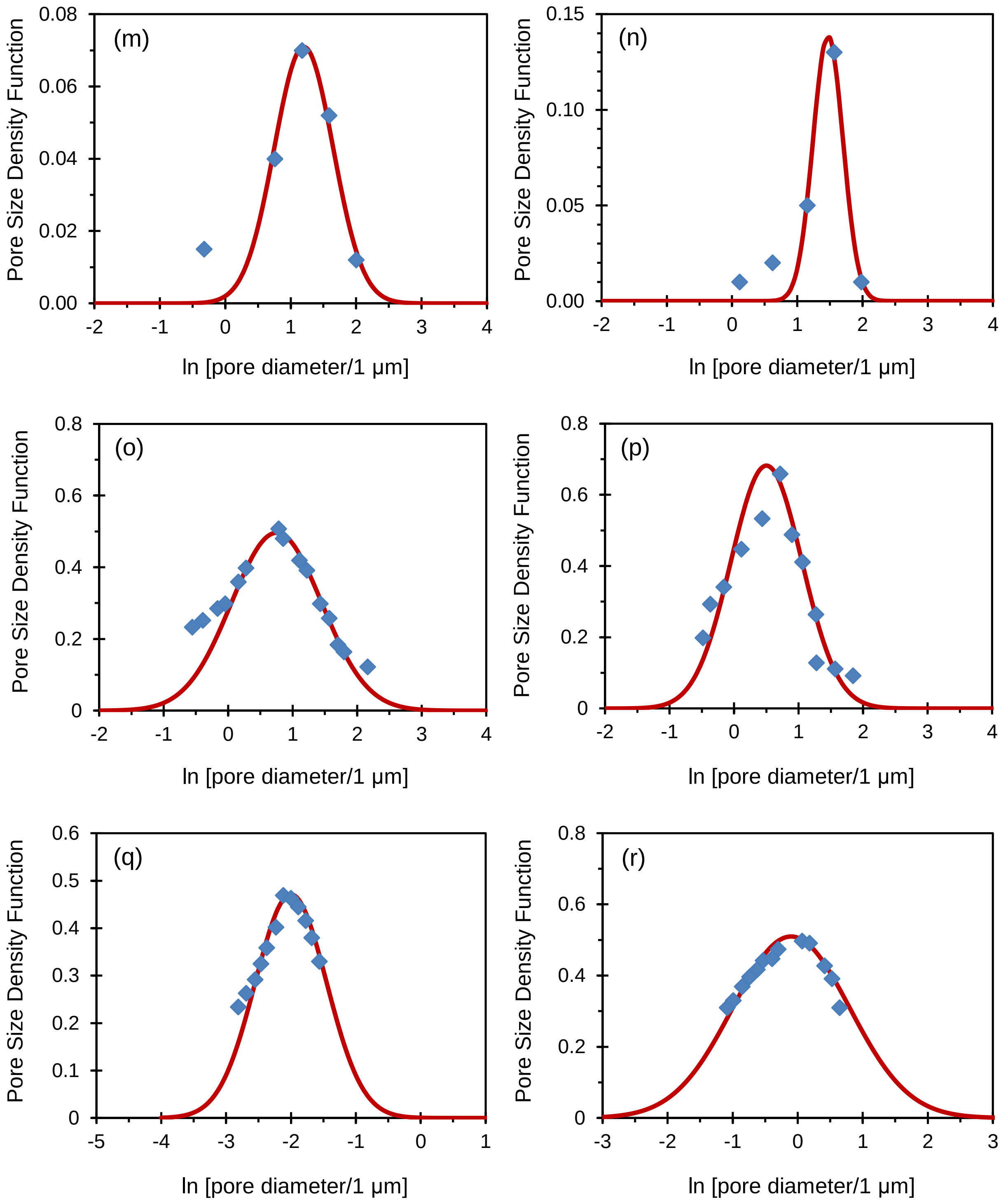

Supplementary Figure S3. Pore size distribution – Remolded soils (Continued). (m) Silty clay 3, (n) Silty clay 4, (o) Glacial till 17%, (p) Glacial till 8%, (q) Glacial 17% (After SWCC), and (r) Glacial 17% (Before SWCC) (Supplementary Table 2 - Refs. 9, 10).

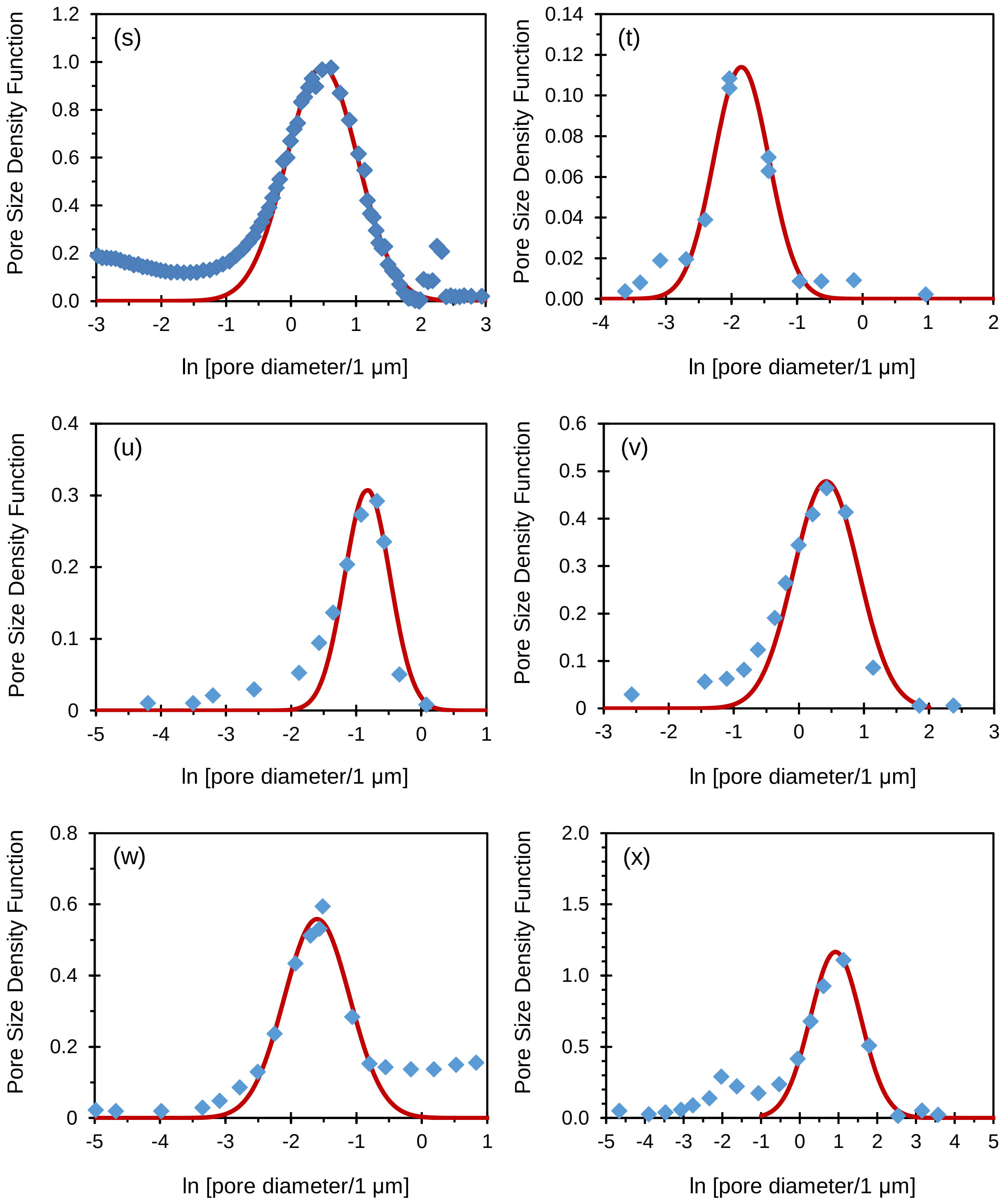

Supplementary Figure S3. Pore size distribution – Remolded soils (Continued). (s) Sample mta-1, (t) Kaolin, (u) Soil 1 (30kPa), (v) Soil 1 (LL), (w) Soil 2 (LL), and (x) Soil 4 (LL) (Supplementary Table 2 - Refs. 11-13).

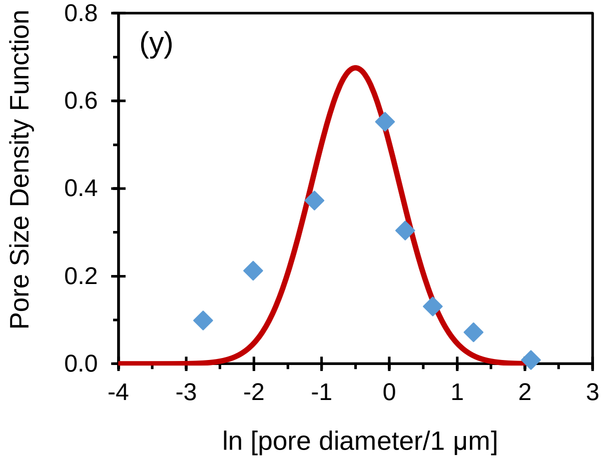

Supplementary Figure S3. Pore size distribution – Remolded soils (Continued). (y) Soil 4 (120kP) (Supplementary Table 2 – Ref. 13).

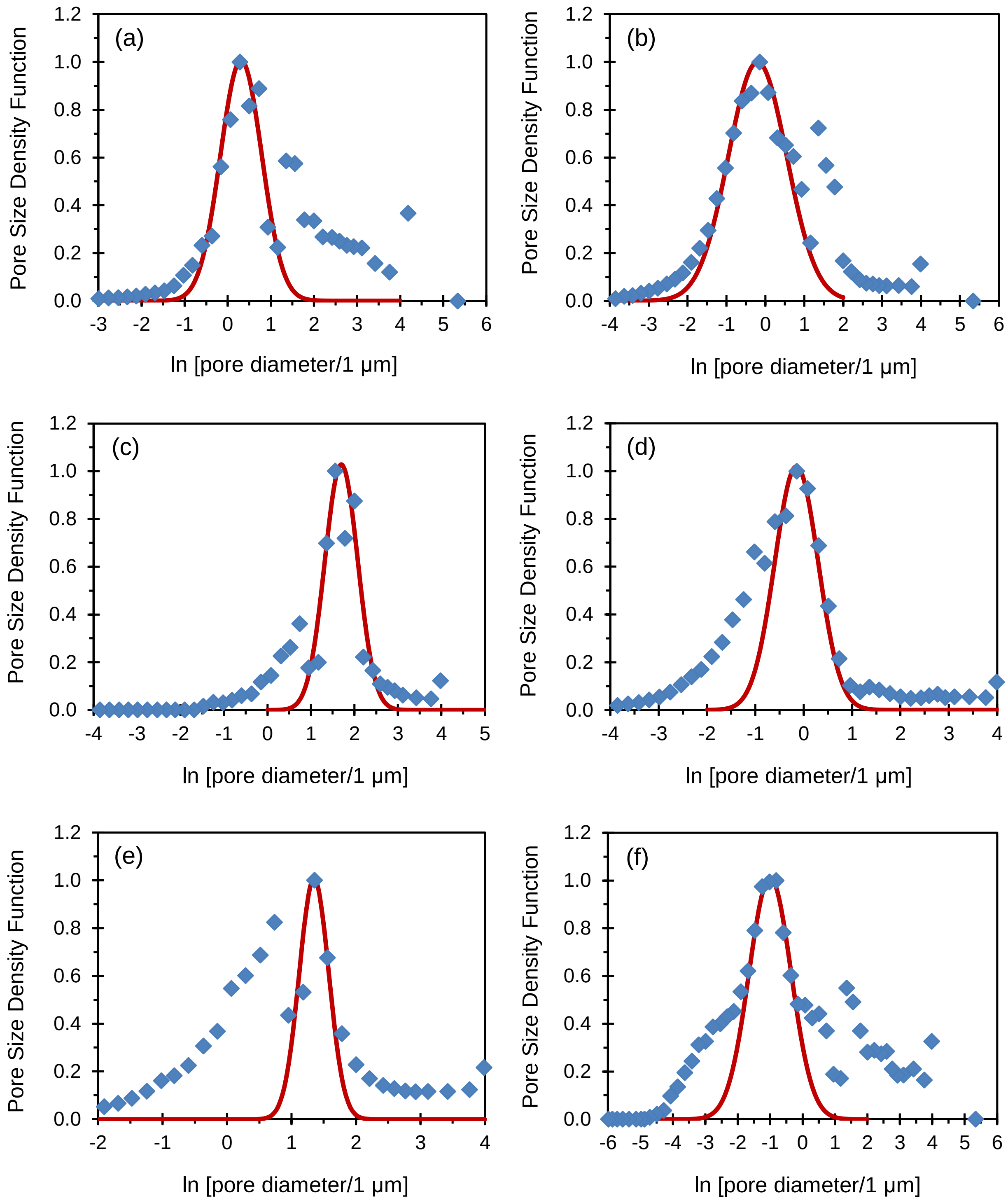

Supplementary Figure S4. Pore size distribution – Carbonates. (a) Winterset, (b) Austin Chalk, (c) Desert Pink, (d) Edwards White, (e) Edward Yellow, and (f) Indiana 2-4 (Supplementary Table 2 – Refs. 14-18).

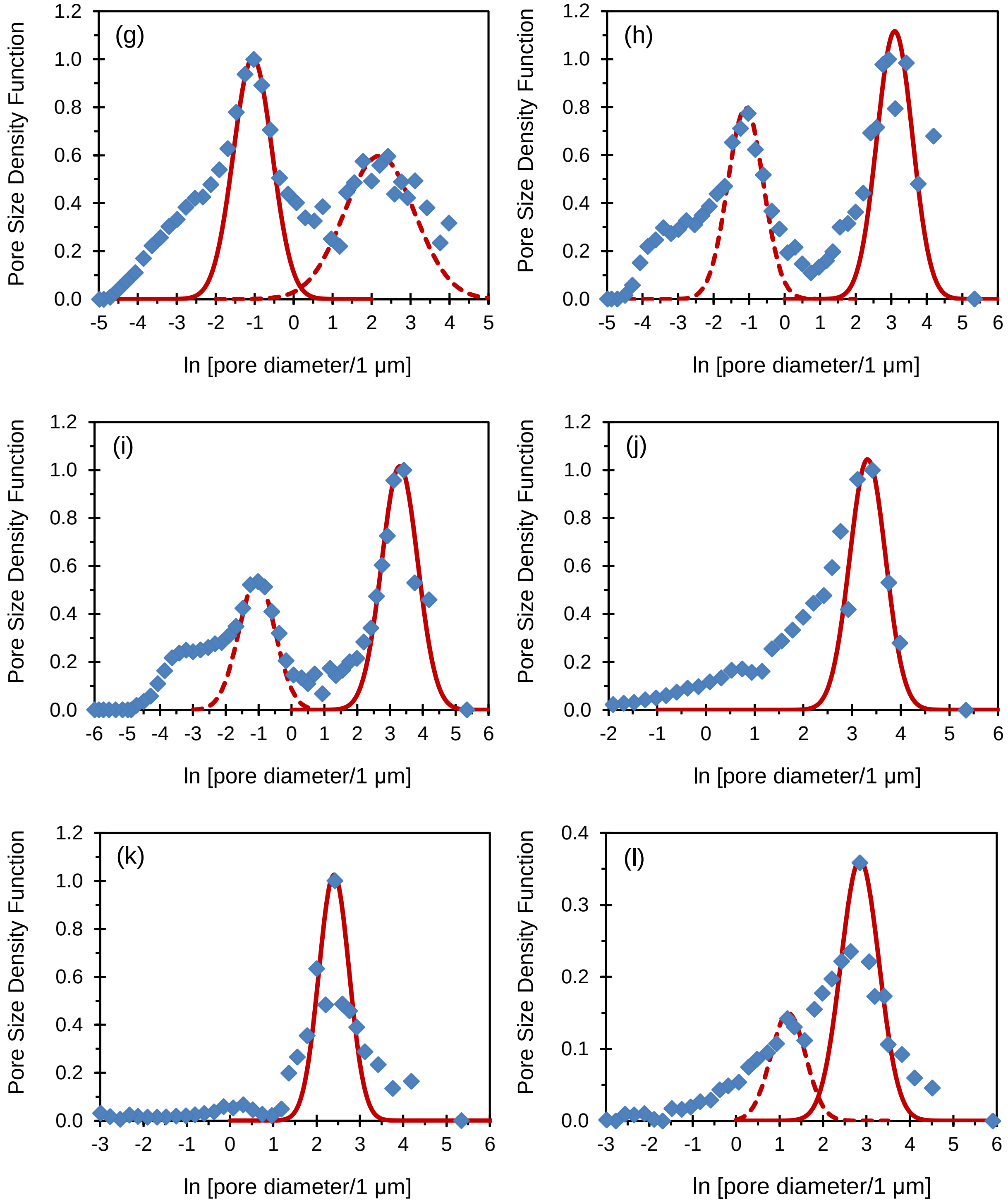

Supplementary Figure S4. Pore size distribution – Carbonates (Continued). (g) Indiana 60, (h) Indiana 70, (i) Indiana 200, (j) Mount Gambier, (k) Silurian Dolomite, and (l) Carbonate-1 (Supplementary Table 2 – Refs. 18-21).

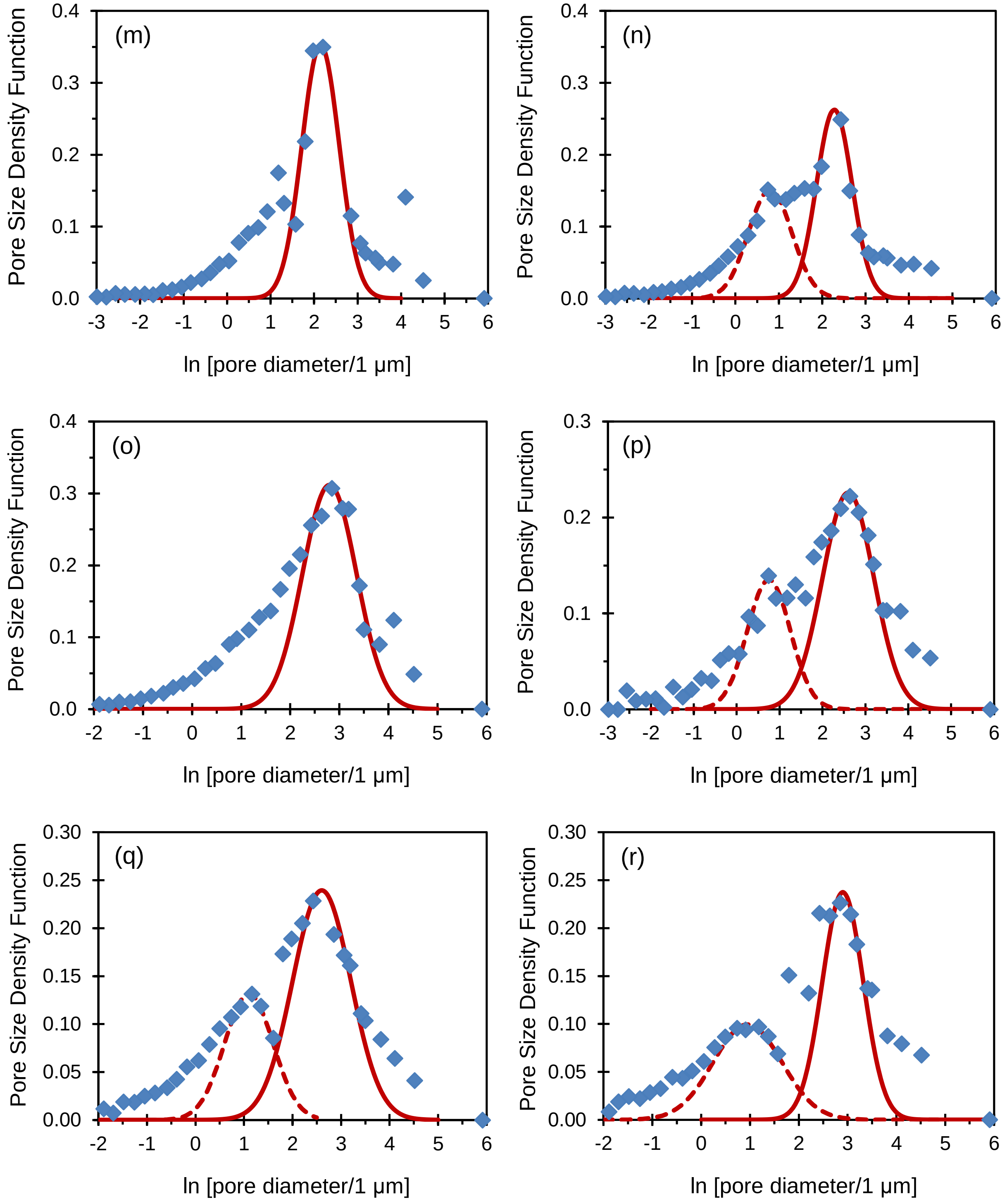

Supplementary Figure S4. Pore size distribution – Carbonates (Continued). (m) Carbonate-2, (n) Carbonate-3, (o) Carbonate-4, (p) Carbonate-5, (q) Carbonate-6, and (r) Carbonate-7 (Supplementary Table 2 – Ref. 21).

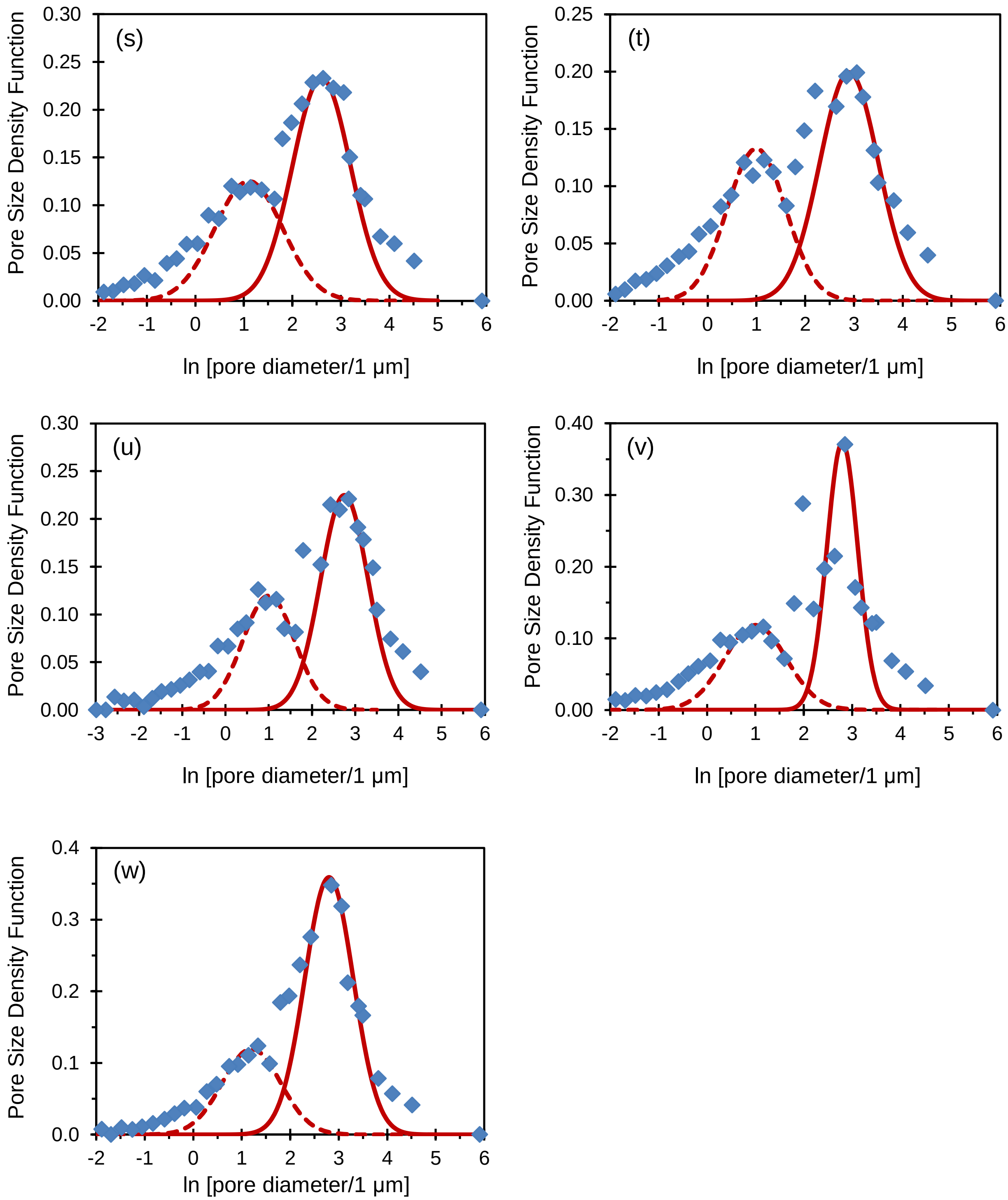

Supplementary Figure S4. Pore size distribution – Carbonates (Continued). (s) Carbonate-8, (t) Carbonate-9, (u) Carbonate-10, (v) Carbonate-11, (w) Carbonate-12 (Supplementary Table 2 – Ref. 21).

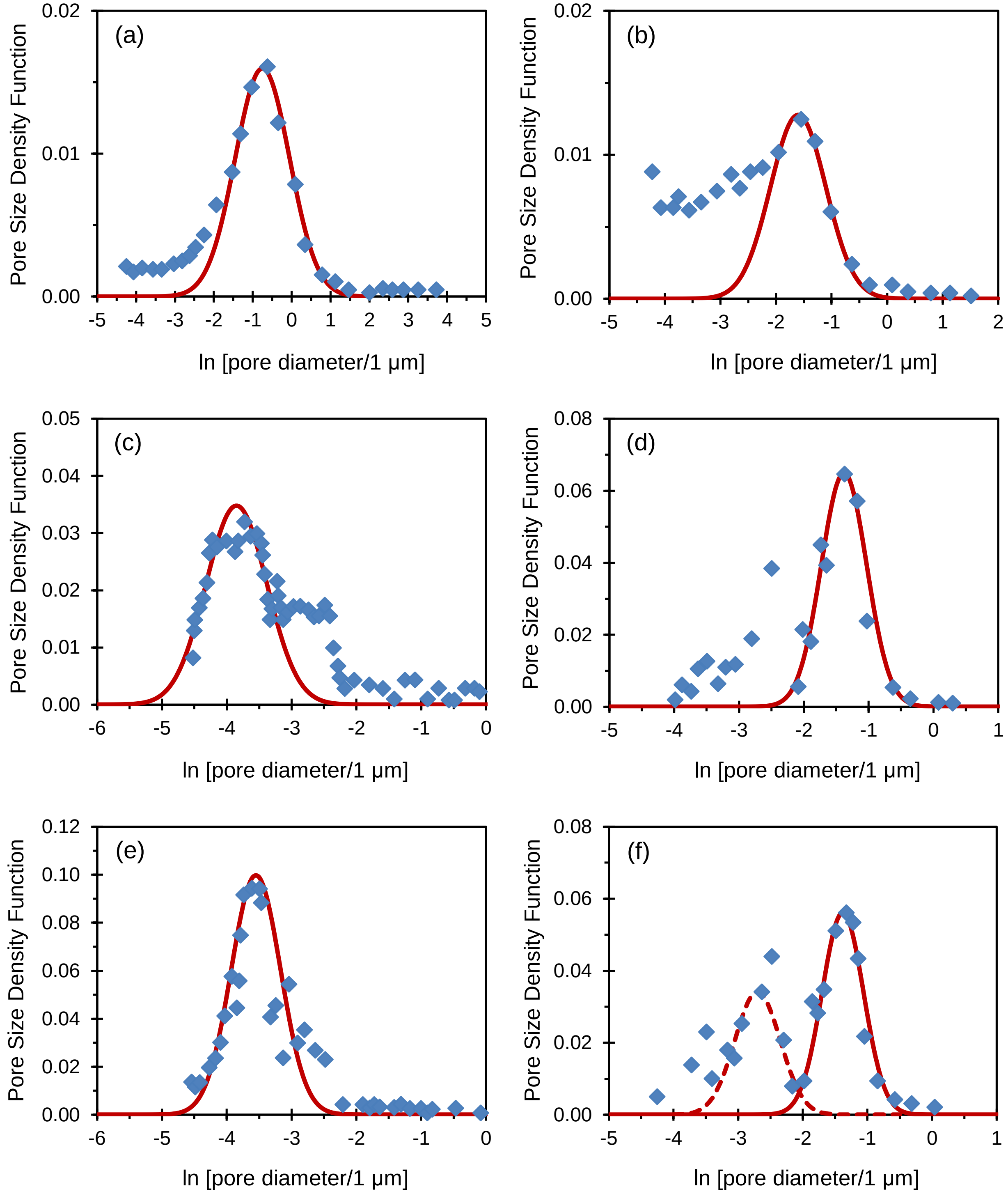

Supplementary Figure S5. Pore size distribution – Sandstones. (a) C11, (b) C29, (c) No.1, (d) No.2, (e) No.3, and (f) No.4 (Supplementary Table 2 – Refs. 22, 23).

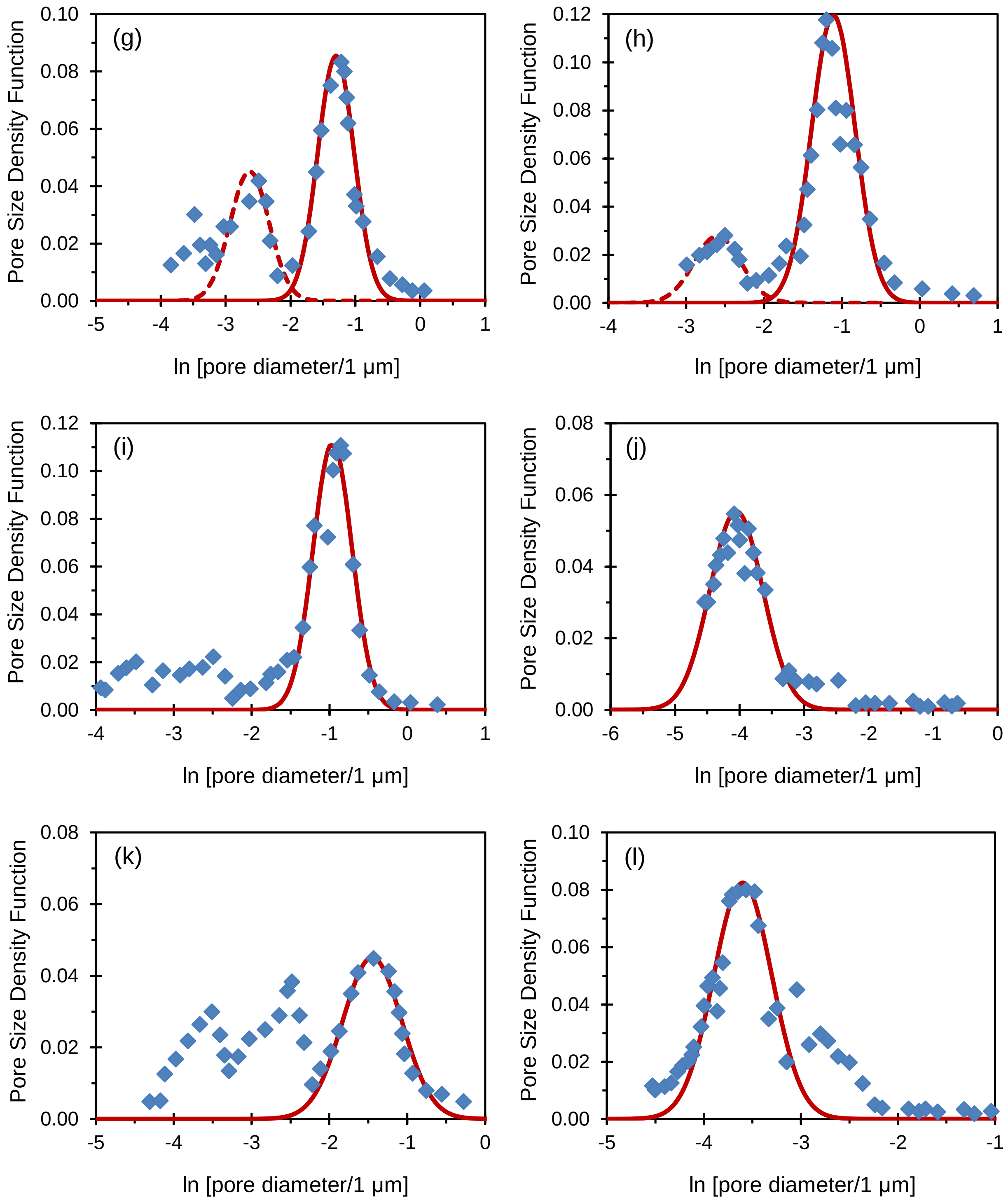

Supplementary Figure S5. Pore size distribution – Sandstones (Continued). (g) No. 5, (h) No. 6, (i) No. 7, (j) No. 8, (k) No. 9, and (l) No. 10 (Supplementary Table 2 – Ref. 23).

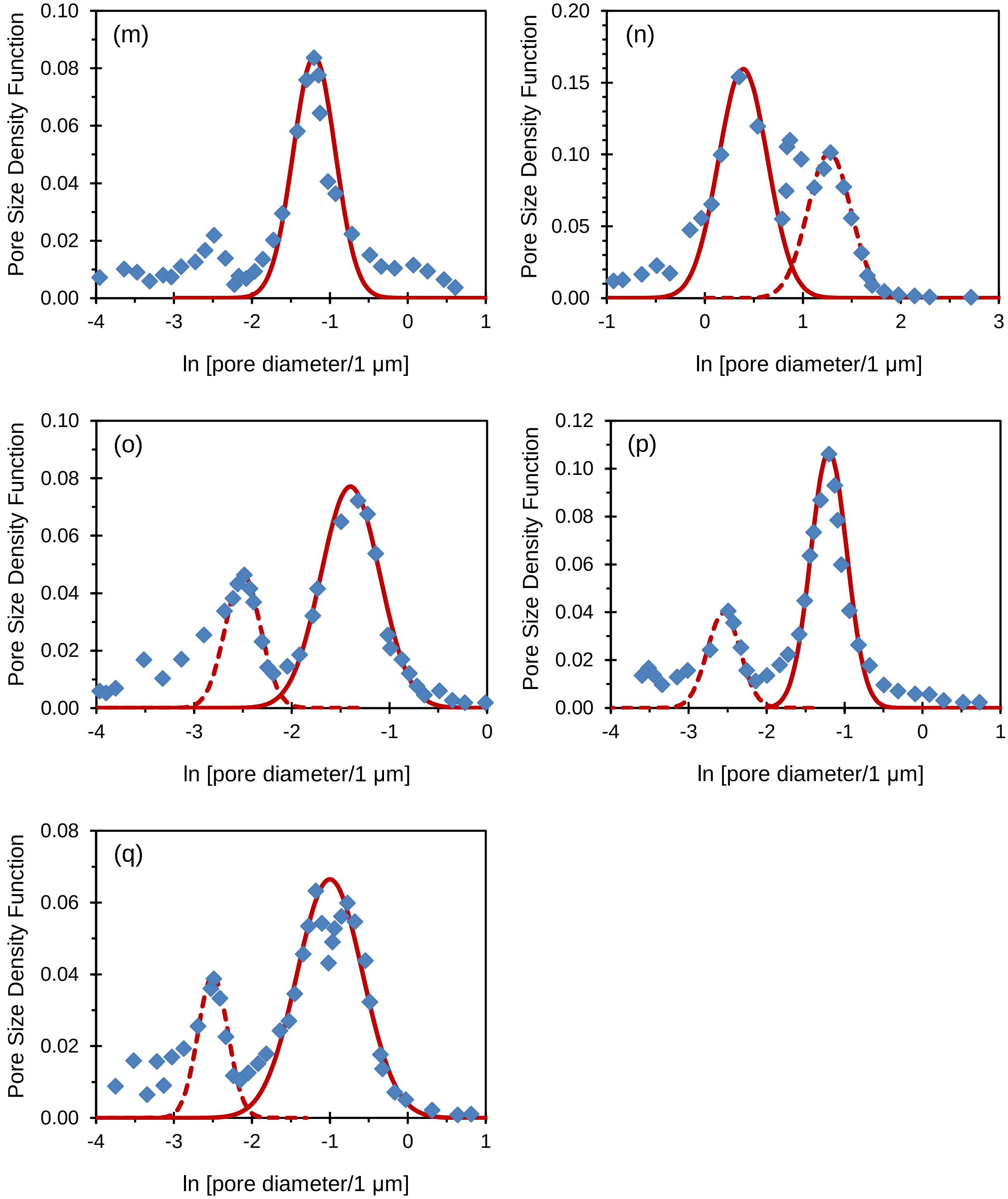

Supplementary Figure S5. Pore size distribution – Sandstones (Continued). (m) No. 11, (n) No. 12, (o) No. 13, (p) No. 14, and (q) No. 15 (Supplementary Table 2 – Ref. 23).

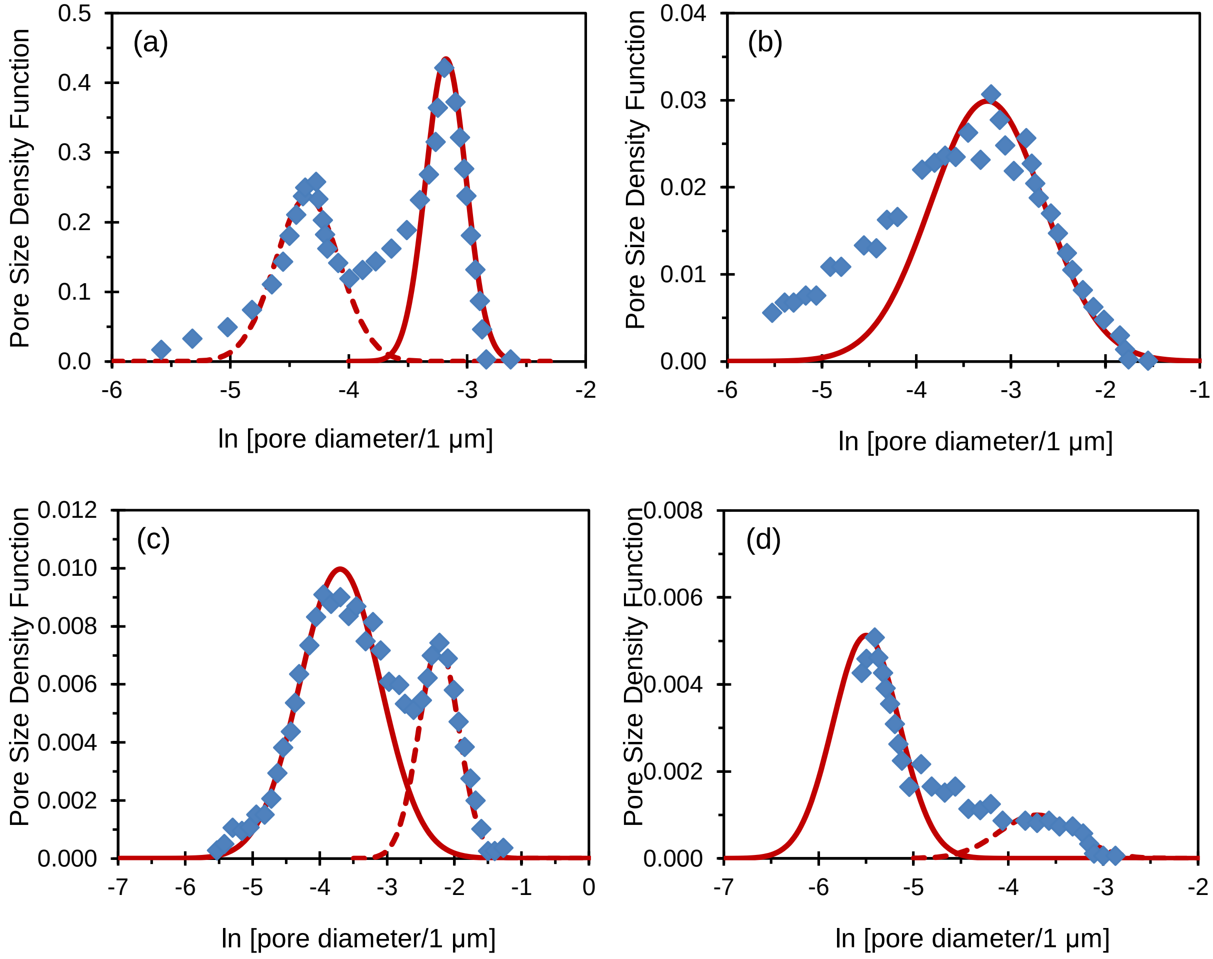

Supplementary Figure S6. Pore size distribution – Shales. (a) North Sea shale, (b) Mancos B, (c) Middle Bakken, and (d) Woodford Shale (Supplementary Table 2 – Ref. 23).

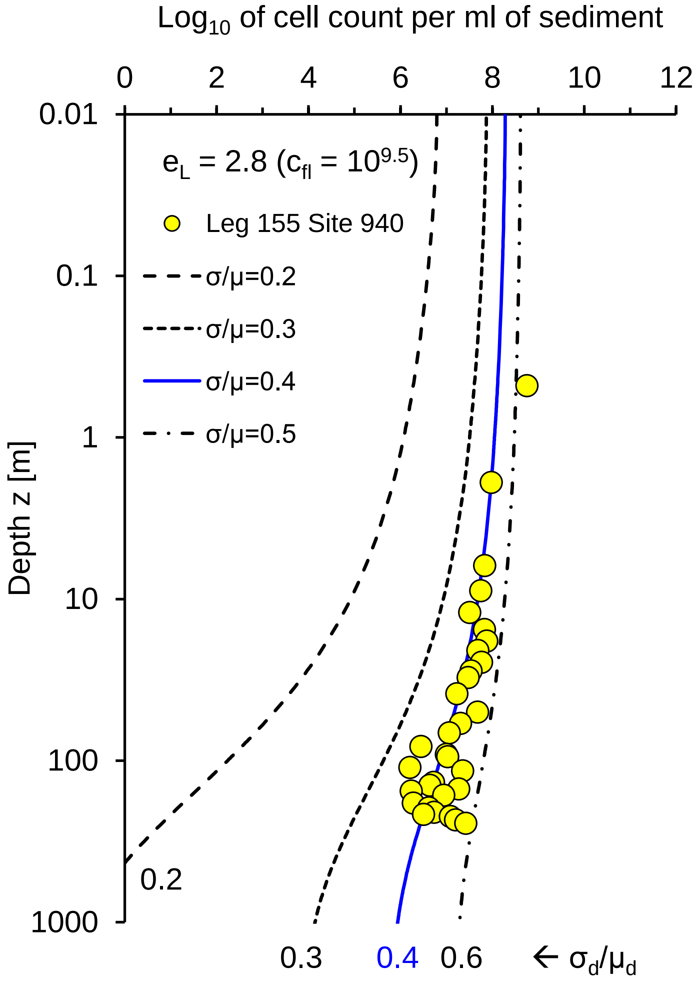

Supplementary Figure S7. Cell count data profiles versus sediment depth and prediction models - Effect of the ratio of standard deviation σ_d to mean pore size μ_d (i.e., σ_d/μ_d) on cell count prediction. Data extracted from Amazon Fen: Leg 155 - Site 940 (Ref. 45). Model parameters include the estimated cell concentration of the pore fluid $c_{fl} = 10^{9.5}$ cell counts/cm^3 and asymptotic void ratio $e_L = 2.8$ (note: all other parameters are kept constant for this analysis).

VOID RATIO AND CELL COUNT PROFILES

The 116 void ratio and cell count profiles are organized into three groups (Supplementary Table S3):

66 cases exhibit clear and consistent trends
44 cases have high variability
6 cases for incomplete data.

For the cases with high variability:

- We recognize that possible causes for variability include stratigraphic changes, sampling effects and testing protocols. For example, typical porosity measurements are based on water content, which can change due to sampling-induced remolding, water loss, gas dissolution, and even hydrate dissociation.
- When bioactivity trends are clear, we use the reported counts to infer the possible soil type. All other model parameters are fixed.
- Plots that present double trends recognize the range of possible values.

VOID RATIO AND CELL COUNT PROFILES

Clear and Consistent trends

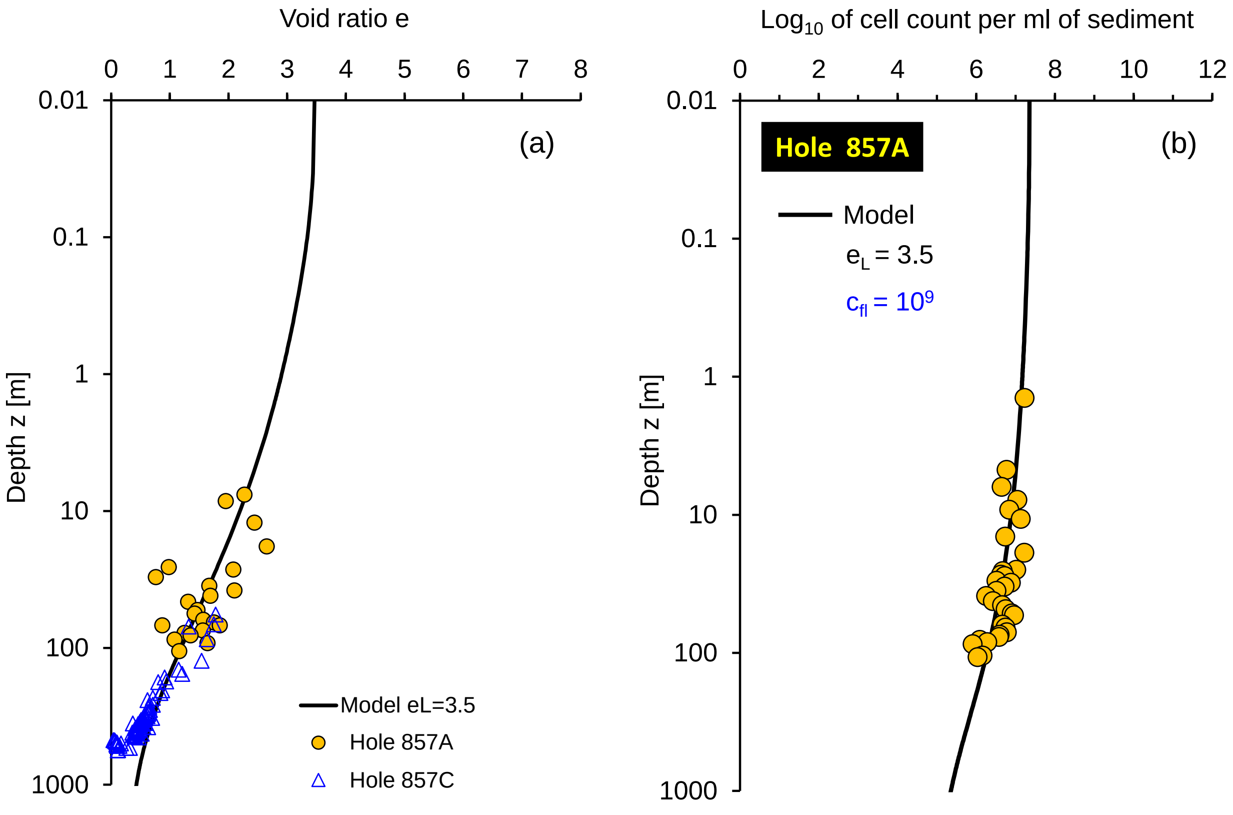

Supplementary Figure S8. Juan de Fuca Ridge: Leg 139 - Site 857A. Void ratio and cell count data profiles versus depth and prediction models. (A) Void ratio depth profile - Site 857A/C [Data extracted from (Ref. 34)] (model parameters: e_L = 3.5). (B) Cell count profile - Site 857A [data extracted from (Ref. 35)] (the estimated cell concentration of the pore fluid c_{fl} = 10^9 cell counts/cm^3).

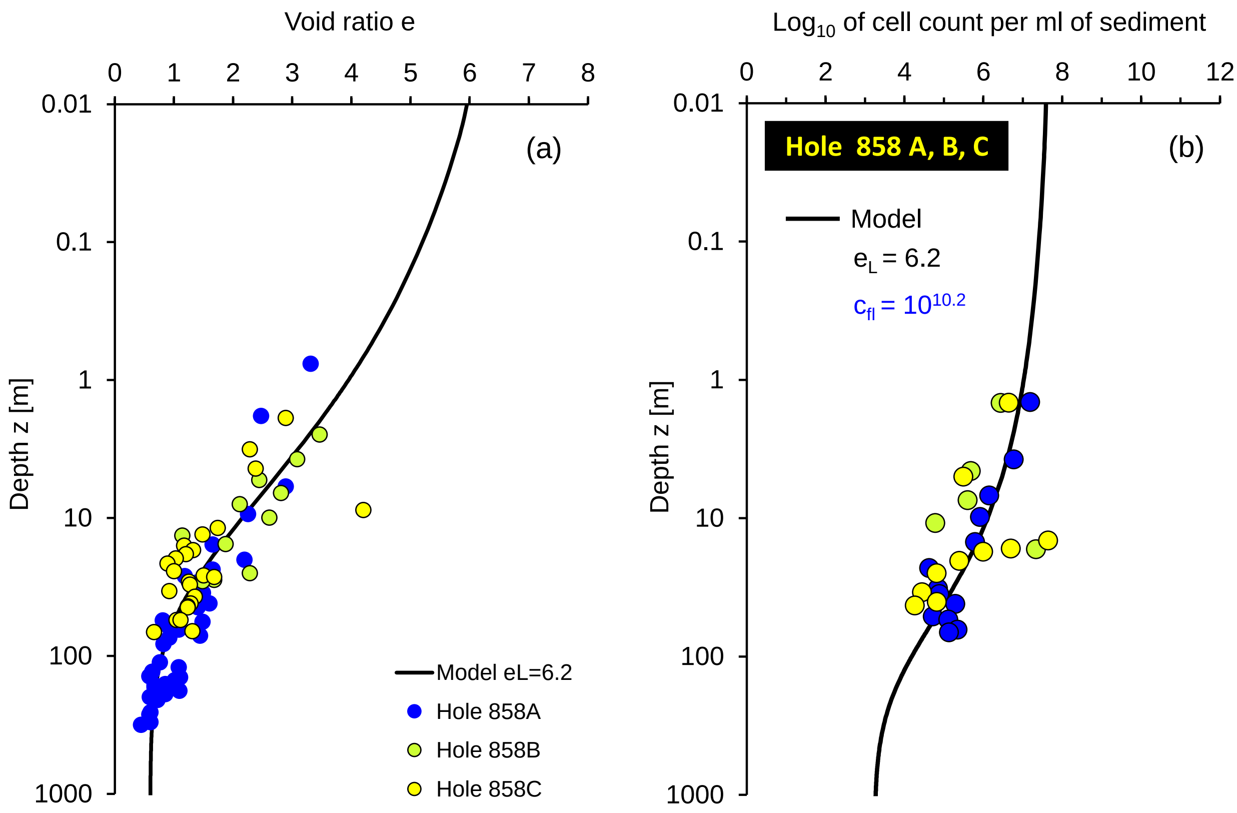

Supplementary Figure S9. Juan de Fuca Ridge: Leg 139 - Site 858A, B, C. Void ratio and cell count data profiles versus depth and prediction models. (A) Void ratio depth profile - Site 858A, B, C [Data extracted from (Ref. 36)] (model parameters: $e_L = 6.2$). (B) Cell count profile - Site 858A, B, C [data extracted from (Ref. 35)] (the estimated cell concentration of the pore fluid $c_{fl} = 10^{10.2}$ cell counts/cm^3).

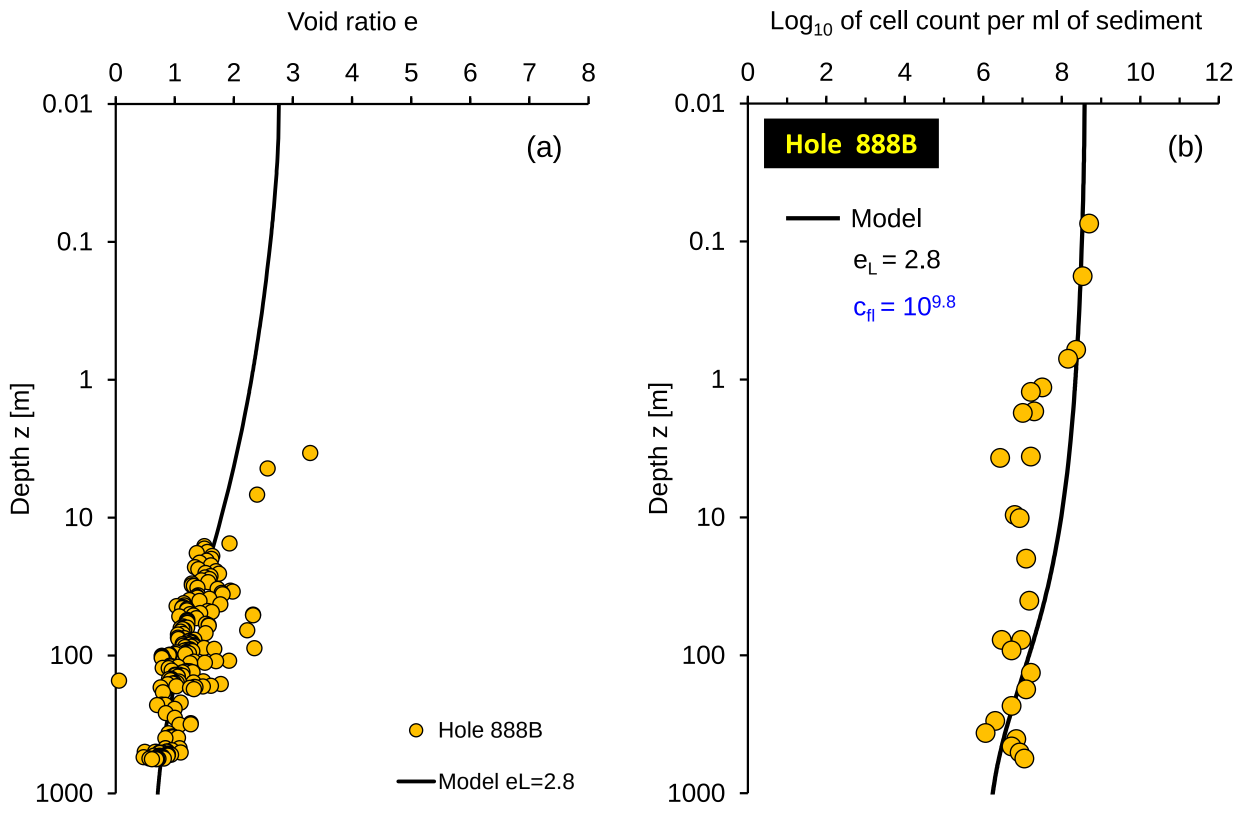

Supplementary Figure S10. Cascadia Margin: Leg 146 - Part 1: Site 888B. Void ratio and cell count data profiles versus depth and prediction models. (A) Void ratio depth profile - Site 888B [Data extracted from (Ref. 37)] (model parameters: e_L = 2.8). (B) Cell count profile - Site 888B [data extracted from (Ref. 38)] (the estimated cell concentration of the pore fluid c_{fl} = $10^{9.8}$ cell counts/cm^3).

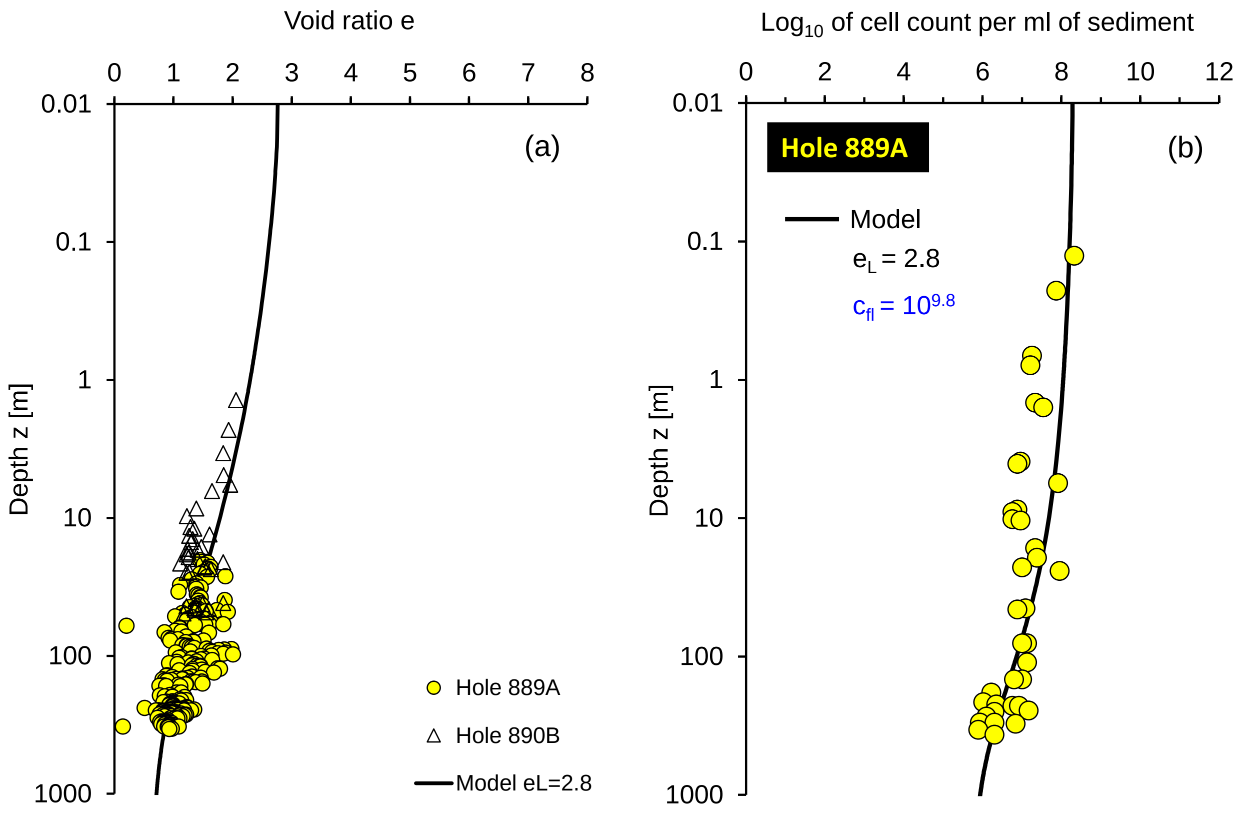

Supplementary Figure S11. Cascadia Margin: Leg 146 - Part 1: Site 889A. Void ratio and cell count data profiles versus depth and prediction models. (A) Void ratio depth profile - Site 889A [Data extracted from (Ref. 39)] (model parameters: e_L = 2.8). (B) Cell count profile - Site 889A [data extracted from (Ref. 38)] (the estimated cell concentration of the pore fluid c_{fl} = $10^{9.8}$ cell counts/cm^3).

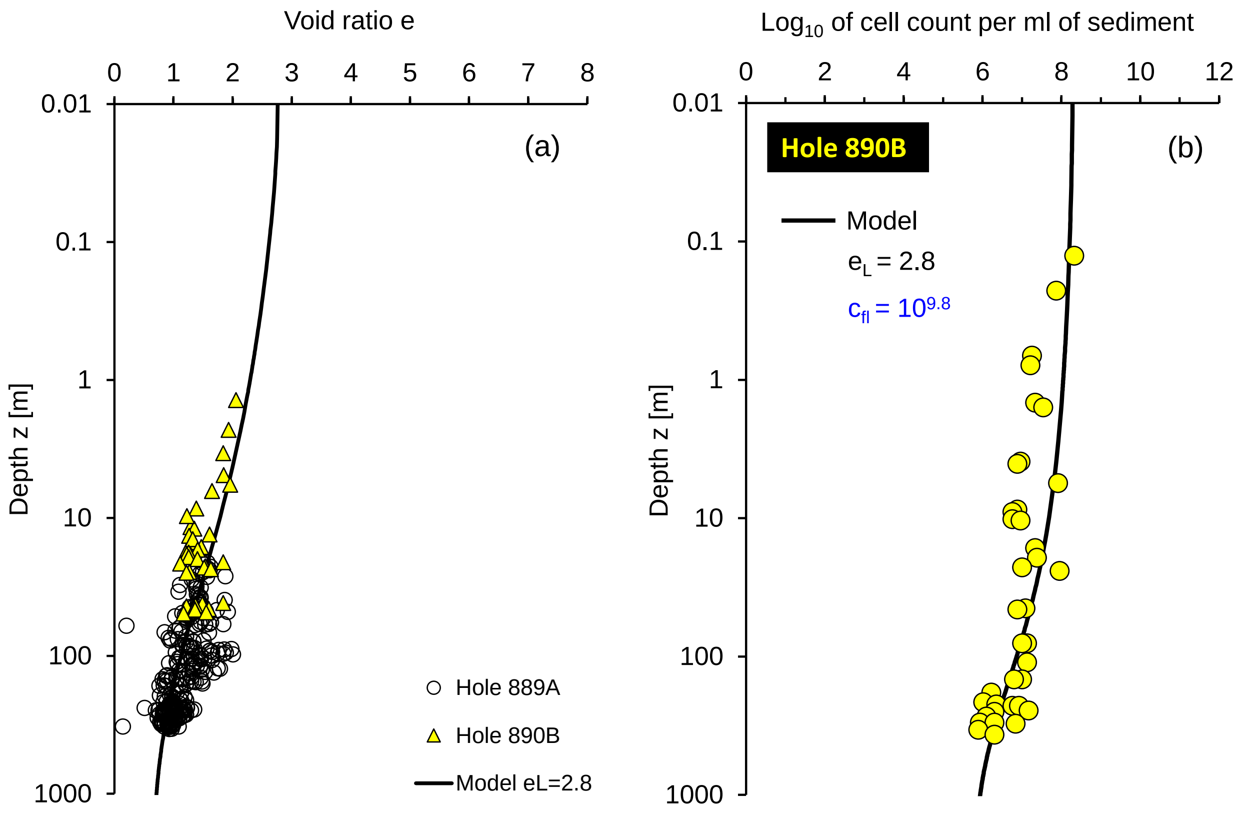

Supplementary Figure S12. Cascadia Margin: Leg 146 - Part 1: Site 890B. Void ratio and cell count data profiles versus depth and prediction models. (A) Void ratio depth profile - Site 889A/890B. [Data extracted from (Ref. 39)] (model parameters: $e_L = 2.8$). (B) Cell count profile - Site 890B [data extracted from (Ref. 38)] (the estimated cell concentration of the pore fluid $c_{fl} = 10^{9.8}$ cell counts/cm^3).

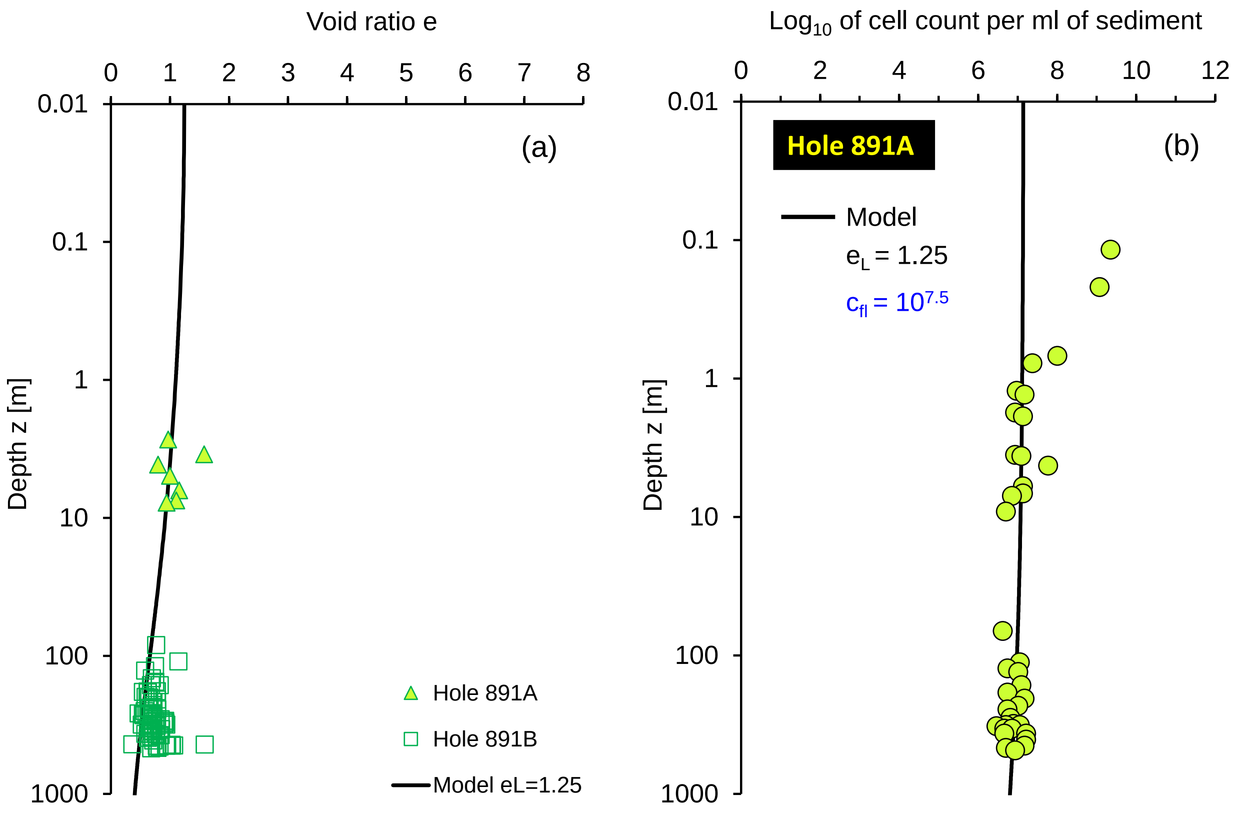

Supplementary Figure S13. Cascadia Margin: Leg 146 - Part 1: Site 891A. Void ratio and cell count data profiles versus depth and prediction models. (A) Void ratio depth profile - Site 891A. [Data extracted from (Ref. 40)] (model parameters: $e_L = 1.25$). (B) Cell count profile - Site 891A [data extracted from (Ref. 38)] (the estimated cell concentration of the pore fluid $c_{fl} = 10^{7.5}$ cell counts/cm^3).

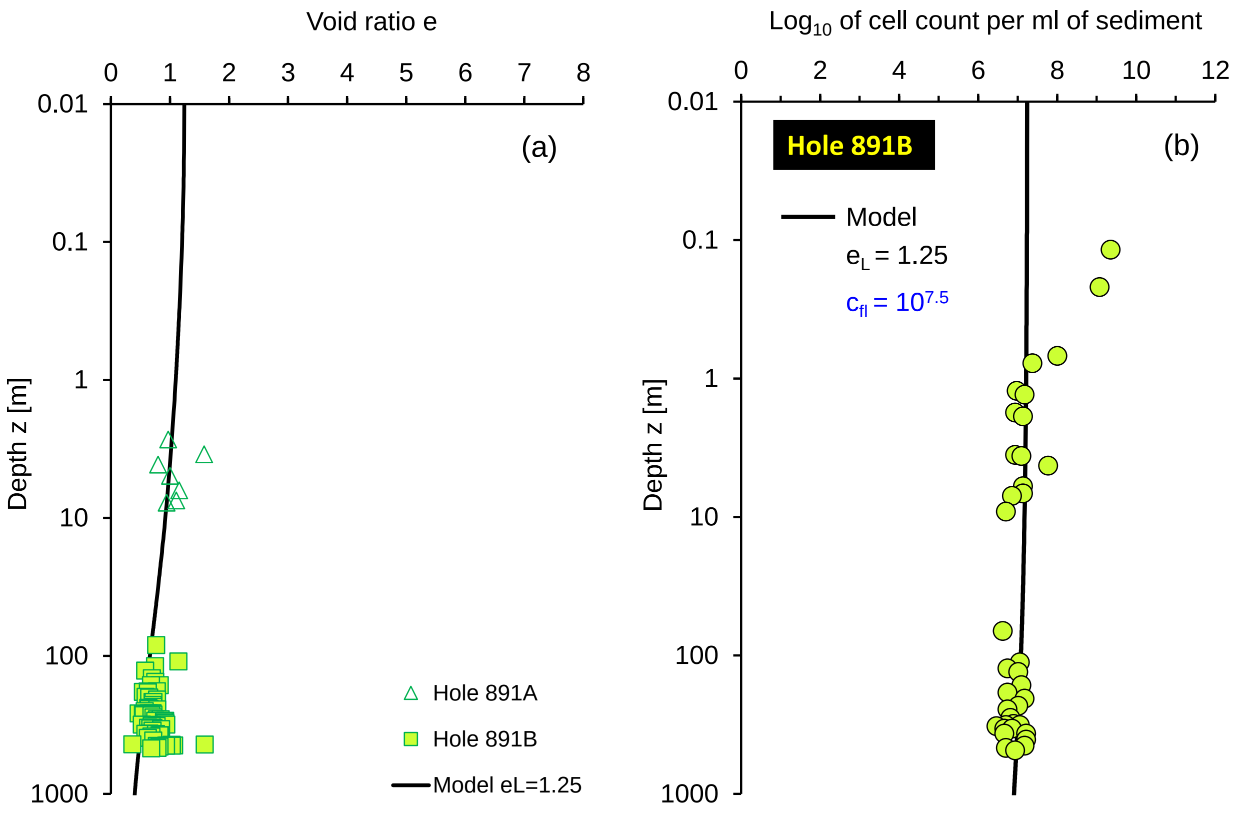

Supplementary Figure S14. Cascadia Margin: Leg 146 - Part 1: Site 891B. Void ratio and cell count data profiles versus depth and prediction models. (A) Void ratio depth profile - Site 891B. [Data extracted from (Ref. 40)] (model parameters: $e_L = 1.25$). (B) Cell count profile - Site 891B [data extracted from (Ref. 38)] (the estimated cell concentration of the pore fluid $c_{fl} = 10^{7.5}$ cell counts/cm^3).

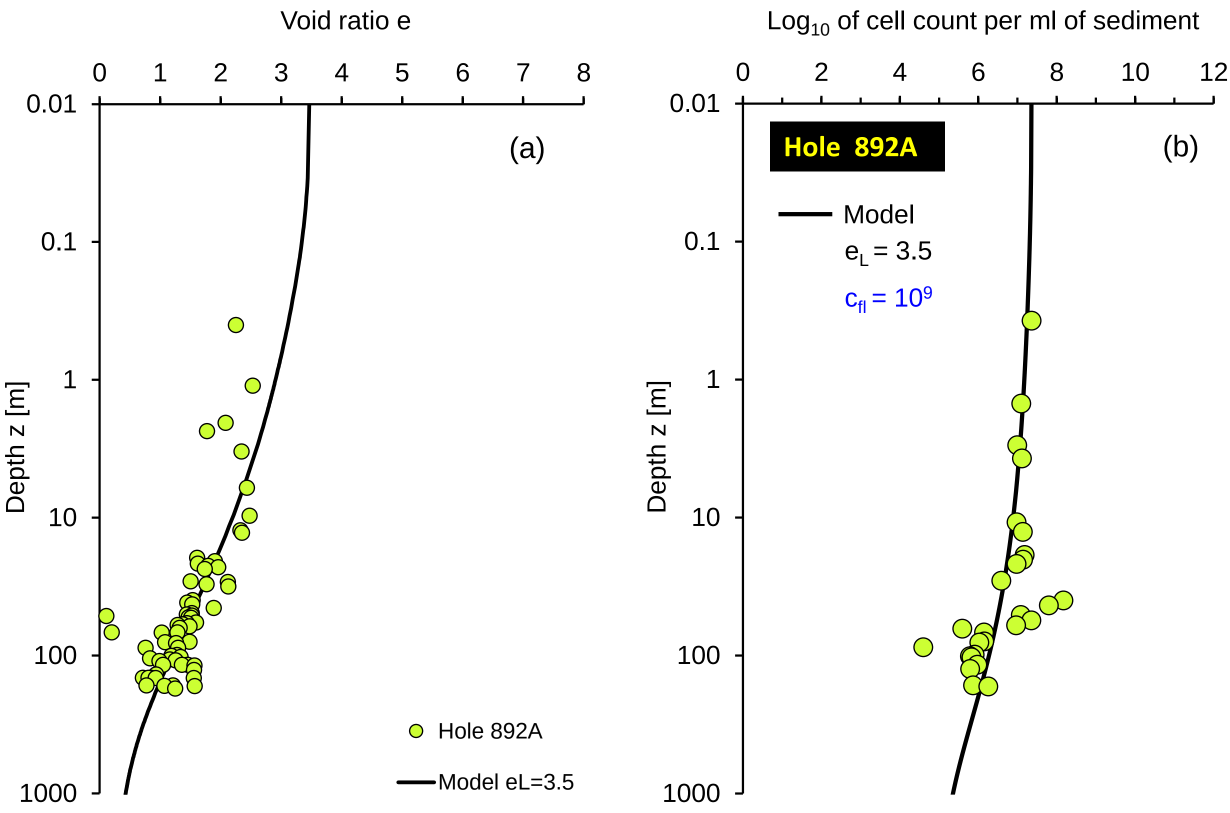

Supplementary Figure S15. Cascadia Margin: Leg 146 - Part 1: Site 892A. Void ratio and cell count data profiles versus depth and prediction models. (A) Void ratio depth profile - Site 892A [Data extracted from (Ref. 41)] (model parameters: e_L = 3.5). (B) Cell count profile - Site 892A [data extracted from (Ref. 38)] (the estimated cell concentration of the pore fluid c_{fl} = 10^9 cell counts/cm^3).

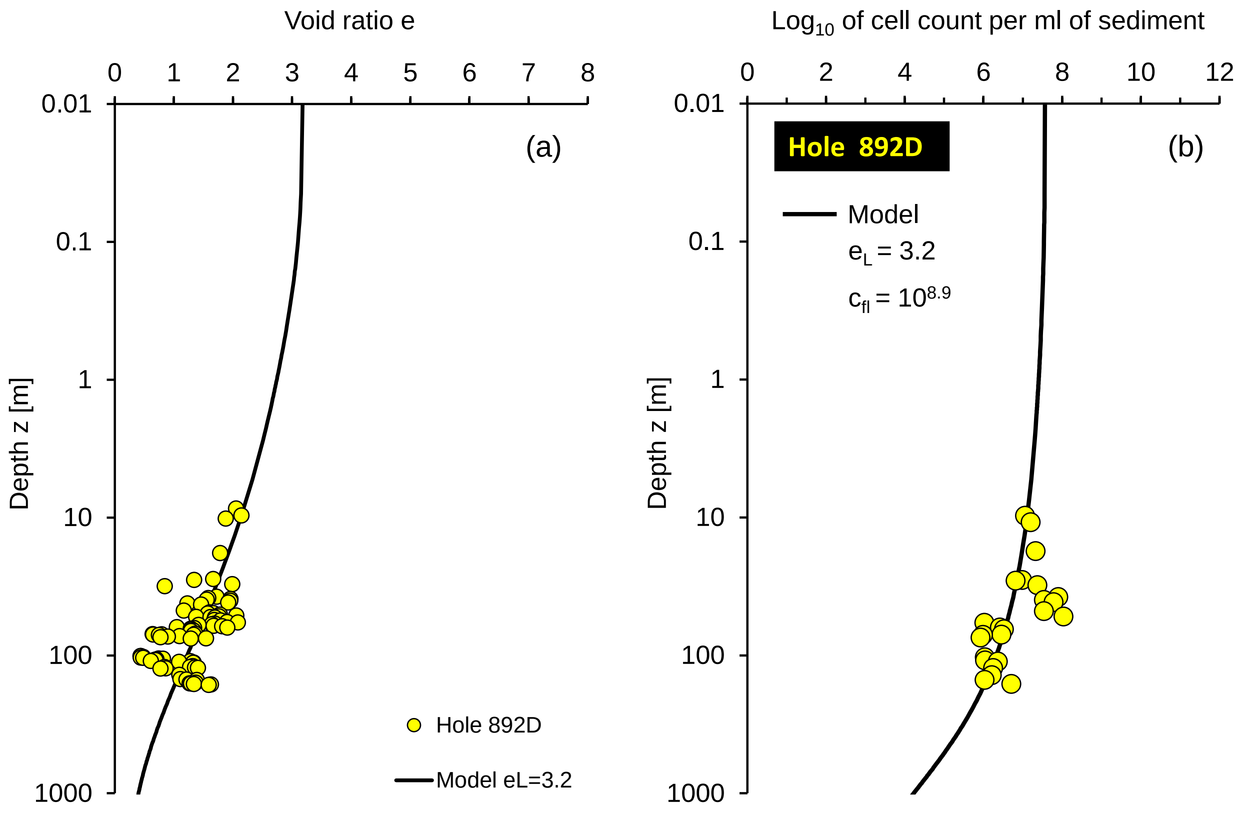

Supplementary Figure S16. Cascadia Margin: Leg 146 - Part 1: Site 892D. Void ratio and cell count data profiles versus depth and prediction models. (A) Void ratio depth profile - Site 892D [Data extracted from (Ref. 41)] (model parameters: e_L = 3.2). (B) Cell count profile - Site 892D [data extracted from (Ref. 38)] (the estimated cell concentration of the pore fluid c_{fl} = $10^{8.9}$ cell counts/cm^3).

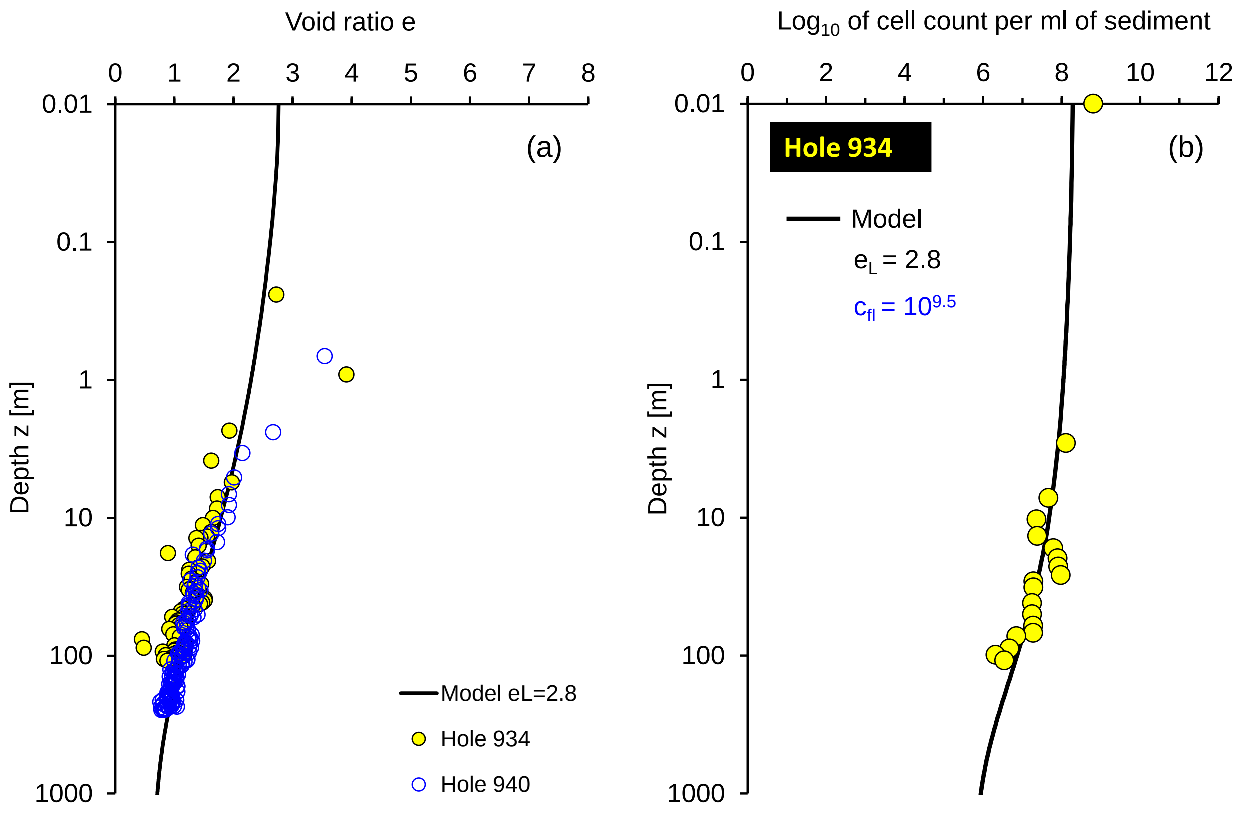

Supplementary Figure S17. Amazon Fen: Leg 155 - Site 934. Void ratio and cell count data profiles versus depth and prediction models. (A) Void ratio depth profile - Site 934 [Data extracted from (Ref. 44)] (model parameters: e_L = 2.8). (B) Cell count profile - Site 934 [data extracted from (Ref. 45)] (the estimated cell concentration of the pore fluid c_{fl} = $10^{9.5}$ cell counts/cm^3).

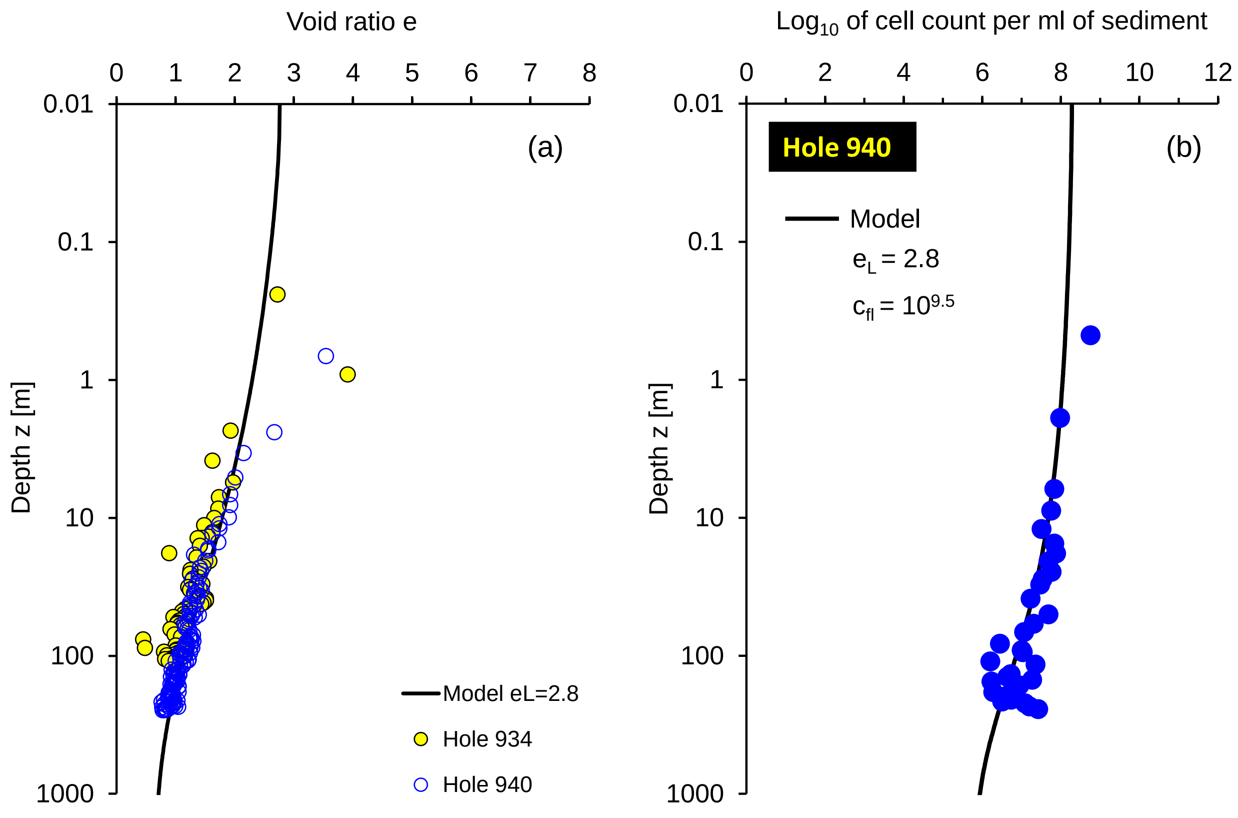

Supplementary Figure S18. Amazon Fen: Leg 155 - Site 940. Void ratio and cell count data profiles versus depth and prediction models. (A) Void ratio depth profile - Site 940 [Data extracted from (Ref. 46)] (model parameters: $e_L = 2.8$). (B) Cell count profile - Site 940 [data extracted from (Ref. 45)] (the estimated cell concentration of the pore fluid $c_{fl} = 10^{9.5}$ cell counts/cm^3).

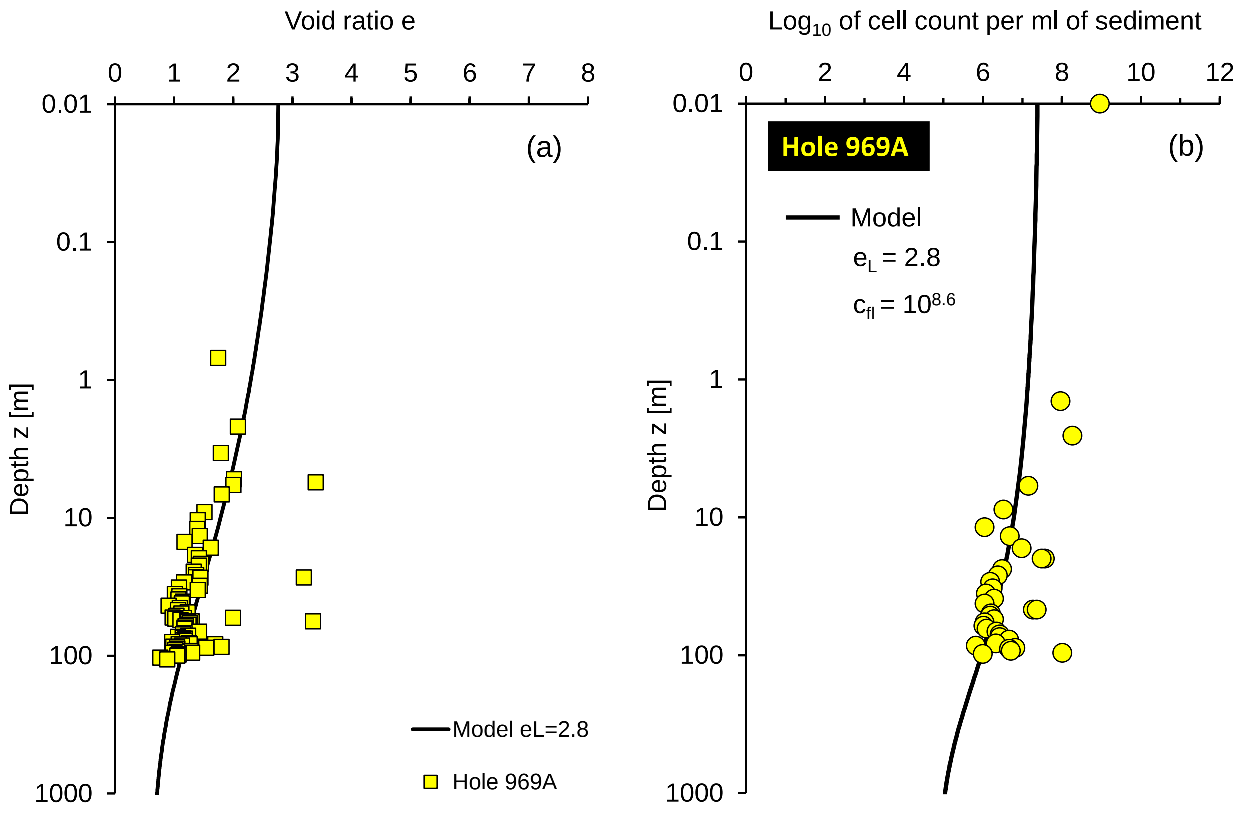

Supplementary Figure S19. Eastern Mediterranean: Leg 160 - Site 969A. Void ratio and cell count data profiles versus depth and prediction models. (A) Void ratio depth profile - Site 969A [Data extracted from (Ref. 47)] (model parameters: e_L = 2.8). (B) Cell count profile - Site 969A [data extracted from (Ref. 48)] (the estimated cell concentration of the pore fluid c_{fl} = $10^{8.6}$ cell counts/cm^3).

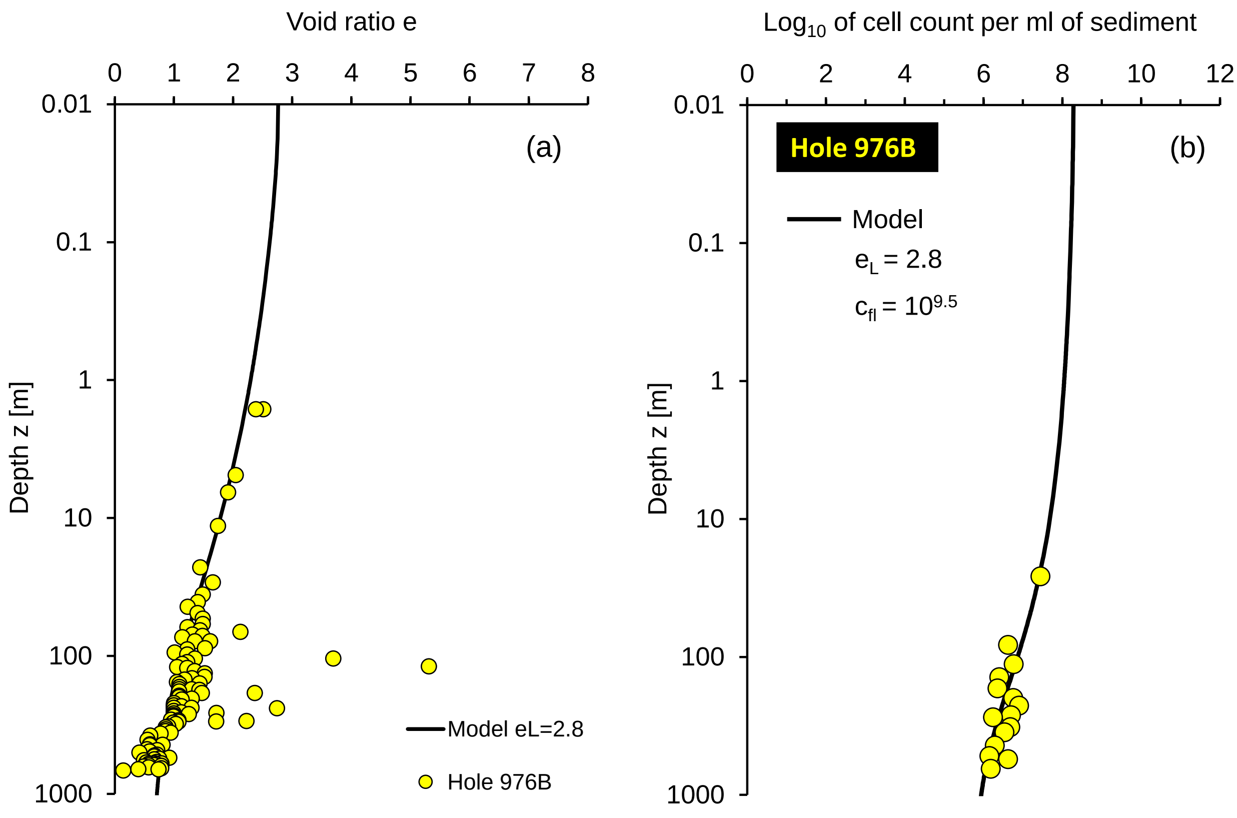

Supplementary Figure S20. Alboran Sea: Leg 161 - Site 976B. Void ratio and cell count data profiles versus depth and prediction models. (A) Void ratio depth profile - 976B [Data extracted from (Ref. 49)] (model parameters: e_L = 2.8). (B) Cell count profile - Site 976B [data extracted from (Ref. 50)] (the estimated cell concentration of the pore fluid $c_{fl} = 10^{9.5}$ cell counts/cm^3).

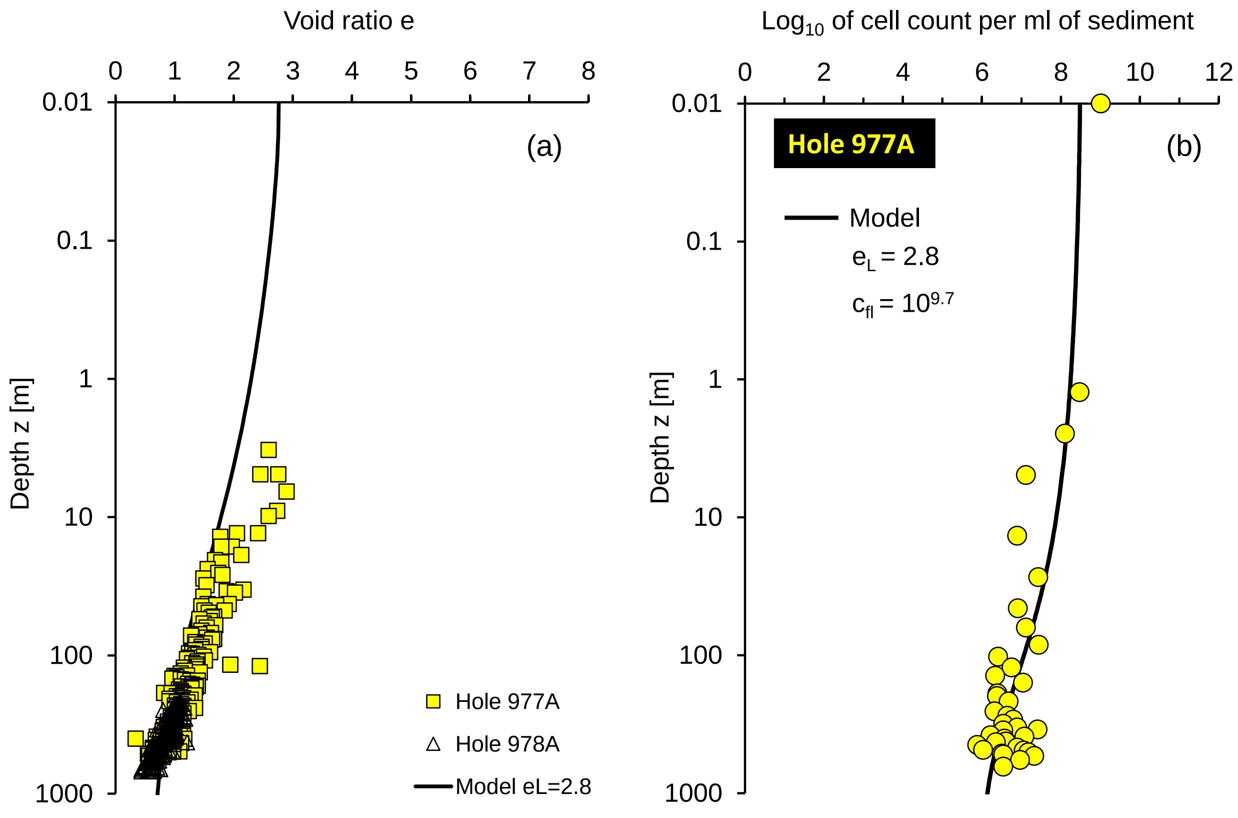

Supplementary Figure S21. Alboran Sea: Leg 161 - Site 977A. Void ratio and cell count data profiles versus depth and prediction models. (A) Void ratio depth profile - 977A [Data extracted from (Ref. 51)] (model parameters: e_L = 2.8). (B) Cell count profile - Site 977A [data extracted from (Ref. 50)] (the estimated cell concentration of the pore fluid $c_{fl} = 10^{9.7}$ cell counts/cm^3).

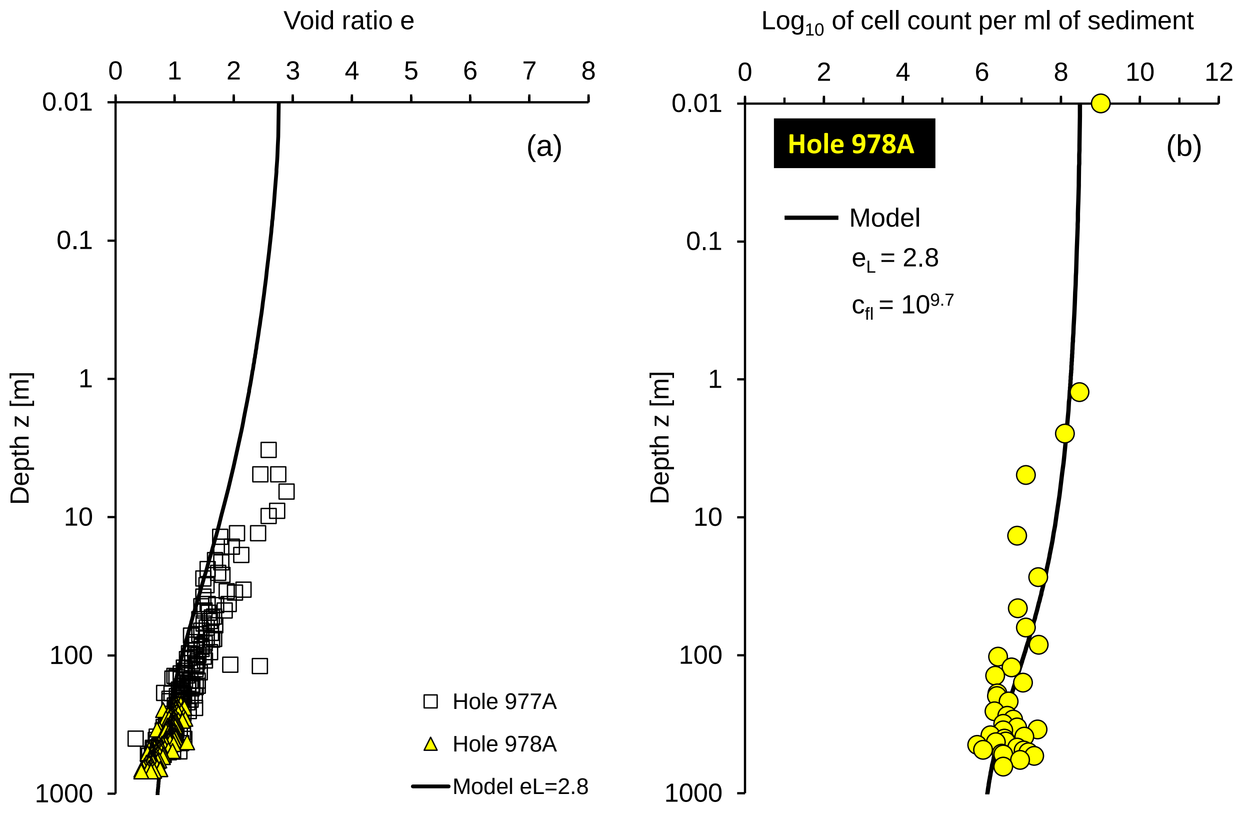

Supplementary Figure S22. Alboran Sea: Leg 161 - Site 978A. Void ratio and cell count data profiles versus depth and prediction models. (A) Void ratio depth profile - 978A [Data extracted from (Ref. 52)] (model parameters: e_L = 2.8). (B) Cell count profile - Site 978A [data extracted from (Ref. 50)] (the estimated cell concentration of the pore fluid c_{fl} = $10^{9.7}$ cell counts/cm^3).

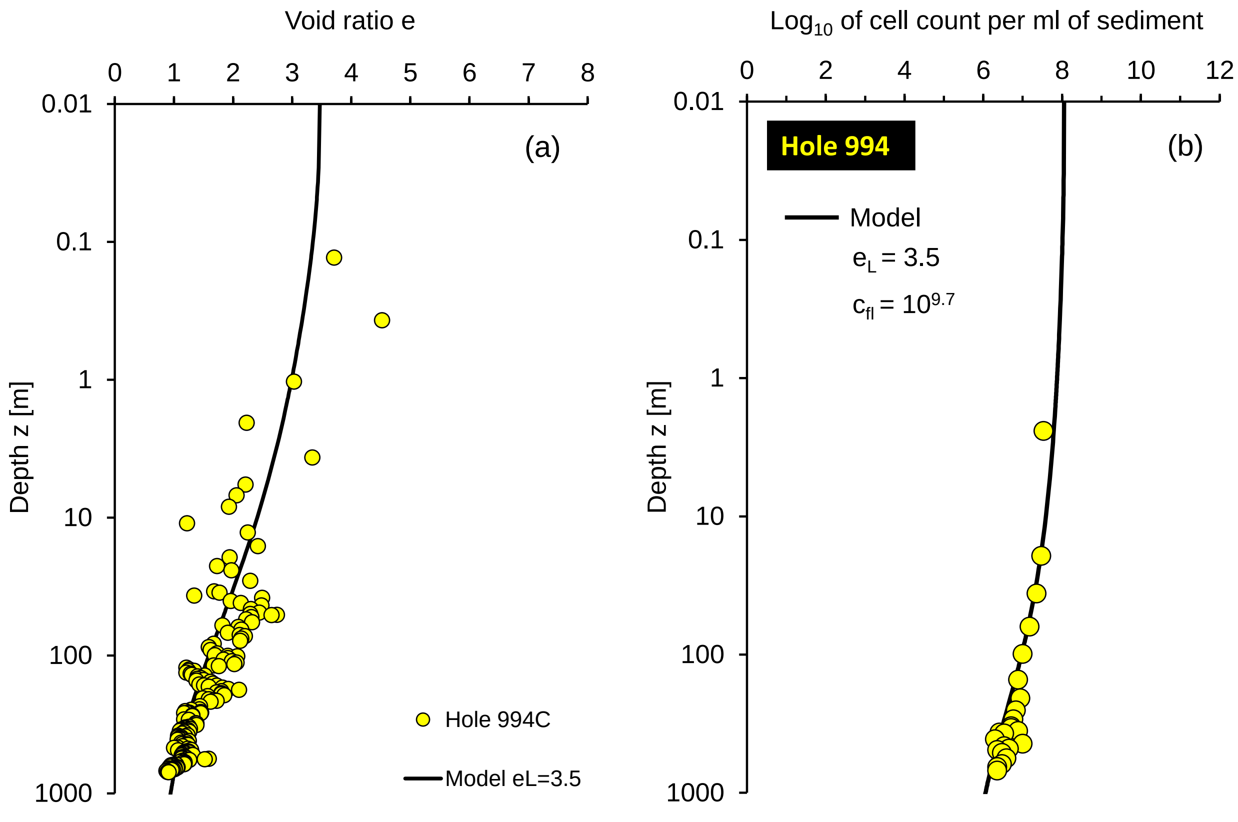

Supplementary Figure S23. Blake Ridge: Leg 164 - Site 994. Void ratio and cell count data profiles versus depth and prediction models. (A) Void ratio depth profile - Site 994C [Data extracted from (Ref. 53)] (model parameters: e_L = 3.5). (B) Cell count profile - Site 994 [data extracted from (Ref. 54)] (the estimated cell concentration of the pore fluid c_{fl} = $10^{9.7}$ cell counts/cm^3).

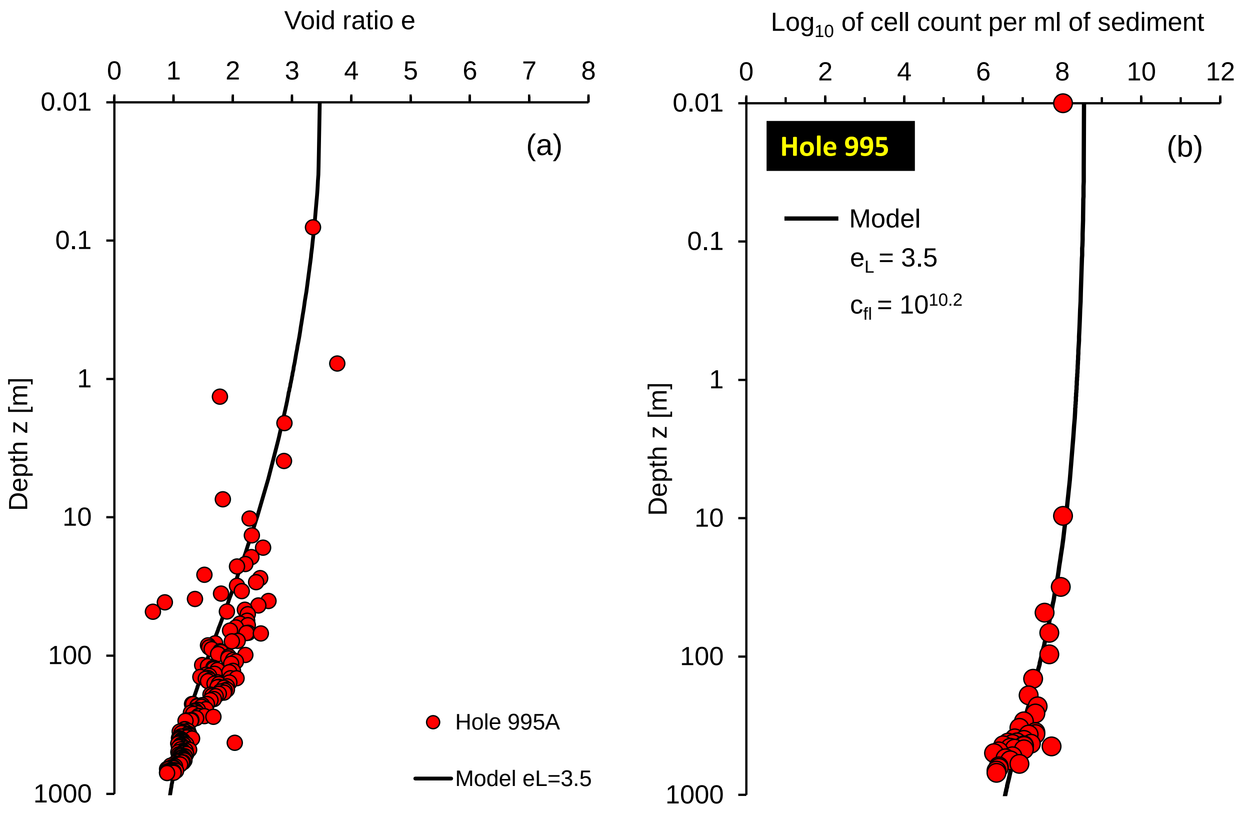

Supplementary Figure S24. Blake Ridge: Leg 164 - Site 995. Void ratio and cell count data profiles versus depth and prediction models. (A) Void ratio depth profile - Site 995A [Data extracted from (Ref. 55)] (model parameters: e_L = 3.5). (B) Cell count profile - Site 995 [data extracted from (Ref. 54)] (the estimated cell concentration of the pore fluid c_{fl} = $10^{10.2}$ cell counts/cm^3).

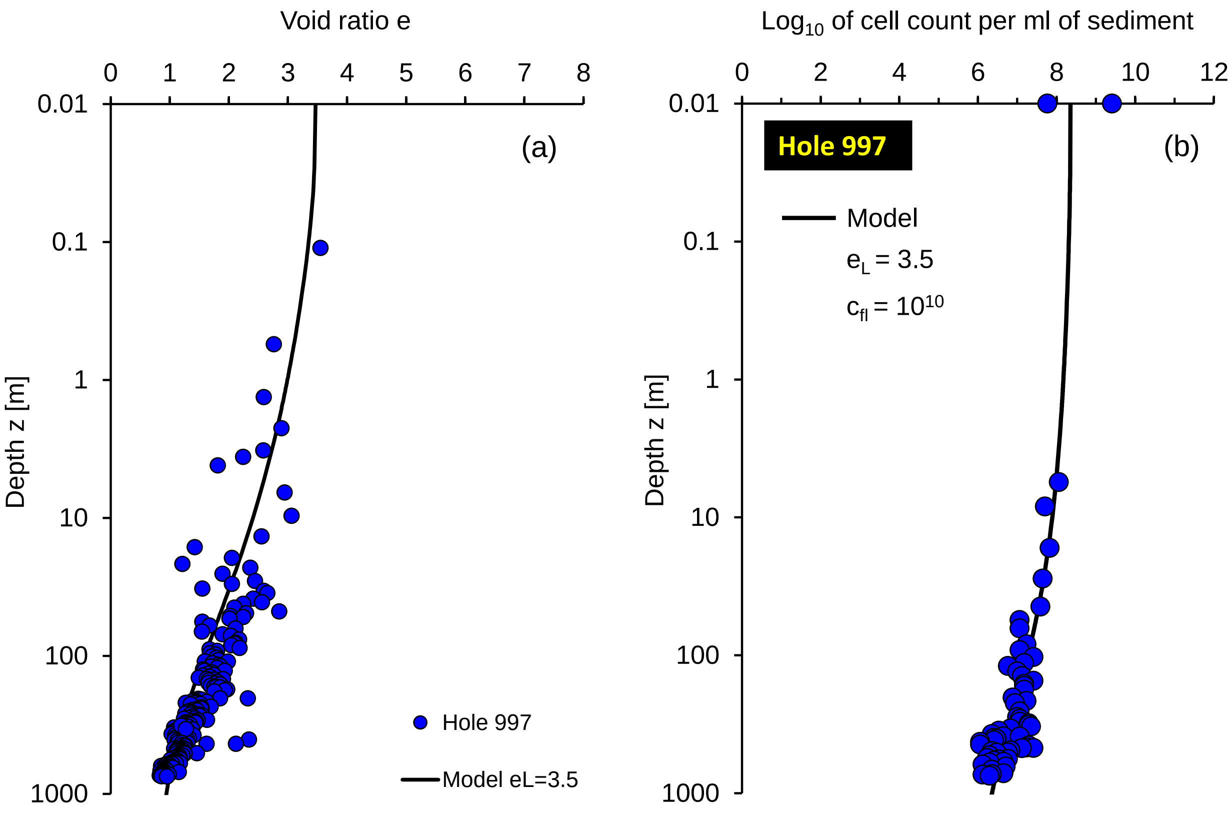

Supplementary Figure S25. Blake Ridge: Leg 164 - Site 997. Void ratio and cell count data profiles versus depth and prediction models. (A) Void ratio depth profile - Site 997 [Data extracted from (Ref. 56)] (model parameters: e_L = 3.5). (B) Cell count profile - Site 997 [data extracted from (Ref. 54)] (the estimated cell concentration of the pore fluid c_{fl} = 10^{10} cell counts/cm^3).

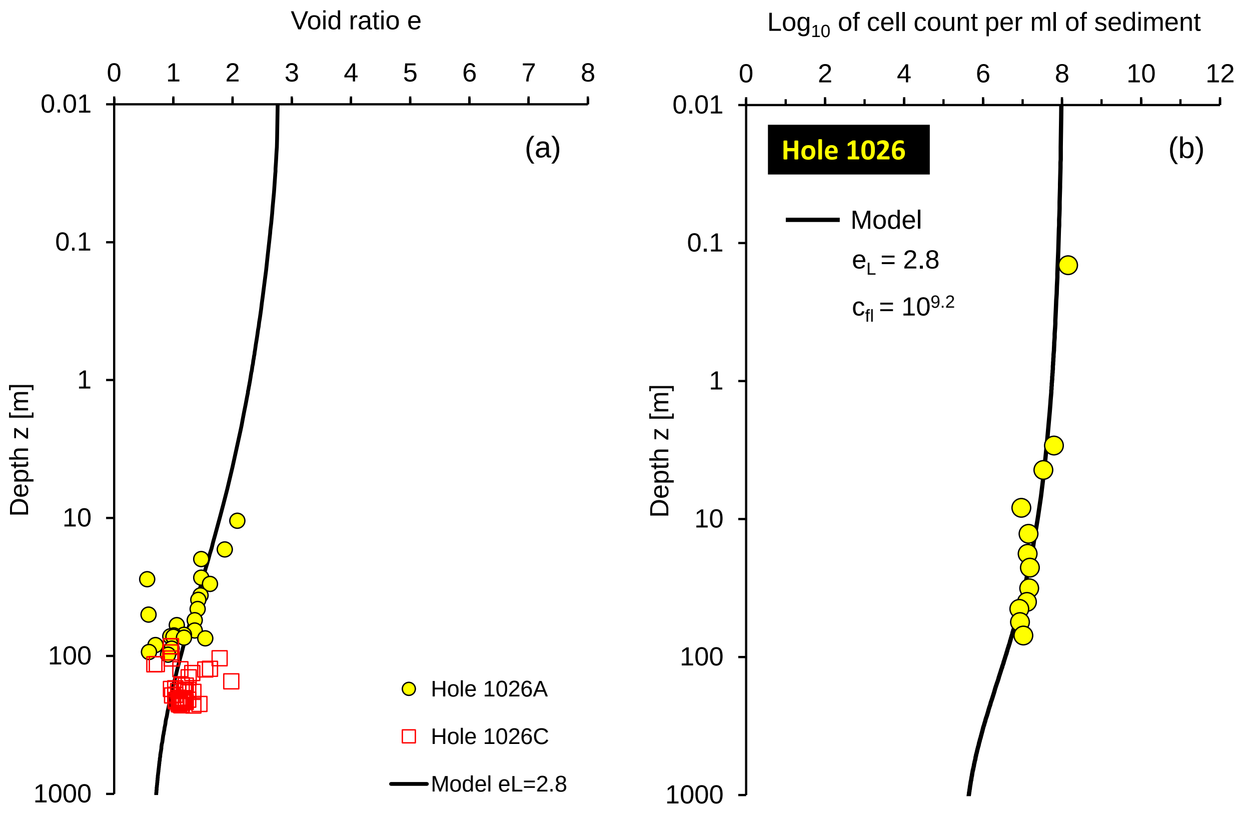

Supplementary Figure S26. Juan de Fuca Ridge: Leg 168 - Site 1026. Void ratio and cell count data profiles versus depth and prediction models. (A) Void ratio depth profile - Site 1026A/C [Data extracted from (Ref. 57)] (model parameters: e_L = 2.8). (B) Cell count profile - Site 1026 [data extracted from (Ref. 58)] (the estimated cell concentration of the pore fluid c_{fl} = $10^{9.2}$ cell counts/cm^3).

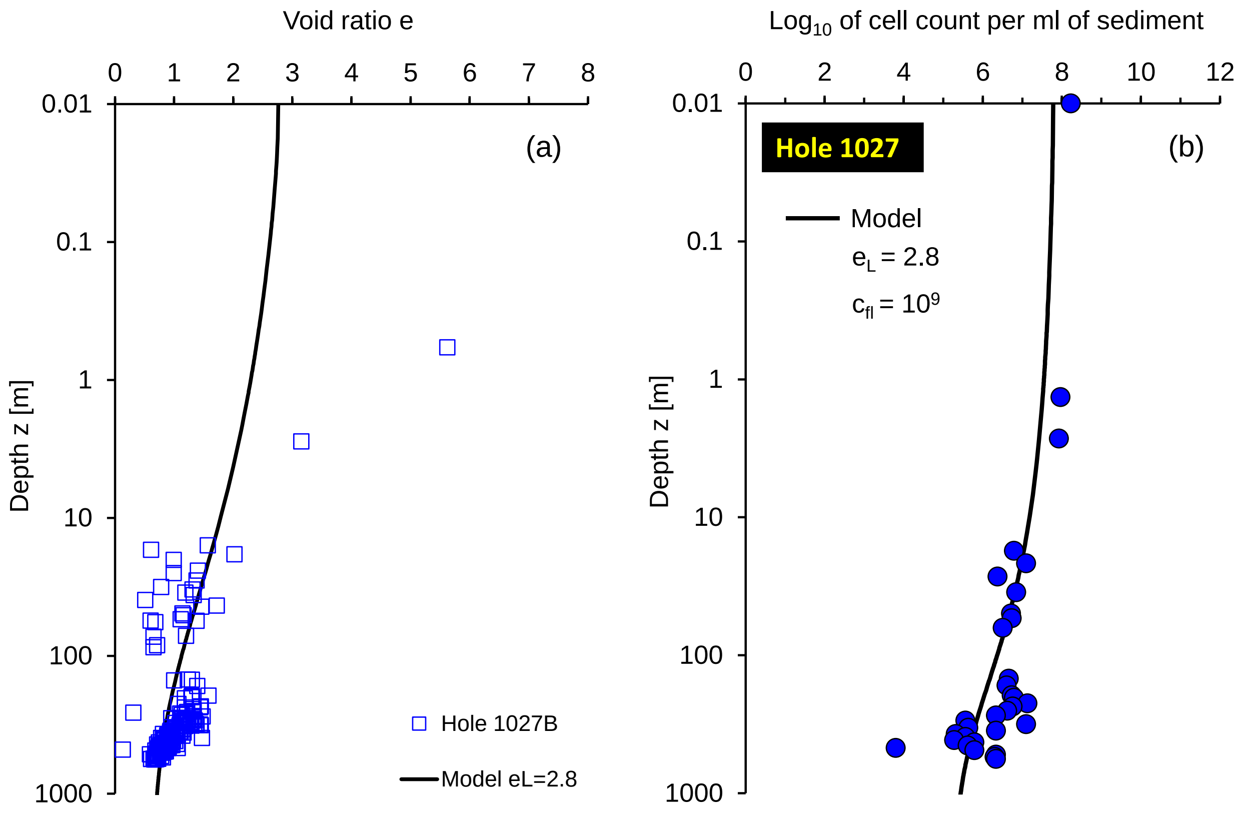

Supplementary Figure S27. Juan de Fuca Ridge: Leg 168 - Site 1027. Void ratio and cell count data profiles versus depth and prediction models. (A) Void ratio depth profile - Site 1027B [Data extracted from (Ref. 57)] (model parameters: e_L = 2.8). (B) Cell count profile - Site 1027 [data extracted from (Ref. 58)] (the estimated cell concentration of the pore fluid c_{fl} = 10^9 cell counts/cm^3).

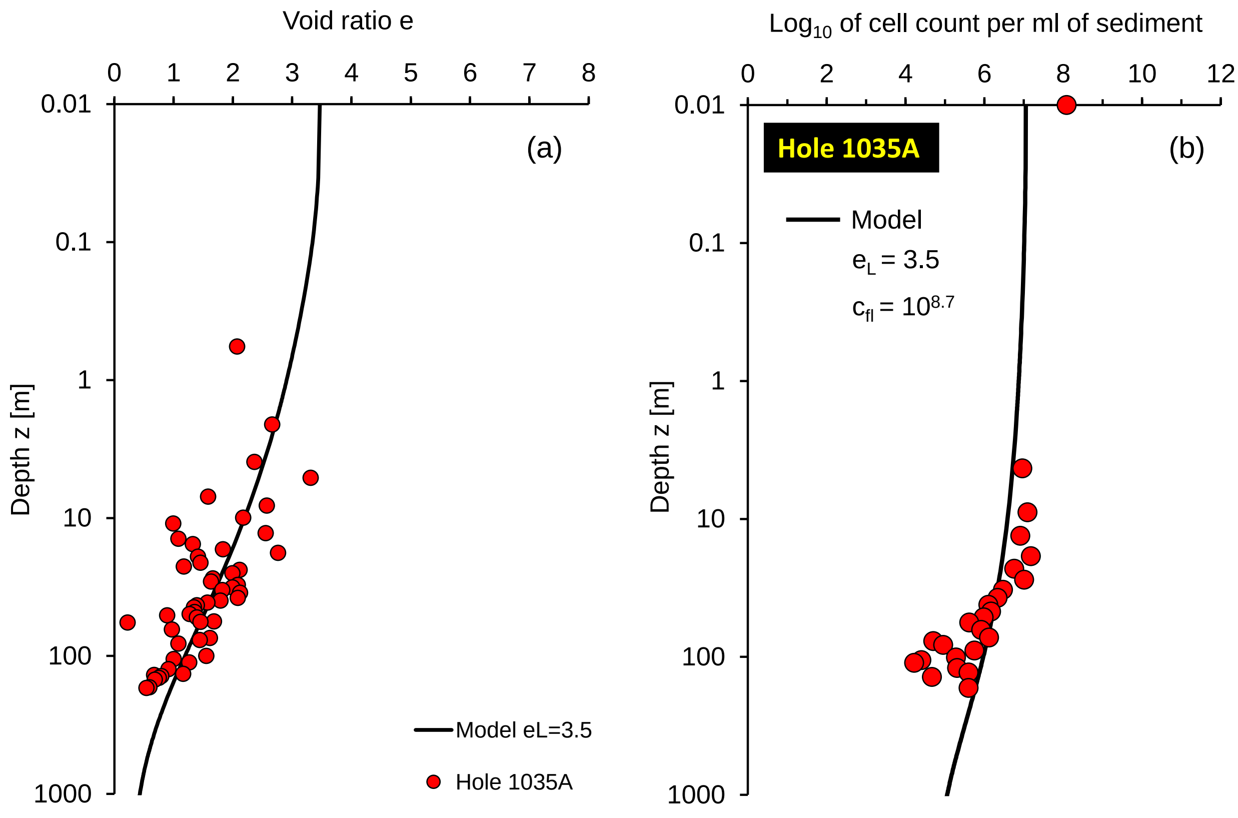

Supplementary Figure S28. Northeast Pacific: Leg 169 - Site 1035A. Void ratio and cell count data profiles versus depth and prediction models. (A) Void ratio depth profile - Site 1035A [Data extracted from (Ref. 59)] (model parameters: e_L = 3.5). (B) Cell count profile - Site 1035A [data extracted from (Ref. 60)] (the estimated cell concentration of the pore fluid c_{fl} = $10^{8.7}$ cell counts/cm^3).

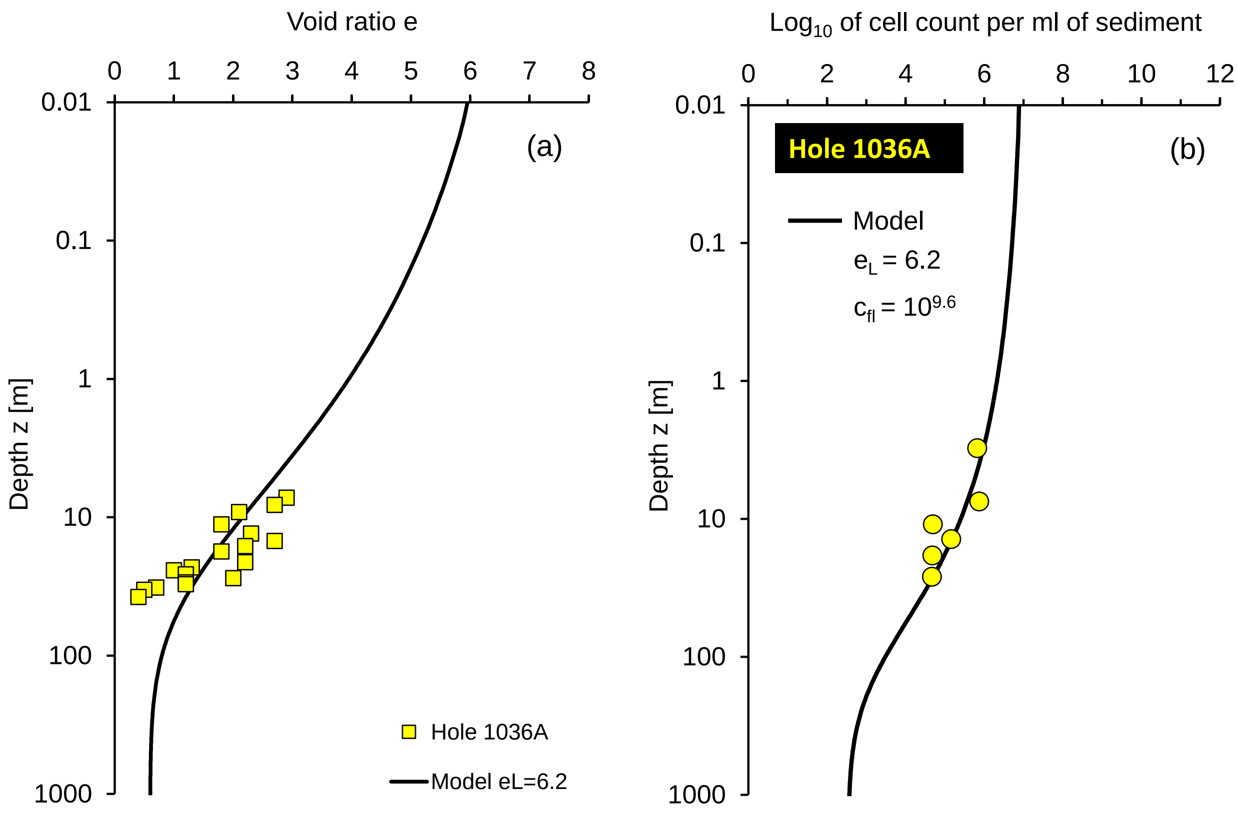

Supplementary Figure S29. Northeast Pacific: Leg 169 - Site 1036A. Void ratio and cell count data profiles versus depth and prediction models. (A) Void ratio depth profile - Site 1036A [Data extracted from (Ref. 61)] (model parameters: e_L = 6.2). (B) Cell count profile - Site 1036A [data extracted from (Ref. 60)] (the estimated cell concentration of the pore fluid c_{fl} = $10^{9.6}$ cell counts/cm^3).

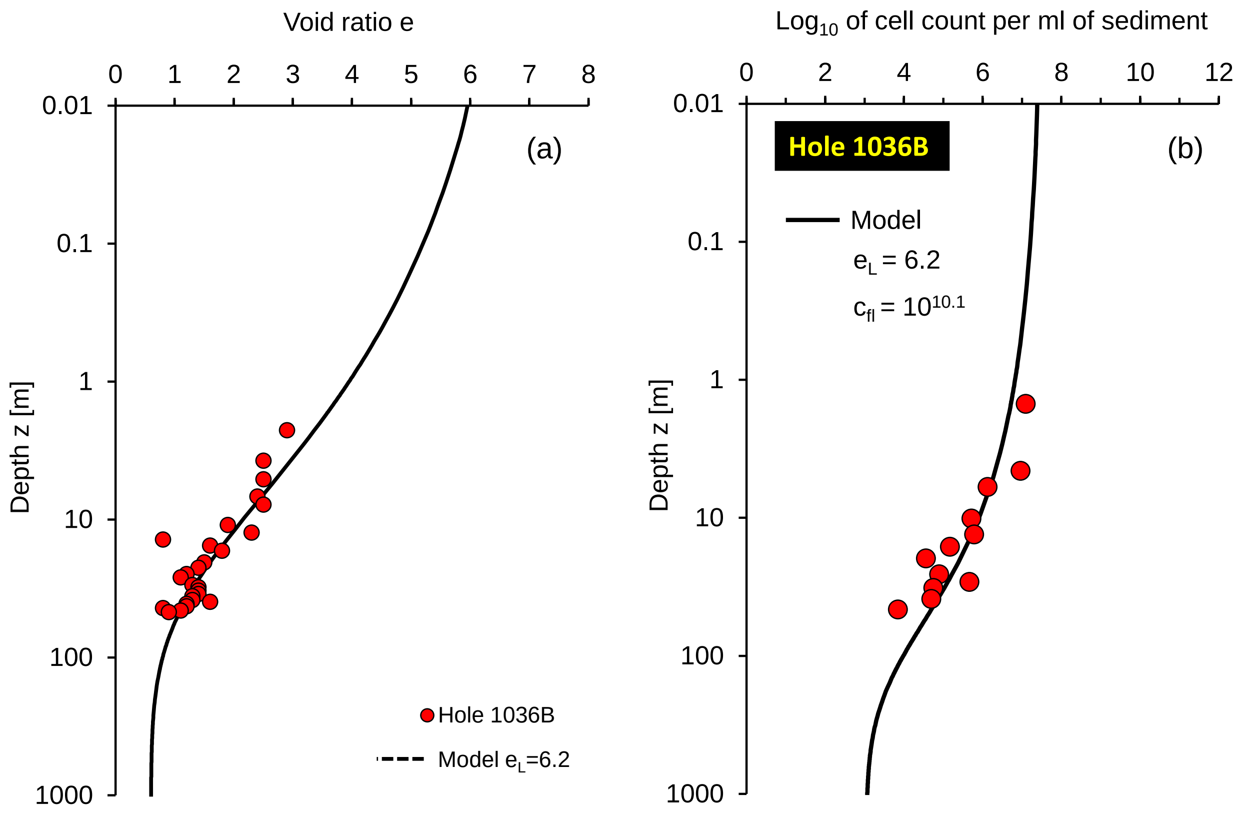

Supplementary Figure S30. Northeast Pacific: Leg 169 - Site 1036B. Void ratio and cell count data profiles versus depth and prediction models. (A) Void ratio depth profile - Site 1036B [Data extracted from (Ref. 61)] (model parameters: $e_L = 6.2$). (B) Cell count profile - Site 1036B [data extracted from (Ref. 60)] (the estimated cell concentration of the pore fluid $c_{fl} = 10^{10.1}$ cell counts/cm^3).

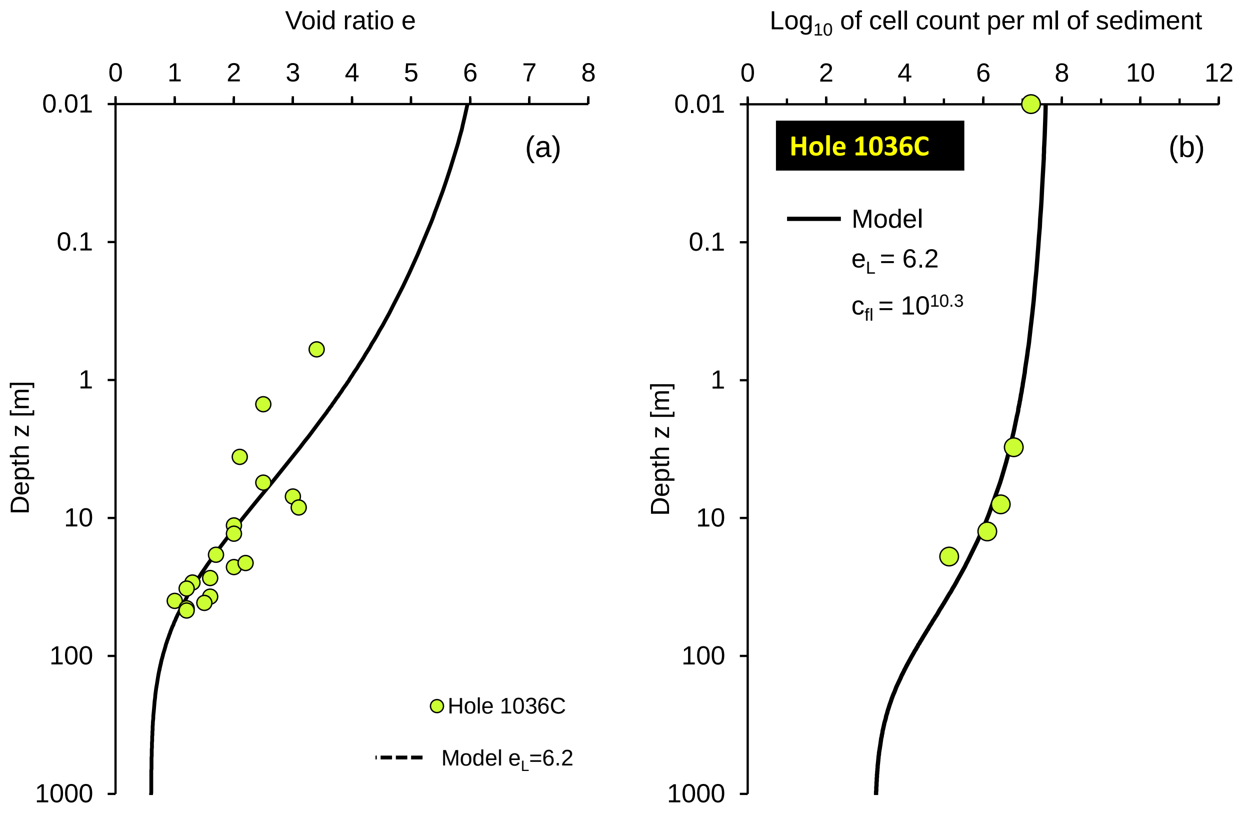

Supplementary Figure S31. Northeast Pacific: Leg 169 - Site 1036C. Void ratio and cell count data profiles versus depth and prediction models. (A) Void ratio depth profile - Site 1036C [Data extracted from (Ref. 61)] (model parameters: e_L = 6.2). (B) Cell count profile - Site 1036C [data extracted from (Ref. 60)] (the estimated cell concentration of the pore fluid c_{fl} = $10^{10.3}$ cell counts/cm^3).

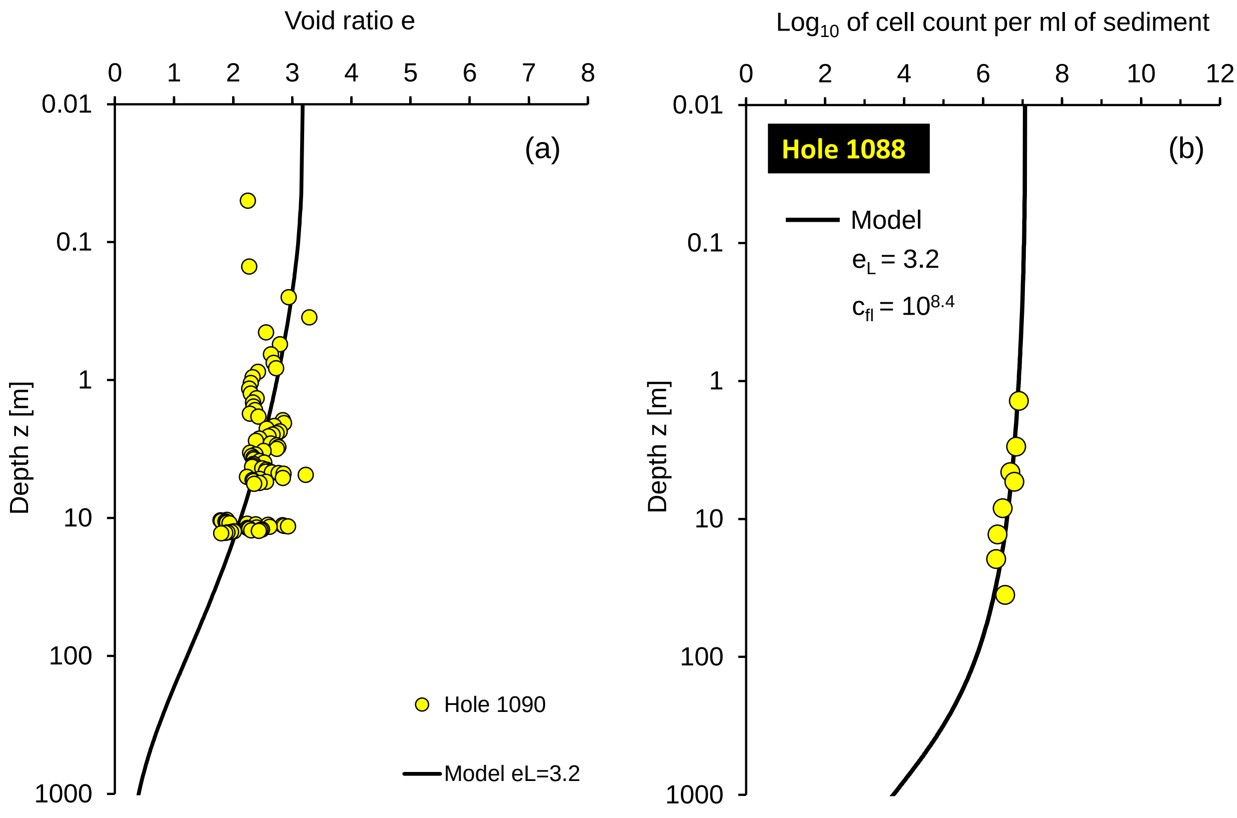

Supplementary Figure S32. Southern Ocean: Leg 177 - Site 1088. Void ratio and cell count data profiles versus depth and prediction models. (A) Void ratio depth profile - Site 1089 [Data extracted from (Ref. 64)] (model parameters: e_L = 3.2). (B) Cell count profile - Site 1088 [data extracted from (Ref. 65)] (the estimated cell concentration of the pore fluid c_{fl} = $10^{8.4}$ cell counts/cm^3).

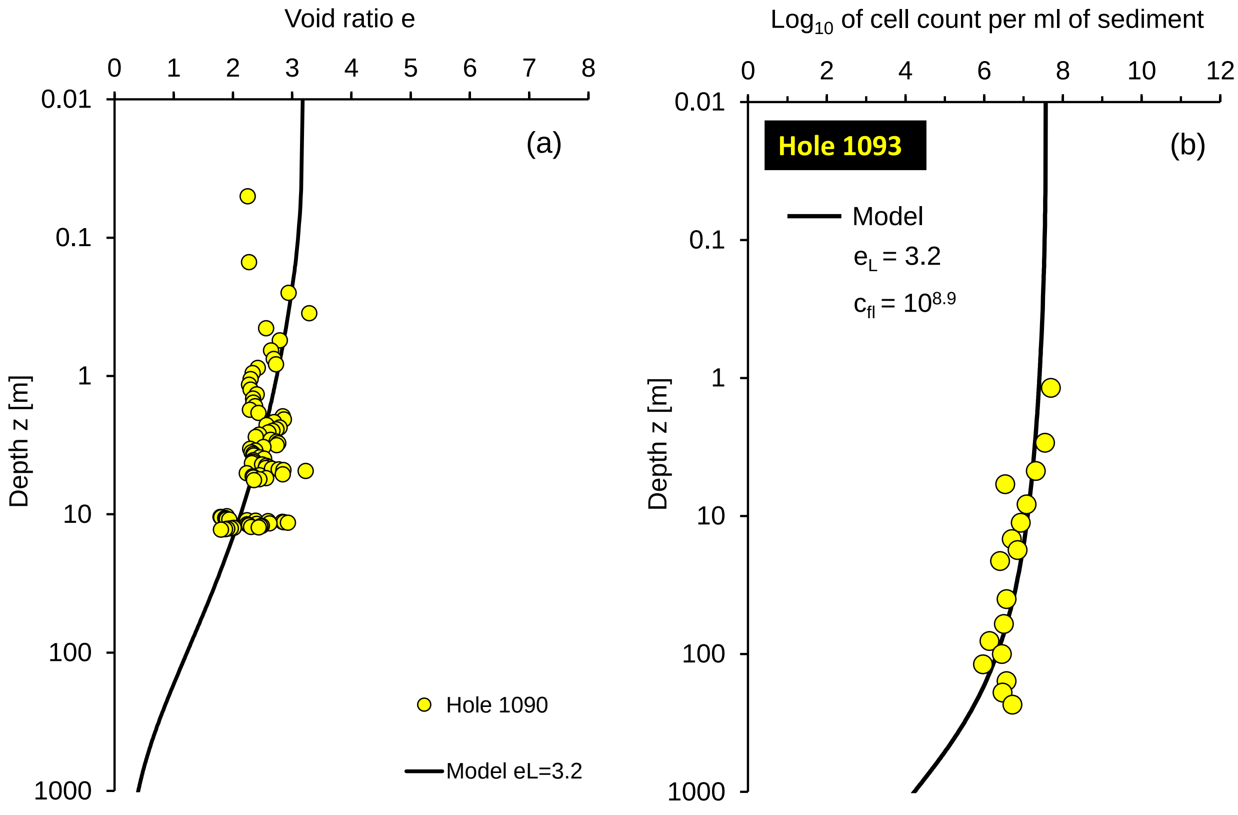

Supplementary Figure S33. Southern Ocean: Leg 177 - Site 1093. Void ratio and cell count data profiles versus depth and prediction models. (A) Void ratio depth profile - Site 1090 [Data extracted from (Ref. 66)] (model parameters: e_L = 3.2). (B) Cell count profile - Site 1093 [data extracted from (Ref. 65)] (the estimated cell concentration of the pore fluid c_{fl} = $10^{8.9}$ cell counts/cm^3).

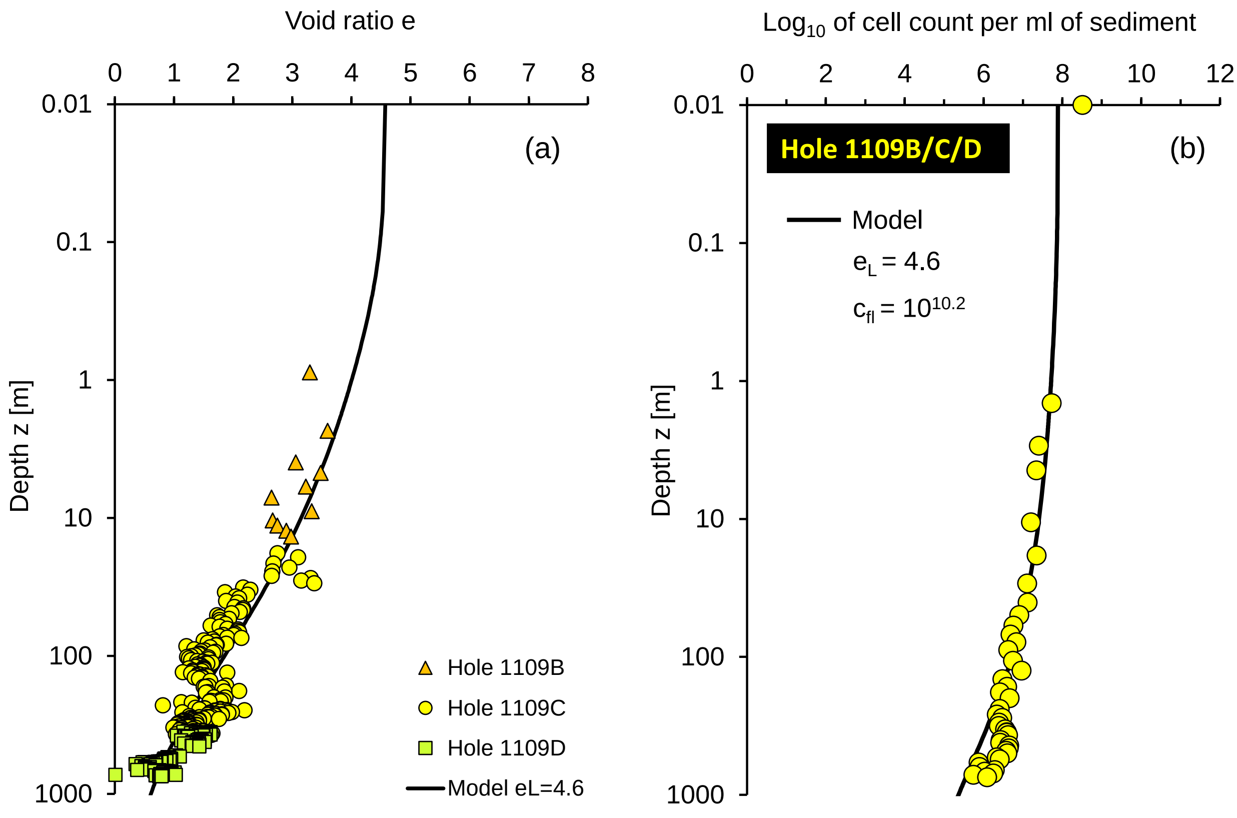

Supplementary Figure S34. Woodlark Basin: Leg 180 - Site 1109B/C/D. Void ratio and cell count data profiles versus depth and prediction models. (A) Void ratio depth profile - Site 1109B/C/D [Data extracted from (Ref. 68)] (model parameters: e_L = 4.6). (B) Cell count profile - Site 1109B/C/D [data extracted from (Ref. 68)] (the estimated cell concentration of the pore fluid c_{fl} = $10^{10.2}$ cell counts/cm^3).

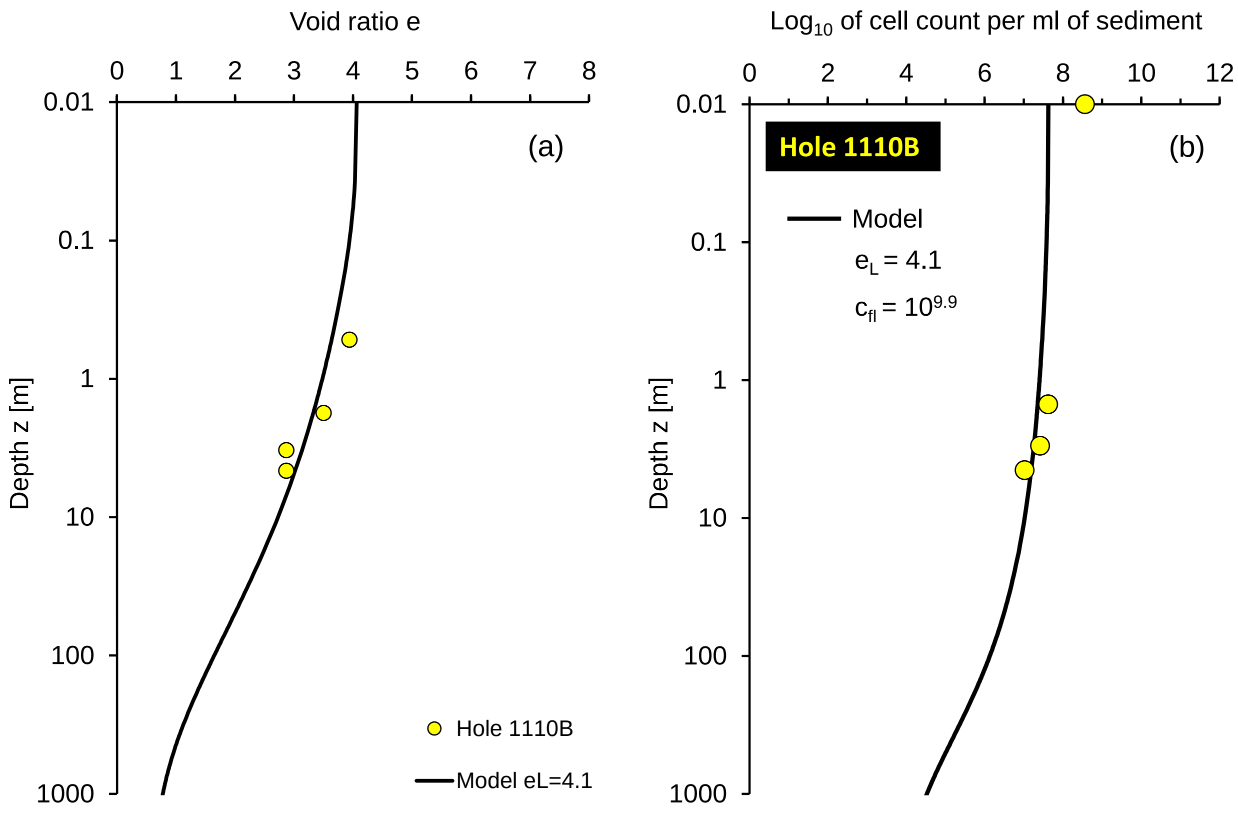

Supplementary Figure S35. Woodlark Basin: Leg 180 - Site 1110B. Void ratio and cell count data profiles versus depth and prediction models. (A) Void ratio depth profile - Site 1110B [Data extracted from (Ref. 69)] (model parameters: e_L = 4.1). (B) Cell count profile - Site 1110B [data extracted from (Ref. 69)] (the estimated cell concentration of the pore fluid c_{fl} = $10^{9.9}$ cell counts/cm^3).

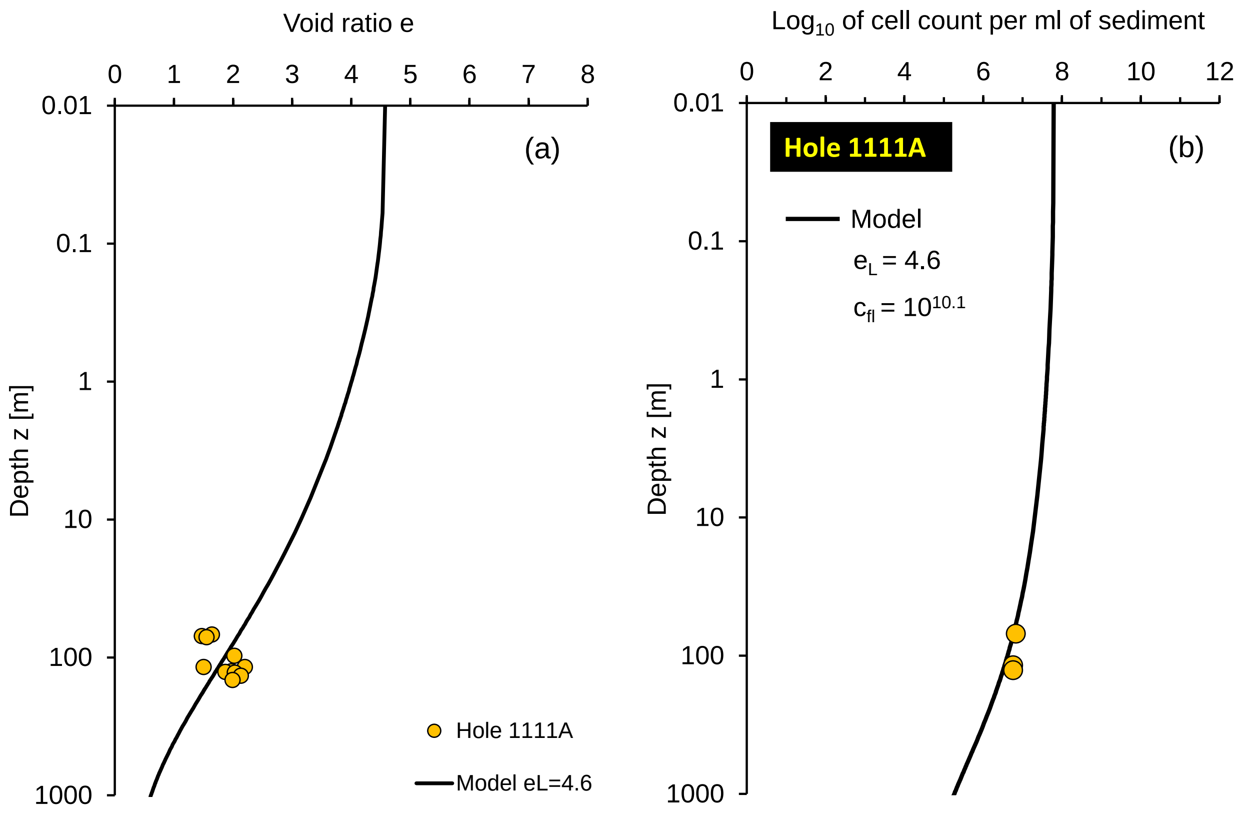

Supplementary Figure S36. Woodlark Basin: Leg 180 - Site 1111A. Void ratio and cell count data profiles versus depth and prediction models. (A) Void ratio depth profile - Site 1111A [Data extracted from (Ref. 69)] (model parameters: e_L = 4.6). (B) Cell count profile - Site 1111A [data extracted from (Ref. 69)] (the estimated cell concentration of the pore fluid c_{fl} = $10^{10.1}$ cell counts/cm^3).

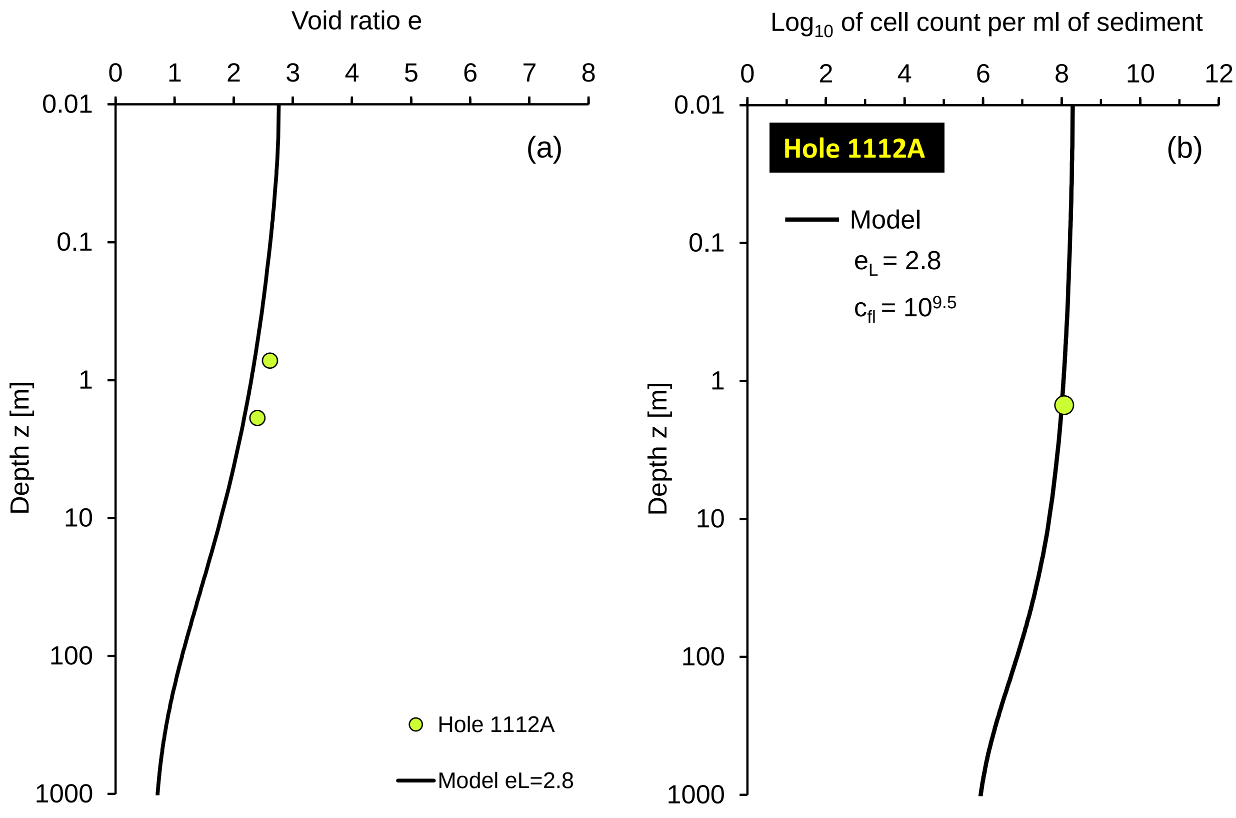

Supplementary Figure S37. Woodlark Basin: Leg 180 - Site 1112A. Void ratio and cell count data profiles versus depth and prediction models. (A) Void ratio depth profile - Site 1112A [Data extracted from (Ref. 69)] (model parameters: e_L = 2.8). (B) Cell count profile - Site 1112A [data extracted from (Ref. 69)] (the estimated cell concentration of the pore fluid c_{fl} = $10^{9.5}$ cell counts/cm^3).

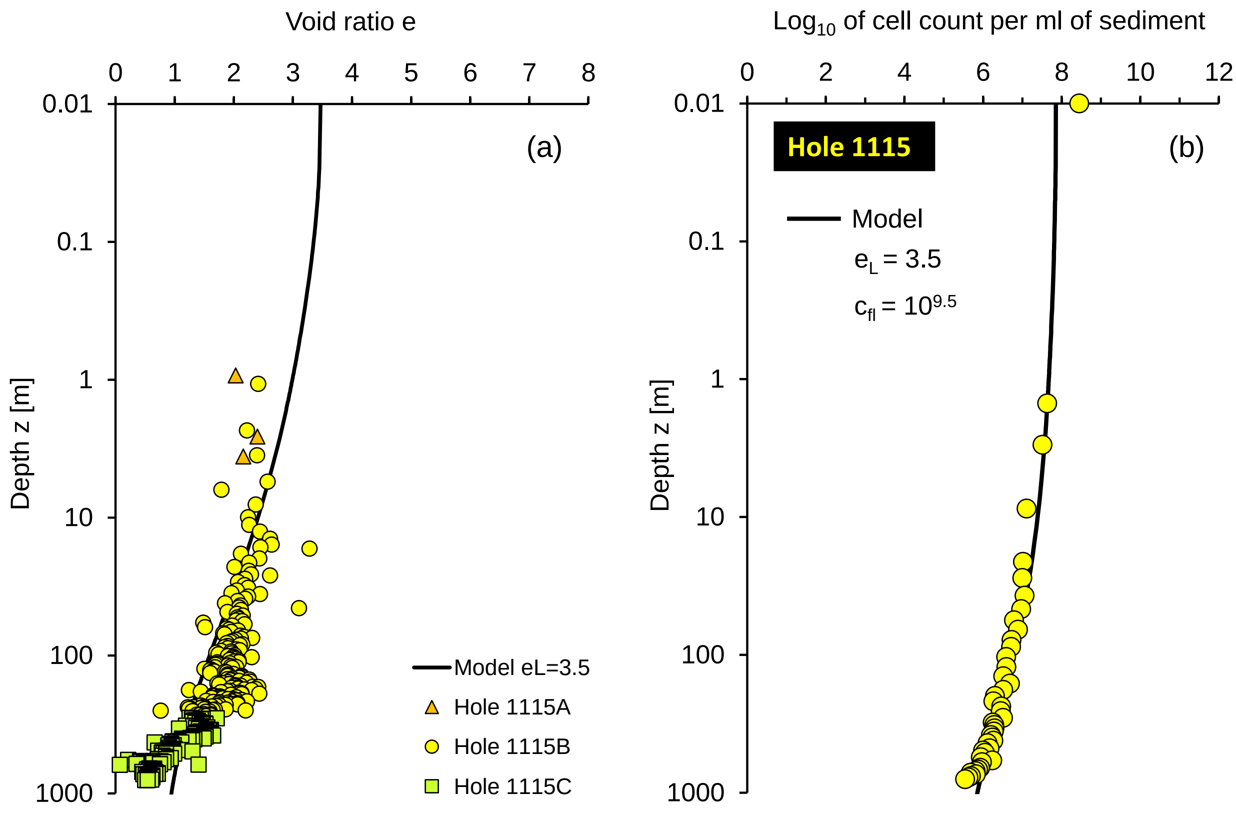

Supplementary Figure S38. Woodlark Basin: Leg 180 - Site 1115. Void ratio and cell count data profiles versus depth and prediction models. (A) Void ratio depth profile - Site 1115A/B/C [Data extracted from (Ref. 70)] (model parameters: e_L = 3.5). (B) Cell count profile - Site 1115 [data extracted from (Ref. 70)] (the estimated cell concentration of the pore fluid c_{fl} = $10^{9.5}$ cell counts/cm^3).

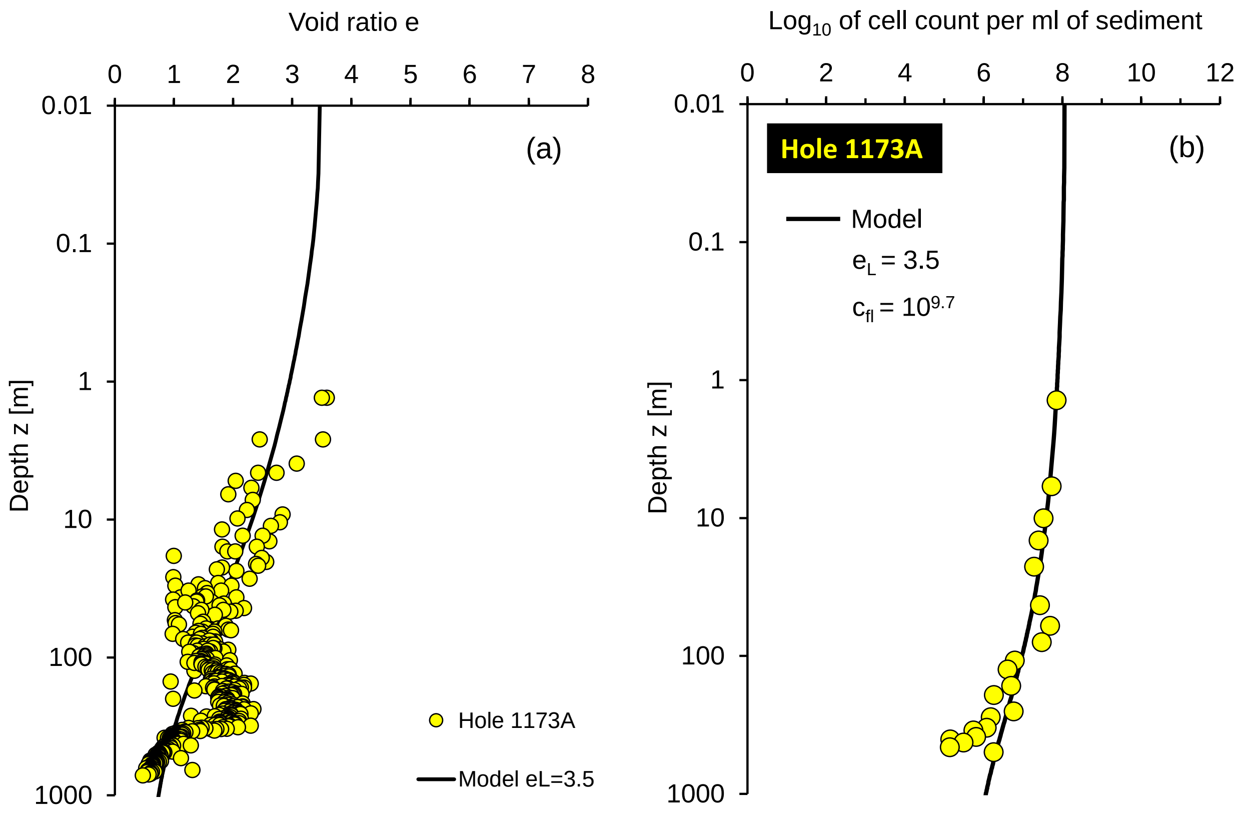

Supplementary Figure S39. Nakai Trough: Leg 190 - Site 1173A. Void ratio and cell count data profiles versus depth and prediction models. (A) Void ratio depth profile - Site 1173A [Data extracted from (Ref. 72)] (model parameters: e_L = 3.5). (B) Cell count profile - Site 1173A [data extracted from (Ref. 72)] (the estimated cell concentration of the pore fluid c_{fl} = $10^{9.7}$ cell counts/cm^3).

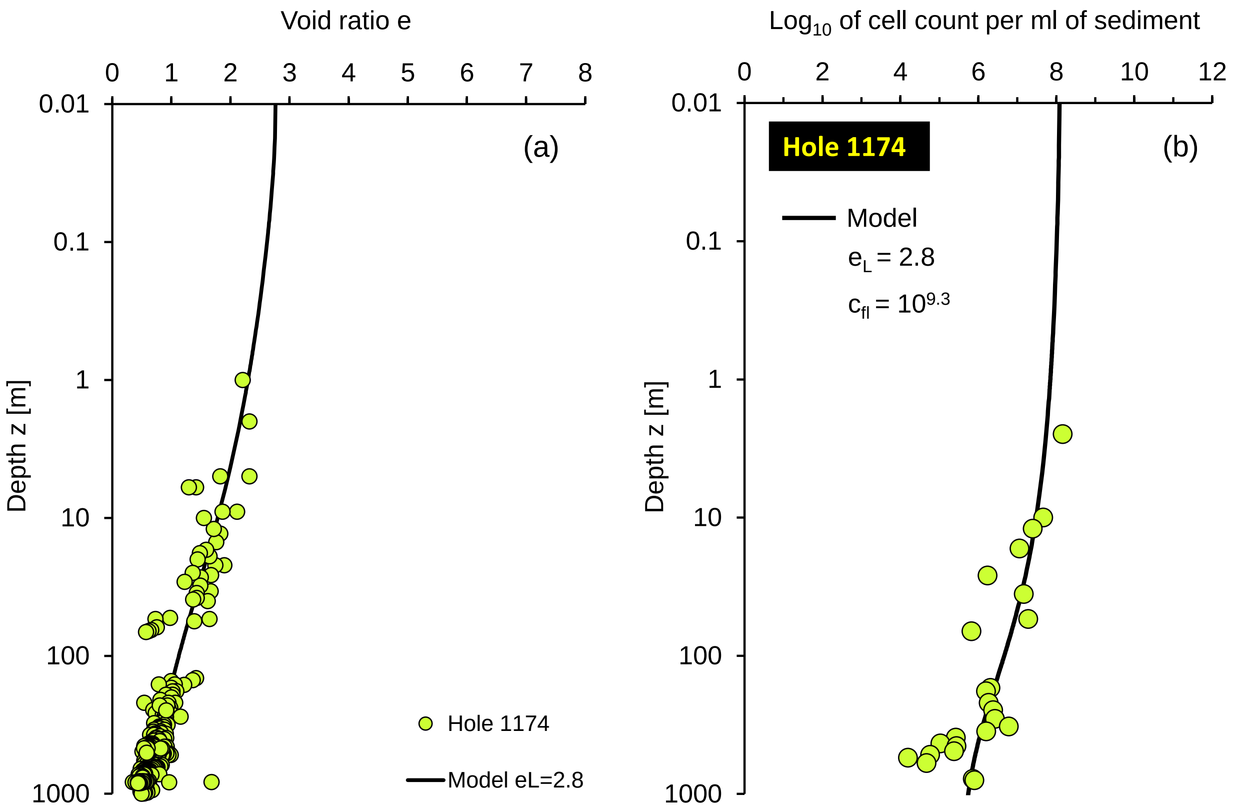

Supplementary Figure S40. Nakai Trough: Leg 190 - Site 1174. Void ratio and cell count data profiles versus depth and prediction models. (A) Void ratio depth profile - Site 1174 [Data extracted from (Ref. 73)] (model parameters: e_L = 2.8). (B) Cell count profile - Site 1174 [data extracted from (Ref. 73)] (the estimated cell concentration of the pore fluid c_{fl} = $10^{9.3}$ cell counts/cm^3).

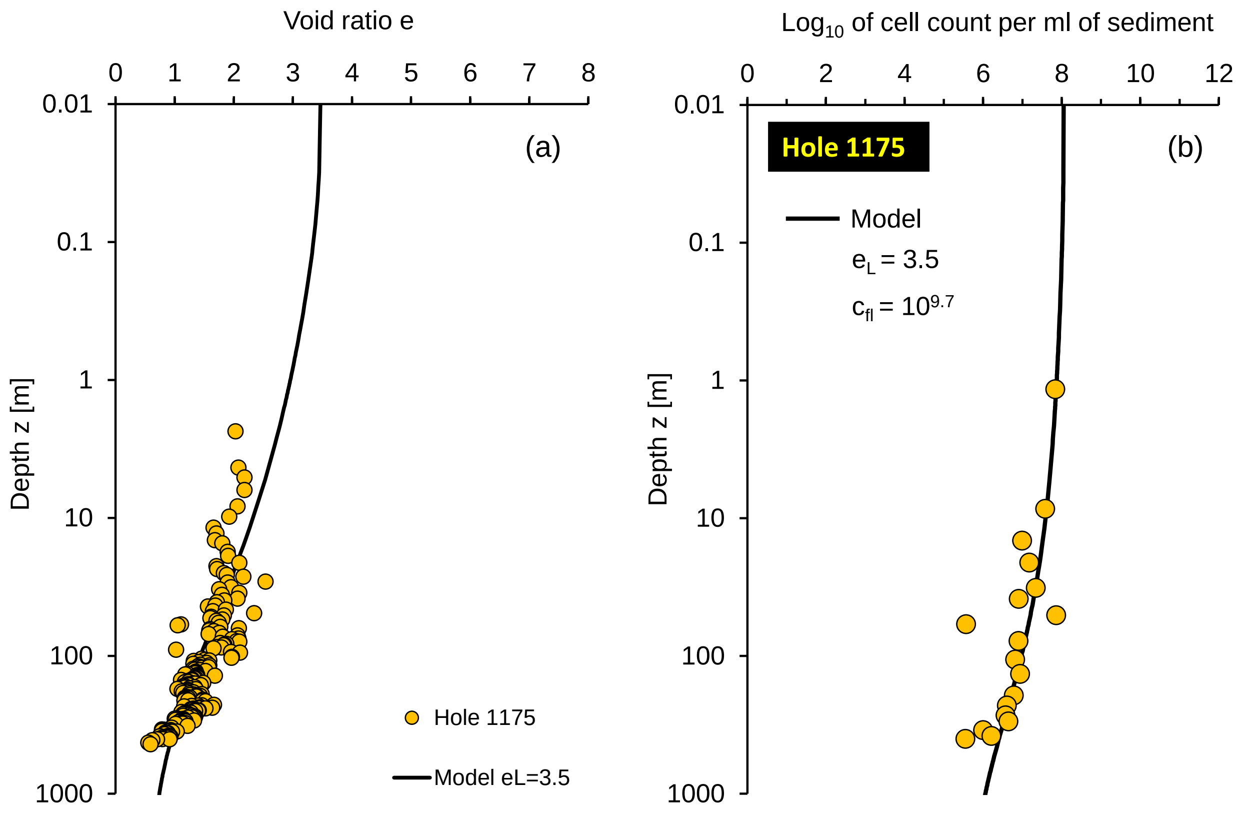

Supplementary Figure S41. Nakai Trough: Leg 190 - Site 1175. Void ratio and cell count data profiles versus depth and prediction models. (A) Void ratio depth profile - Site 1175 [Data extracted from (Ref. 74)] (model parameters: e_L = 3.5). (B) Cell count profile - Site 1175 [data extracted from (Ref. 74)] (the estimated cell concentration of the pore fluid c_{fl} = $10^{9.7}$ cell counts/cm^3).

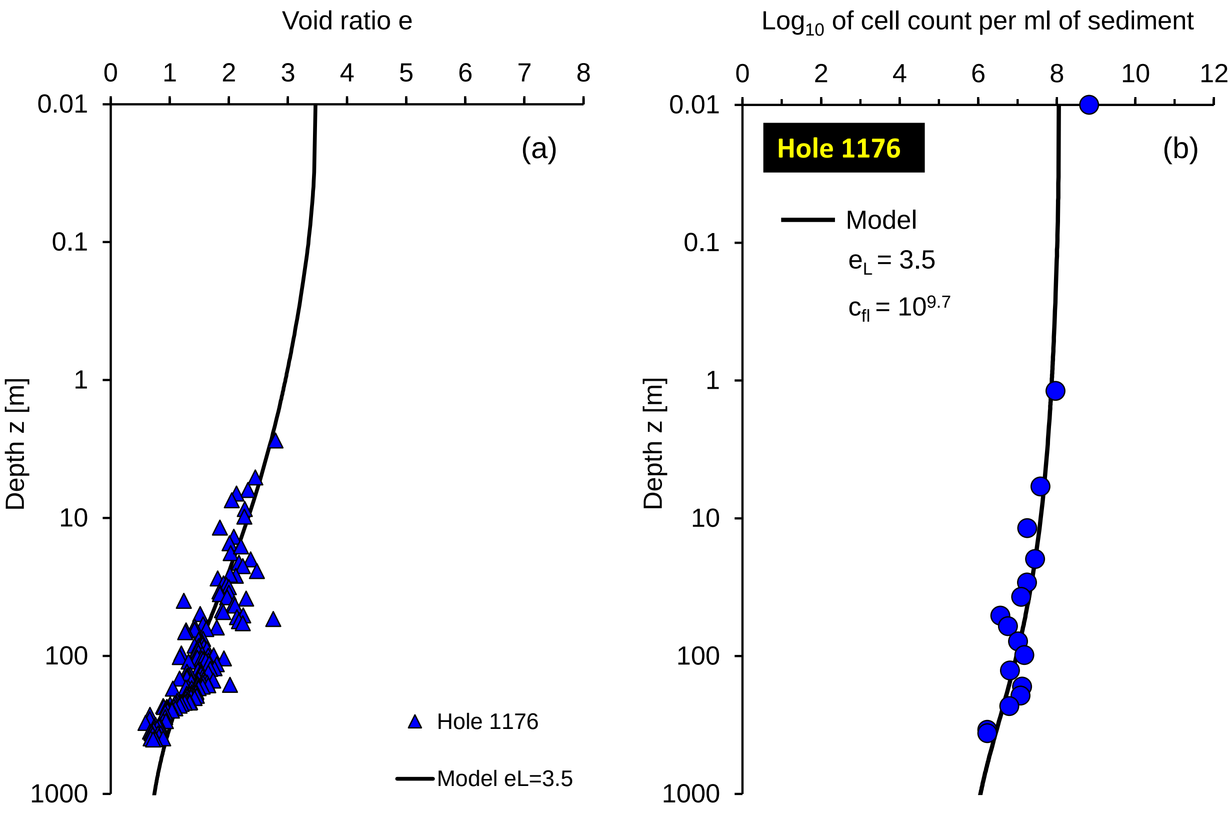

Supplementary Figure S42. Nakai Trough: Leg 190 - Site 1176. Void ratio and cell count data profiles versus depth and prediction models. (A) Void ratio depth profile - Site 1176 [Data extracted from (Ref. 75)] (model parameters: e_L = 3.5). (B) Cell count profile - Site 1176 [data extracted from (Ref. 75)] (the estimated cell concentration of the pore fluid c_{fl} = $10^{9.7}$ cell counts/cm^3).

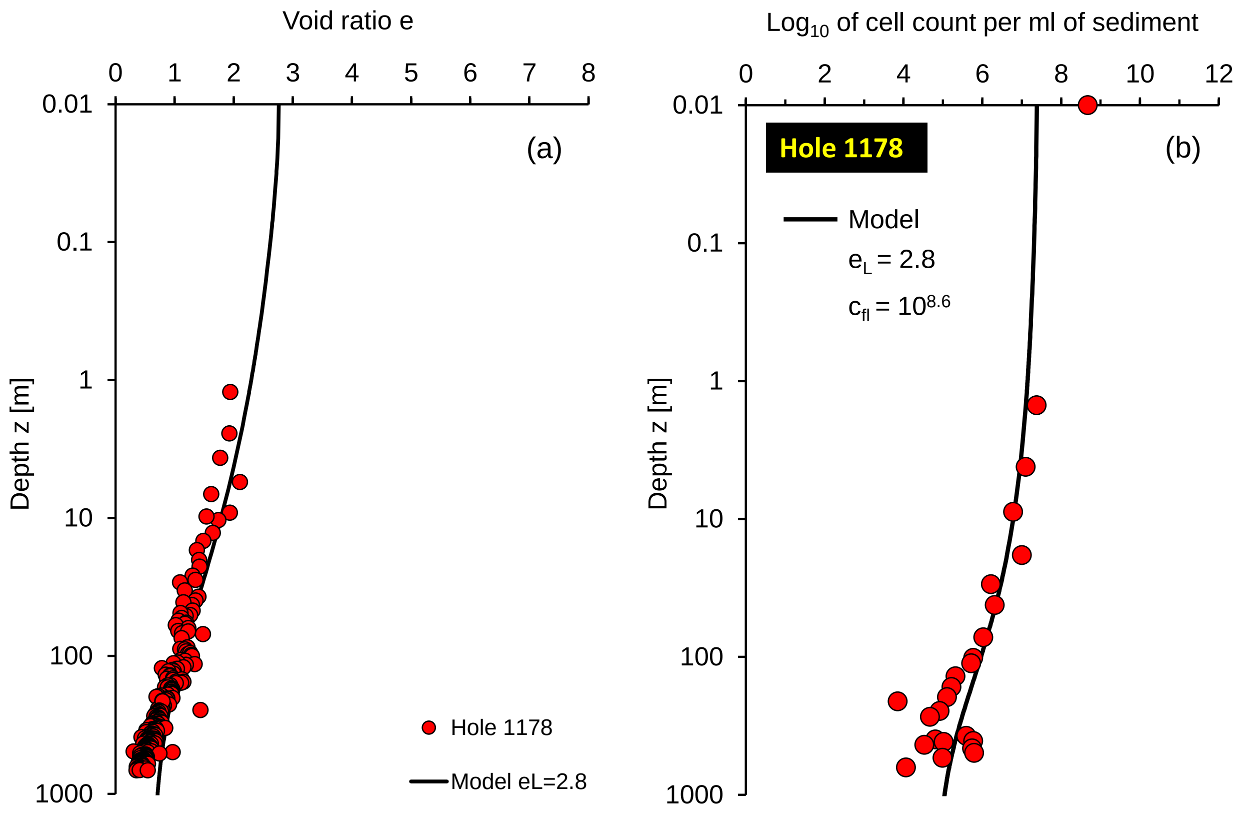

Supplementary Figure S43. Nakai Trough: Leg 190 - Site 1178. Void ratio and cell count data profiles versus depth and prediction models. (A) Void ratio depth profile - Site 1178 [Data extracted from (Ref. 77)] (model parameters: e_L = 2.8). (B) Cell count profile - Site 1178 [data extracted from (Ref. 77)] (the estimated cell concentration of the pore fluid c_{fl} = $10^{8.6}$ cell counts/cm^3).

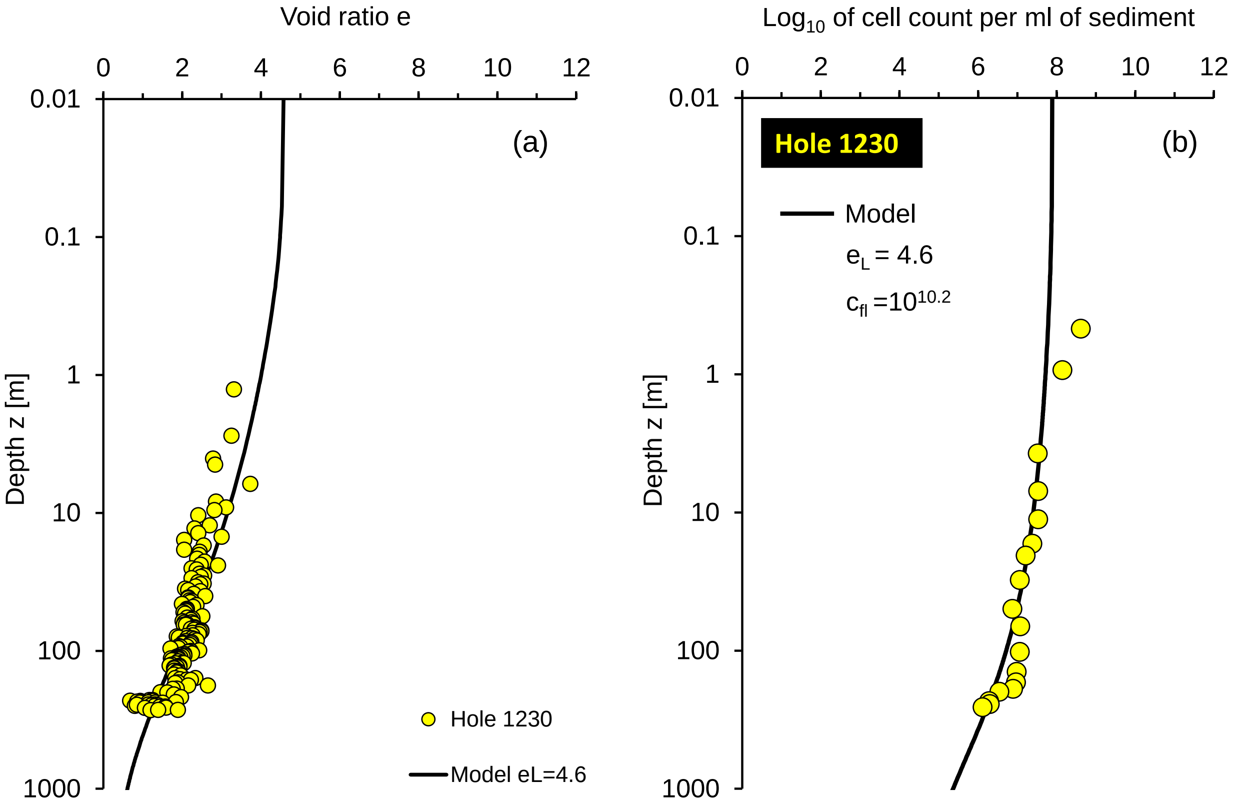

Supplementary Figure S44. Peru Margin: Leg 201 - Site 1230. Void ratio and cell count data profiles versus depth and prediction models. (A) Void ratio depth profile - Site 1230 [Data extracted from (Ref. 82)] (model parameters: e_L = 4.6). (B) Cell count profile - Site 1230 [data extracted from (Ref. 82)] (the estimated cell concentration of the pore fluid c_{fl} = $10^{10.2}$ cell counts/cm^3).

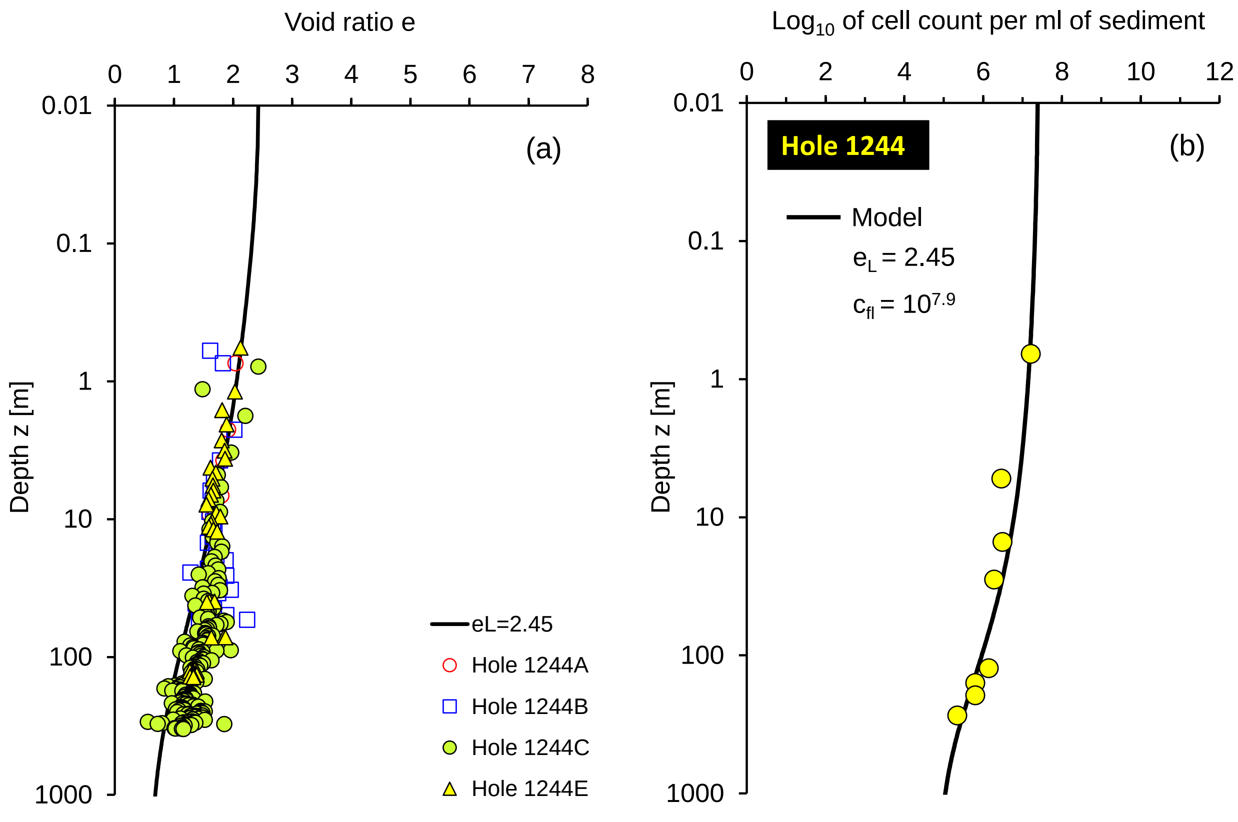

Supplementary Figure S45. Cascadia Margin: Leg 204 - Site 1244/1245. Void ratio and cell count data profiles versus depth and prediction models. (A) Void ratio depth profile - Site 1244A/B/C/E [Data extracted from (Ref. 84)] (model parameters: $e_L = 2.45$). (B) Cell count profile - Site 1244/1245 [data extracted from (Ref. 85)] (the estimated cell concentration of the pore fluid $c_{fl} = 10^{7.9}$ cell counts/cm^3).

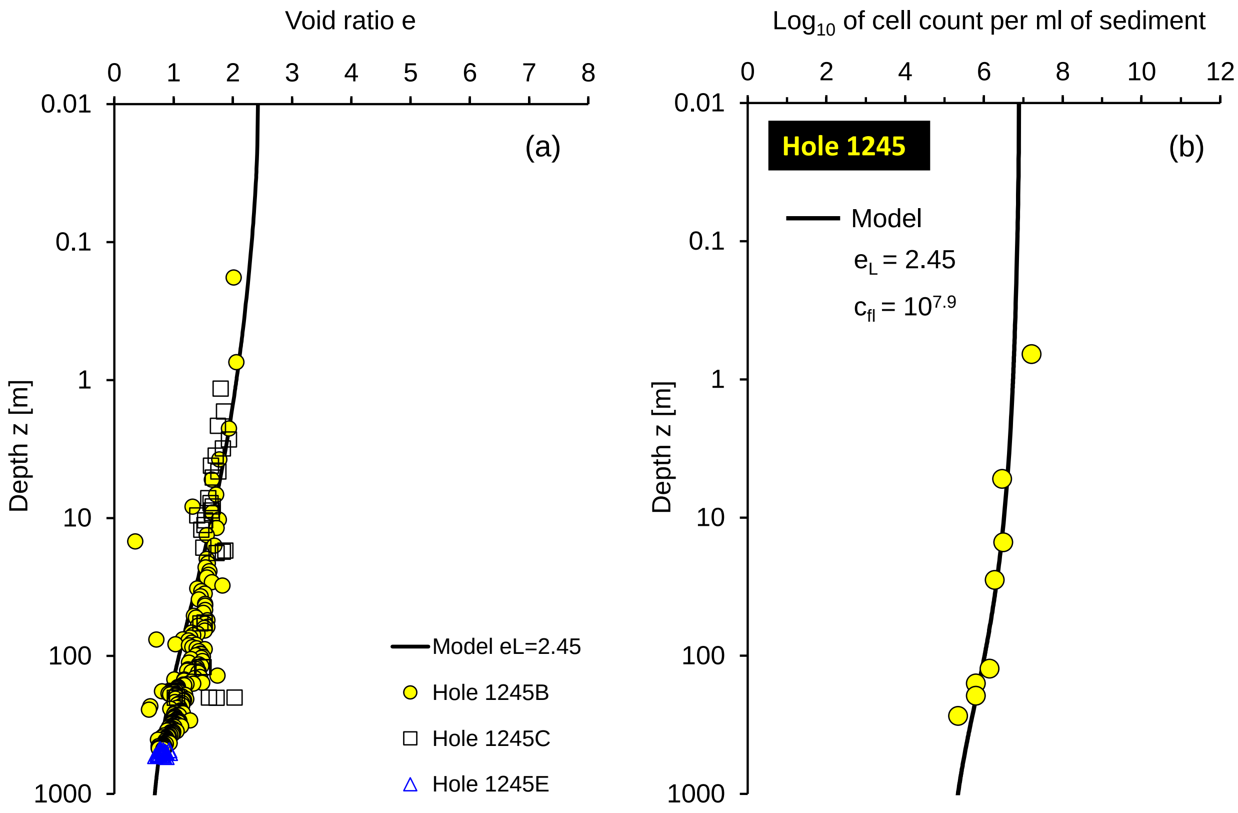

Supplementary Figure S46. Cascadia Margin: Leg 204 - Site 1244/1245. Void ratio and cell count data profiles versus depth and prediction models. (A) Void ratio depth profile - Site 1245B/C/E [Data extracted from (Ref. 86)] (model parameters: $e_L = 2.45$). (B) Cell count profile - Site 1244/1245 [data extracted from (Ref. 85)] (the estimated cell concentration of the pore fluid $c_{fl} = 10^{7.9}$ cell counts/cm^3).

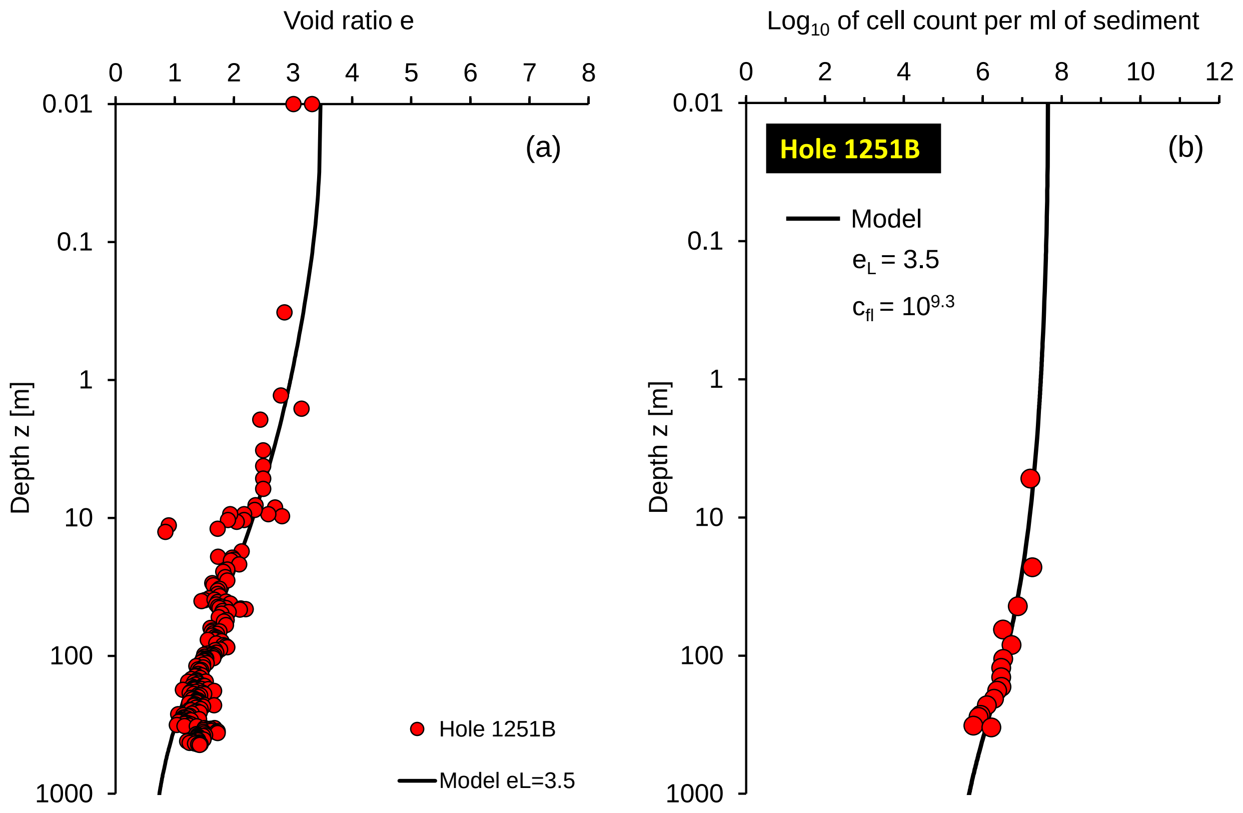

Supplementary Figure S47. Cascadia Margin: Leg 204 - Site 1251B. Void ratio and cell count data profiles versus depth and prediction models. (A) Void ratio depth profile - Site 1251B [Data extracted from (Ref. 87)] (model parameters: e_L = 3.5). (B) Cell count profile - Site 1251B [data extracted from (Ref. 85)] (the estimated cell concentration of the pore fluid c_{fl} = $10^{9.3}$ cell counts/cm^3).

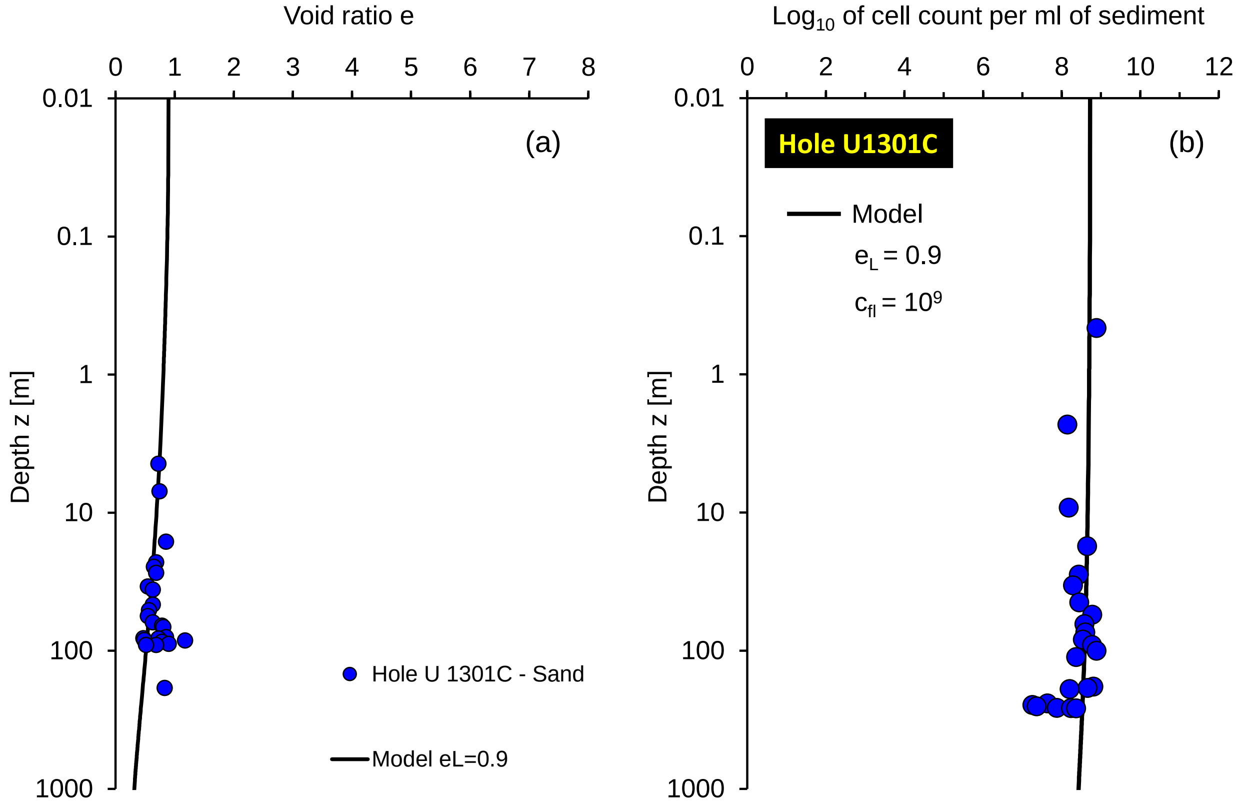

Supplementary Figure S48. Juan de Fuca Ridge: Leg 301 - Site U1301C. Void ratio and cell count data profiles versus depth and prediction models. (A) Void ratio depth profile - U1301C [Data extracted from (Ref. 88)] (model parameters: e_L = 0.9). (B) Cell count profile - U1301C [data extracted from (Ref. 88)] (the estimated cell concentration of the pore fluid c_{fl} = 10^9 cell counts/cm^3).

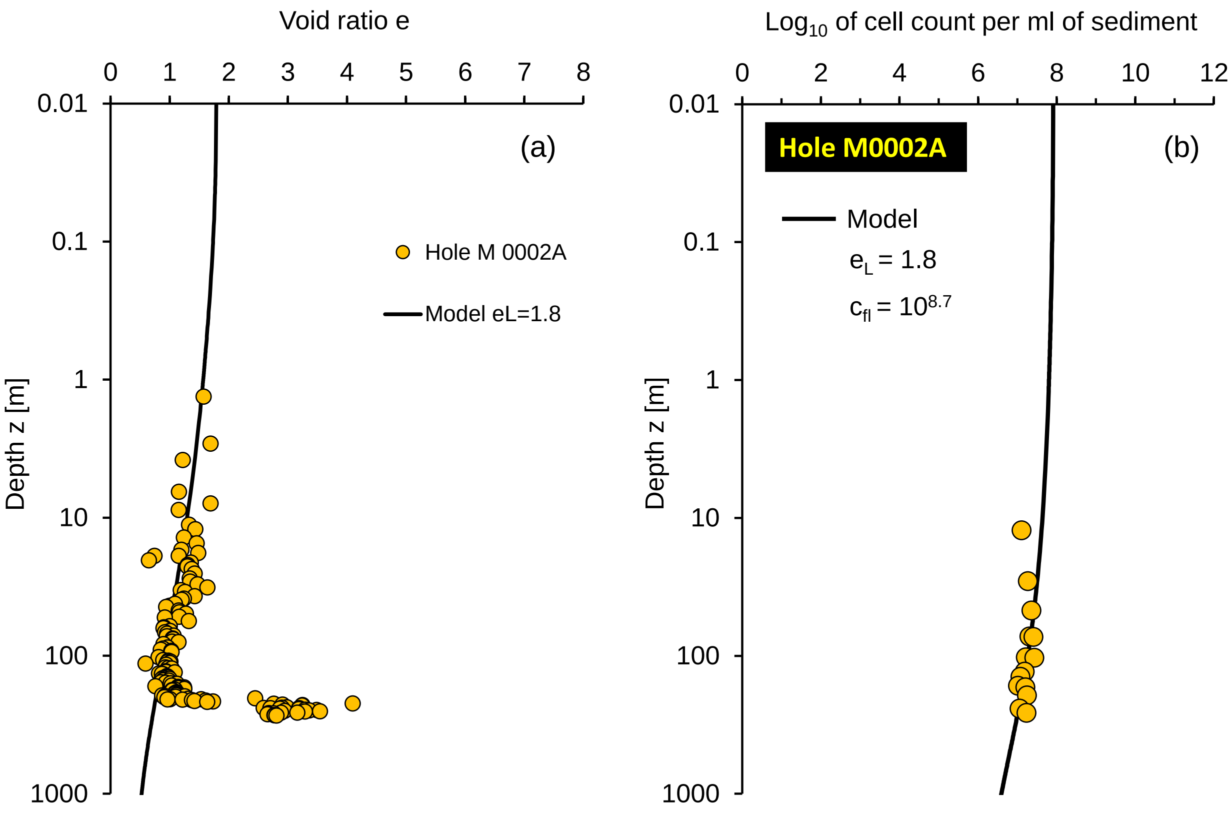

Supplementary Figure S49. Lomonosov Ridge: Leg 302 - Site M0002A. Void ratio and cell count data profiles versus depth and prediction models. (A) Void ratio depth profile - M0002A [Data extracted from (Ref. 89)] (model parameters: e_L = 1.8). (B) Cell count profile - M0002A [data extracted from (Ref. 90)] (the estimated cell concentration of the pore fluid c_{fl} = $10^{8.7}$ cell counts/cm^3).

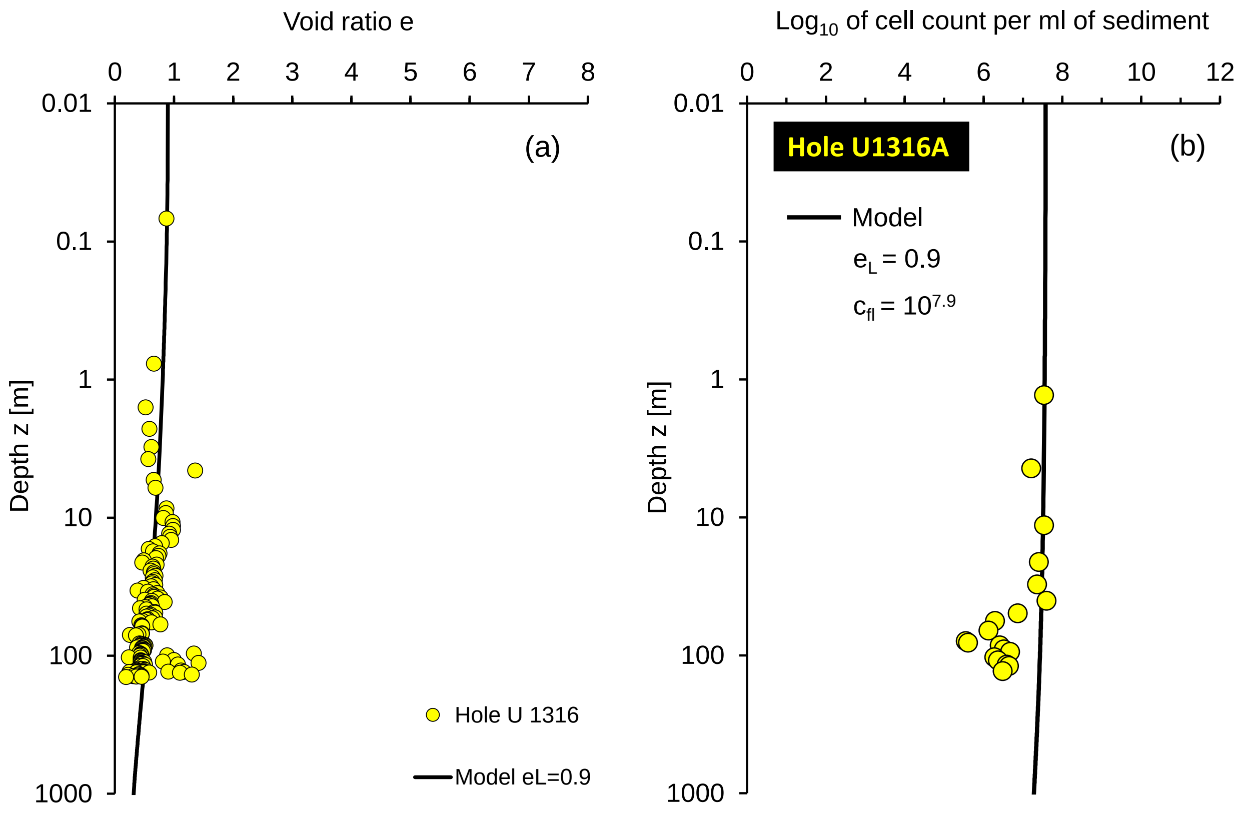

Supplementary Figure S50. Porcupine Seamount: Leg 307 - Site U1316A. Void ratio and cell count data profiles versus depth and prediction models. (A) Void ratio depth profile - Site U1316A [Data extracted from (Ref. 91)] (model parameters: $e_L = 0.9$). (B) Cell count profile - Site U1316A [data extracted from (Ref. 91)] (the estimated cell concentration of the pore fluid $c_{fl} = 10^{7.9}$ cell counts/cm^3).

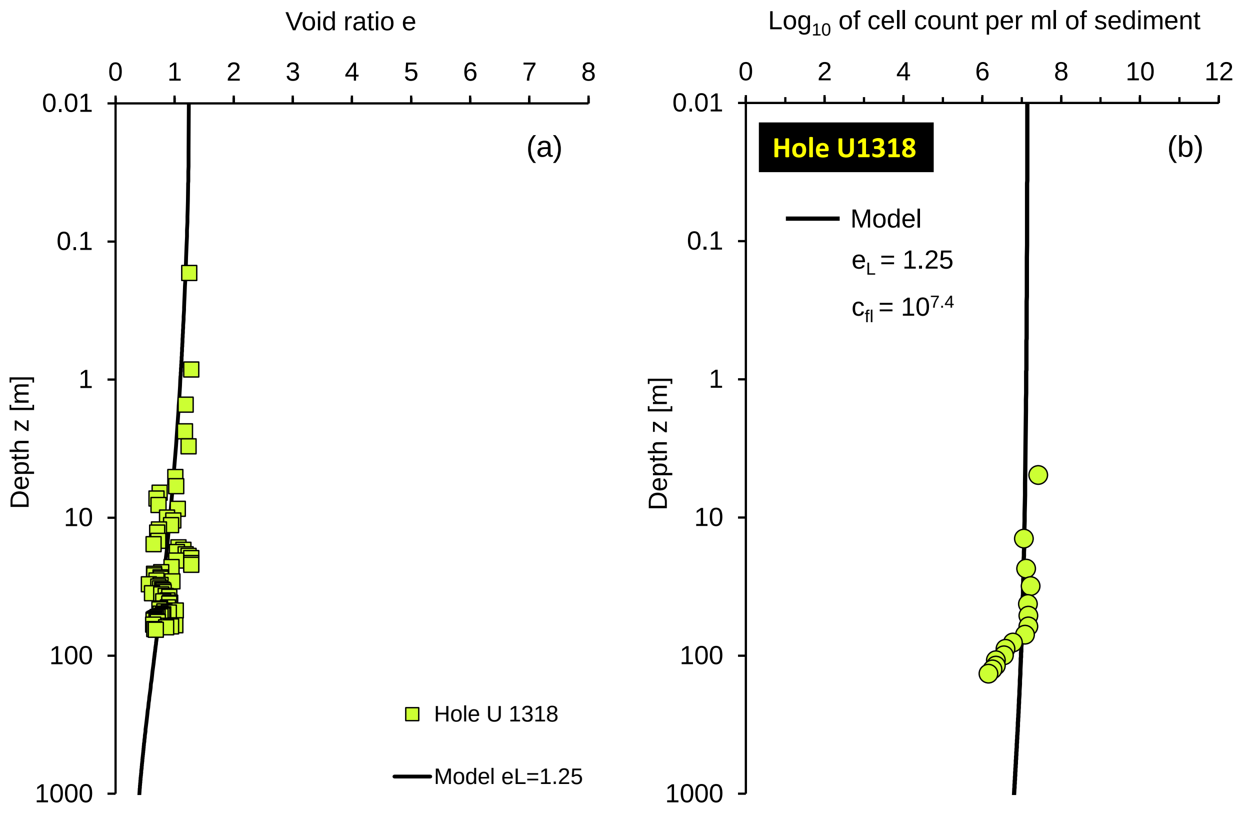

Supplementary Figure S51. Porcupine Seamount: Leg 307 - Site U1318. Void ratio and cell count data profiles versus depth and prediction models. (A) Void ratio depth profile - Site U1318 [Data extracted from (Ref. 93)] (model parameters: $e_L = 1.25$). (B) Cell count profile - Site U1318 [data extracted from (Ref. 93)] (the estimated cell concentration of the pore fluid $c_{fl} = 10^{7.4}$ cell counts/cm^3).

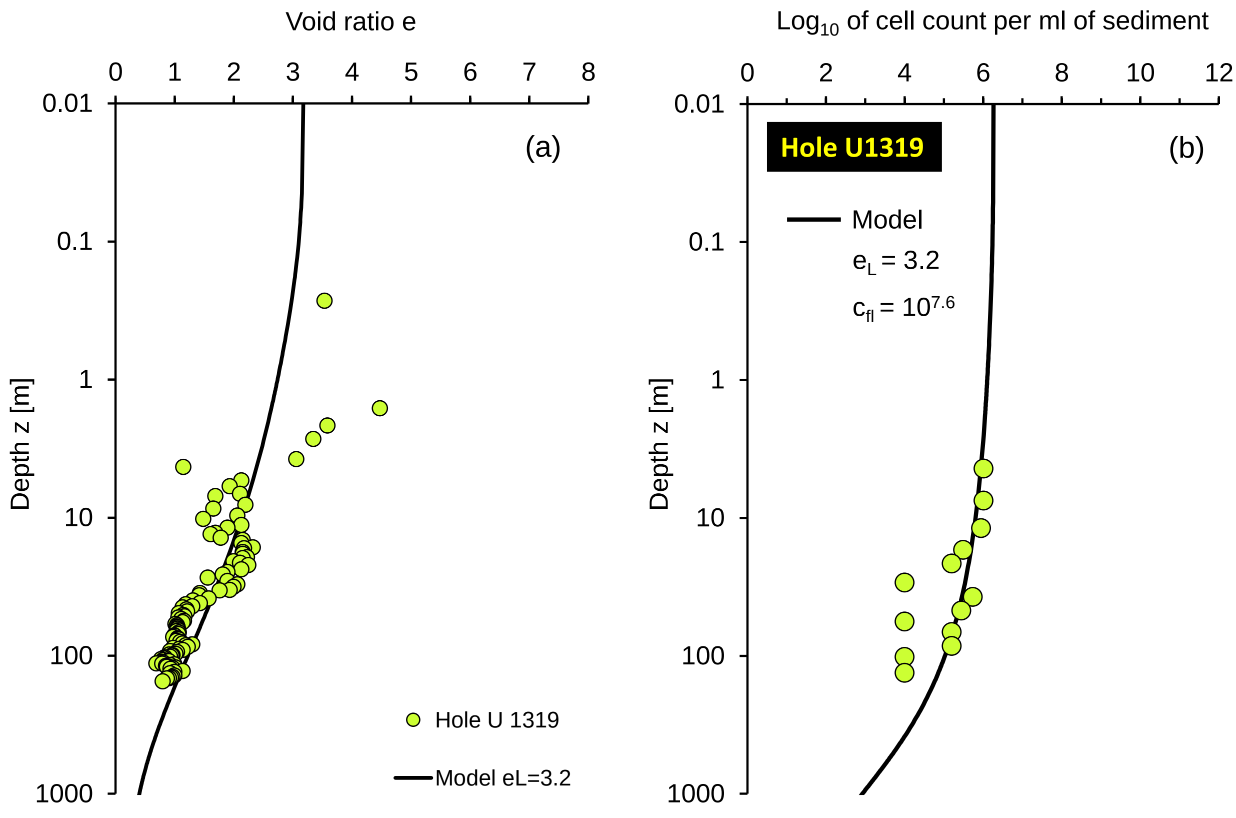

Supplementary Figure S52. Gulf of Mexico: Leg 308 - Site U1319. Void ratio and cell count data profiles versus depth and prediction models. (A) Void ratio depth profile - Site U1319 [Data extracted from (Ref. 94)] (model parameters: $e_L = 3.2$). (B) Cell count profile - Site U1319 [data extracted from (Ref. 94)] (the estimated cell concentration of the pore fluid $c_{fl} = 10^{7.6}$ cell counts/cm^3).

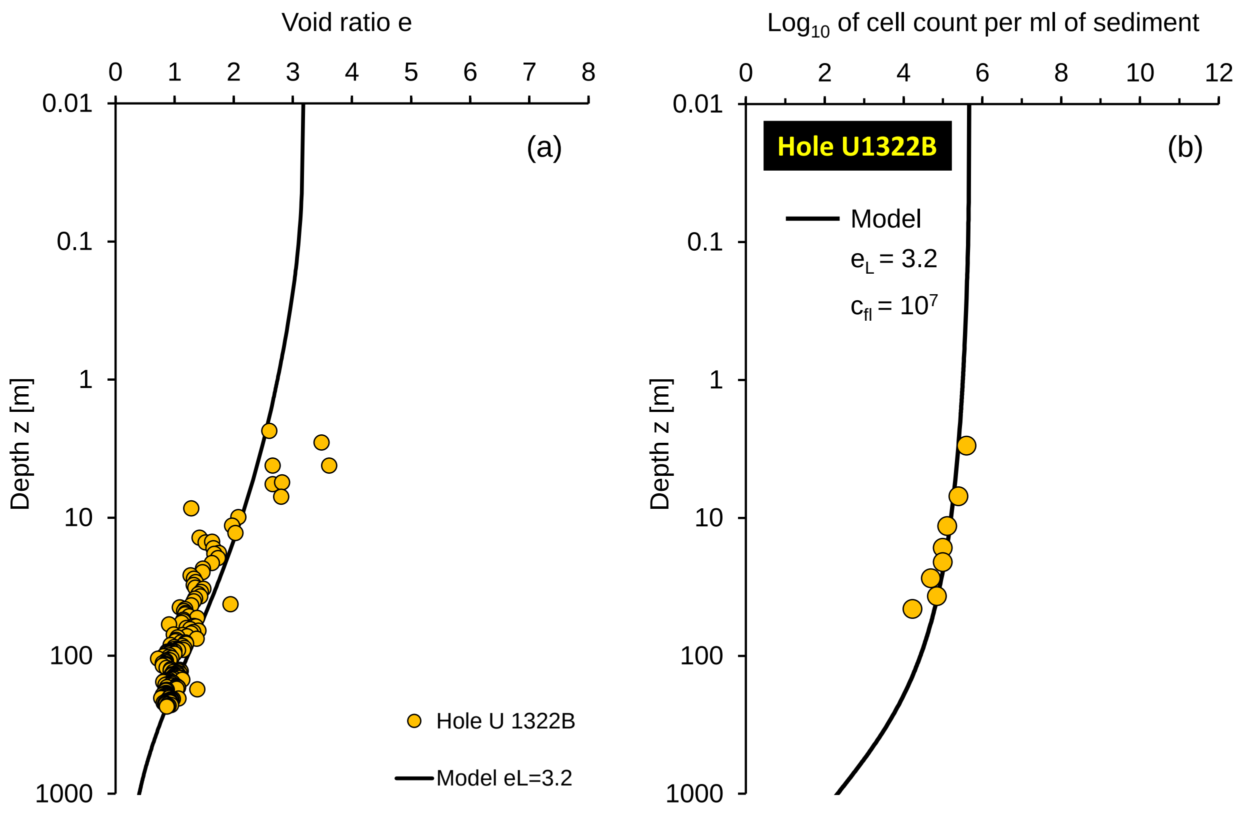

Supplementary Figure S53. Gulf of Mexico: Leg 308 - Site U1322B. Void ratio and cell count data profiles versus depth and prediction models. (A) Void ratio depth profile - Site U1322B [Data extracted from (Ref. 96)] (model parameters: e_L = 3.2). (B) Cell count profile - Site U1322B [data extracted from (Ref. 96)] (the estimated cell concentration of the pore fluid c_{fl} = 10^7 cell counts/cm^3).

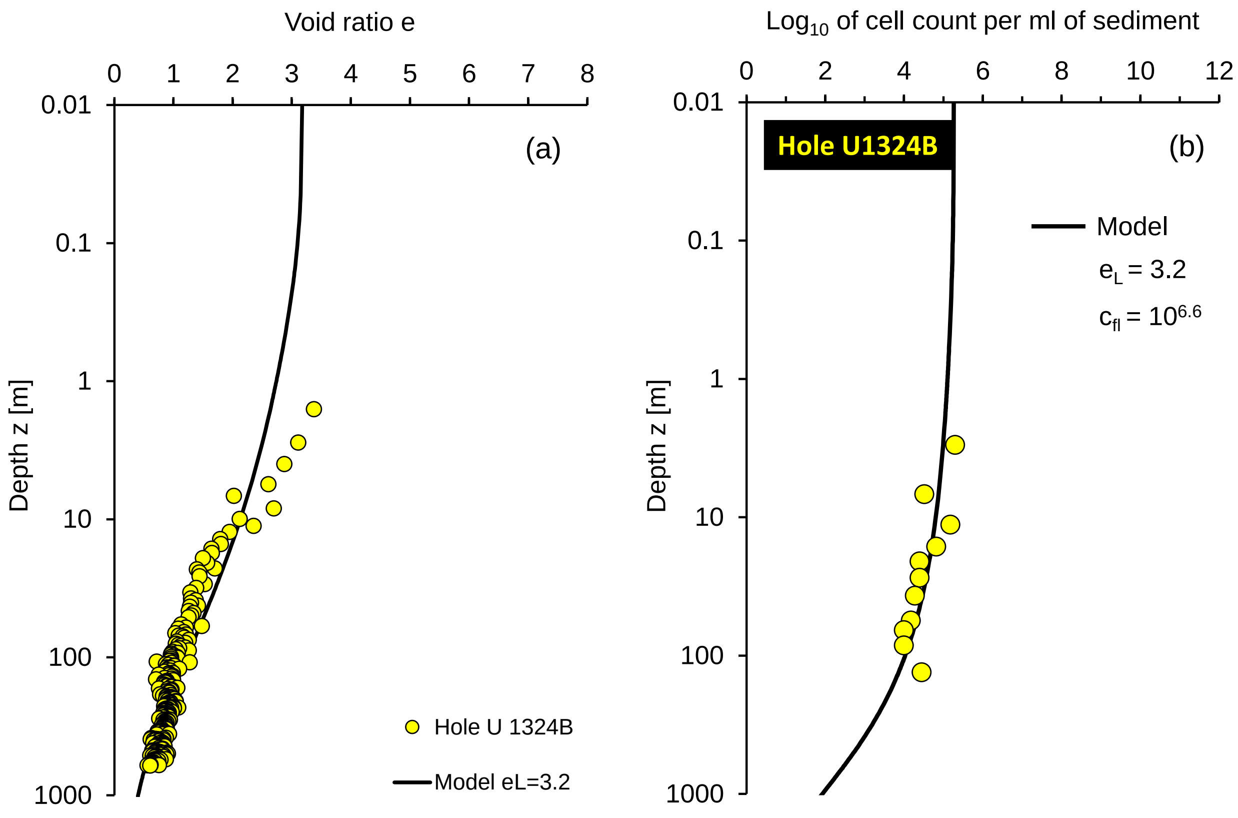

Supplementary Figure S54. Gulf of Mexico: Leg 308 - Site U1324B. Void ratio and cell count data profiles versus depth and prediction models. (A) Void ratio depth profile - Site U1324B [Data extracted from (Ref. 97)] (model parameters: e_L = 3.2). (B) Cell count profile - Site U1324B [data extracted from (Ref. 97)] (the estimated cell concentration of the pore fluid c_{fl} = $10^{6.6}$ cell counts/cm^3).

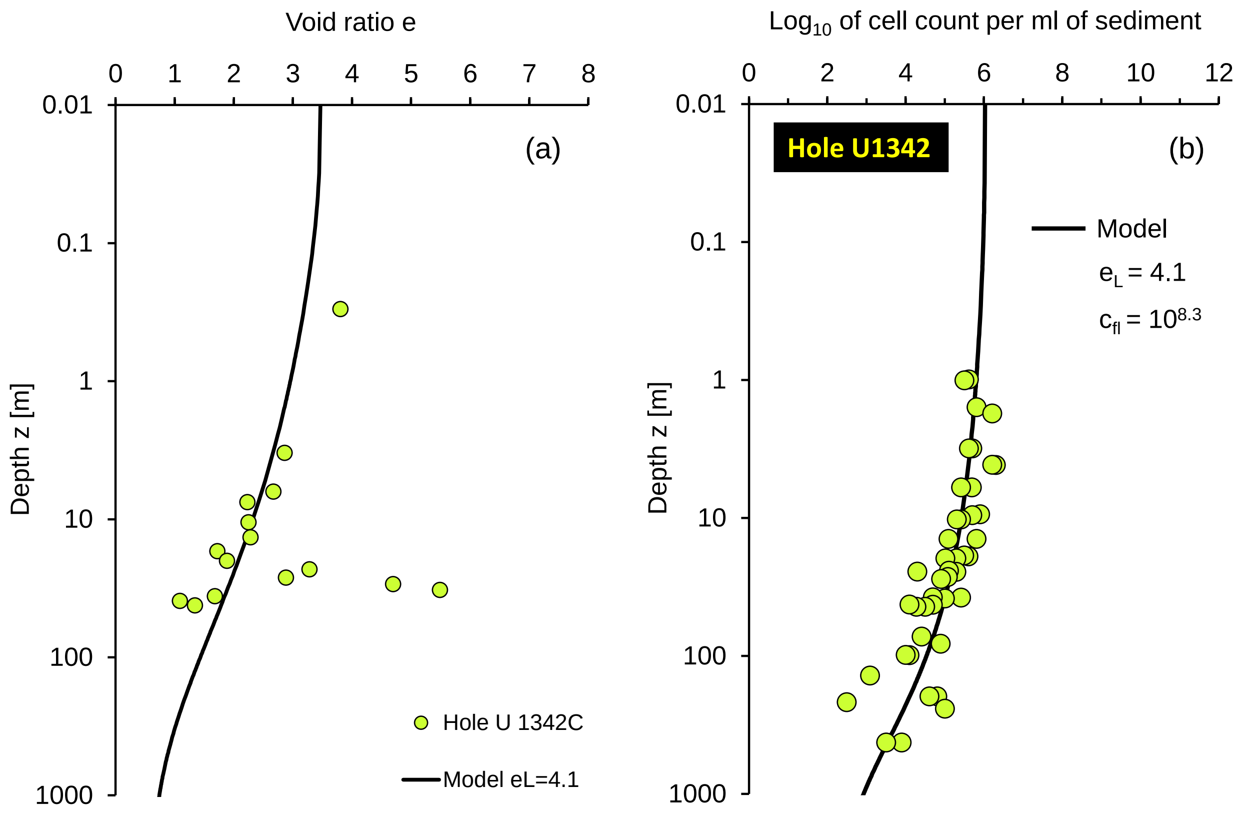

Supplementary Figure S55. Bering Sea: Leg 323 - Site U1342/1343. Void ratio and cell count data profiles versus depth and prediction models. (A) Void ratio depth profile - Site U1342C [Data extracted from (Ref. 105)] (model parameters: e_L = 4.1). (B) Cell count profile - Site U1342 [data extracted from (Ref. 106)] (the estimated cell concentration of the pore fluid c_{fl} = $10^{8.3}$ cell counts/cm^3).

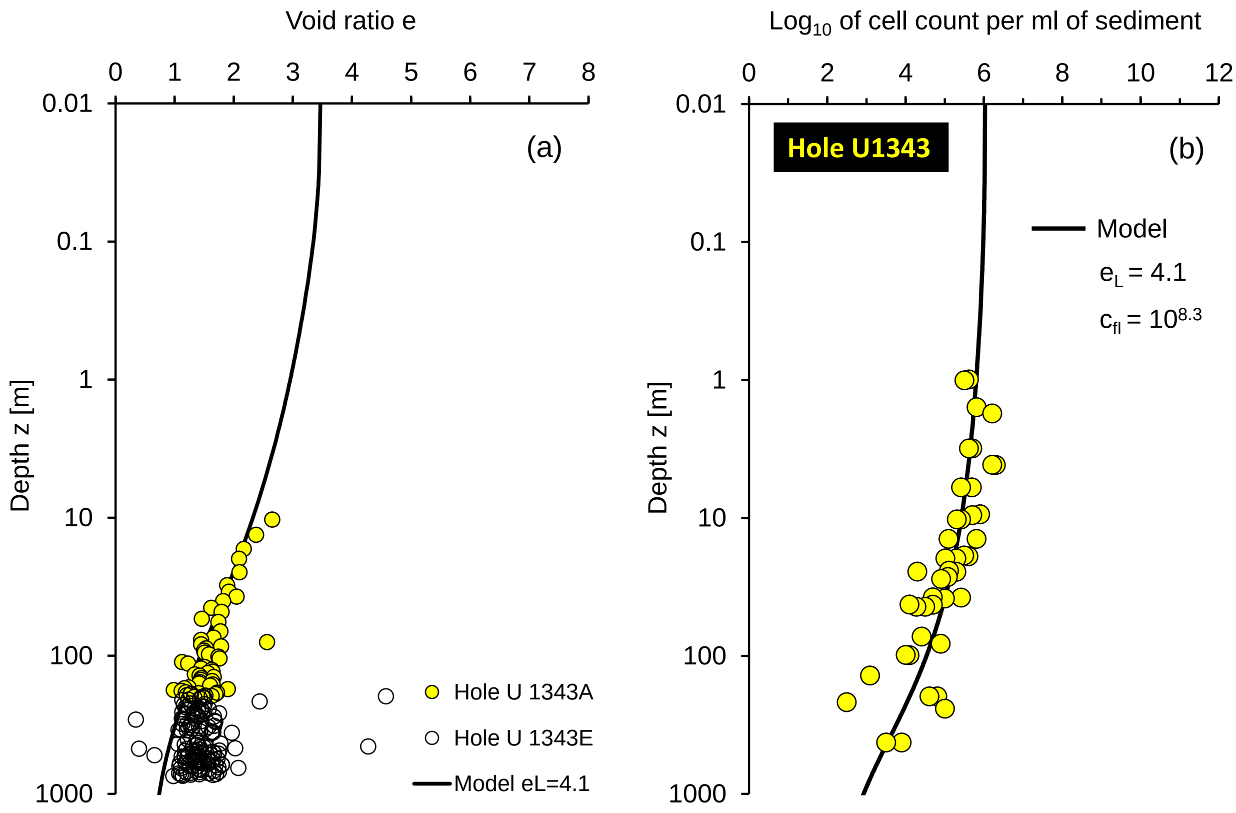

Supplementary Figure S56. Bering Sea: Leg 323 - Site U1343/1343. Void ratio and cell count data profiles versus depth and prediction models. (A) Void ratio depth profile - Site U1343A/E [Data extracted from (Ref. 107)] (model parameters: e_L = 4.1). (B) Cell count profile - Site U1343 [data extracted from (Ref. 106)] (the estimated cell concentration of the pore fluid c_{fl} = $10^{8.3}$ cell counts/cm^3).

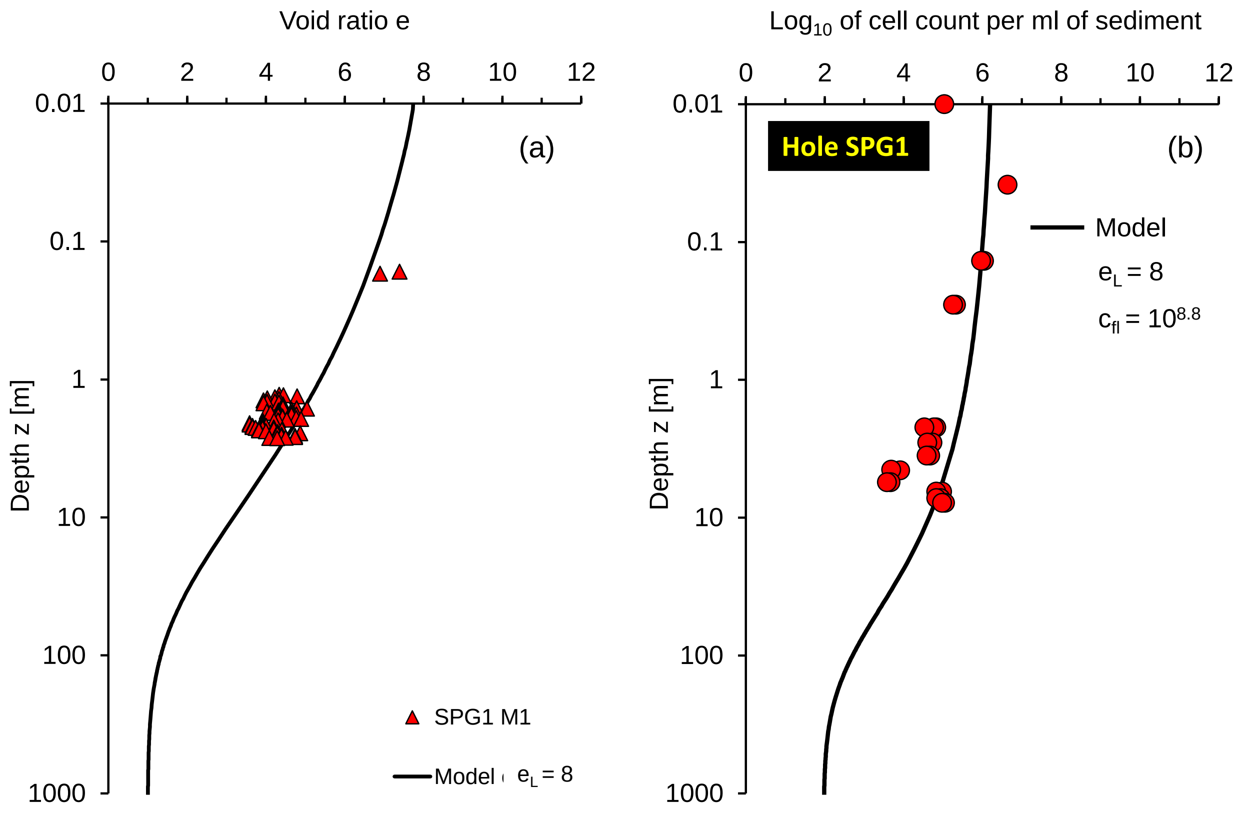

Supplementary Figure S57. South Pacific Gyre: Leg 329 - Site SPG1. Void ratio and cell count data profiles versus depth and prediction models. (A) Void ratio depth profile - Site SPG1 M1 [Data extracted from (Ref. 108)] (model parameters: e_L = 8). (B) Cell count profile - Site SPG1 [data extracted from (Ref. 108)] (the estimated cell concentration of the pore fluid $c_{fl} = 10^{8.8}$ cell counts/cm^3).

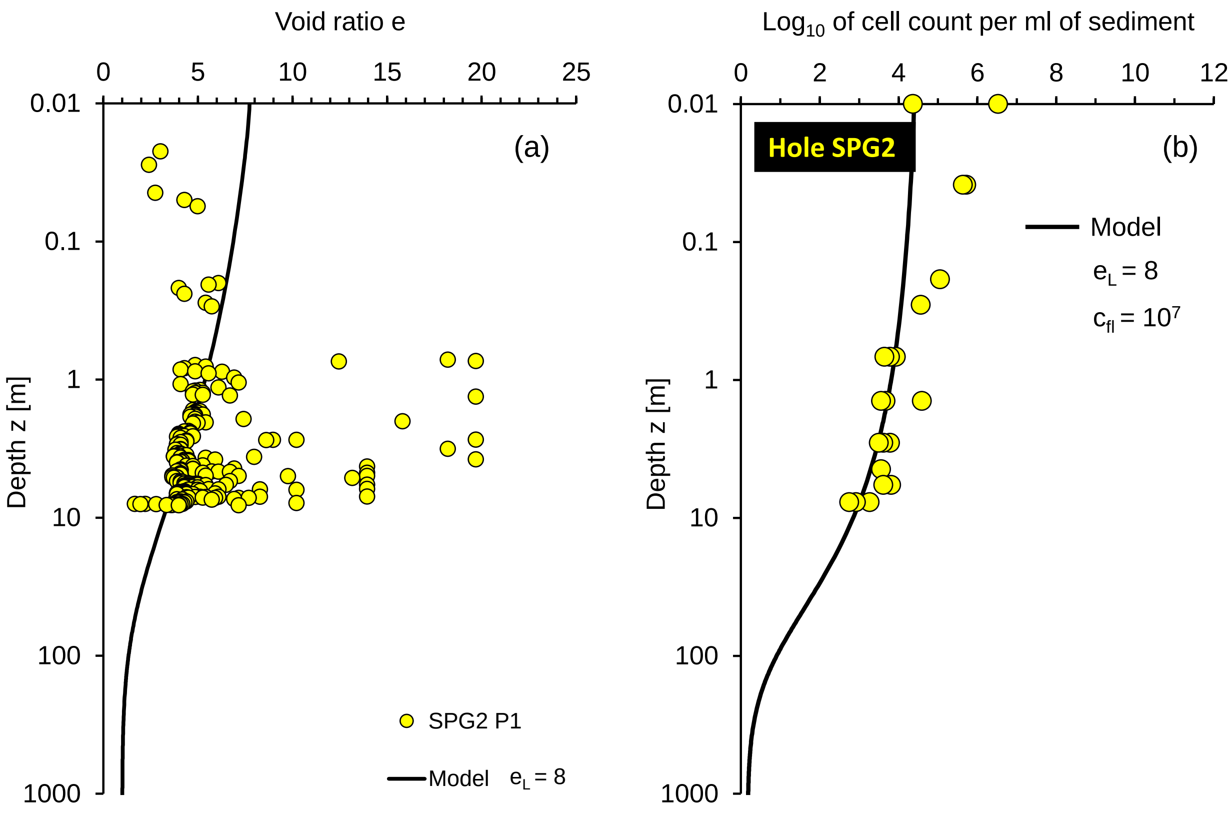

Supplementary Figure S58. South Pacific Gyre: Leg 329 - Site SPG2. Void ratio and cell count data profiles versus depth and prediction models. (A) Void ratio depth profile - Site SPG2 P1 [Data extracted from (Ref. 108)] (model parameters: $e_L = 8$). (B) Cell count profile - Site SPG2 [data extracted from (Ref. 108)] (the estimated cell concentration of the pore fluid $c_{fl} = 10^7$ cell counts/cm^3).

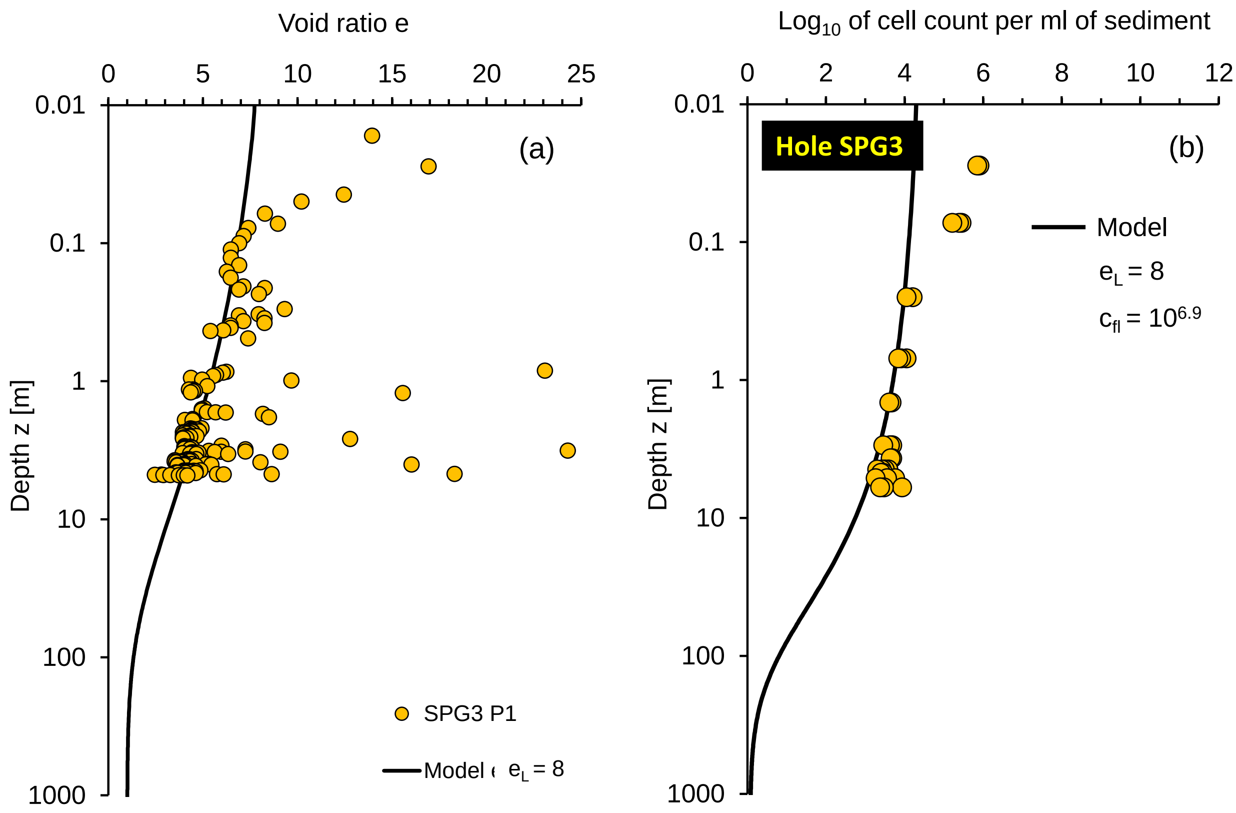

Supplementary Figure S59. South Pacific Gyre: Leg 329 - Site SPG3. Void ratio and cell count data profiles versus depth and prediction models. (A) Void ratio depth profile - Site SPG3 P1 [Data extracted from (Ref. 108)] (model parameters: e_L = 8). (B) Cell count profile - Site SPG3 [data extracted from (Ref. 108)] (the estimated cell concentration of the pore fluid c_{fl} = $10^{6.9}$ cell counts/cm^3).

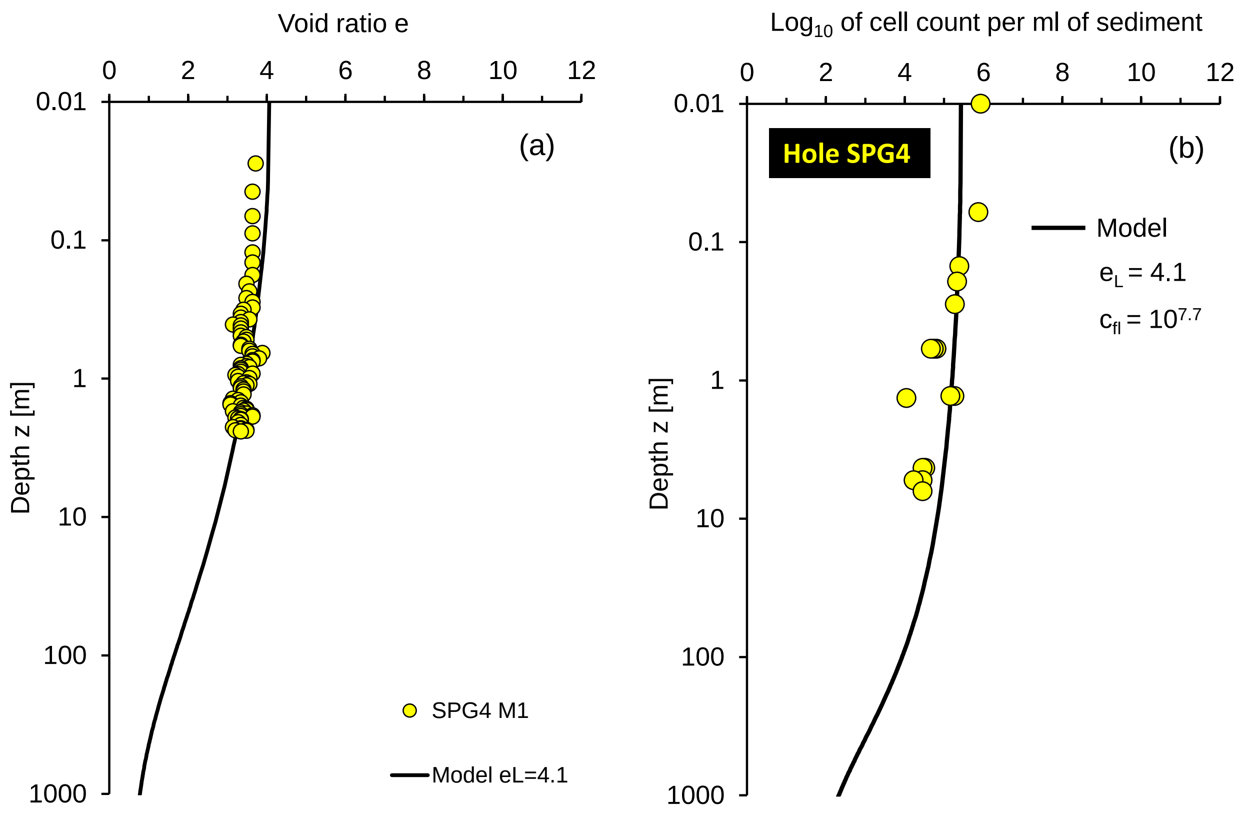

Supplementary Figure S60. South Pacific Gyre: Leg 329 - Site SPG4. Void ratio and cell count data profiles versus depth and prediction models. (A) Void ratio depth profile - Site SPG4 M1/P1 [Data extracted from (Ref. 108)] (model parameters: e_L = 4.1). (B) Cell count profile - Site SPG4 [data extracted from (Ref. 108)] (the estimated cell concentration of the pore fluid c_{fl} = $10^{7.7}$ cell counts/cm^3).

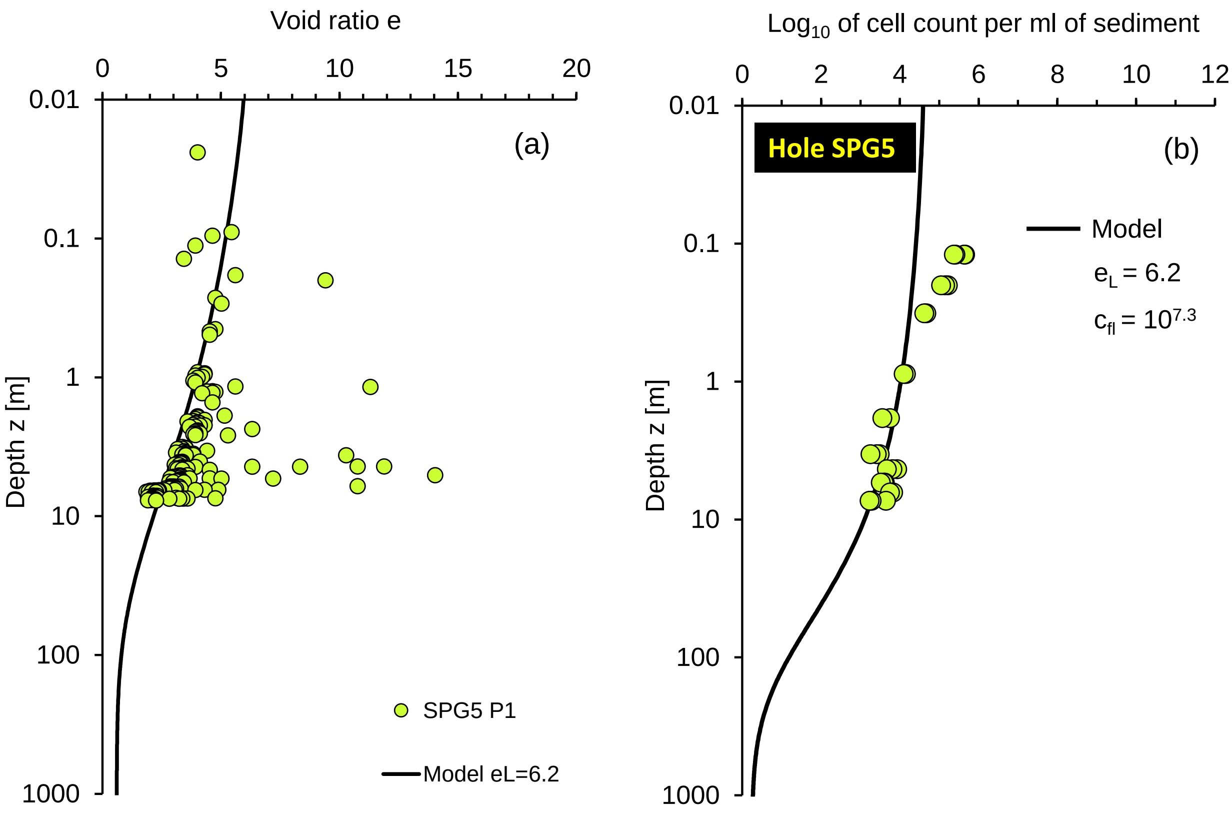

Supplementary Figure S61. South Pacific Gyre: Leg 329 - Site SPG5. Void ratio and cell count data profiles versus depth and prediction models. (A) Void ratio depth profile - Site SPG5 M1/P1 [Data extracted from (Ref. 108)] (model parameters: e_L = 6.2). (B) Cell count profile - Site SPG5 [data extracted from (Ref. 108)] (the estimated cell concentration of the pore fluid c_{fl} = $10^{7.3}$ cell counts/cm^3).

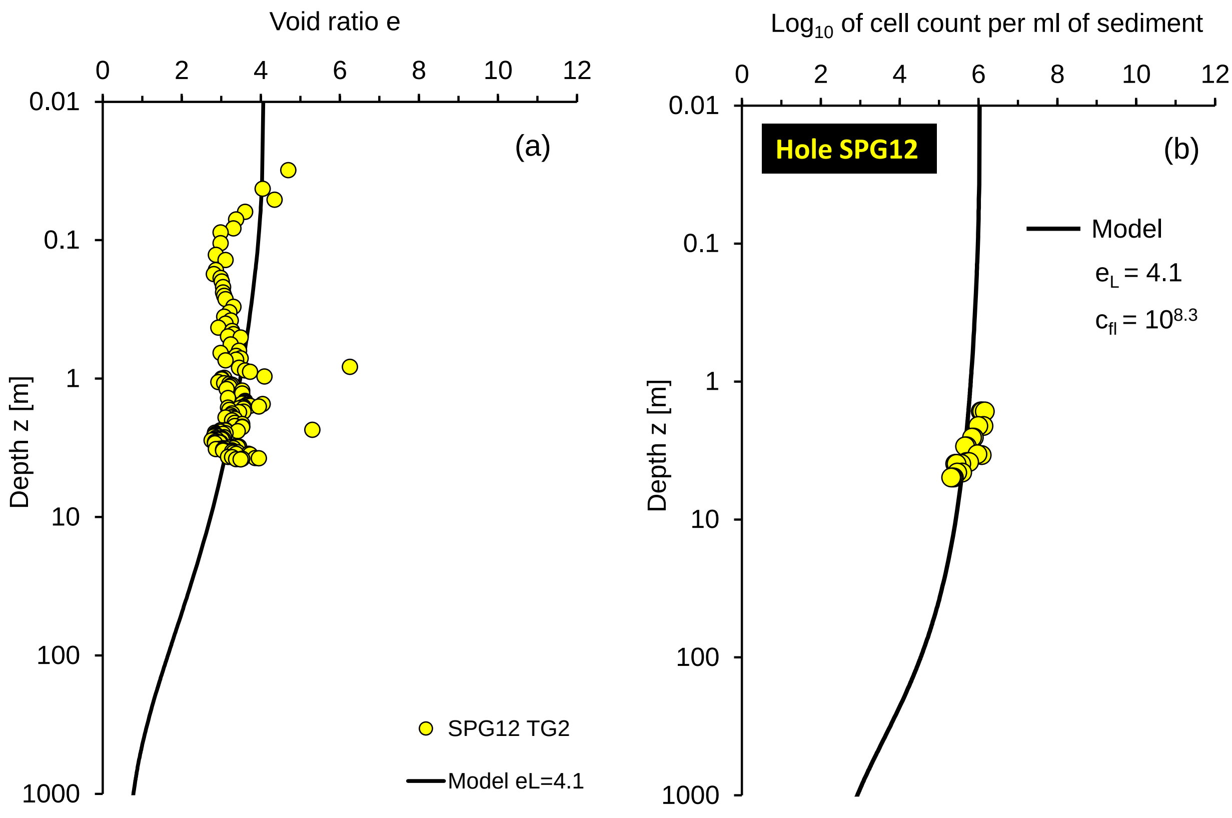

Supplementary Figure S62. South Pacific Gyre: Leg 329 - Site SPG12. Void ratio and cell count data profiles versus depth and prediction models. (A) Void ratio depth profile - Site SPG12 TG2 [Data extracted from (Ref. 108)] (model parameters: $e_L = 4.1$). (B) Cell count profile - Site SPG12 [data extracted from (Ref. 108)] (the estimated cell concentration of the pore fluid $c_{fl} = 10^{8.3}$ cell counts/cm^3).

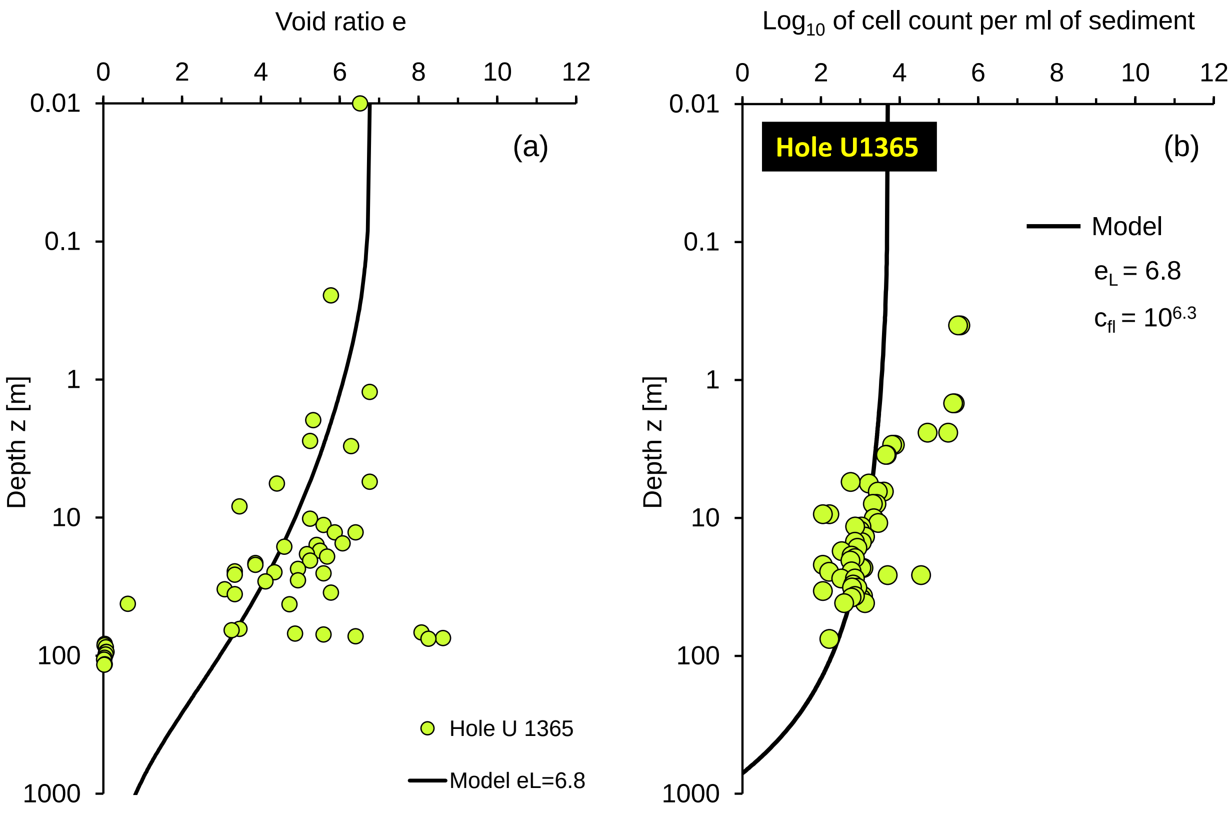

Supplementary Figure S63. South Pacific Gyre: Leg 329 - Site U1365. Void ratio and cell count data profiles versus depth and prediction models. (A) Void ratio depth profile - Site U1365 [Data extracted from (Ref. 109)] (model parameters: e_L = 6.8). (B) Cell count profile - Site U1365 [data extracted from (Ref. 109)] (the estimated cell concentration of the pore fluid c_{fl} = $10^{6.3}$ cell counts/cm^3).

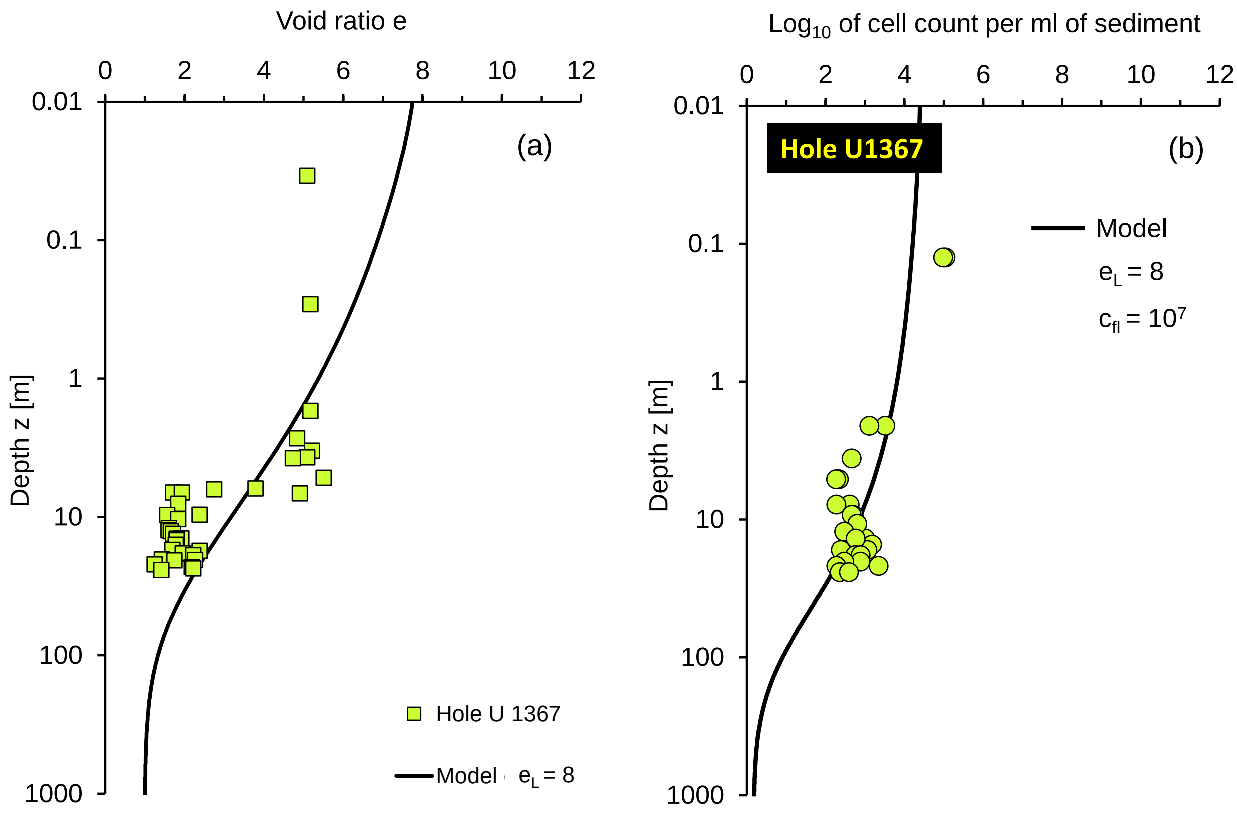

Supplementary Figure S64. South Pacific Gyre: Leg 329 - Site U1367. Void ratio and cell count data profiles versus depth and prediction models. (A) Void ratio depth profile - Site U1367 [Data extracted from (Ref. 111)] (model parameters: $e_L = 8$). (B) Cell count profile - Site U1367 [data extracted from (Ref. 111)] (the estimated cell concentration of the pore fluid $c_{fl} = 10^7$ cell counts/cm^3).

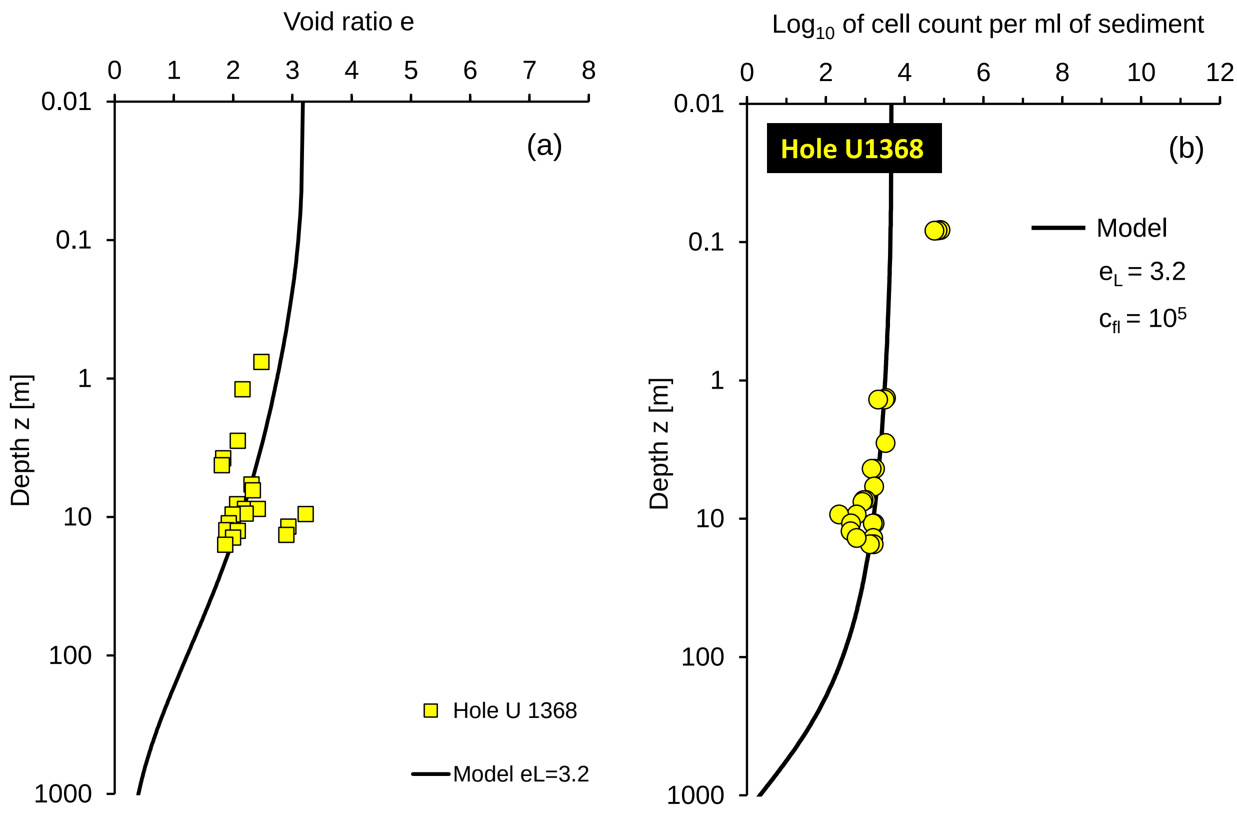

Supplementary Figure S65. South Pacific Gyre: Leg 329 - Site U1368. Void ratio and cell count data profiles versus depth and prediction models. (A) Void ratio depth profile - Site U1368 [Data extracted from (Ref. 112)] (model parameters: e_L = 3.2). (B) Cell count profile - Site U1368 [data extracted from (Ref. 112)] (the estimated cell concentration of the pore fluid c_{fl} = 10^5 cell counts/cm^3).

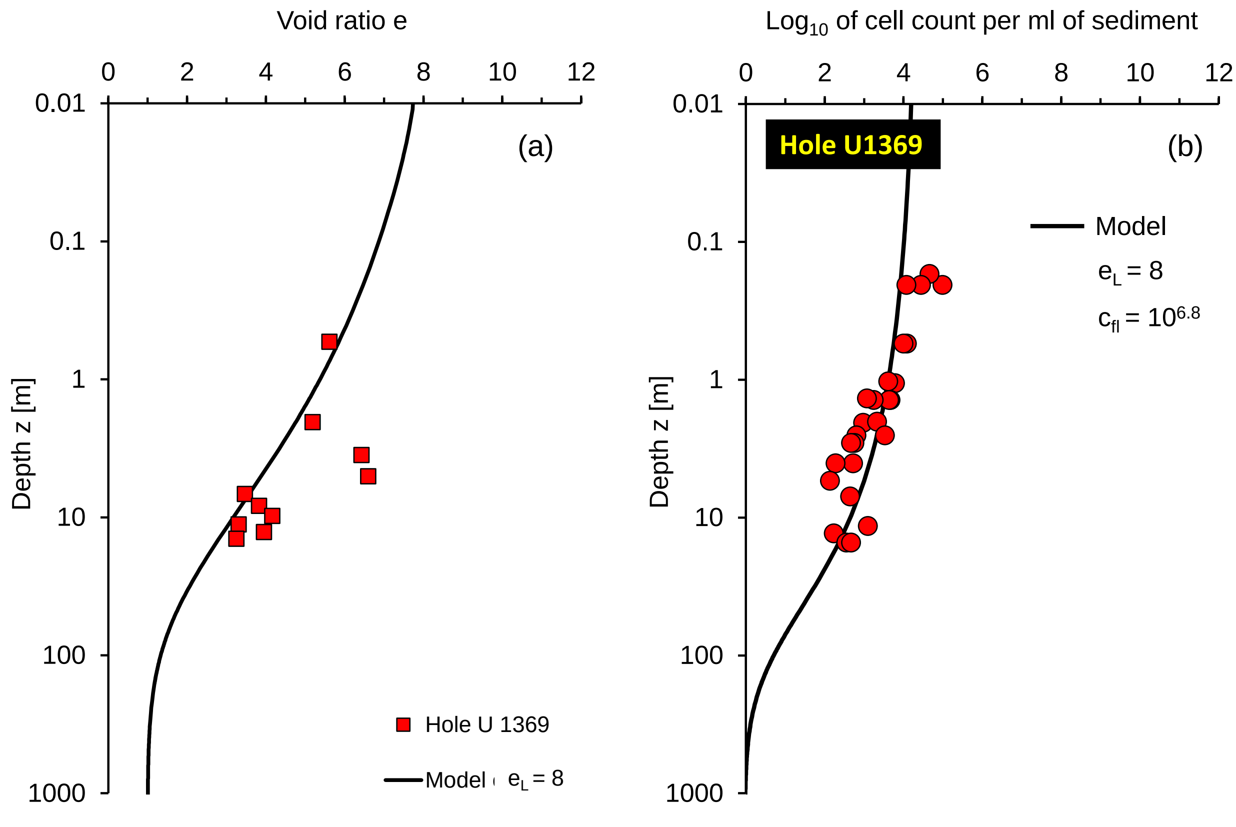

Supplementary Figure S66. South Pacific Gyre: Leg 329 - Site U1369. Void ratio and cell count data profiles versus depth and prediction models. (A) Void ratio depth profile - Site U1369 [Data extracted from (Ref. 113)] (model parameters: $e_L = 8$). (B) Cell count profile - Site U1369 [data extracted from (Ref. 113)] (the estimated cell concentration of the pore fluid $c_{fl} = 10^{6.8}$ cell counts/cm^3).

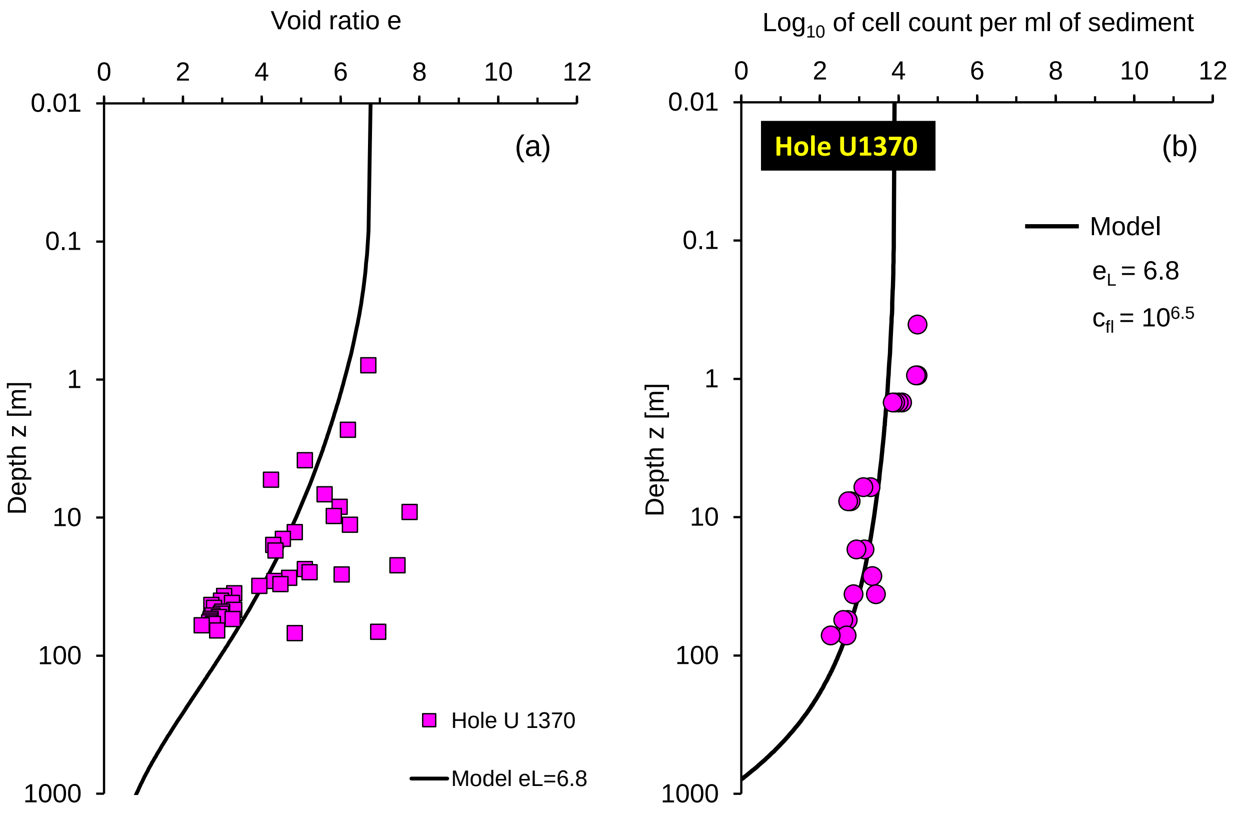

Supplementary Figure S67. South Pacific Gyre: Leg 329 - Site U1370. Void ratio and cell count data profiles versus depth and prediction models. (A) Void ratio depth profile - Site U1370 [Data extracted from (Ref. 114)] (model parameters: $e_L = 6.8$). (B) Cell count profile - Site U1370 [data extracted from (Ref. 114)] (the estimated cell concentration of the pore fluid $c_{fl} = 10^{6.5}$ cell counts/cm^3).

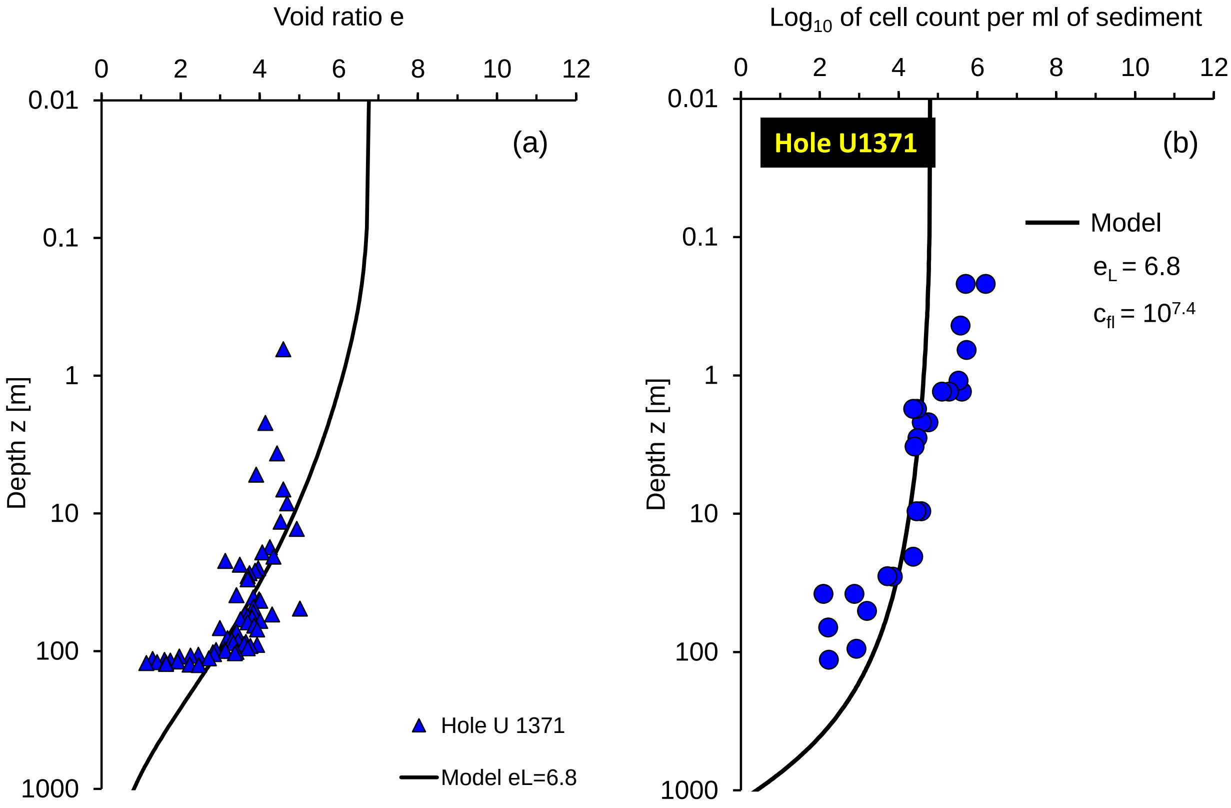

Supplementary Figure S68. South Pacific Gyre: Leg 329 - Site U1371. Void ratio and cell count data profiles versus depth and prediction models. (A) Void ratio depth profile - Site U1371 [Data extracted from (Ref. 115)] (model parameters: $e_L = 6.8$). (B) Cell count profile - Site U1371 [data extracted from (Ref. 115)] (the estimated cell concentration of the pore fluid $c_{fl} = 10^{7.4}$ cell counts/cm^3).

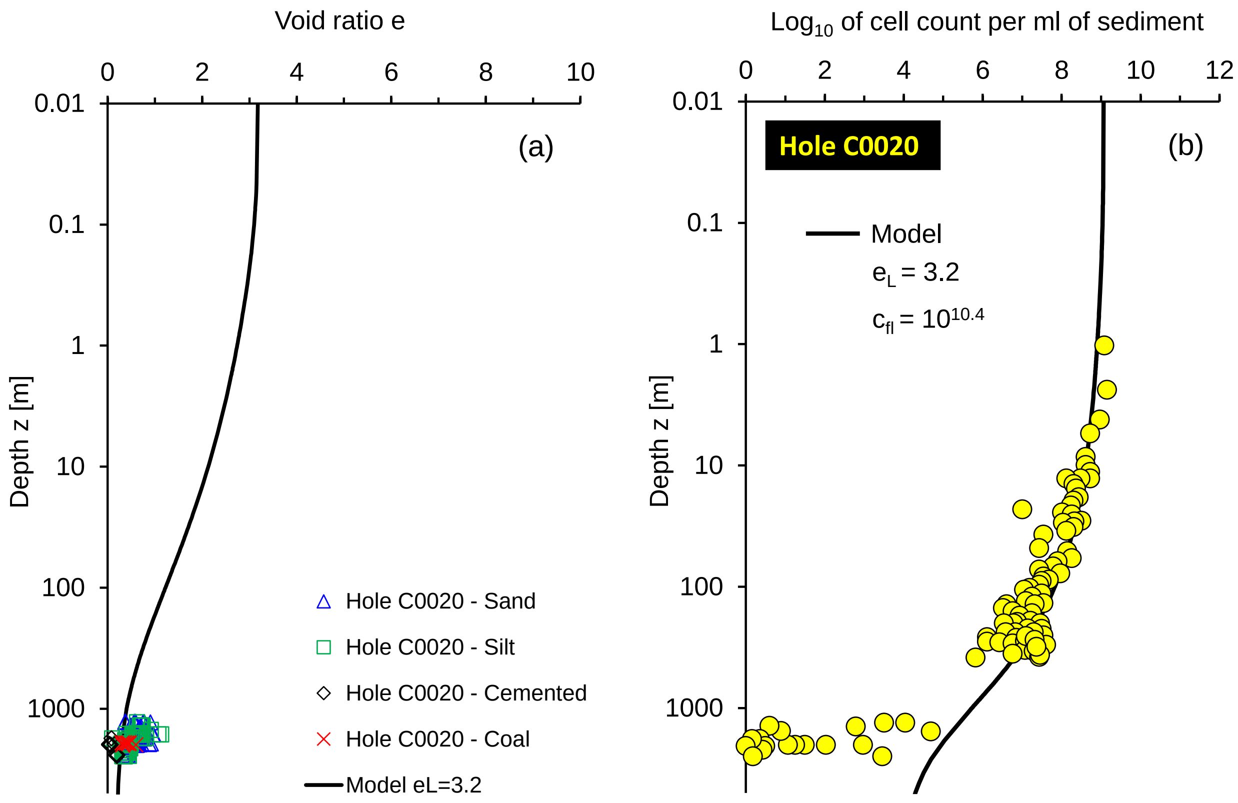

Supplementary Figure S69. Shimokita: Leg 337 - Site C0020. Void ratio and cell count data profiles versus depth and prediction models. (A) Void ratio depth profile - Site C0020 [Data extracted from (Ref. 124)] (model parameters: e_L = 3.2). (B) Cell count profile - Site C0020 [data extracted from (Ref. 124)] (the estimated cell concentration of the pore fluid c_{fl} = $10^{10.4}$ cell counts/cm^3).

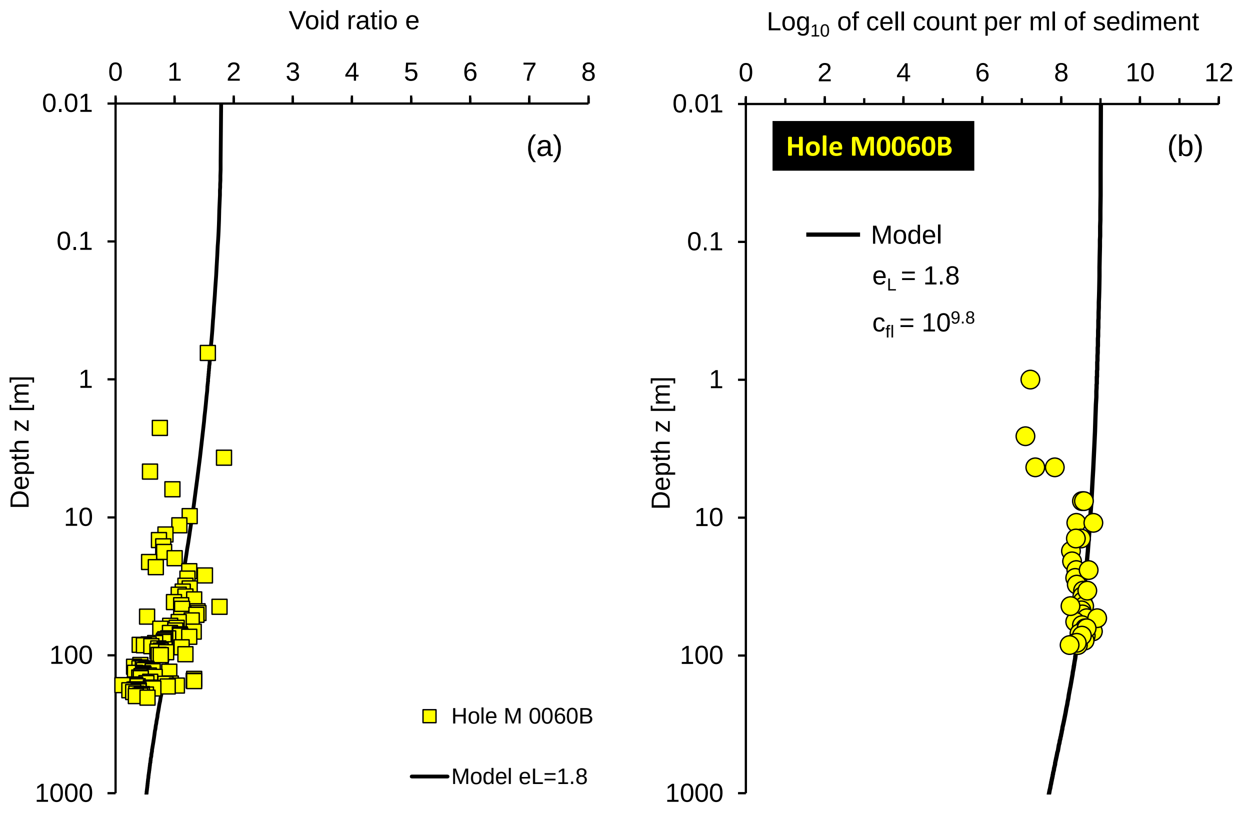

Supplementary Figure S70. Baltic Sea Basin: Leg 347 - Site M0060B. Void ratio and cell count data profiles versus depth and prediction models. (A) Void ratio depth profile - M0060B [Data extracted from (Ref. 126)] (model parameters: e_L = 1.8). (B) Cell count profile - Site M0060B [data extracted from (Ref. 126)] (the estimated cell concentration of the pore fluid $c_{fl} = 10^{9.8}$ cell counts/cm^3).

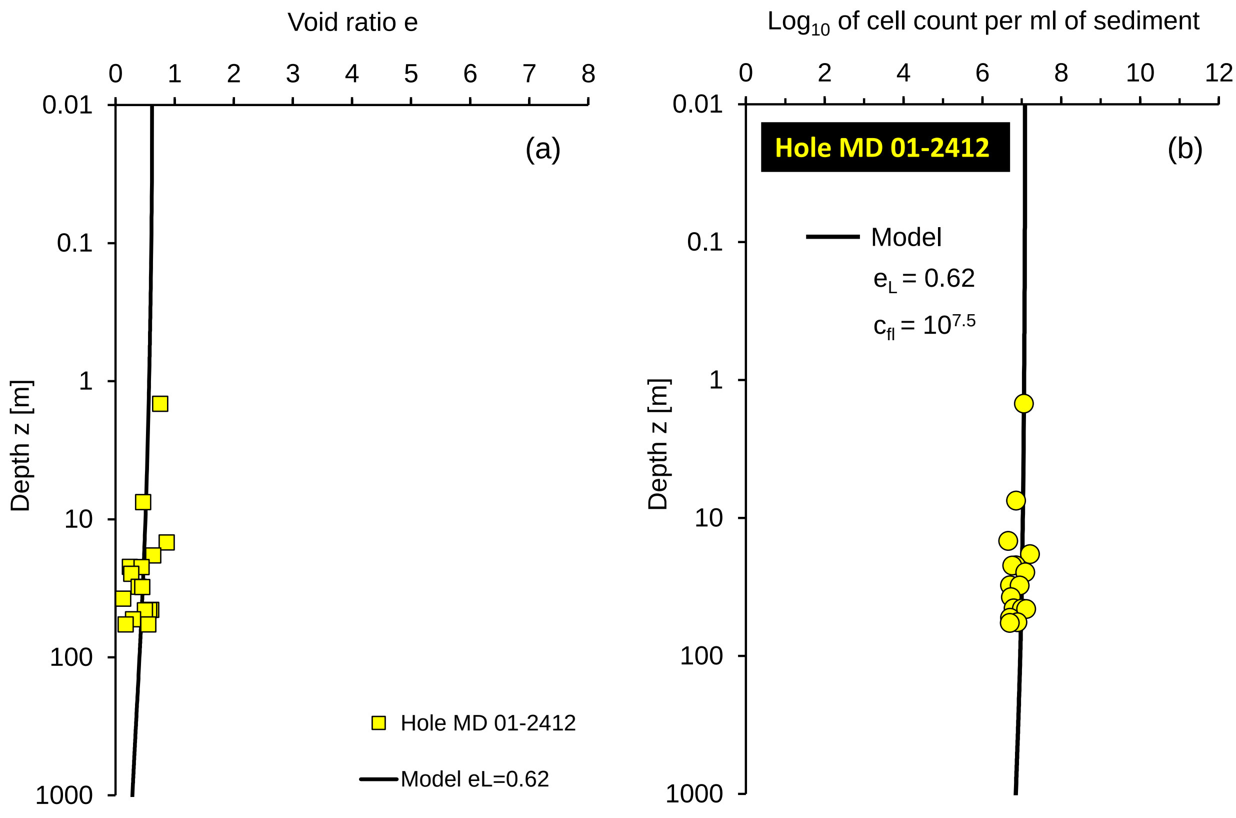

Supplementary Figure S71. Sea of Okhotsk - Site MD 01-2412. Void ratio and cell count data profiles versus depth and prediction models. (A) Void ratio depth profile - Site MD 01-2412 [data extracted from (Ref. 130)] (model parameters: e_L = 0.62). (B) Cell count profile - Site MD 01-2412 [data extracted from (Ref. 130)] (the estimated cell concentration of the pore fluid $c_{fl} = 10^{7.5}$ cell counts/cm^3).

VOID RATIO AND CELL COUNT PROFILES

Data with high variability

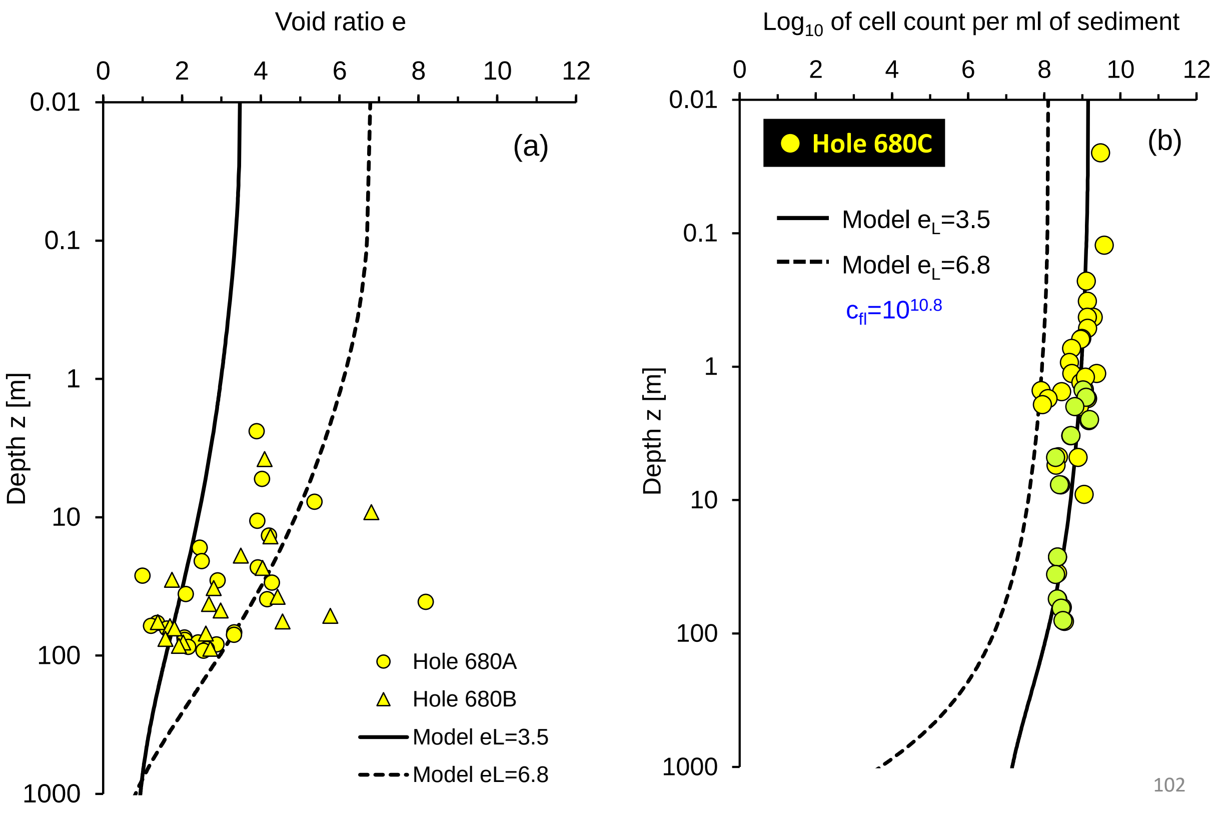

Supplementary Figure S72. Deep Peru Margin: Leg 112 - Site 680C. Void ratio and cell count data profiles versus depth and prediction models. (A) Void ratio depth profile - Site 680A/B. [Data extracted from (Ref. 25)] (model parameters: $e_L = 3.5$). (B) Cell count profile - Site 680C [data extracted from (Ref. 26)] (the estimated cell concentration of the pore fluid $c_{fl} = 10^{10.8}$ cell counts/cm^3).

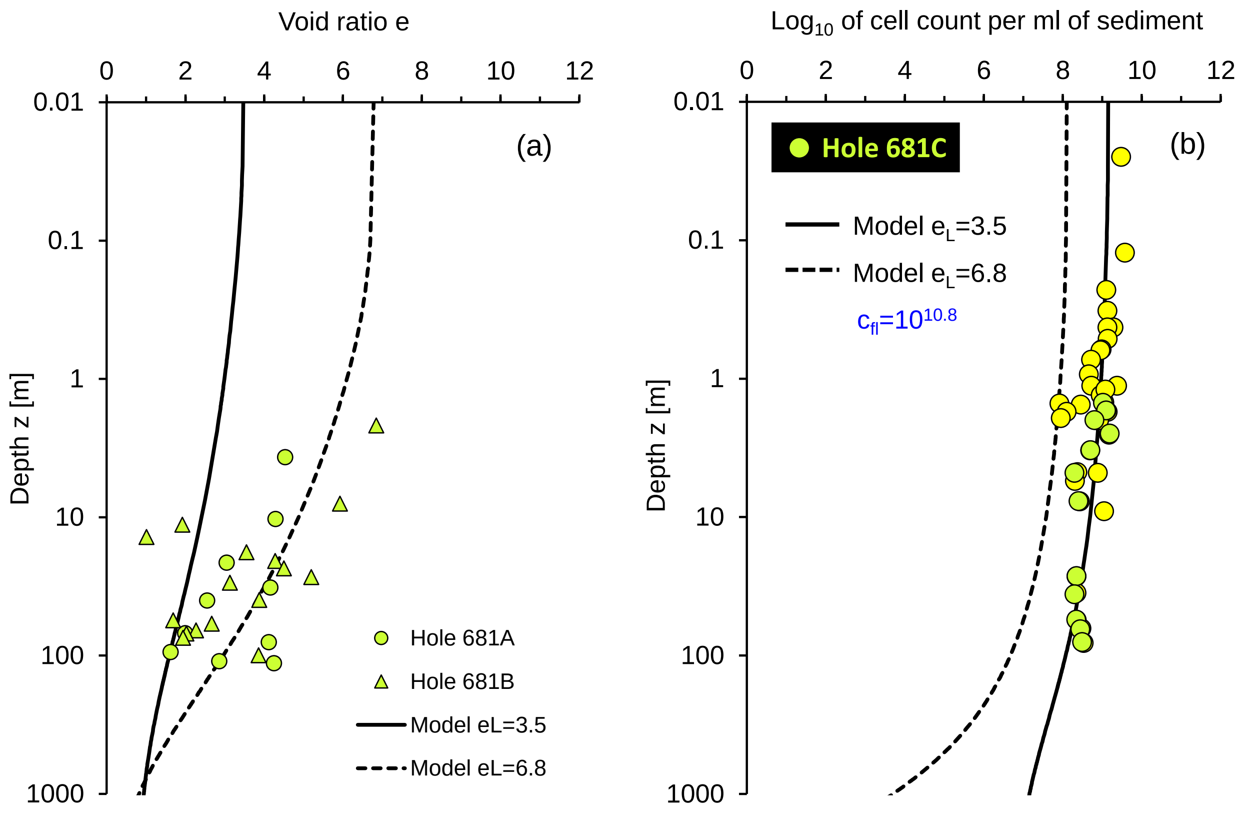

Supplementary Figure S73. Deep Peru Margin: Leg 112 - Site 681C. Void ratio and cell count data profiles versus depth and prediction models. (A) Void ratio depth profile - Site 681A/B. [Data extracted from (Ref. 27)] (model parameters: e_L = 3.5). (B) Cell count profile - Site 681C [data extracted from (Ref. 26)] (the estimated cell concentration of the pore fluid c_{fl} = $10^{10.8}$ cell counts/cm^3).

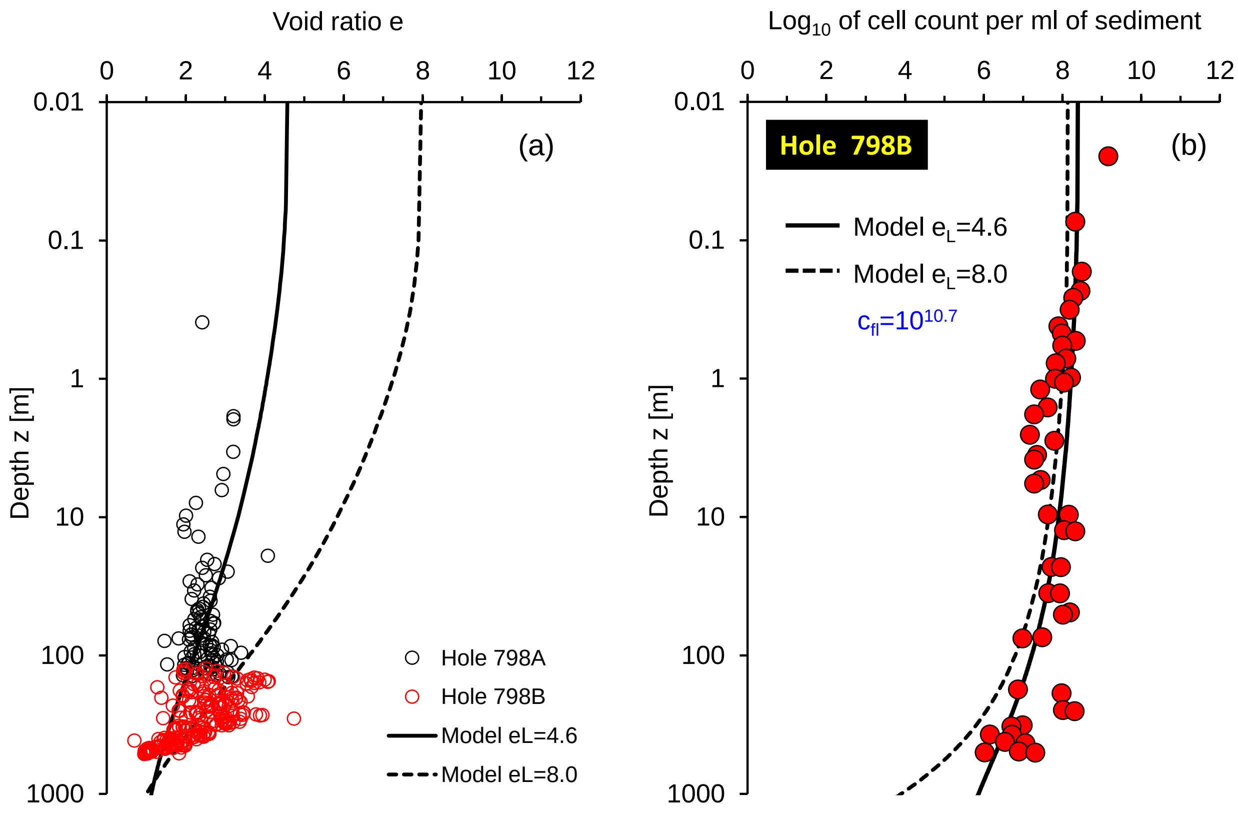

Supplementary Figure S74. Japan Sea: Leg 128 - Site 798B. Void ratio and cell count data profiles versus depth and prediction models. (A) Void ratio depth profile - Site 798A/B [Data extracted from (Ref. 28)] (model parameters: e_L = 4.6). (B) Cell count profile - Site 798B [data extracted from (Ref. 29)] (the estimated cell concentration of the pore fluid c_{fl} = $10^{10.7}$ cell counts/cm^3).

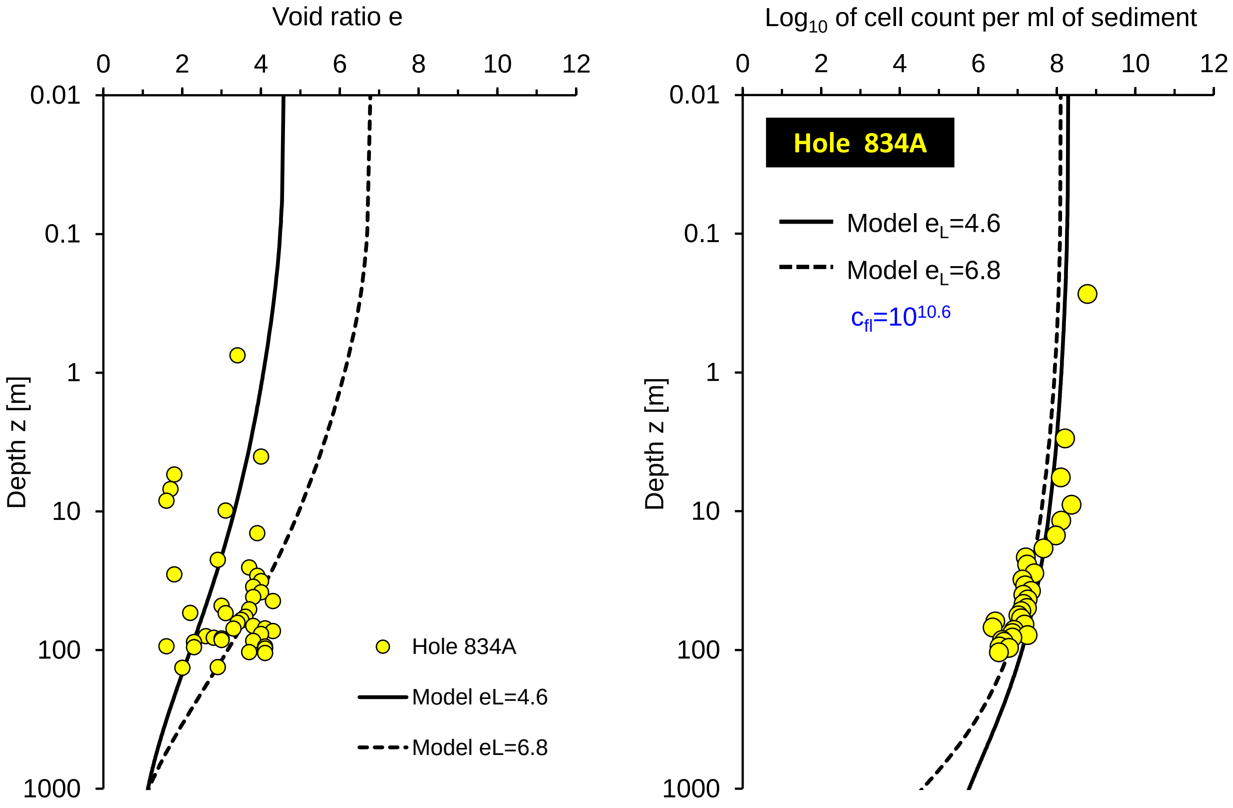

Supplementary Figure S75. Lau Basin: Leg 135 - Site 834A. Void ratio and cell count data profiles versus depth and prediction models. (A) Void ratio depth profile - Site 864A [Data extracted from (Ref. 30)] (model parameters: e_L = 4.6). (B) Cell count profile - Site 834A [data extracted from (Ref. 31)] (the estimated cell concentration of the pore fluid c_{fl} = $10^{10.6}$ cell counts/cm^3).

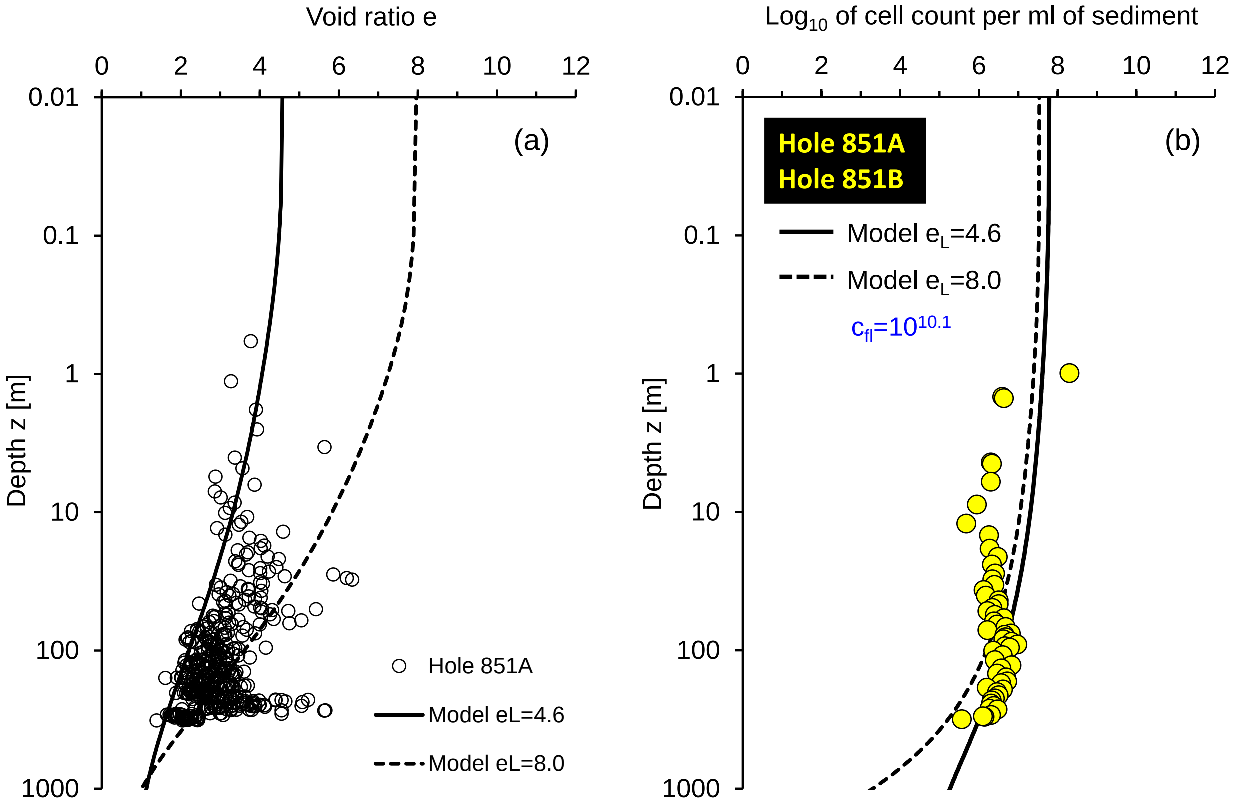

Supplementary Figure S76. Eastern Equatorial Pacific: Leg 138 - Site 851A. Void ratio and cell count data profiles versus depth and prediction models. (A) Void ratio depth profile - Site 851A [Data extracted from (Ref. 32)] (model parameters: e_L = 4.6). (B) Cell count profile - Site 851A [data extracted from (Ref. 33)] (the estimated cell concentration of the pore fluid $c_{fl} = 10^{10.1}$ cell counts/cm^3).

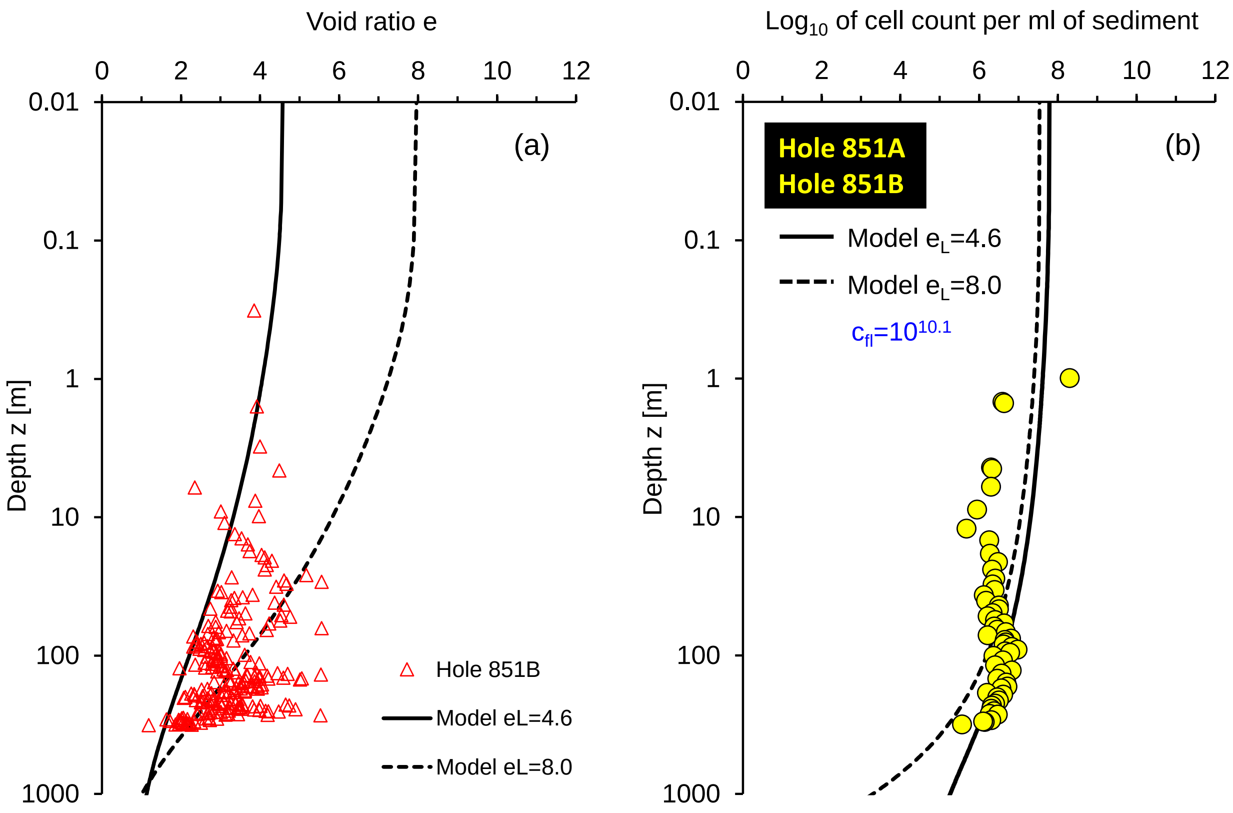

Supplementary Figure S77. Eastern Equatorial Pacific: Leg 138 - Site 851B. Void ratio and cell count data profiles versus depth and prediction models. (A) Void ratio depth profile - Site 851B [Data extracted from (Ref. 32)] (model parameters: e_L = 4.6). (B) Cell count profile - Site 851B [data extracted from (Ref. 33)] (the estimated cell concentration of the pore fluid c_{fl} = $10^{10.1}$ cell counts/cm^3).

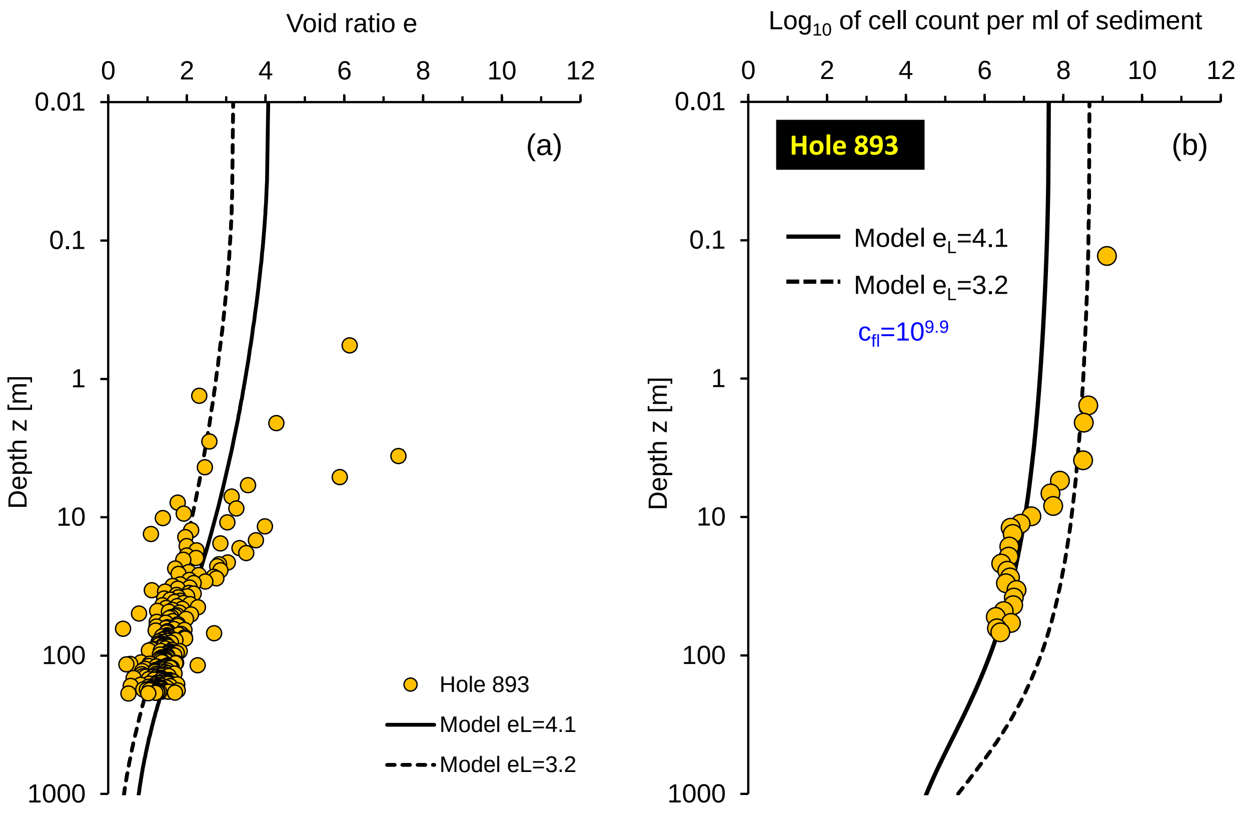

Supplementary Figure S78. Santa Barbara basin: Leg 146 - Part 2: Site 893. Void ratio and cell count data profiles versus depth and prediction models. (A) Void ratio depth profile - Site 893 [Data extracted from (Ref. 42)] (model parameters: $e_L = 4.1$). (B) Cell count profile - Site 893 [data extracted from (Ref. 43)] (the estimated cell concentration of the pore fluid $c_{fl} = 10^{9.9}$ cell counts/cm^3).

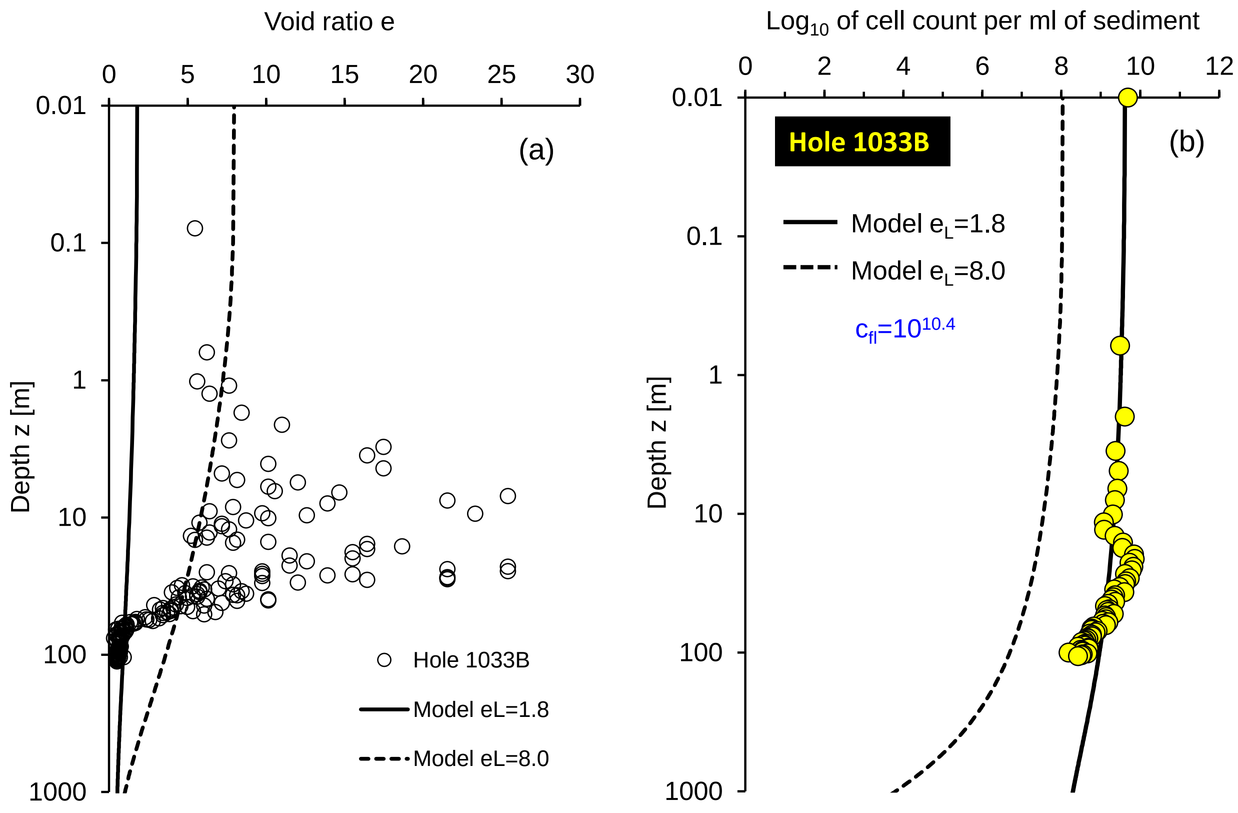

Supplementary Figure S79. Saanich Inlet, BC: Leg 169(S) - Site 1033B. Void ratio and cell count data profiles versus depth and prediction models. (A) Void ratio depth profile - Site 1033B [Data extracted from (Ref. 62)] (model parameters: e_L = 1.8). (B) Cell count profile - Site 1033B [data extracted from (Ref. 63)] (the estimated cell concentration of the pore fluid c_{fl} = $10^{10.4}$ cell counts/cm^3).

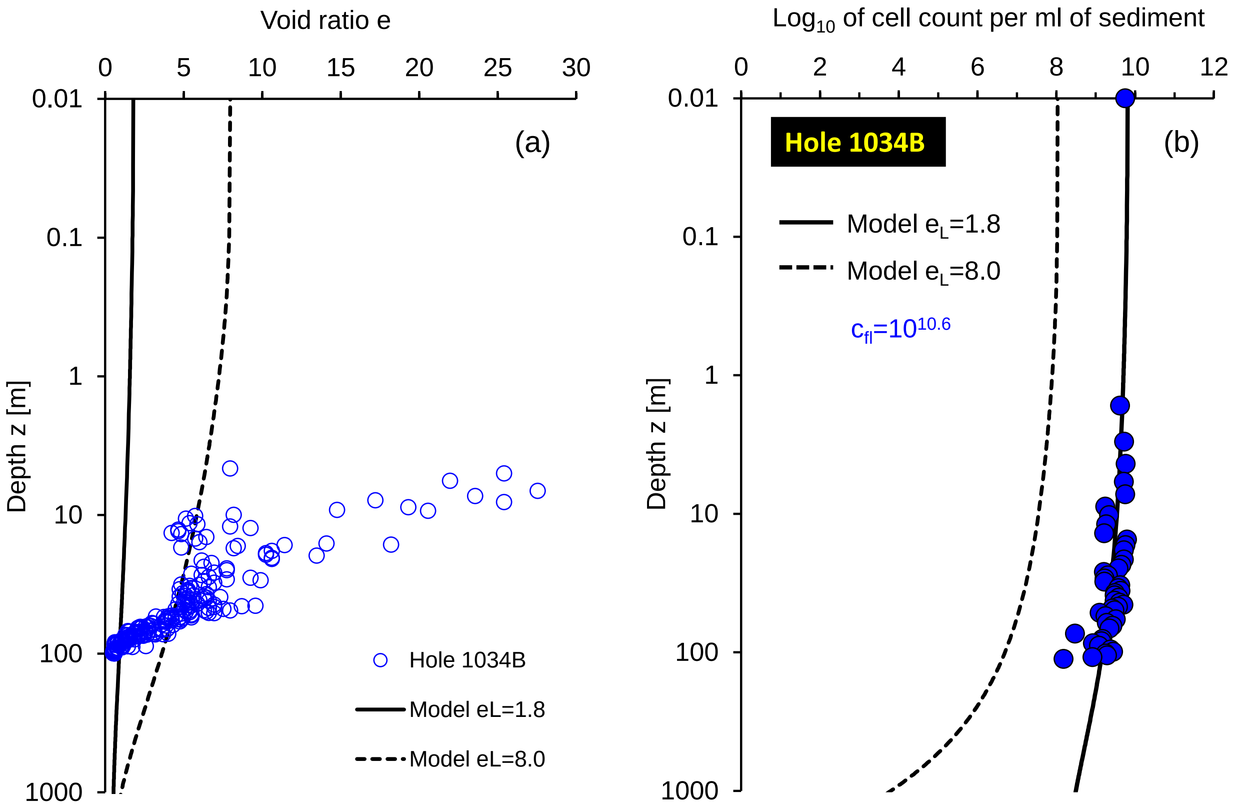

Supplementary Figure S80. Saanich Inlet, BC: Leg 169(S) - Site 1034B. Void ratio and cell count data profiles versus depth and prediction models. (A) Void ratio depth profile - Site 1034B [Data extracted from (Ref. 62)] (model parameters: e_L = 1.8). (B) Cell count profile - Site 1034B [data extracted from (Ref. 63)] (the estimated cell concentration of the pore fluid c_{fl} = $10^{10.6}$ cell counts/cm^3).

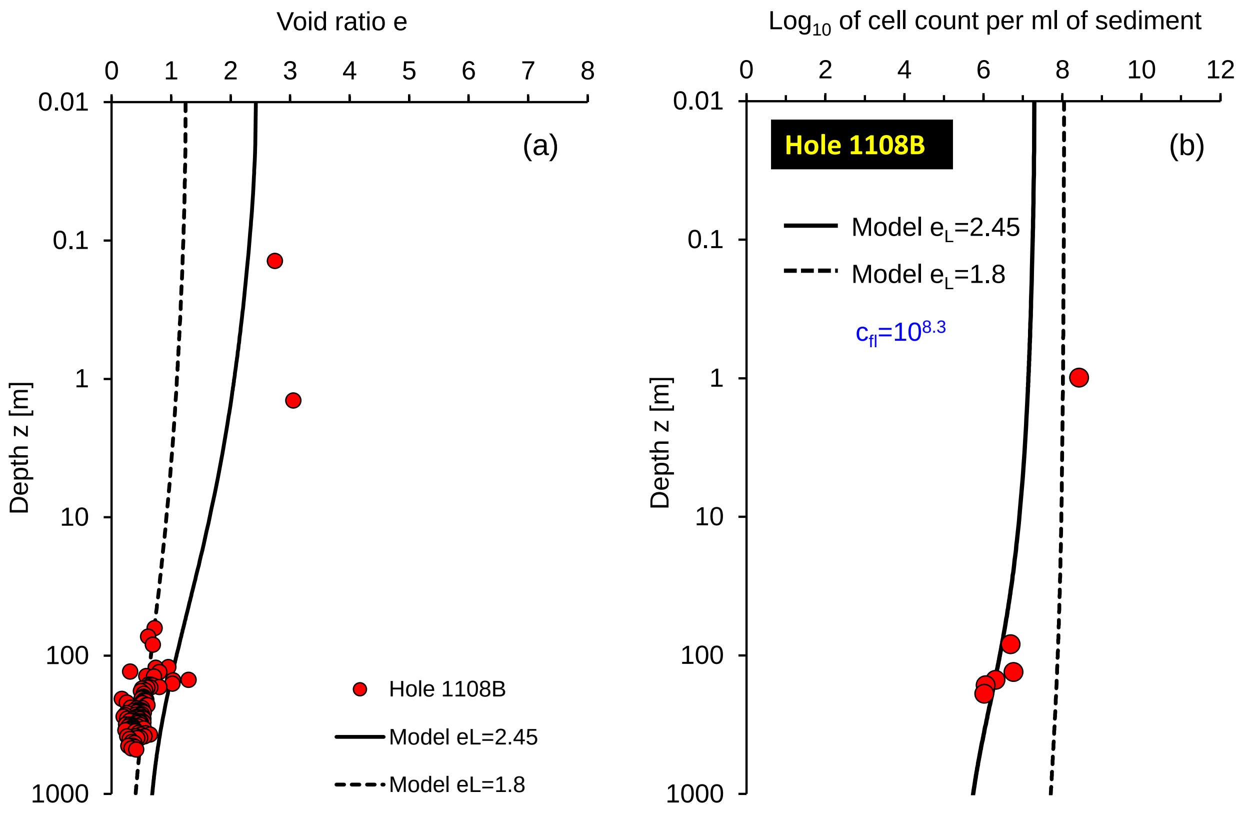

Supplementary Figure S81. Woodlark Basin: Leg 180 - Site 1108B. Void ratio and cell count data profiles versus depth and prediction models. (A) Void ratio depth profile - Site 1108B [Data extracted from (Ref. 67)] (model parameters: e_L = 2.45). (B) Cell count profile - Site 1108B [data extracted from (Ref. 67)] (the estimated cell concentration of the pore fluid c_{fl} = $10^{8.3}$ cell counts/cm^3).

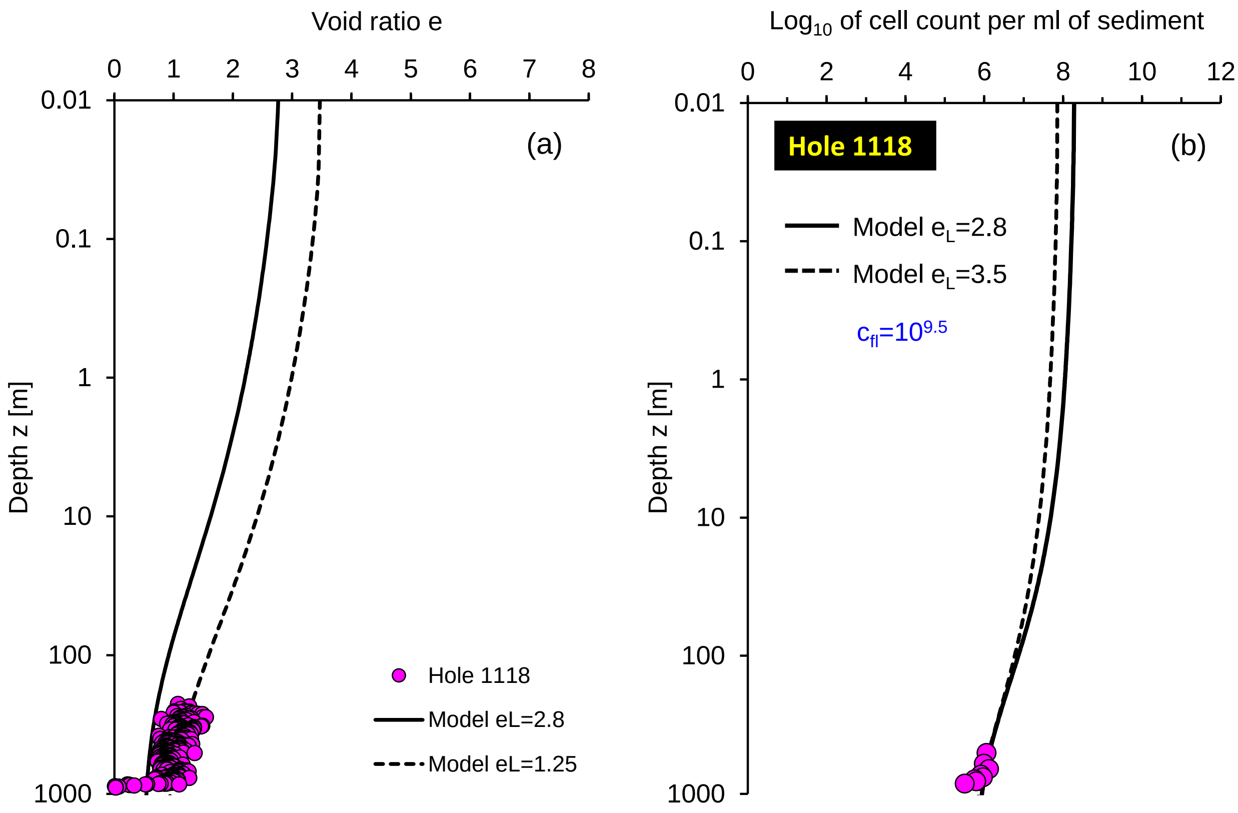

Supplementary Figure S82. Woodlark Basin: Leg 180 - Site 1118. Void ratio and cell count data profiles versus depth and prediction models. (A) Void ratio depth profile - Site 1118 [Data extracted from (Ref. 71)] (model parameters: e_L = 2.8). (B) Cell count profile - Site 1118 [data extracted from (Ref. 71)] (the estimated cell concentration of the pore fluid c_{fl} = $10^{9.5}$ cell counts/cm^3).

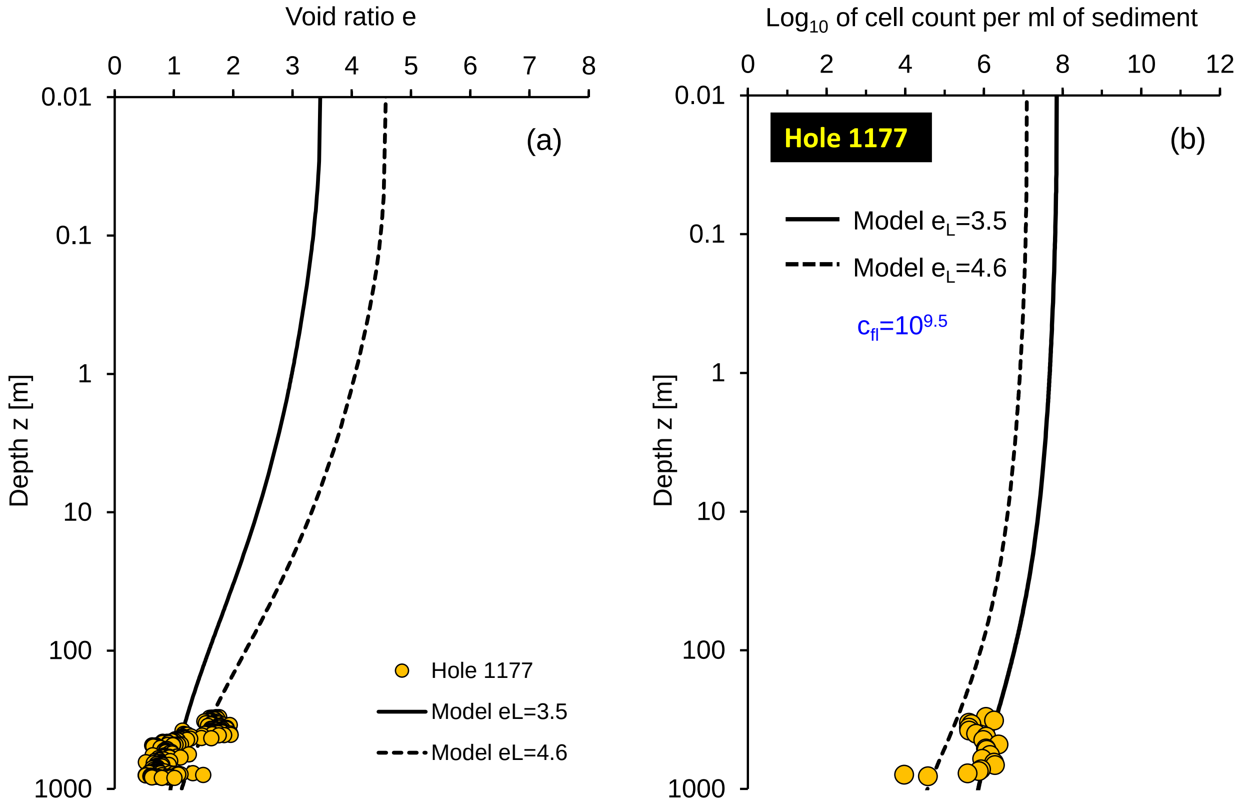

Supplementary Figure S83. Nakai Trough: Leg 190 - Site 1177. Void ratio and cell count data profiles versus depth and prediction models. (A) Void ratio depth profile - Site 1177 [Data extracted from (Ref. 76)] (model parameters: e_L = 3.5). (B) Cell count profile - Site 1177 [data extracted from (Ref. 76)] (the estimated cell concentration of the pore fluid c_{fl} = $10^{9.5}$ cell counts/cm^3).

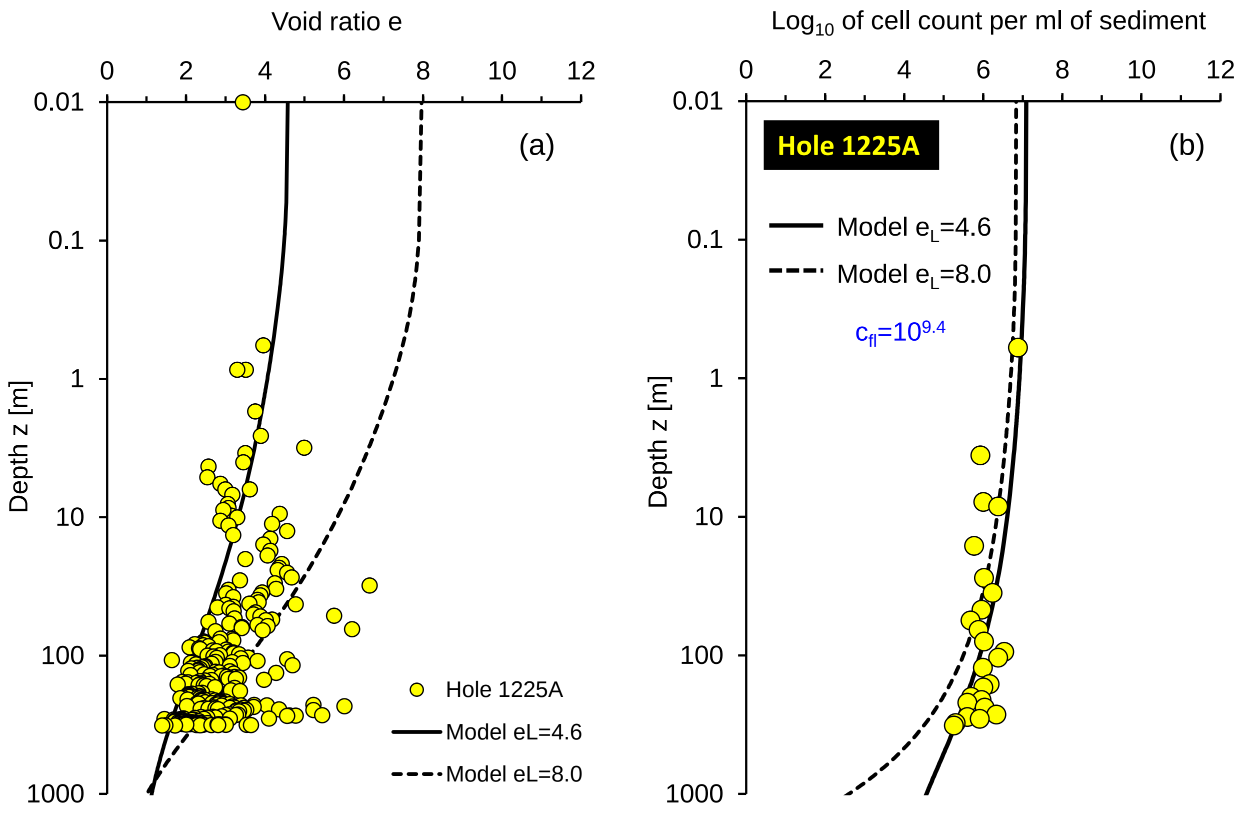

Supplementary Figure S84. Peru Margin: Leg 201 - Site 1225A. Void ratio and cell count data profiles versus depth and prediction models. (A) Void ratio depth profile - Site 1225A [Data extracted from (Ref. 78)] (model parameters: e_L = 4.6). (B) Cell count profile - Site 1225A [data extracted from (Ref. 78)] (the estimated cell concentration of the pore fluid c_{fl} = $10^{9.4}$ cell counts/cm^3).

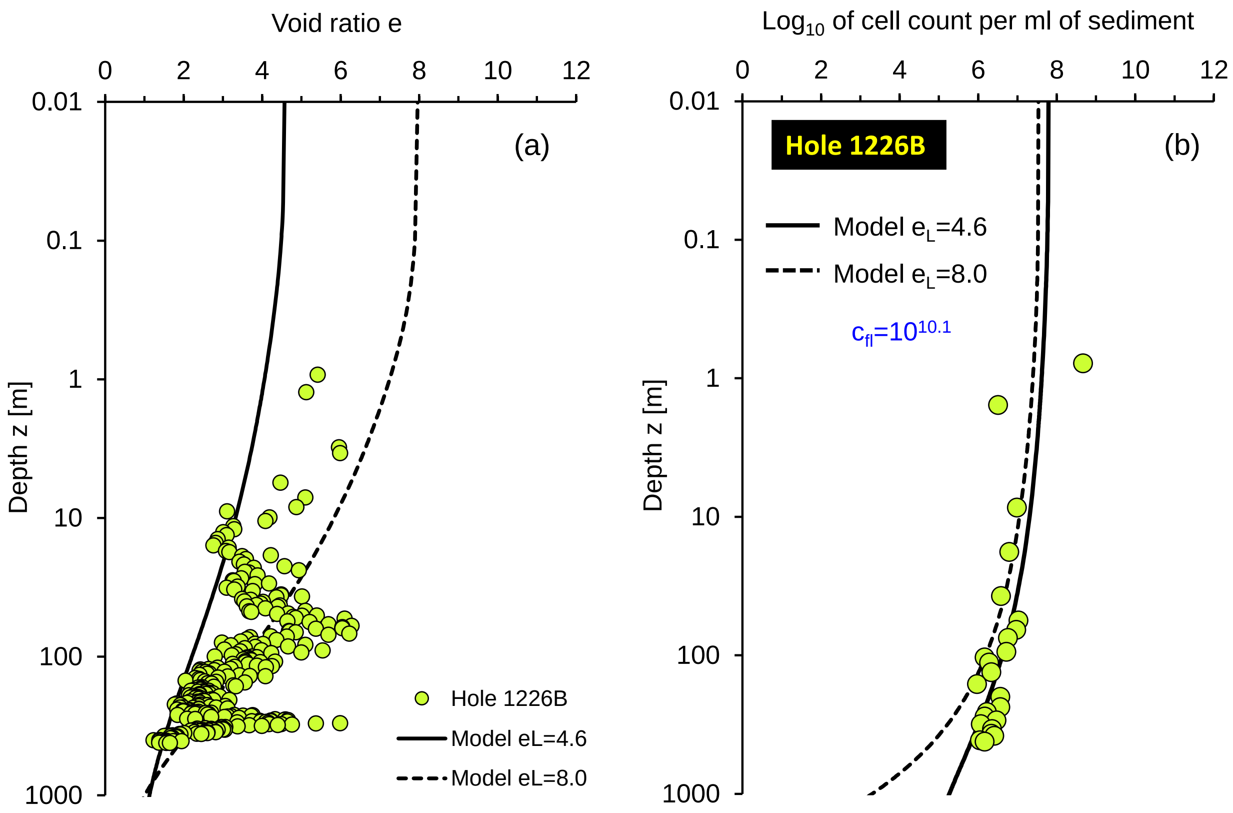

Supplementary Figure S85. Peru Margin: Leg 201 - Site 1226B. Void ratio and cell count data profiles versus depth and prediction models. (A) Void ratio depth profile - Site 1226B [Data extracted from (Ref. 79)] (model parameters: e_L = 8.0). (B) Cell count profile - Site 1226B [data extracted from (Ref. 79)] (the estimated cell concentration of the pore fluid c_{fl} = $10^{10.1}$ cell counts/cm^3).

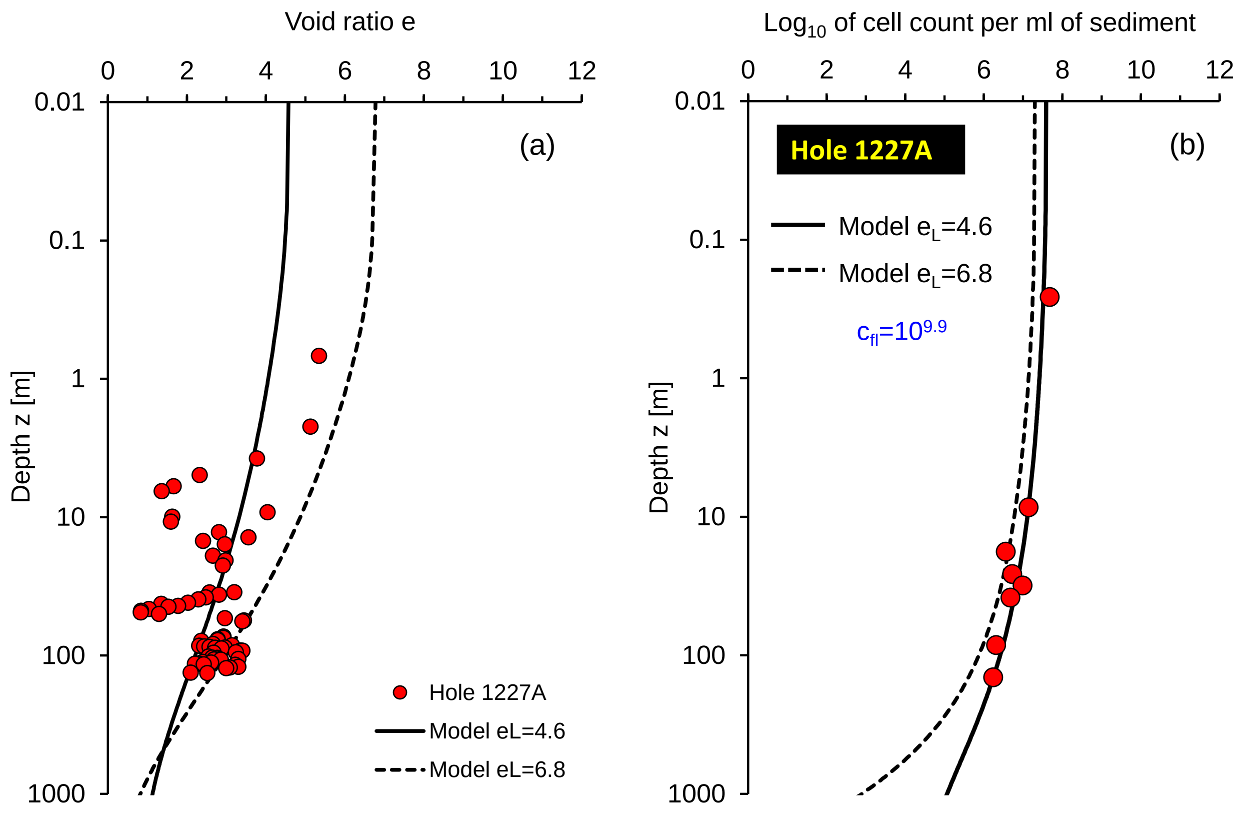

Supplementary Figure S86. Peru Margin: Leg 201 - Site 1227A. Void ratio and cell count data profiles versus depth and prediction models. (A) Void ratio depth profile - Site 1227A [Data extracted from (Ref. 80)] (model parameters: e_L = 4.6). (B) Cell count profile - Site 1227A [data extracted from (Ref. 80)] (the estimated cell concentration of the pore fluid c_{fl} = $10^{9.9}$ cell counts/cm^3).

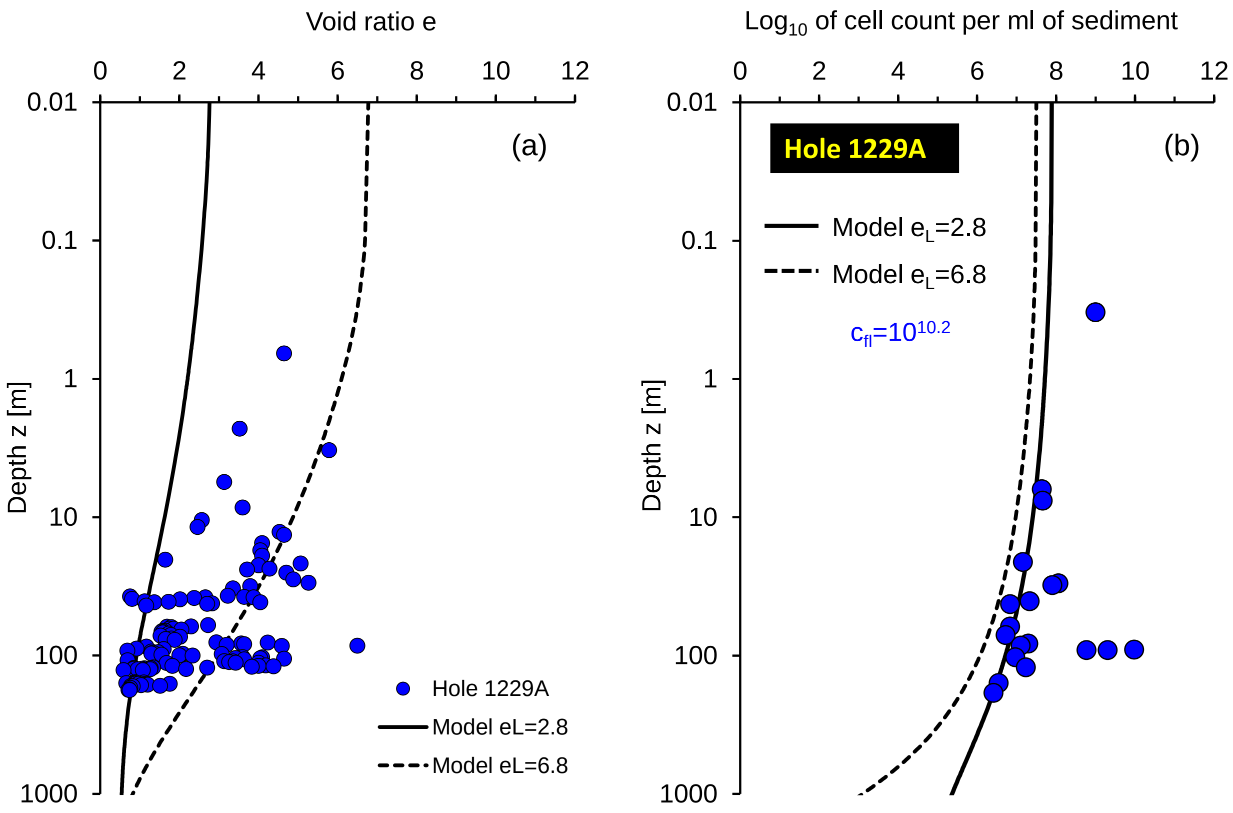

Supplementary Figure S87. Peru Margin: Leg 201 - Site 1229A. Void ratio and cell count data profiles versus depth and prediction models. (A) Void ratio depth profile - Site 1129A [Data extracted from (Ref. 81)] (model parameters: e_L = 4.6). (B) Cell count profile - Site 1129A [data extracted from (Ref. 81)] (the estimated cell concentration of the pore fluid c_{fl} = $10^{10.2}$ cell counts/cm^3).

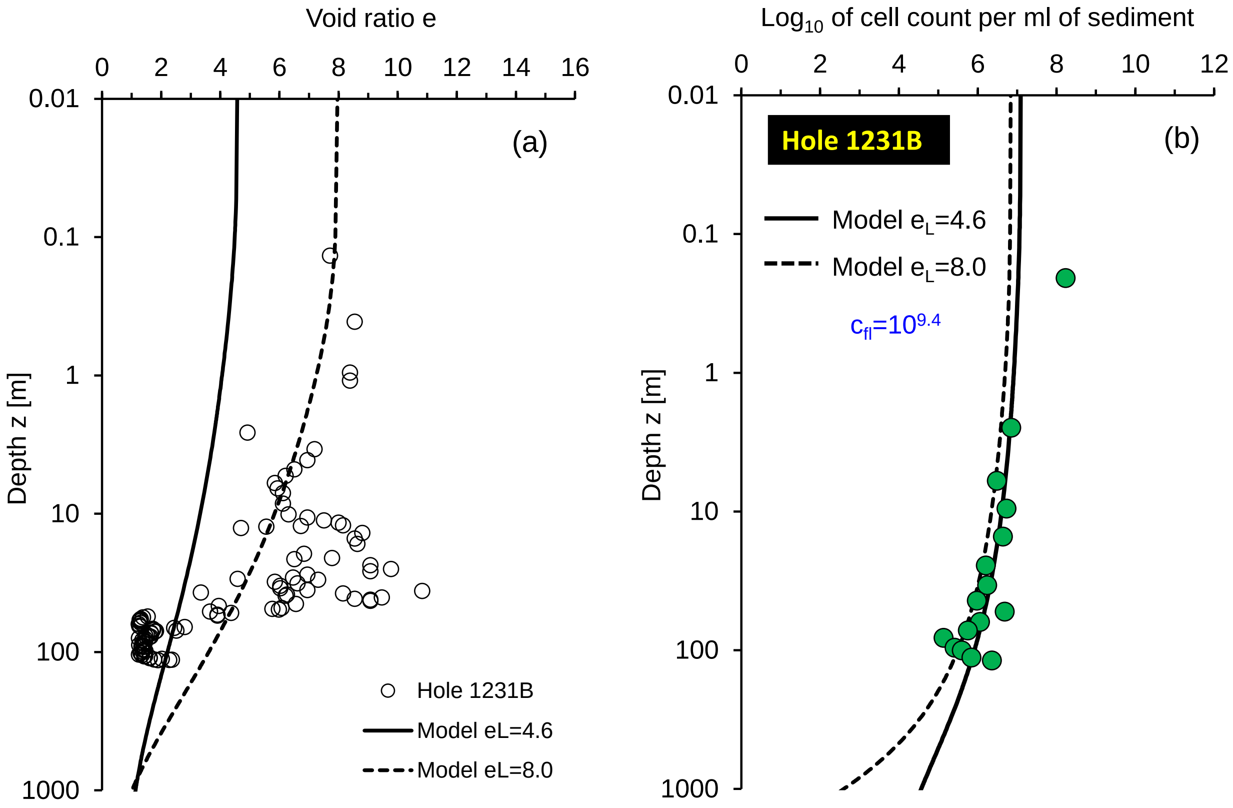

Supplementary Figure S88. Peru Margin: Leg 201 - Site 1231B. Void ratio and cell count data profiles versus depth and prediction models. (A) Void ratio depth profile - Site 1231B [Data extracted from (Ref. 83)] (model parameters: e_L = 4.6). (B) Cell count profile - Site 1231B [data extracted from (Ref. 83)] (the estimated cell concentration of the pore fluid c_{fl} = $10^{9.4}$ cell counts/cm^3).

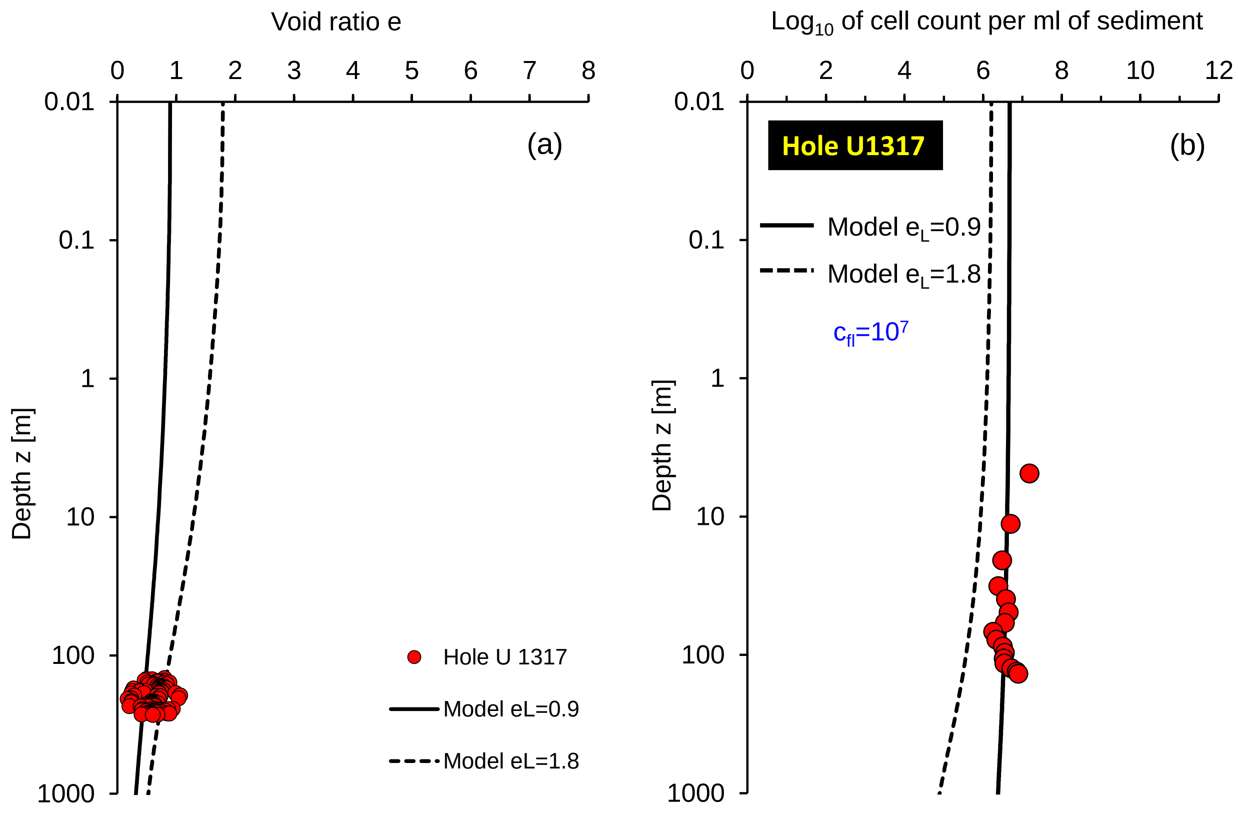

Supplementary Figure S89. Porcupine Seamount: Leg 307 - Site U1317. Void ratio and cell count data profiles versus depth and prediction models. (A) Void ratio depth profile - Site U1317 [Data extracted from (Ref. 92)] (model parameters: $e_L = 0.9$). (B) Cell count profile - Site U1317 [data extracted from (Ref. 92)] (the estimated cell concentration of the pore fluid $c_{fl} = 10^7$ cell counts/cm^3).

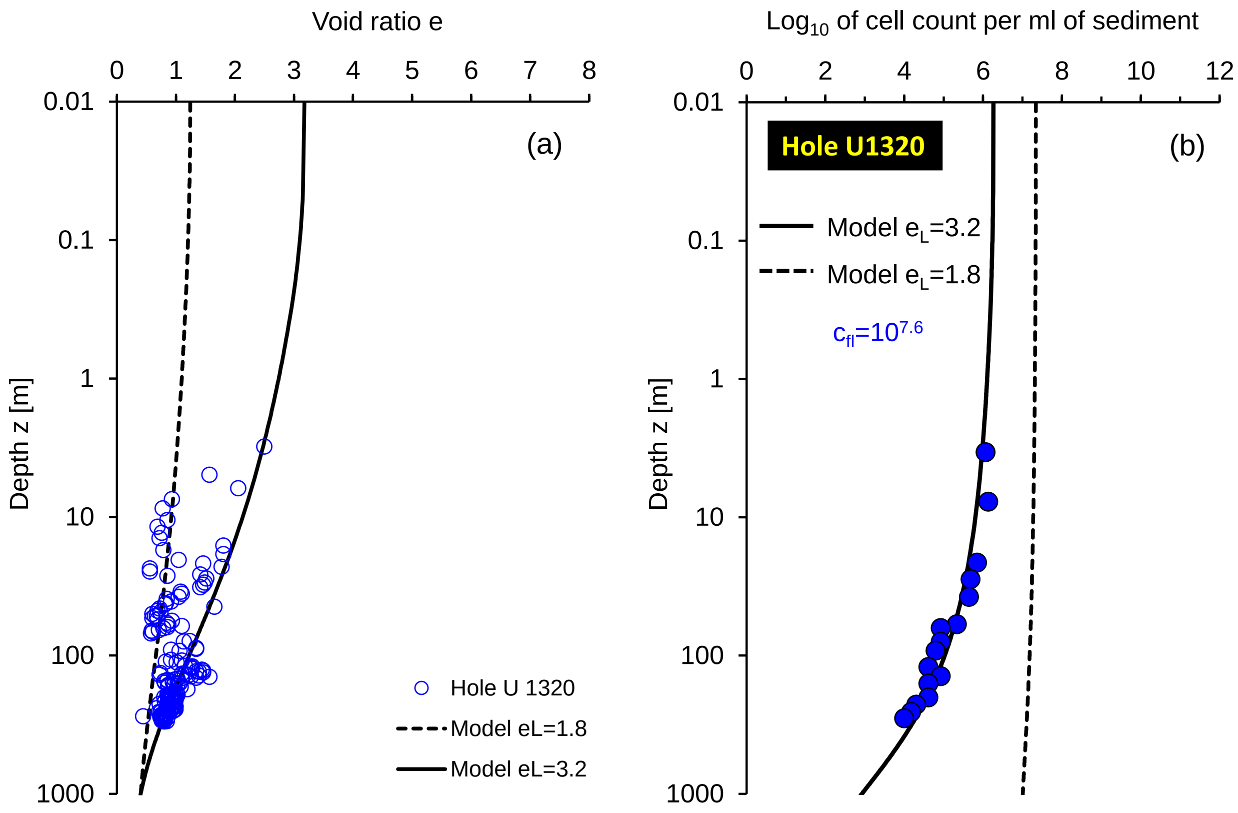

Supplementary Figure S90. Gulf of Mexico: Leg 308 - Site U1320. Void ratio and cell count data profiles versus depth and prediction models. (A) Void ratio depth profile - Site U1320 [Data extracted from (Ref. 95)] (model parameters: e_L = 3.2). (B) Cell count profile - Site U1320 [data extracted from (Ref. 95)] (the estimated cell concentration of the pore fluid c_{fl} = $10^{7.6}$ cell counts/cm^3).

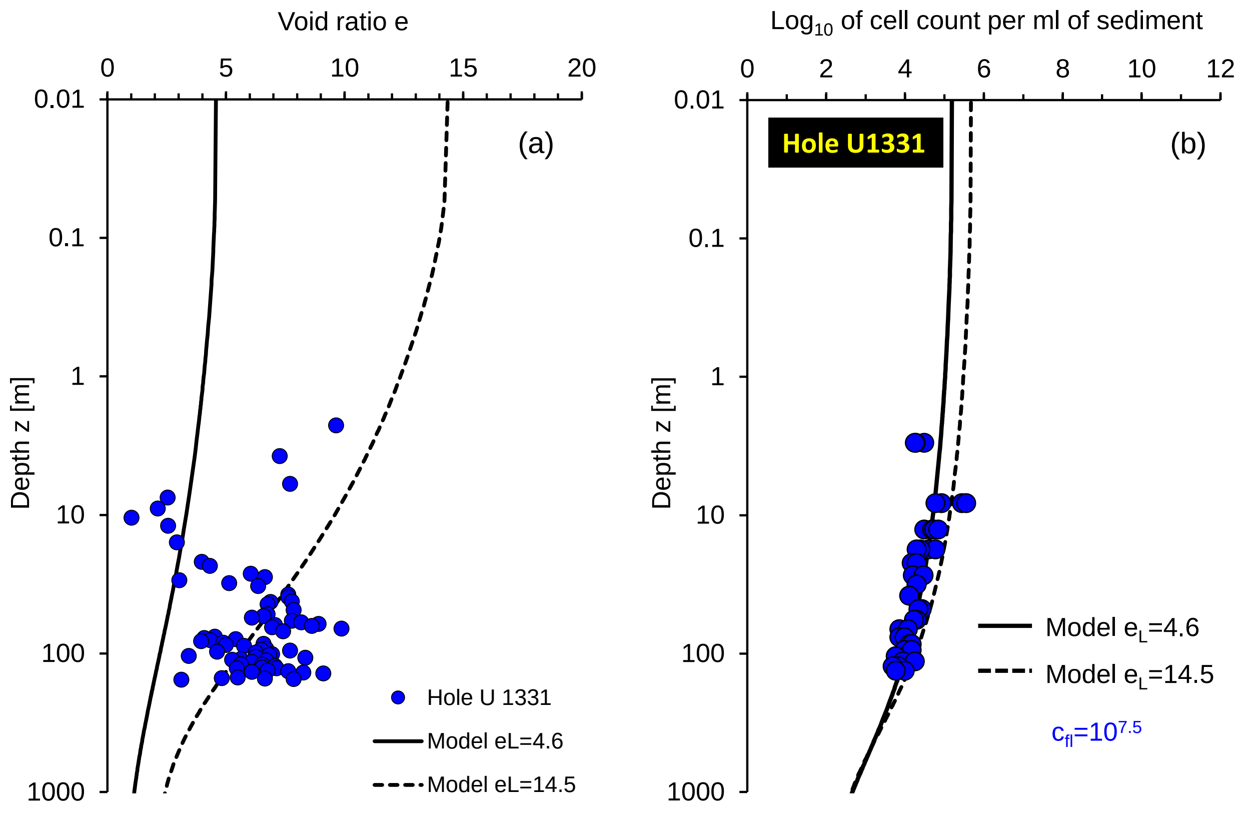

Supplementary Figure S91. Pacific Equatorial: Leg 320/321 - Site U1331. Void ratio and cell count data profiles versus depth and prediction models. (A) Void ratio depth profile - Site U1331 [Data extracted from (Ref. 98)] (model parameters: e_L = 4.6). (B) Cell count profile - Site U1331 [data extracted from (Ref. 98)] (the estimated cell concentration of the pore fluid c_{fl} = $10^{7.5}$ cell counts/cm^3).

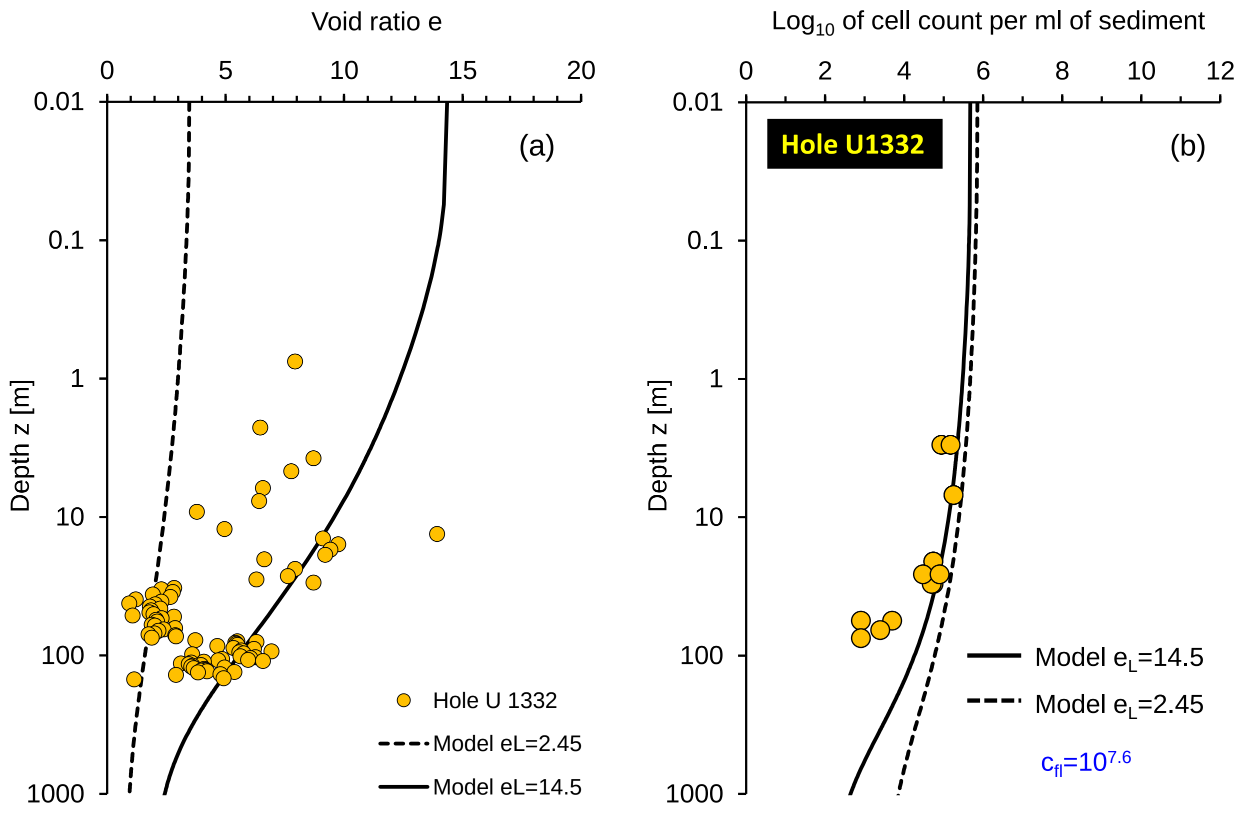

Supplementary Figure S92. Pacific Equatorial: Leg 320/321 - Site U1332. Void ratio and cell count data profiles versus depth and prediction models. (A) Void ratio depth profile - Site U1332 [Data extracted from (Ref. 100)] (model parameters: $e_L = 14.5$). (B) Cell count profile - Site U1332 [data extracted from (Ref. 99)] (the estimated cell concentration of the pore fluid $c_{fl} = 10^{7.6}$ cell counts/cm^3).

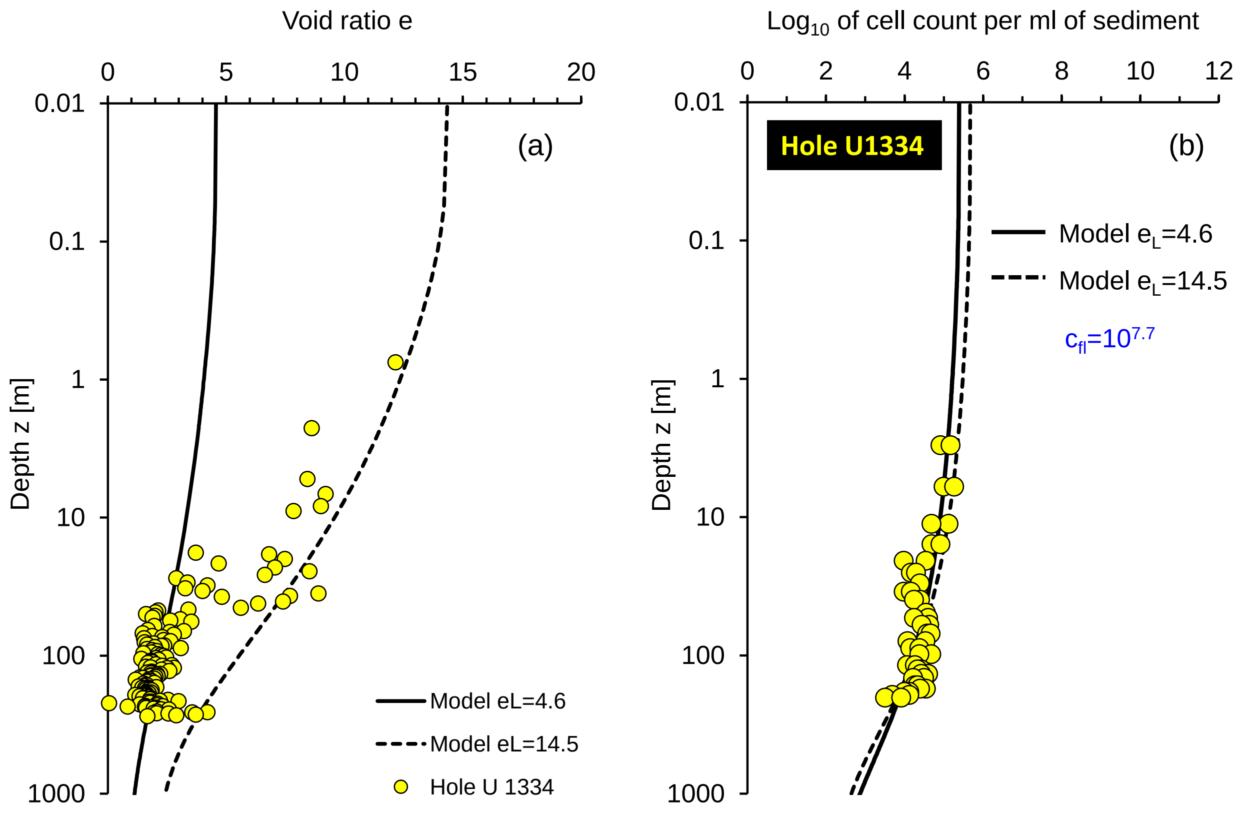

Supplementary Figure S93. Pacific Equatorial: Leg 320/321 - Site U1334. Void ratio and cell count data profiles versus depth and prediction models. (A) Void ratio depth profile - Site U1334 [Data extracted from (Ref. 101)] (model parameters: e_L = 4.6). (B) Cell count profile - Site U1334 [data extracted from (Ref. 99)] (the estimated cell concentration of the pore fluid c_{fl} = $10^{7.7}$ cell counts/cm^3).

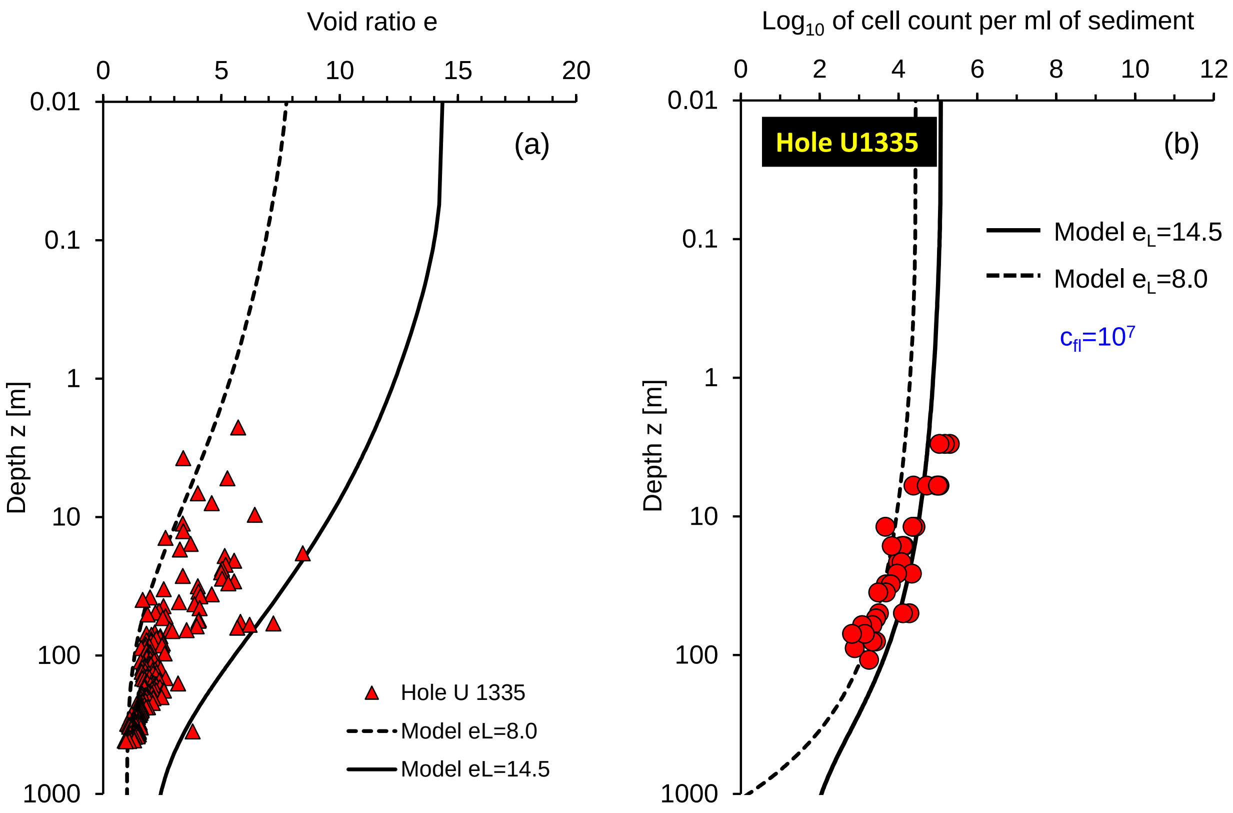

Supplementary Figure S94. Pacific Equatorial: Leg 320/321 - Site U1335. Void ratio and cell count data profiles versus depth and prediction models. (A) Void ratio depth profile - Site U1335 [Data extracted from (Ref. 102)] (model parameters: e_L = 14.5). (B) Cell count profile - Site U1335 [data extracted from (Ref. 99)] (the estimated cell concentration of the pore fluid c_{fl} = 10^7 cell counts/cm^3).

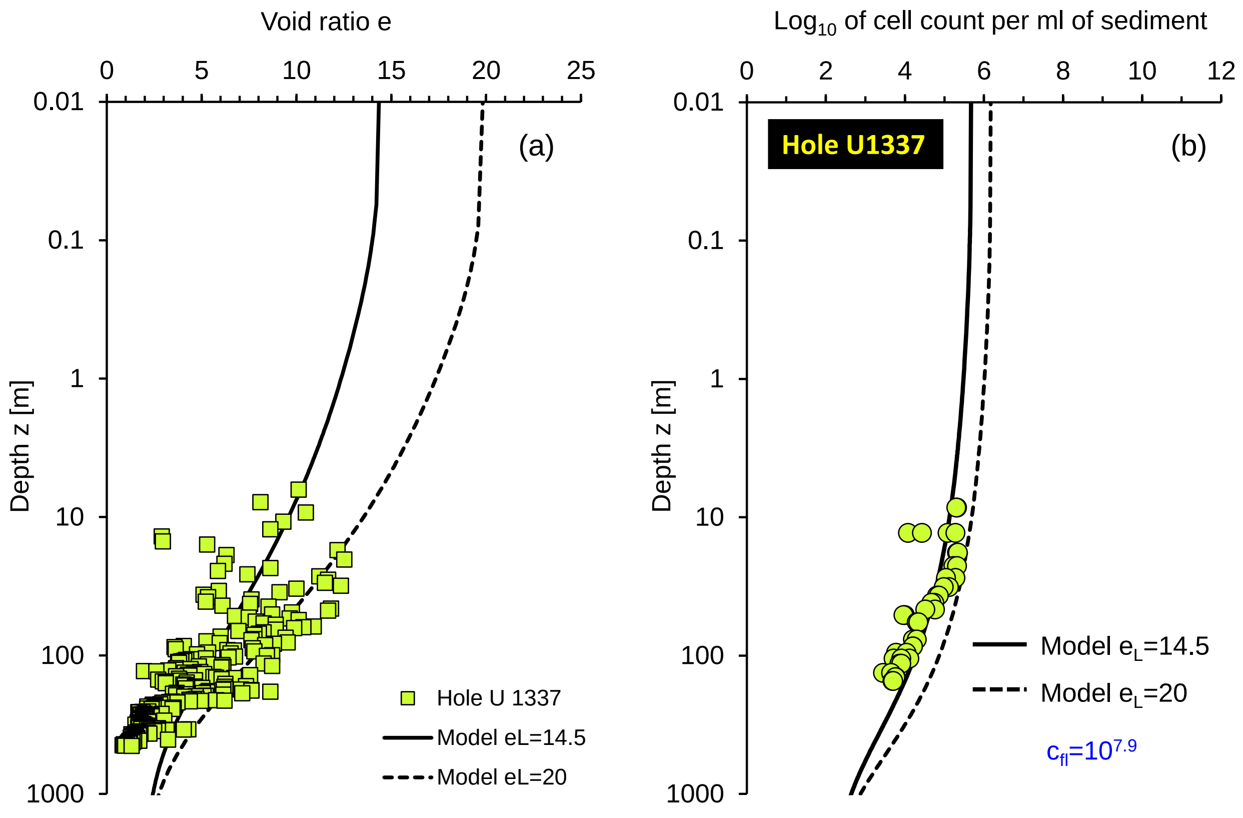

Supplementary Figure S95. Pacific Equatorial: Leg 320/321 - Site U1337. Void ratio and cell count data profiles versus depth and prediction models. (A) Void ratio depth profile - Site U1337 [Data extracted from (Ref. 103)] (model parameters: $e_L = 14.5$). (B) Cell count profile - Site U1337 [data extracted from (Ref. 99)] (the estimated cell concentration of the pore fluid $c_{fl} = 10^{7.9}$ cell counts/cm^3).

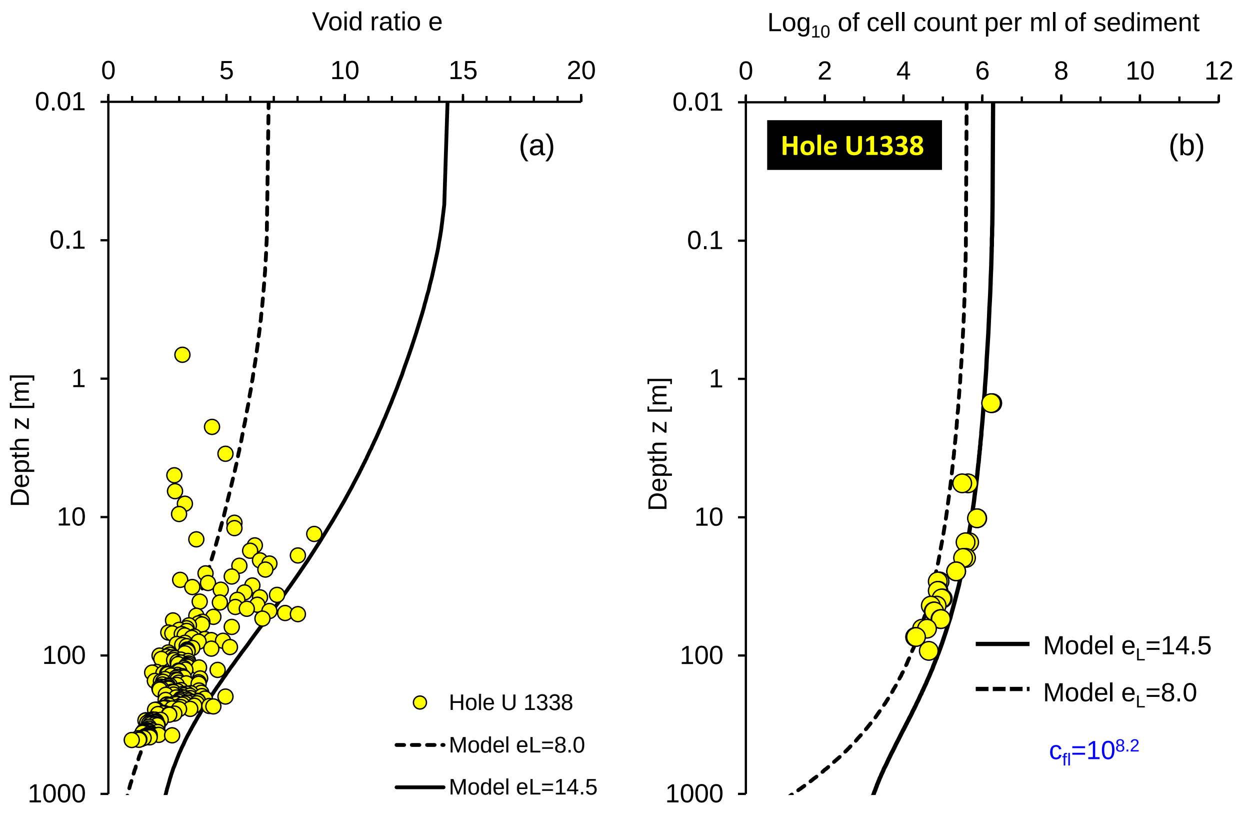

Supplementary Figure S96. Pacific Equatorial: Leg 320/321 - Site U1338. Void ratio and cell count data profiles versus depth and prediction models. (A) Void ratio depth profile - Site U1338 [Data extracted from (Ref. 104)] (model parameters: $e_L = 14.5$). (B) Cell count profile - Site U1338 [data extracted from (Ref. 99)] (the estimated cell concentration of the pore fluid $c_{fl} = 10^{8.2}$ cell counts/cm^3).

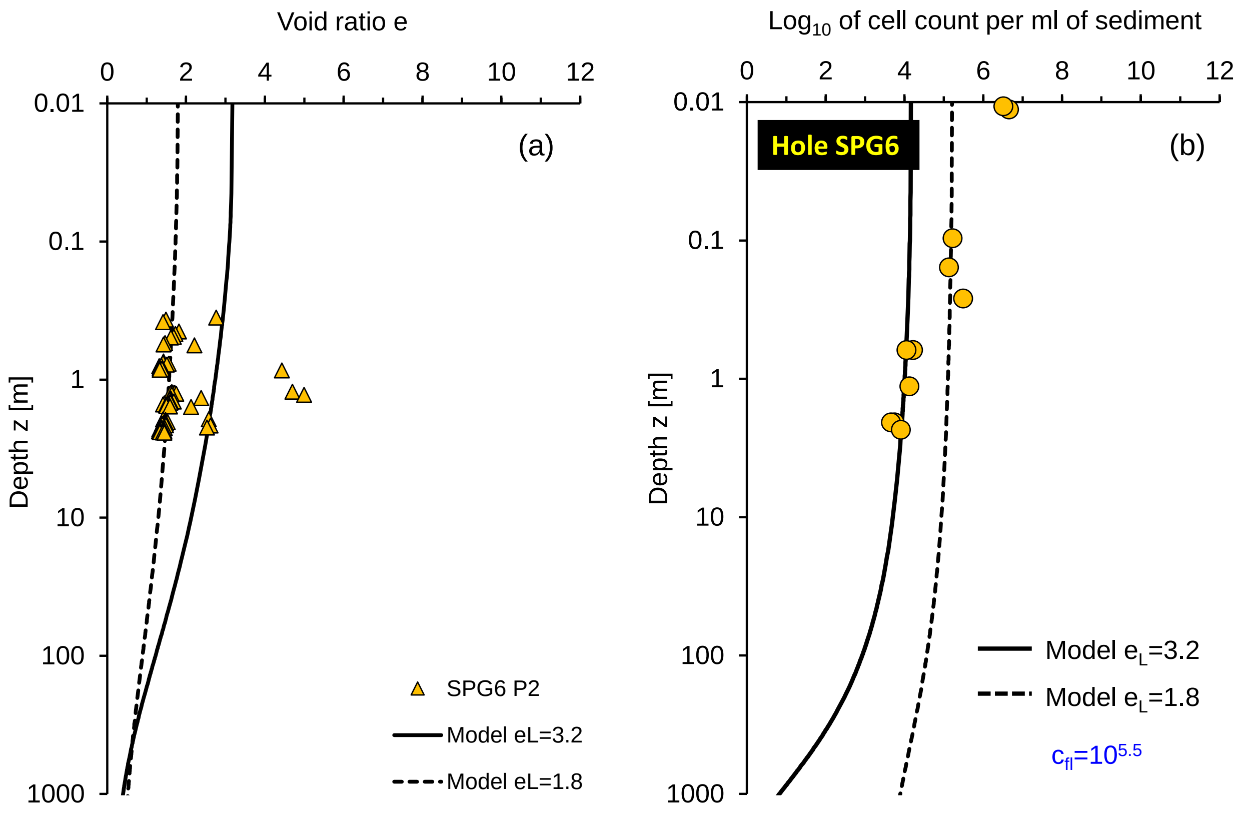

Supplementary Figure S97. South Pacific Gyre: Leg 329 - Site SPG6. Void ratio and cell count data profiles versus depth and prediction models. (A) Void ratio depth profile - Site SPG6 P2 [Data extracted from (Ref. 108)] (model parameters: e_L = 3.2). (B) Cell count profile - Site SPG6 [data extracted from (Ref. 108)] (the estimated cell concentration of the pore fluid c_{fl} = $10^{6.6}$ cell counts/cm^3).

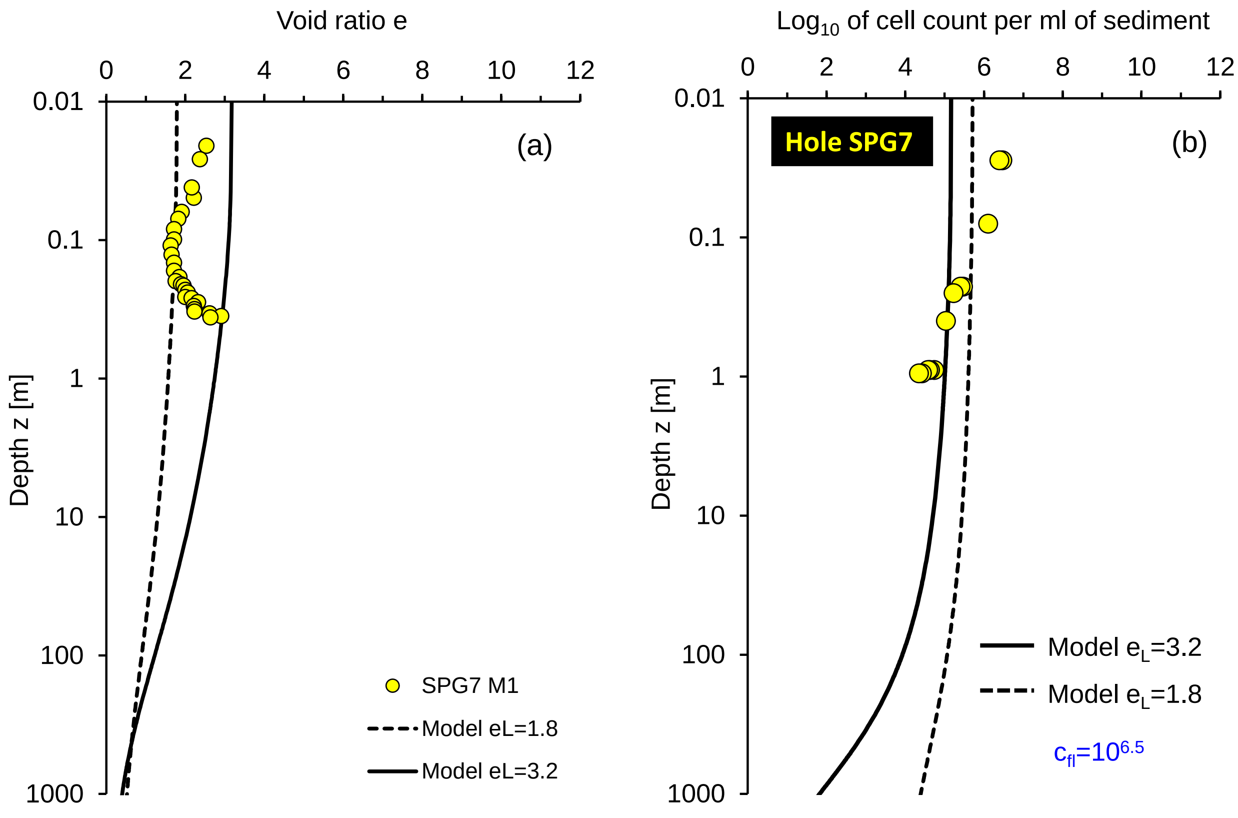

Supplementary Figure S98. South Pacific Gyre: Leg 329 - Site SPG7. Void ratio and cell count data profiles versus depth and prediction models. (A) Void ratio depth profile - Site SPG7 M1 [Data extracted from (Ref. 108)] (model parameters: $e_L = 3.2$). (B) Cell count profile - Site SPG7 [data extracted from (Ref. 108)] (the estimated cell concentration of the pore fluid $c_{fl} = 10^{6.5}$ cell counts/cm^3).

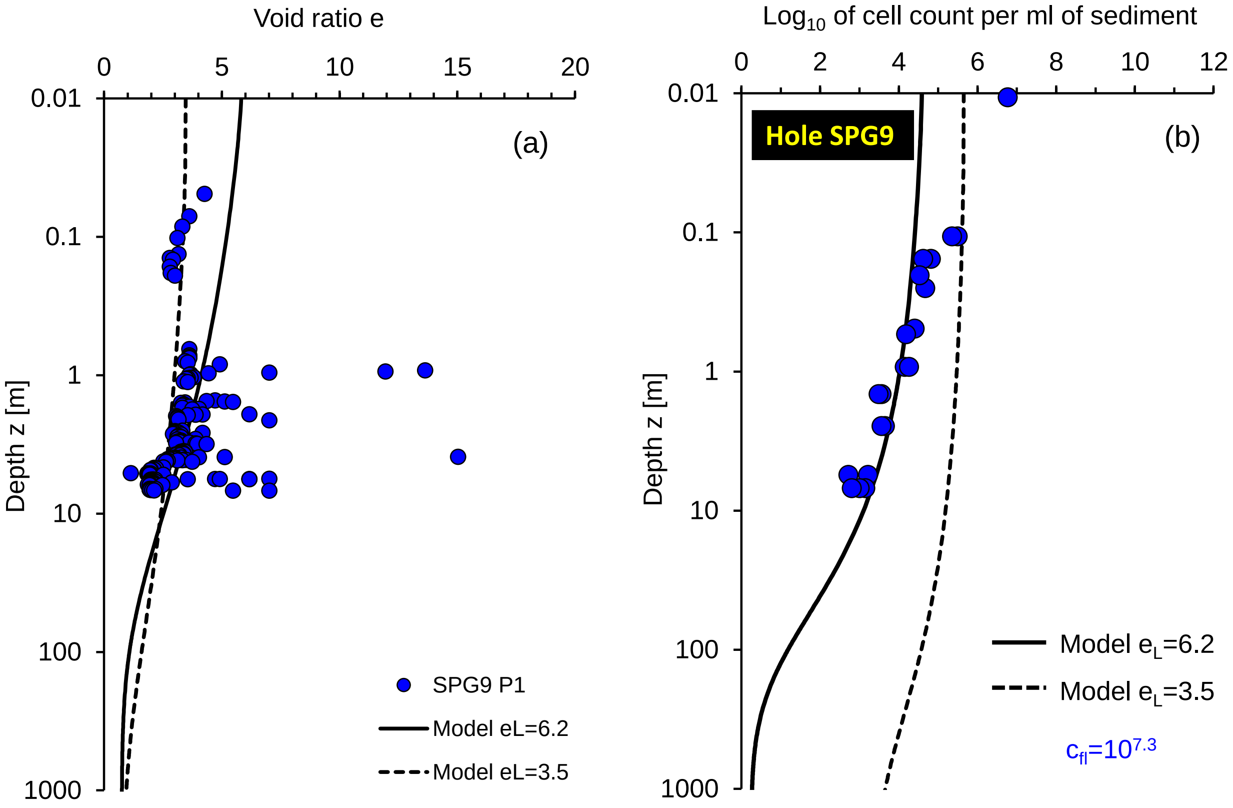

Supplementary Figure S99. South Pacific Gyre: Leg 329 - Site SPG9. Void ratio and cell count data profiles versus depth and prediction models. (A) Void ratio depth profile - Site SPG9 P1 [Data extracted from (Ref. 108)] (model parameters: e_L = 6.2). (B) Cell count profile - Site SPG9 [data extracted from (Ref. 108)] (the estimated cell concentration of the pore fluid c_{fl} = $10^{7.3}$ cell counts/cm^3).

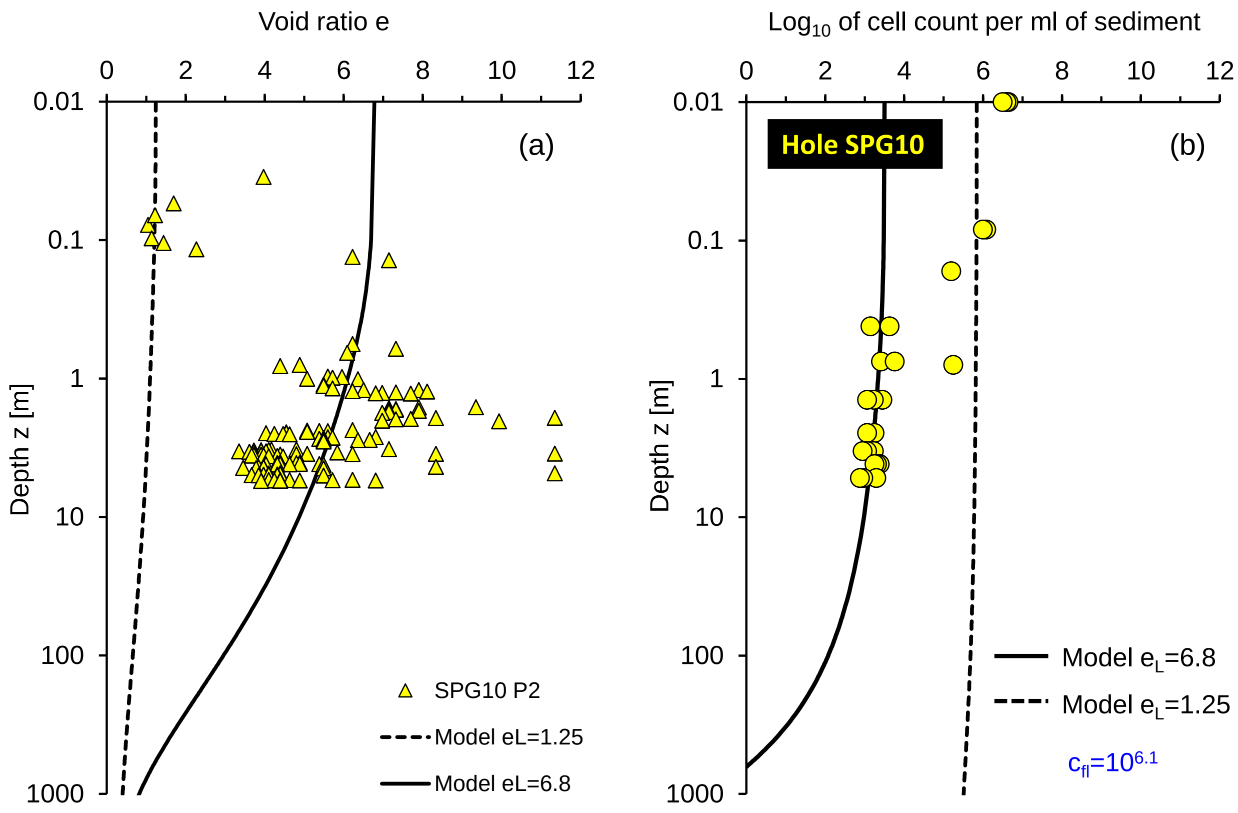

Supplementary Figure S100. South Pacific Gyre: Leg 329 - Site SPG10. Void ratio and cell count data profiles versus depth and prediction models. (A) Void ratio depth profile - Site SPG10 P2 [Data extracted from (Ref. 108)] (model parameters: e_L = 6.8). (B) Cell count profile - Site SPG10 [data extracted from (Ref. 108)] (the estimated cell concentration of the pore fluid c_{fl} = $10^{6.1}$ cell counts/cm^3).

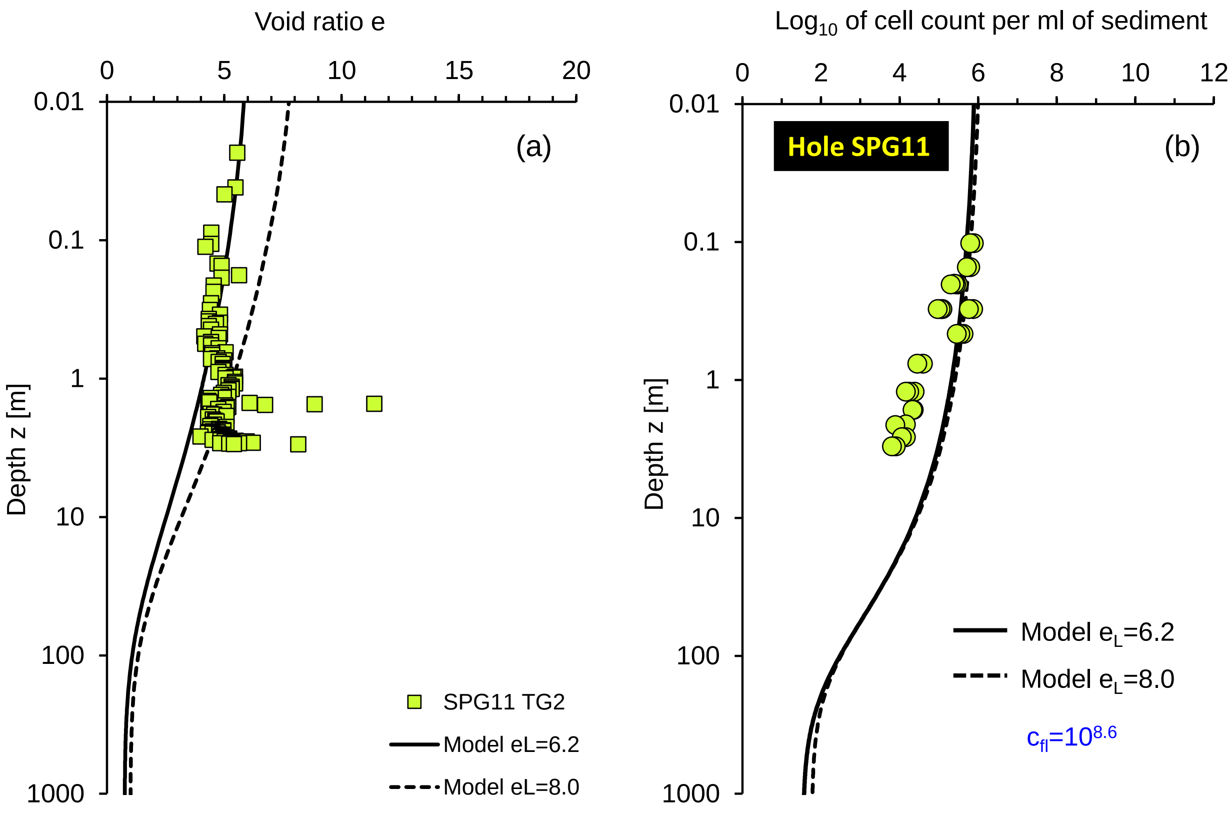

Supplementary Figure S101. South Pacific Gyre: Leg 329 - Site SPG11. Void ratio and cell count data profiles versus depth and prediction models. (A) Void ratio depth profile - Site SPG11 TG2 [Data extracted from (Ref. 108)] (model parameters: e_L = 6.2). (B) Cell count profile - Site SPG11 [data extracted from (Ref. 108)] (the estimated cell concentration of the pore fluid c_{fl} = $10^{8.6}$ cell counts/cm^3).

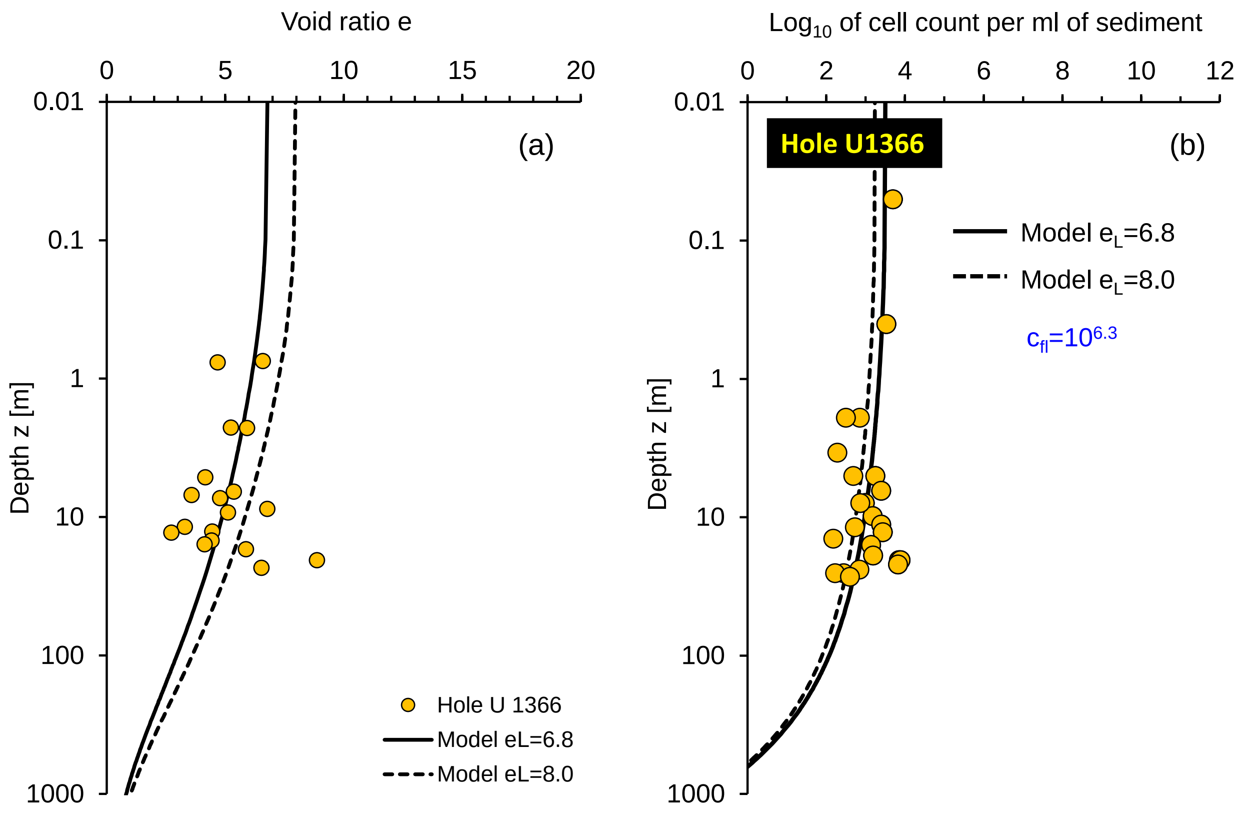

Supplementary Figure S102. South Pacific Gyre: Leg 329 - Site U1366. Void ratio and cell count data profiles versus depth and prediction models. (A) Void ratio depth profile - Site U1366 [Data extracted from (Ref. 110)] (model parameters: e_L = 6.8). (B) Cell count profile - Site U1366 [data extracted from (Ref. 110)] (the estimated cell concentration of the pore fluid c_{fl} = $10^{6.3}$ cell counts/cm^3).

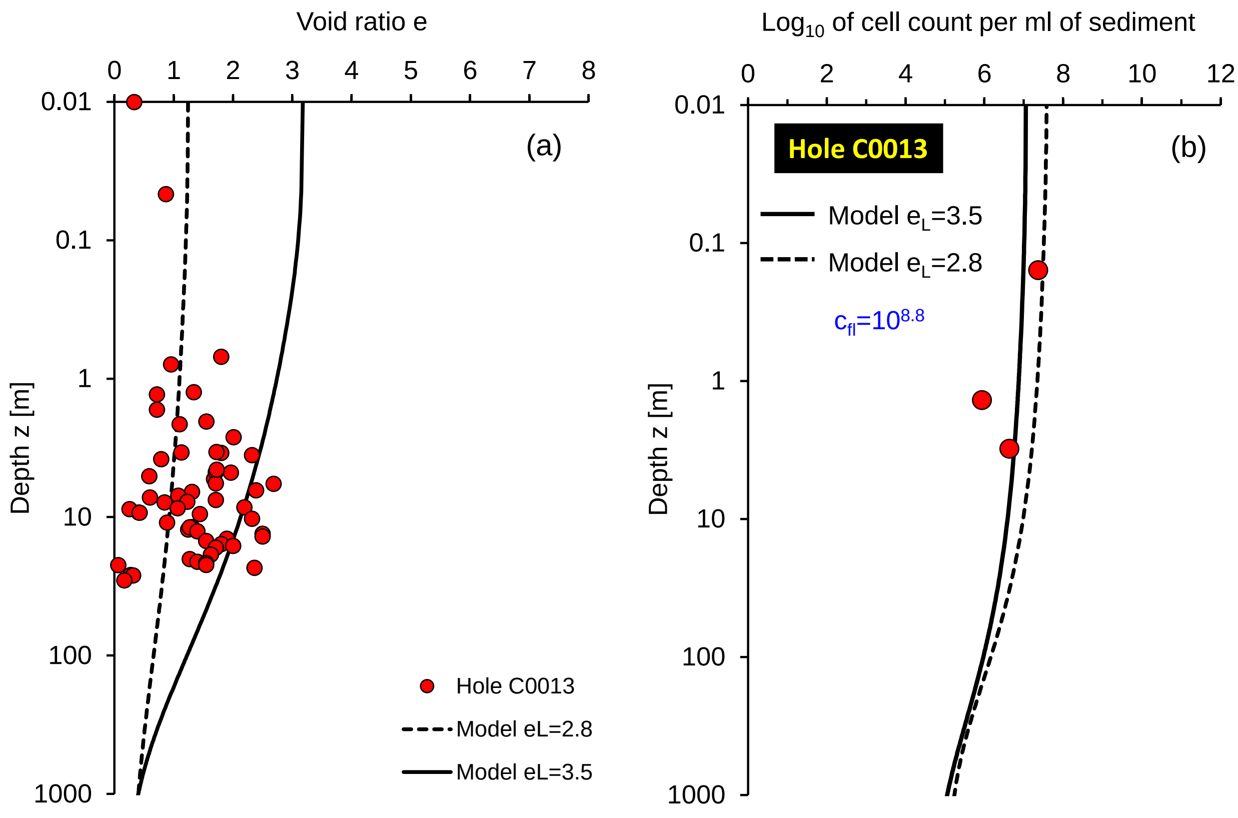

Supplementary Figure S103. Mid-Okinawa Trough: Leg 331 - Site C0013. Void ratio and cell count data profiles versus depth and prediction models. (A) Void ratio depth profile - Site C0013C-to-F [Data extracted from (Ref. 116)] (model parameters: e_L = 3.5). (B) Cell count profile - Site C0013 [data extracted from (Ref. 116)] (the estimated cell concentration of the pore fluid $c_{fl} = 10^{8.8}$ cell counts/cm^3).

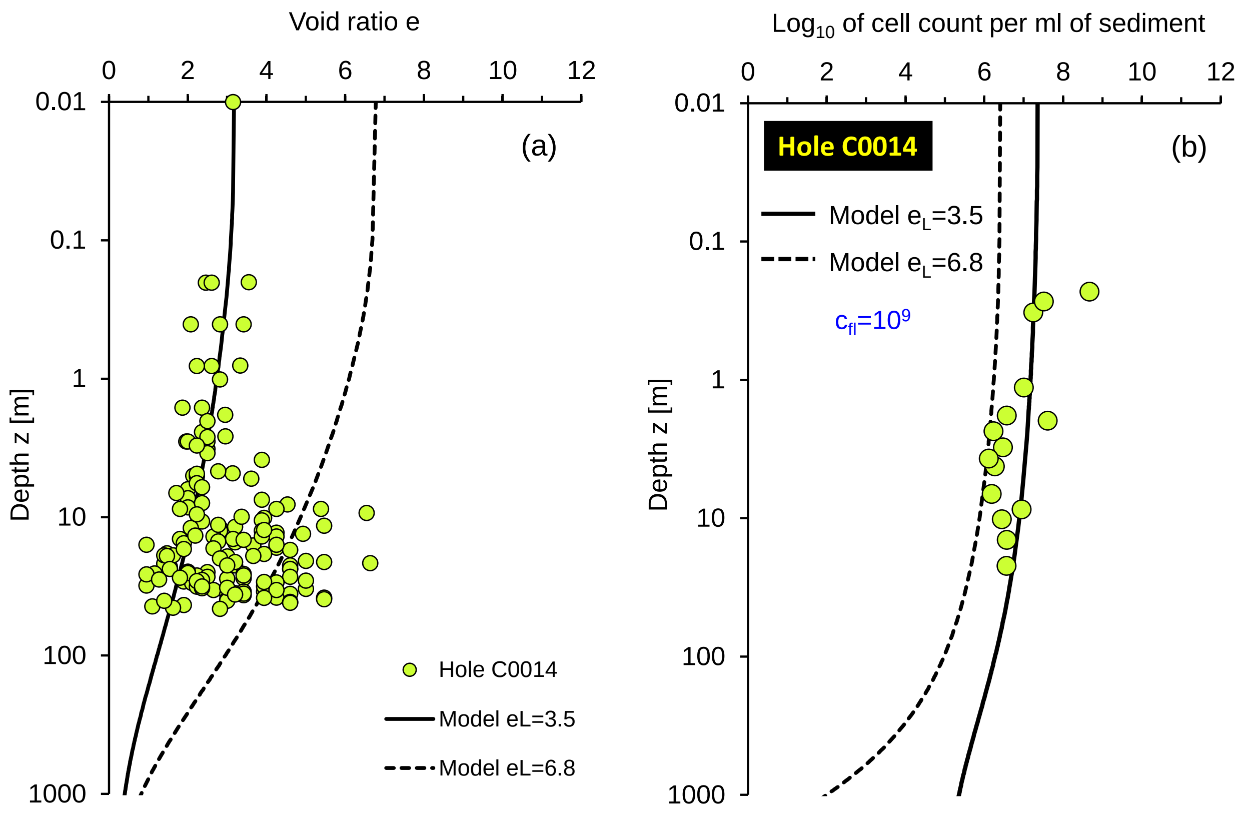

Supplementary Figure S104. Mid-Okinawa Trough: Leg 331 - Site C0014. Void ratio and cell count data profiles versus depth and prediction models. (A) Void ratio depth profile - Site C0014A-to-G [Data extracted from (Ref. 117)] (model parameters: e_L = 3.5). (B) Cell count profile - Site C0014B/D/E/G [data extracted from (Ref. 117)] (the estimated cell concentration of the pore fluid c_{fl} = 10^9 cell counts/cm^3).

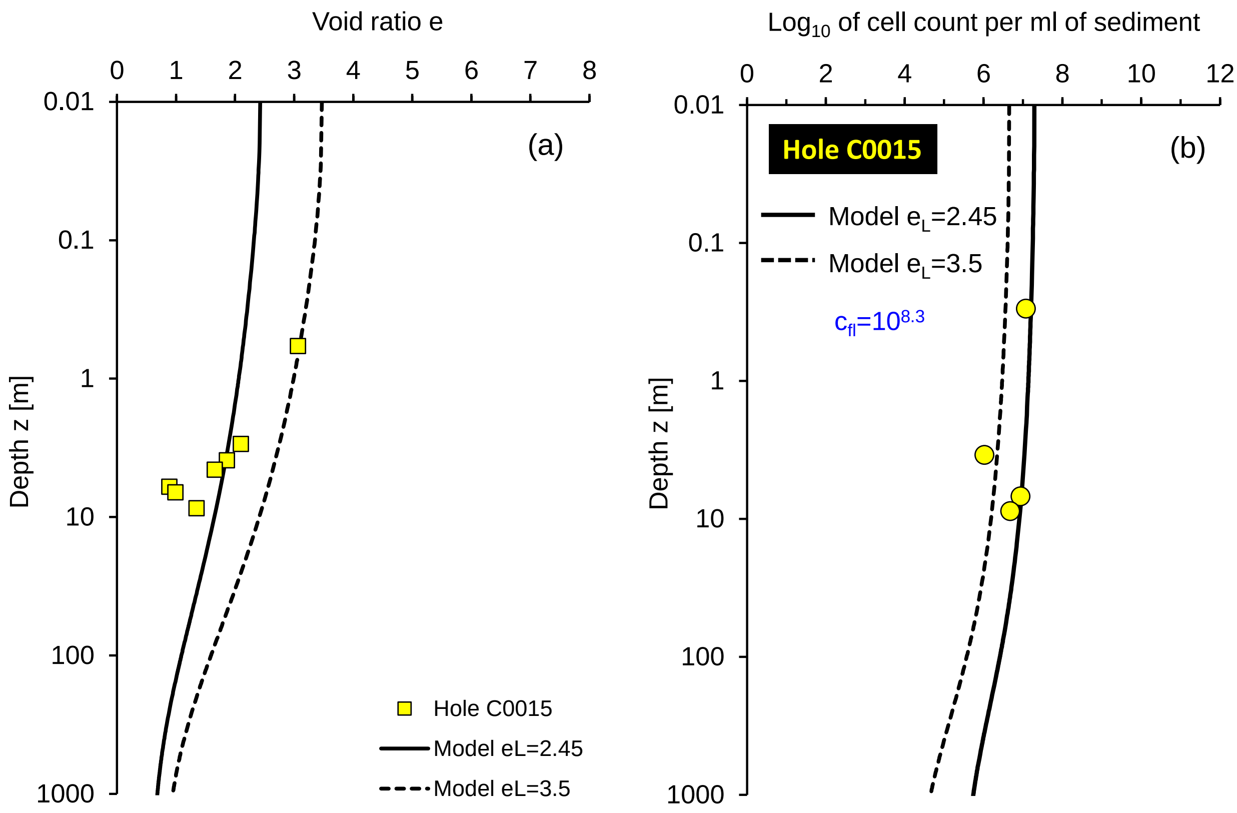

Supplementary Figure S105. Mid-Okinawa Trough: Leg 331 - Site C0015. Void ratio and cell count data profiles versus depth and prediction models. (A) Void ratio depth profile - Site C0015 [Data extracted from (Ref. 118)] (model parameters: e_L = 2.45). (B) Cell count profile - Site C0015B/C [data extracted from (Ref. 118)] (the estimated cell concentration of the pore fluid c_{fl} = $10^{8.3}$ cell counts/cm^3).

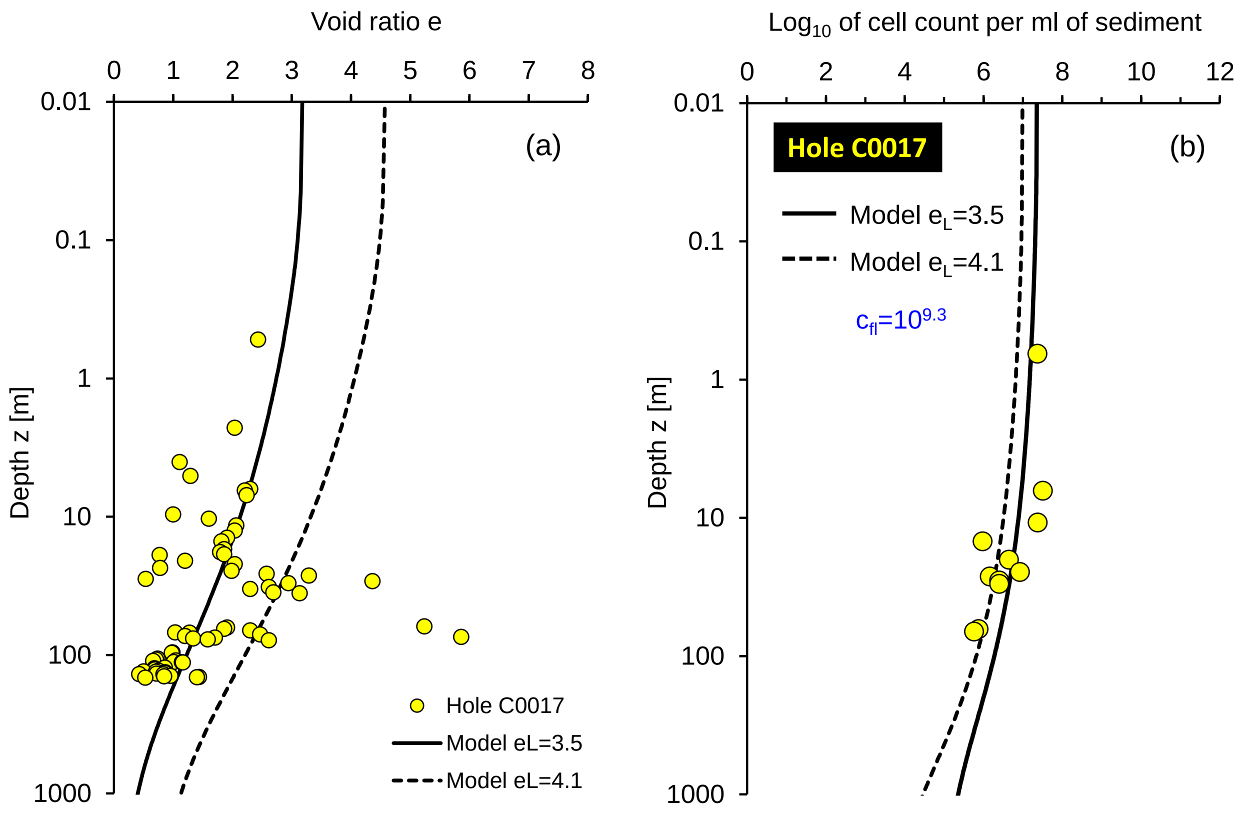

Supplementary Figure S106. Mid-Okinawa Trough: Leg 331 - Site C0017. Void ratio and cell count data profiles versus depth and prediction models. (A) Void ratio depth profile - Site C0017 [Data extracted from (Ref. 119)] (model parameters: e_L = 3.5). (B) Cell count profile - Site C0017A/B/C/D [data extracted from (Ref. 119)] (the estimated cell concentration of the pore fluid $c_{fl} = 10^{9.3}$ cell counts/cm^3).

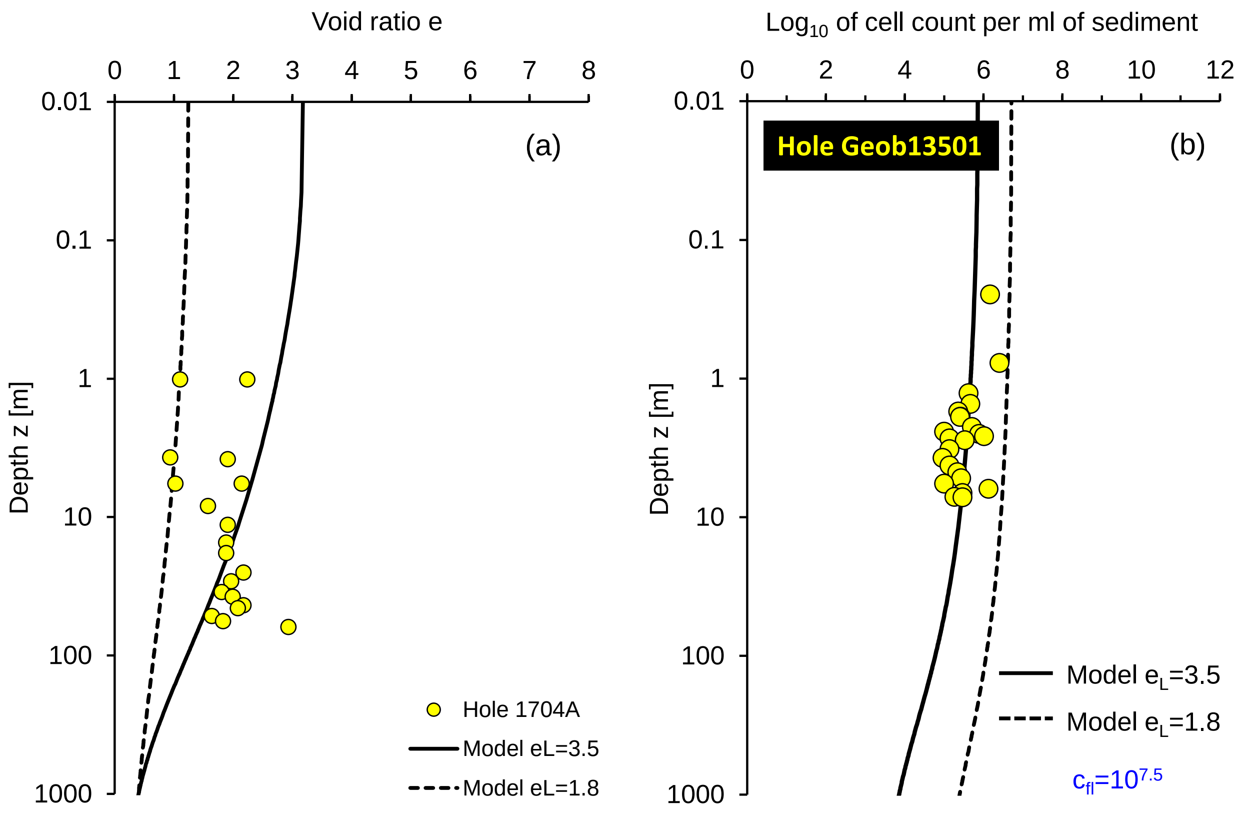

Supplementary Figure S107. Mid-Atlantic Ridge: Leg 336 - Site Geob13501. Void ratio and cell count data profiles versus depth and prediction models. (A) Void ratio depth profile - Site 1704A/1382/1383 [Data extracted from (Refs. 120-122)] (model parameters: e_L = 3.5). (B) Cell count profile - Site Geob13501 [data extracted from (Ref. 123)] (the estimated cell concentration of the pore fluid c_{fl} = $10^{7.5}$ cell counts/cm^3).

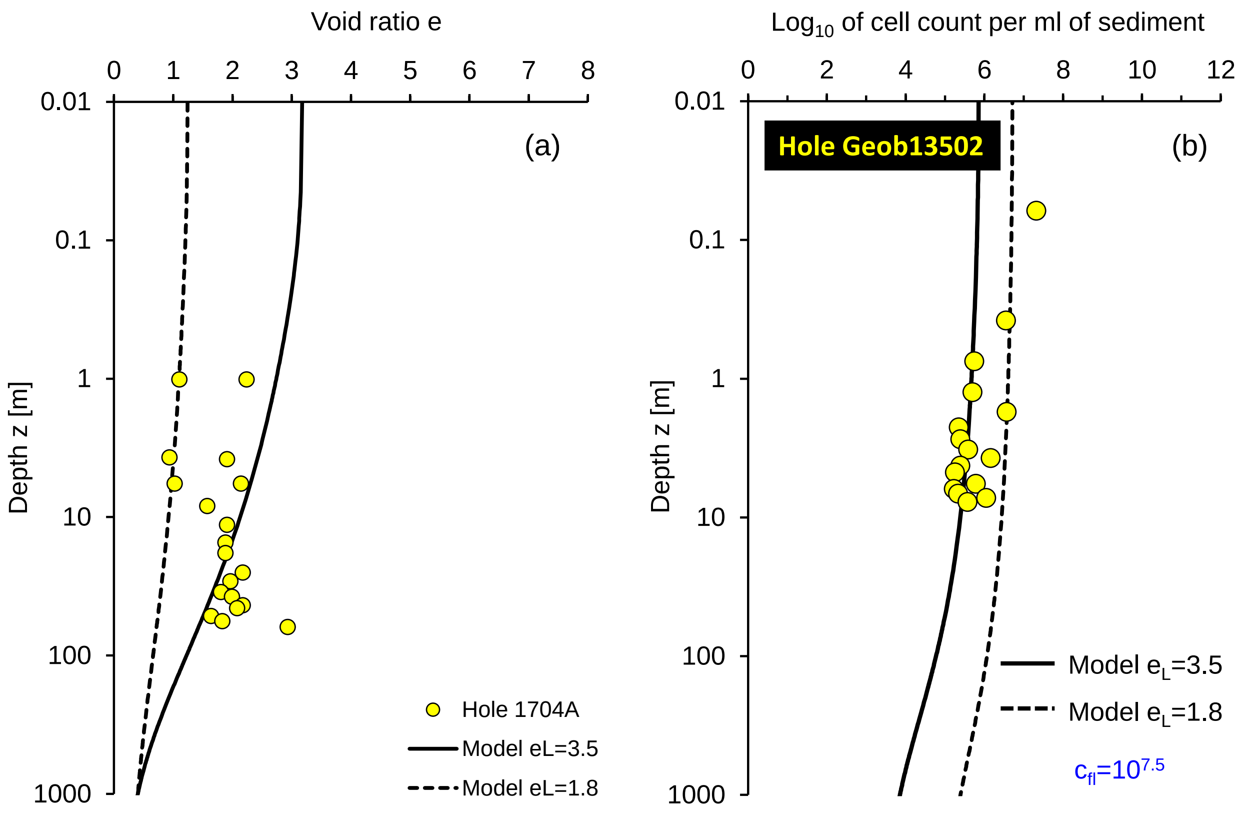

Supplementary Figure S108. Mid-Atlantic Ridge: Leg 336 - Site Geob13502. Void ratio and cell count data profiles versus depth and prediction models. (A) Void ratio depth profile - Site 1704A/1382/1383 [Data extracted from (Refs. 120-122)] (model parameters: e_L = 3.5). (B) Cell count profile - Site Geob13502 [data extracted from (Ref. 123)] (the estimated cell concentration of the pore fluid c_{fl} = $10^{7.5}$ cell counts/cm^3).

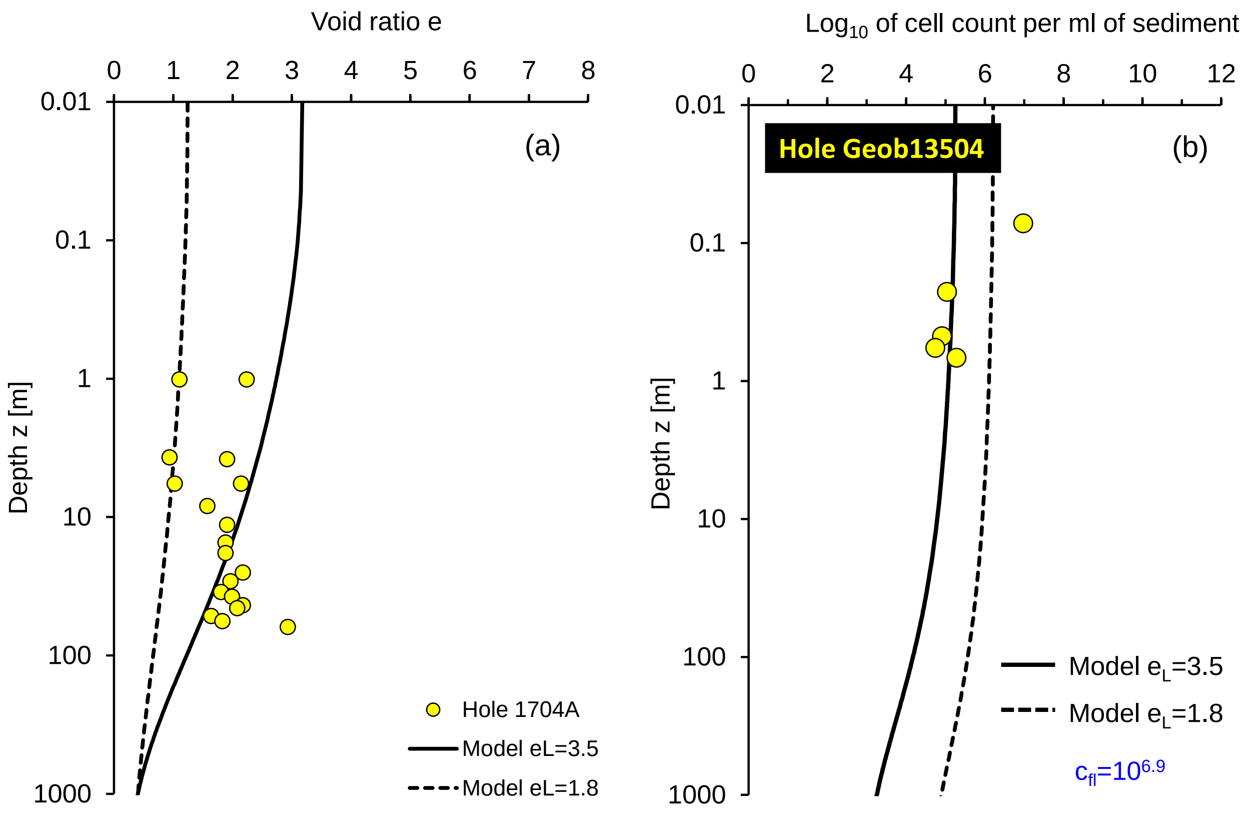

Supplementary Figure S109. Mid-Atlantic Ridge: Leg 336 - Site Geob13504. Void ratio and cell count data profiles versus depth and prediction models. (A) Void ratio depth profile - Site 1704A/1382/1383 [Data extracted from (Refs. 120-122)] (model parameters: e_L = 3.5). (B) Cell count profile - Site Geob13504 [data extracted from (Ref. 123)] (the estimated cell concentration of the pore fluid $c_{fl} = 10^{6.9}$ cell counts/cm^3).

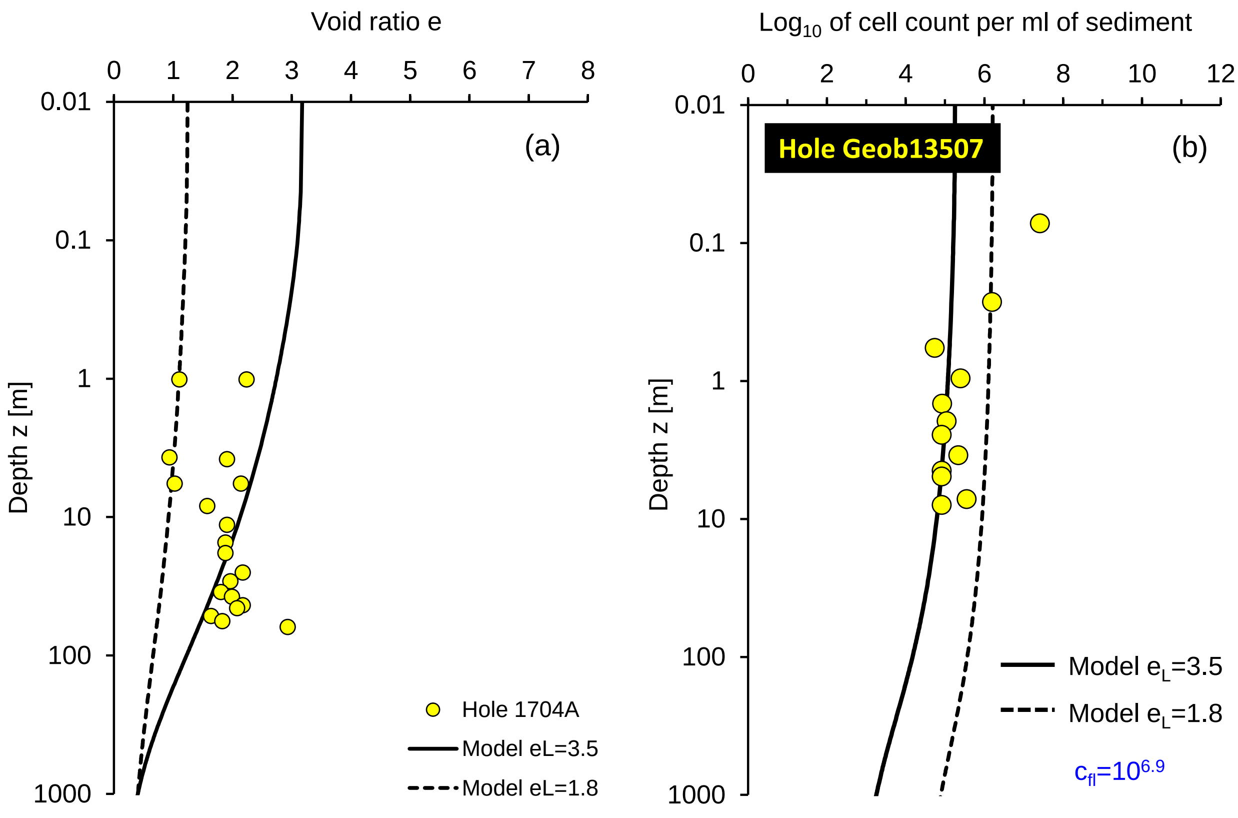

Supplementary Figure S110. Mid-Atlantic Ridge: Leg 336 - Site Geob13507. Void ratio and cell count data profiles versus depth and prediction models. (A) Void ratio depth profile - Site 1704A/1382/1383 [Data extracted from (Refs. 120-122)] (model parameters: e_L = 3.5). (B) Cell count profile - Site Geob13507 [data extracted from (Ref. 123)] (the estimated cell concentration of the pore fluid $c_{fl} = 10^{6.9}$ cell counts/cm^3).

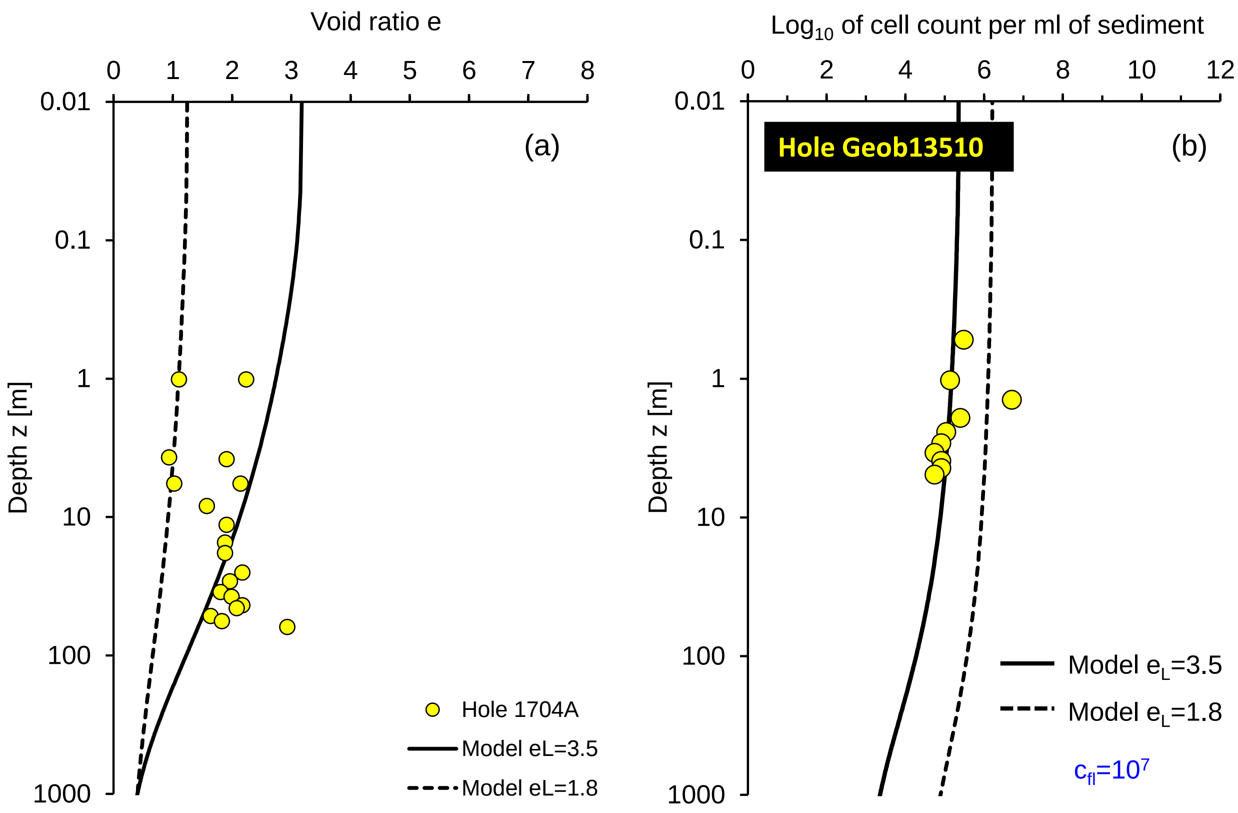

Supplementary Figure S111. Mid-Atlantic Ridge: Leg 336 - Site Geob13510. Void ratio and cell count data profiles versus depth and prediction models. (A) Void ratio depth profile - Site 1704A/1382/1383 [Data extracted from (Refs. 120-122)] (model parameters: e_L = 3.5). (B) Cell count profile - Site Geob13510 [data extracted from (Ref. 123)] (the estimated cell concentration of the pore fluid c_{fl} = 10^7 cell counts/cm^3).

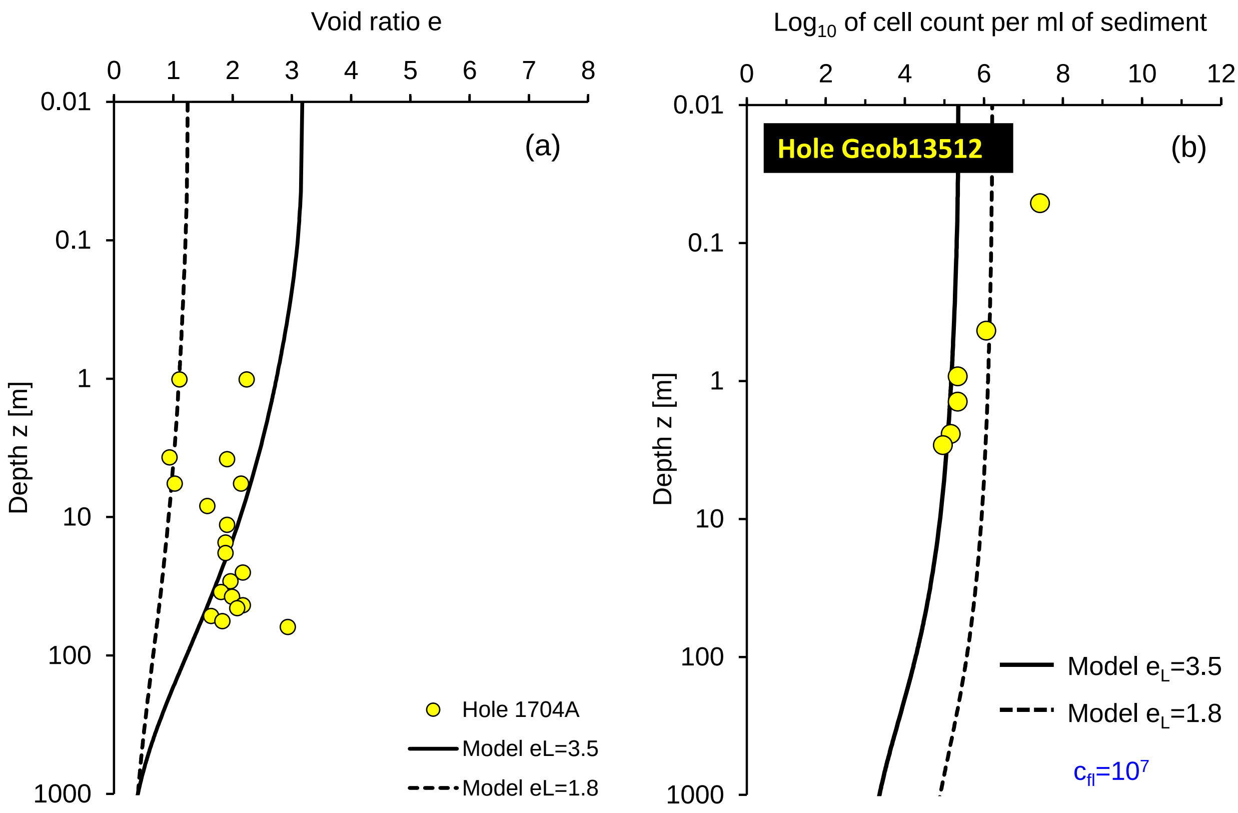

Supplementary Figure S112. Mid-Atlantic Ridge: Leg 336 - Site Geob13512. Void ratio and cell count data profiles versus depth and prediction models. (A) Void ratio depth profile - Site 1704A/1382/1383 [Data extracted from (Refs. 120-122)] (model parameters: e_L = 3.5). (B) Cell count profile - Site Geob13512 [data extracted from (Ref. 123)] (the estimated cell concentration of the pore fluid c_{fl} = 10^7 cell counts/cm^3).

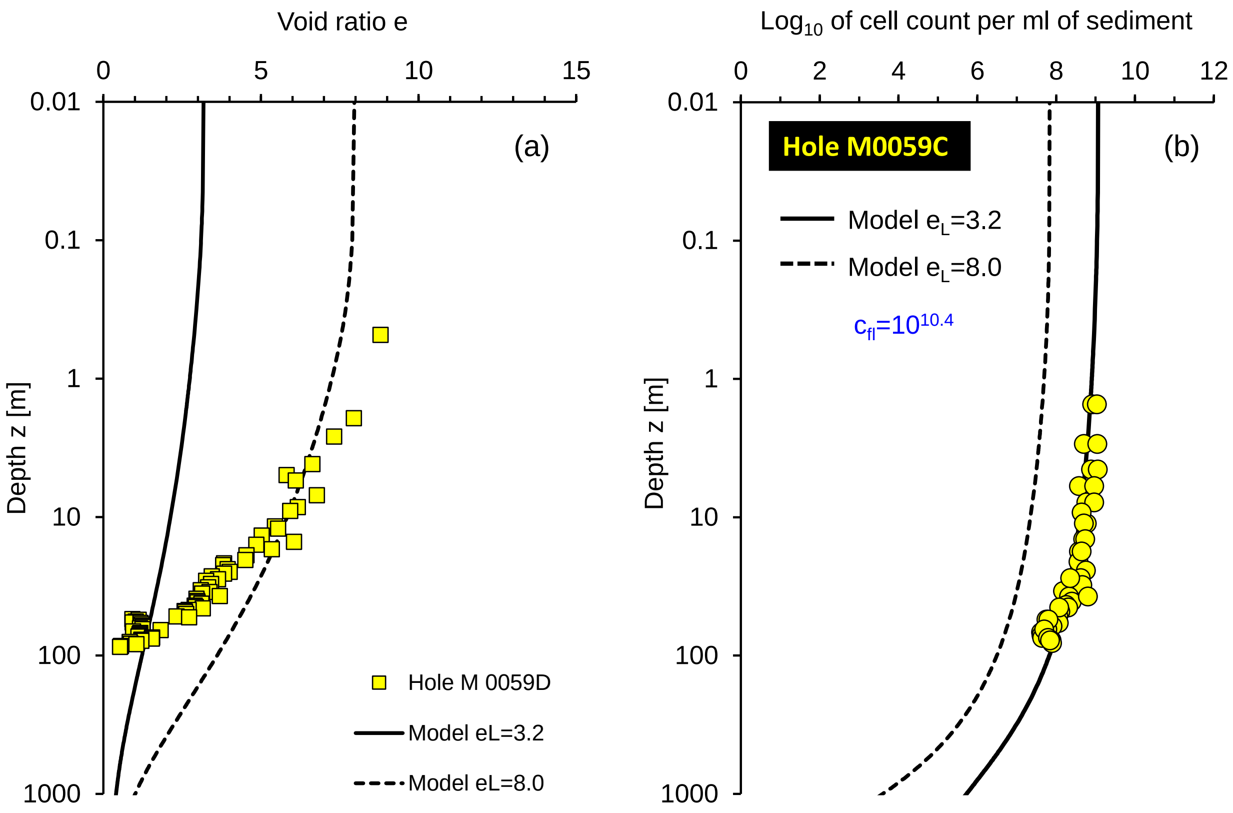

Supplementary Figure S113. Baltic Sea Basin: Leg 347 - Site M0059C. Void ratio and cell count data profiles versus depth and prediction models. (A) Void ratio depth profile - M0059D [Data extracted from (Ref. 125)] (model parameters: e_L = 3.2). (B) Cell count profile - Site M0059C [data extracted from (Ref. 125)] (the estimated cell concentration of the pore fluid c_{fl} = $10^{10.4}$ cell counts/cm^3).

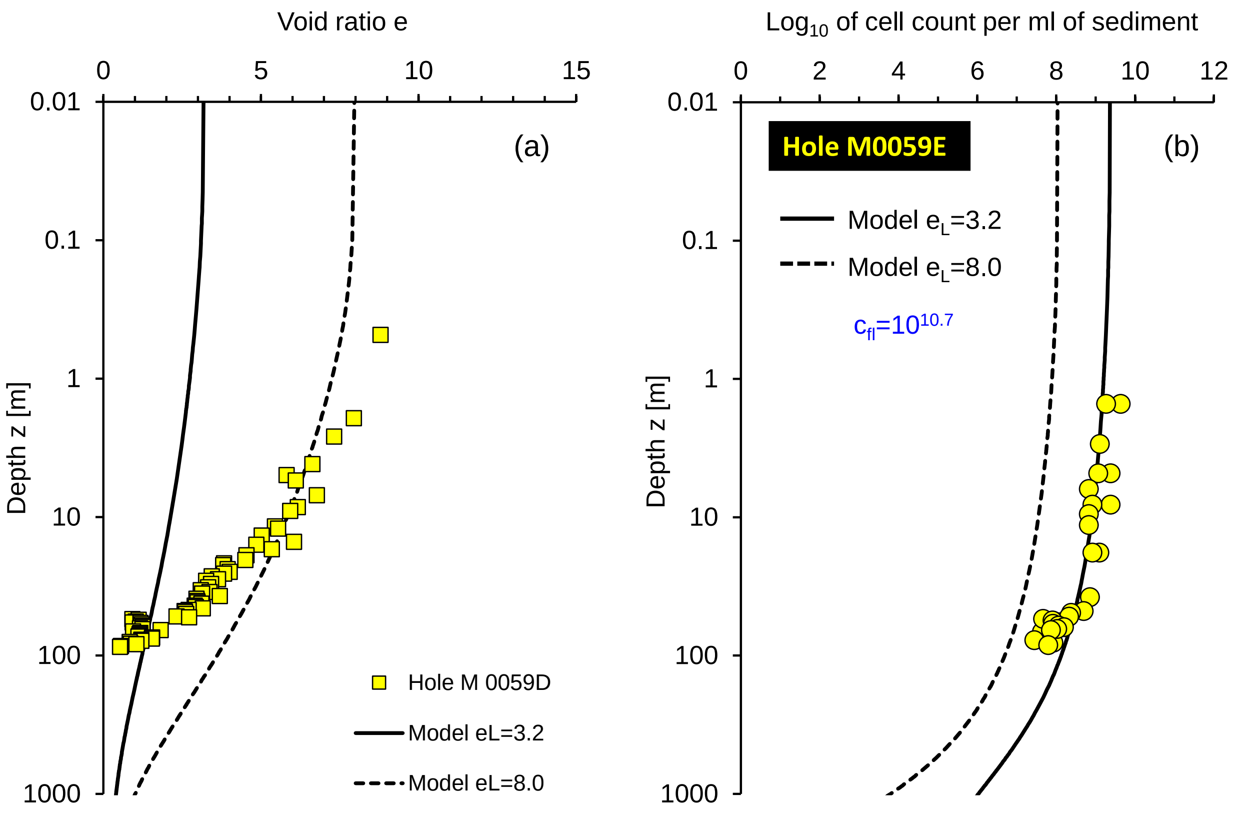

Supplementary Figure S114. Baltic Sea Basin: Leg 347 - Site M0059E. Void ratio and cell count data profiles versus depth and prediction models. (A) Void ratio depth profile - M0059D [Data extracted from (Ref. 125)] (model parameters: e_L = 3.2). (B) Cell count profile - Site M0059E [data extracted from (Ref. 125)] (the estimated cell concentration of the pore fluid $c_{fl} = 10^{10.7}$ cell counts/cm^3).

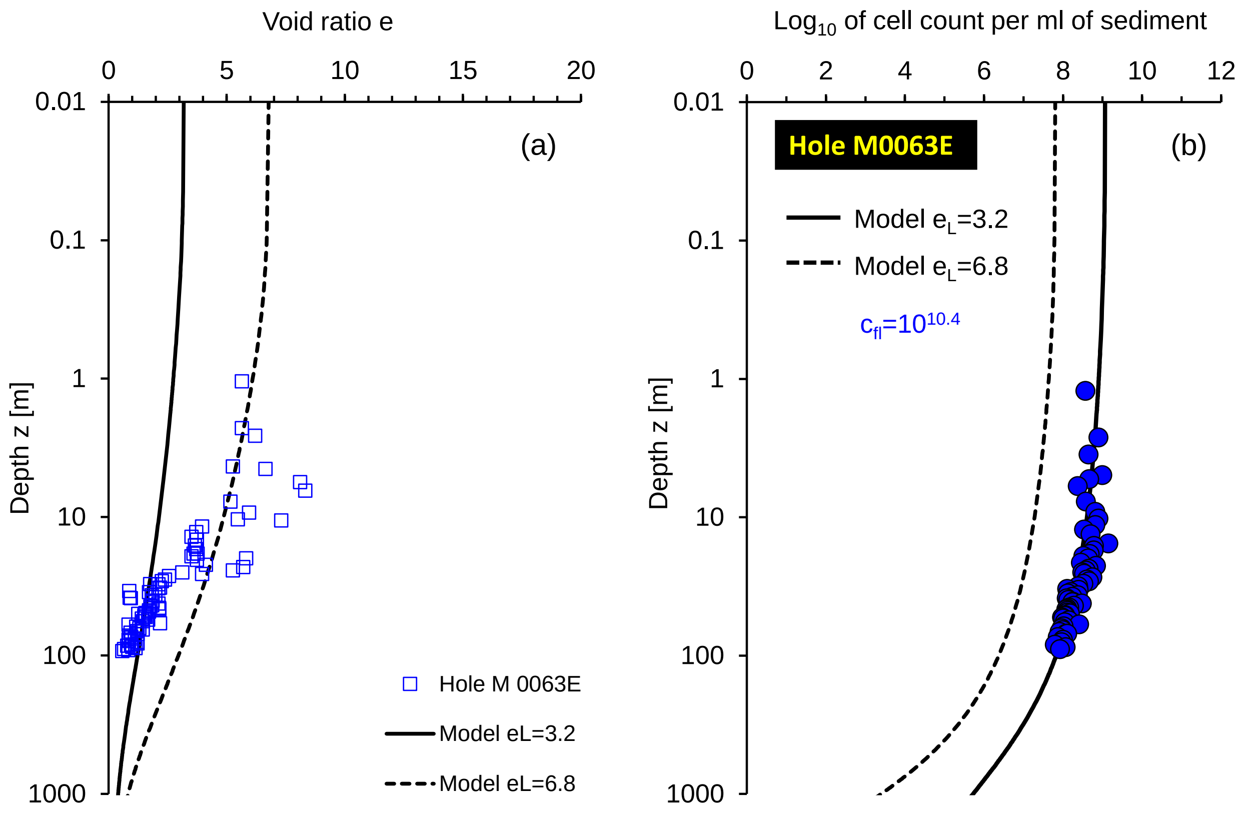

Supplementary Figure S115. Baltic Sea Basin: Leg 347 - Site M0063E. Void ratio and cell count data profiles versus depth and prediction models. (A) Void ratio depth profile – Site M0063E [Data extracted from (Ref. 128)] (model parameters: e_L = 3.2). (B) Cell count profile - Site M0063E [data extracted from (Ref. 128)] (the estimated cell concentration of the pore fluid $c_{fl} = 10^{10.4}$ cell counts/cm^3).

VOID RATIO AND CELL COUNT PROFILES

Incomplete data

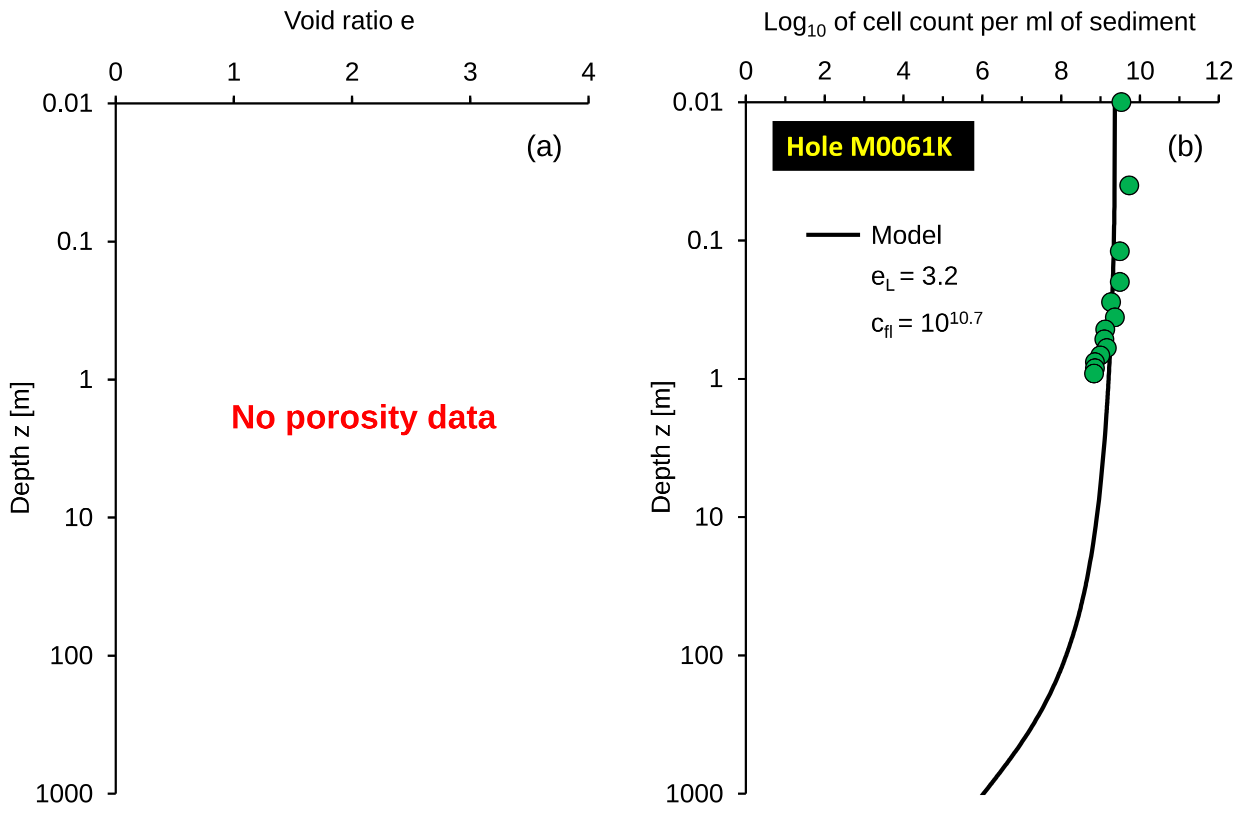

Supplementary Figure S116. Baltic Sea Basin: Leg 347 - Site M0061K. Void ratio and cell count data profiles versus depth and prediction models. (A) Void ratio depth profile – Not available (model parameters: e_L = 3.2). (B) Cell count profile - Site M0061K [data extracted from (Ref. 127)] (the estimated cell concentration of the pore fluid $c_{fl} = 10^{10.7}$ cell counts/cm^3).

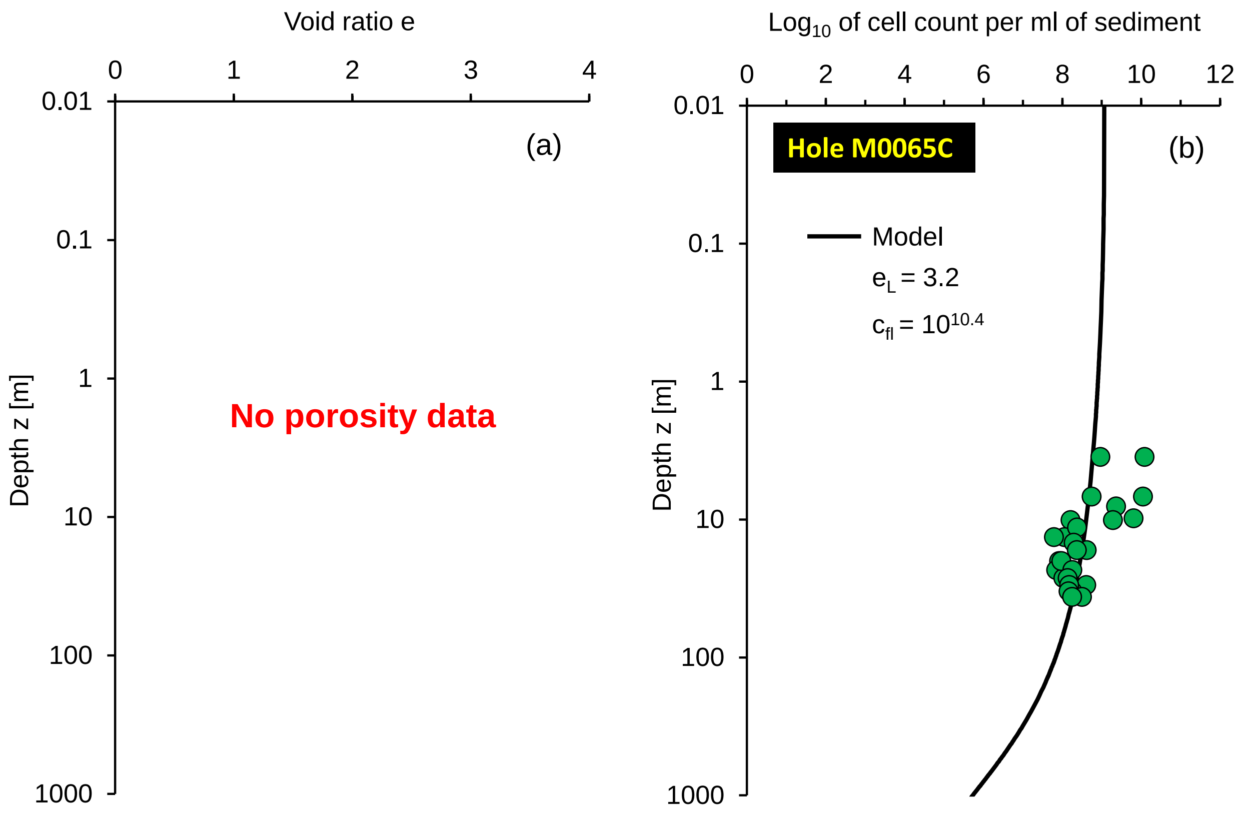

Supplementary Figure S117. Baltic Sea Basin: Leg 347 - Site M0065C. Void ratio and cell count data profiles versus depth and prediction models. (A) Void ratio depth profile – Not available (model parameters: $e_L = 3.2$). (B) Cell count profile - Site M0065C [data extracted from (Ref. 129)] (the estimated cell concentration of the pore fluid $c_{fl} = 10^{10.4}$ cell counts/cm^3).

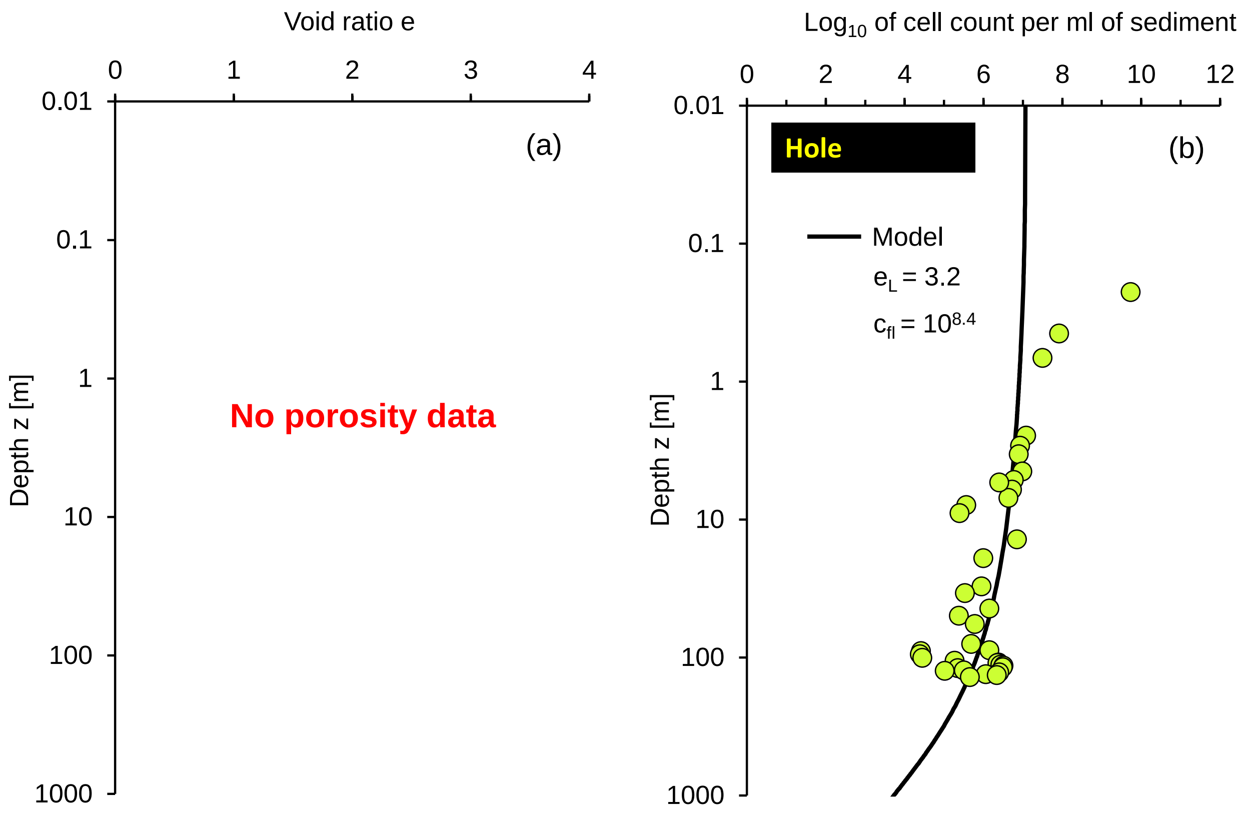

Supplementary Figure S118. Chesapeake Bay. Void ratio and cell count data profiles versus depth and prediction models. (A) Void ratio depth profile – Not available (model parameters: e_L = 3.2). (B) Cell count profile [data extracted from (Ref. 131)] (the estimated cell concentration of the pore fluid $c_{fl} = 10^{8.4}$ cell counts/cm^3).

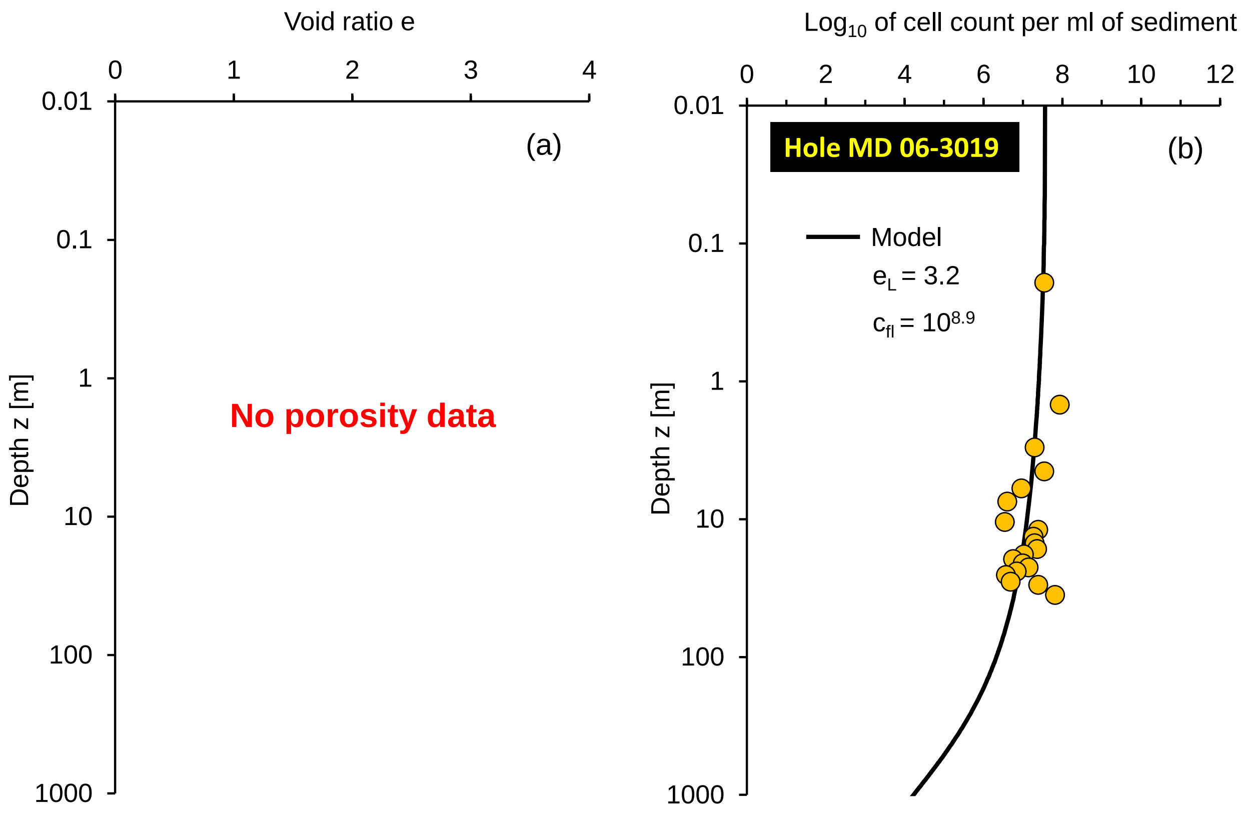

Supplementary Figure S119. New Caledonia - Site MD 06-3019. Void ratio and cell count data profiles versus depth and prediction models. (A) Void ratio depth profile – Not available (model parameters: e_L = 3.2). (B) Cell count profile - Site MD 06-3019 [data extracted from (Ref. 132)] (the estimated cell concentration of the pore fluid $c_{fl} = 10^{8.9}$ cell counts/cm^3).

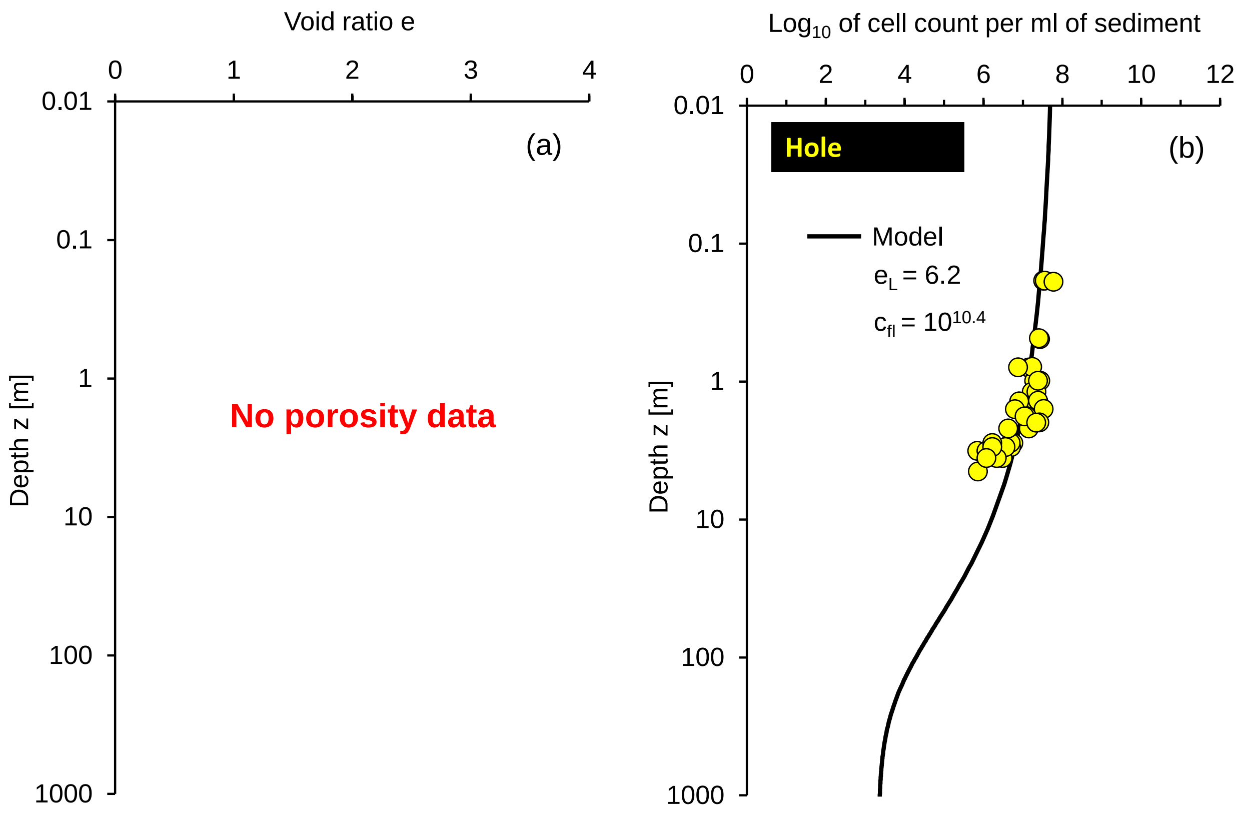

Supplementary Figure S120. North Sea Tidal Flat. Void ratio and cell count data profiles versus depth and prediction models. (A) Void ratio depth profile – Not available (model parameters: e_L = 6.2). (B) Cell count profile [data extracted from (Ref. 133)] (the estimated cell concentration of the pore fluid $c_{fl} = 10^{10.4}$ cell counts/cm^3).

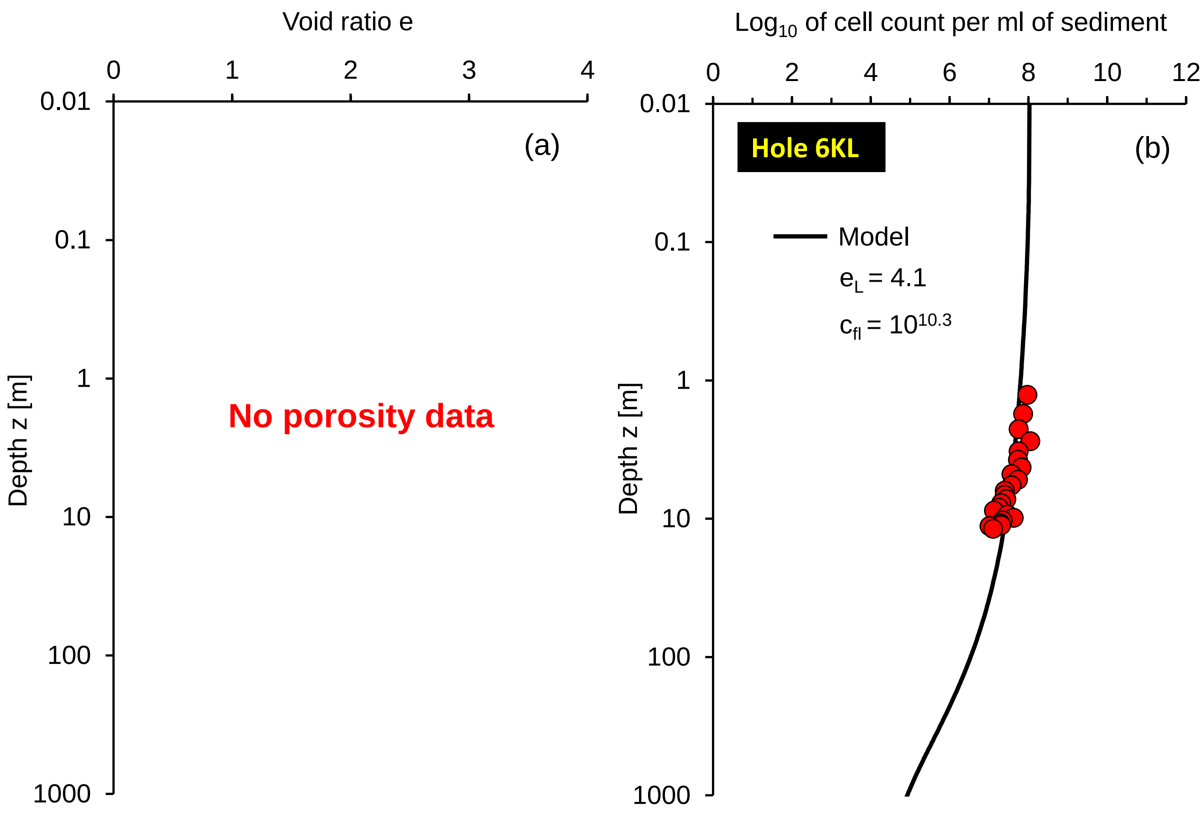

Supplementary Figure S121. SO 189 Forearc off Sumatra - Site 6KL. Void ratio and cell count data profiles versus depth and prediction models. (A) Void ratio depth profile - Not available (model parameters: e_L = 4.1). (B) Cell count profile [data extracted from (Ref. 134)] (the estimated cell concentration of the pore fluid c_{fl} = $10^{10.3}$ cell counts/cm^3).

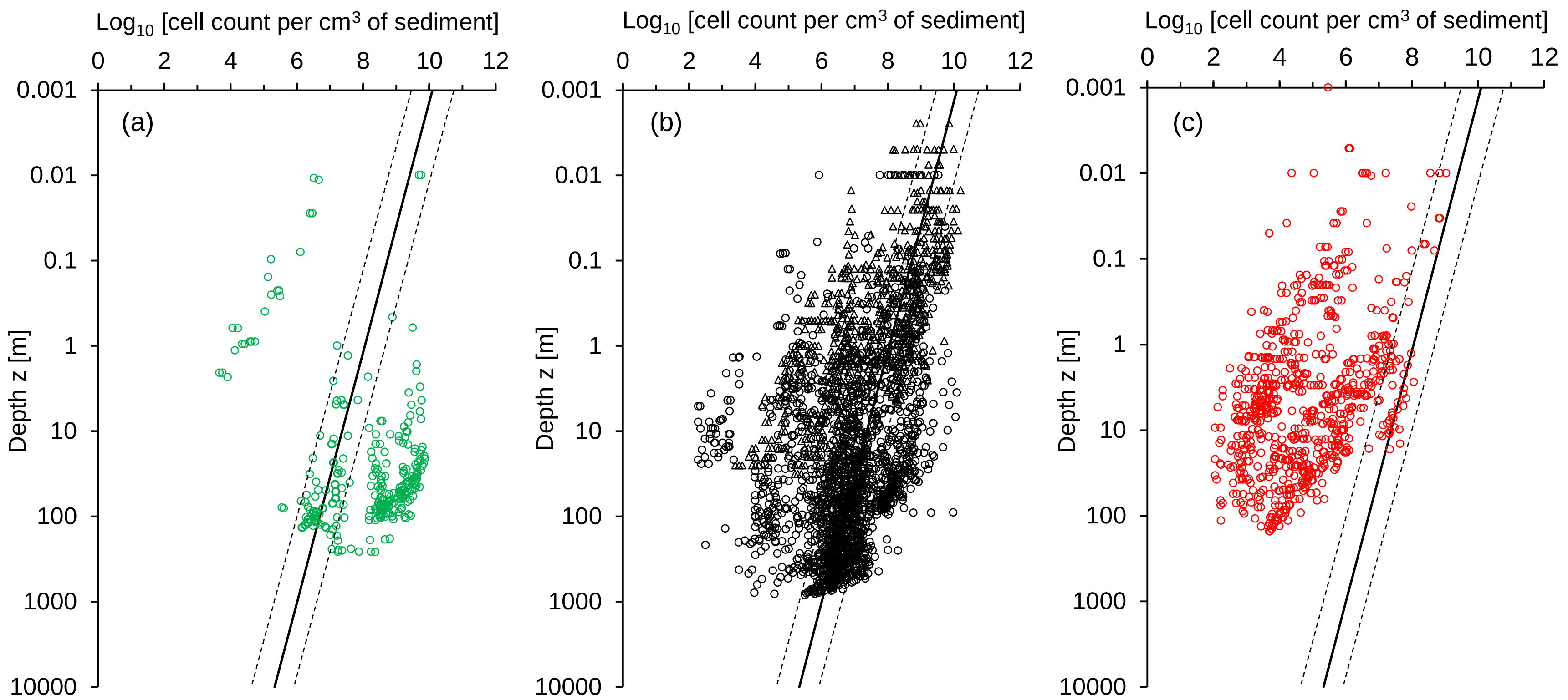

Supplementary Figure S122. Depth distribution of cell counts compiled from 116 expedition sites. (a) Sandy and silty sediments for $e_L < 2$. (b) Intermediate plasticity sediments for $2 < e_L < 5$. (c) High plasticity clayey sediments for $e_L > 5$. The black straight line indicates the regression line $\log_{10}$[cell counts] = 8.05-0.68·$\log_{10}$[depth/m] (Ref. 137). See Supplementary Table S3 and Supplementary Figures S7 to S120 for the complete database.

References

1. Phadnis, H. S. & Santamarina, J. C. Bacteria in sediments: pore size effects. *Geotech. Lett.* **1**, 91-93 (2011).
2. J. C. Santamarina, K. A. Klein, M. A. Fam, *Soils and waves: Particulate materials behavior, characterization and process monitoring* (Wiley, Chichester, 2001).
3. Tanaka, H., Shiwakoti, D. R., Omukai, N., Rito, F., Locat, J. & Tanaka, M. Pore size distribution of clayey soils measured by mercury intrusion porosimetry and its relation to hydraulic conductivity. *Soils. Found*. **43**, 63–73 (2003).
4. Juang, C. H. & Holtz, R. D. Fabric, pore size distribution, and permeability of sandy soils. *J. Geotech. Engng*. **112**, 355–368 (1986).
5. Jones, K. W., Feng, H., Tomov, S., Winters, W. J., Prodanović, M. & Mahajan, D. Characterization of methane hydrate host sediments using synchrotron-computed microtomography (CMT). *J. Petrol. Sci. Eng*. **56**, 136-145 (2007).
6. Ninjgarav, E., Chung, S. G., Jang, W. Y. & Ryu, C. K. Pore size distribution of Pusan clay measured by mercury intrusion porosimetry. *KSCE J. Civ. Eng.* **11**, 133-139 (2007).
7. Angeli, M., Benavente, D., Bigas, J. P., Menéndez, B., Hébert, R. & David, C. Modification of the porous network by salt crystallization in experimentally weathered sedimentary stones. *Mater. Struct*. **41**, 1091-1108 (2008).
8. Delage, P. A microstructure approach to the sensitivity and compressibility of some eastern Canada sensitive clays. *Géotechnique*. **60**, 353–368 (2010).
9. Garcia-Bengochea, I., Altschaeffl, A. G. & Lovell, C.W. Pore distribution and permeability of silty clays. *J. Geotech. Engng Div*. **105**, 839–855 (1979).
10. Simms, P. H. & Yanful, E. K. Measurement and estimation of pore shrinkage and pore distribution in a clayey till during soil–water characteristic curve tests. *Can. J. Geotech.* **38**, 741–754 (2001).
11. Monroy, R., Zdravkovic, L. & Ridley, A. Evolution of microstructure in compacted London Clay during wetting and loading. *Géotechnique*, **60**, 105-119 (2010).
12. Penumadu, D., & Dean, J. Compressibility effect in evaluating the pore-size distribution of kaolin clay using mercury intrusion porosimetry. *Can. Geotech. J*. **37**, 393-405 (2000).
13. Griffiths, F. J. & Joshi, R. C. Clay fabric response to consolidation. *Appl. Clay Sci*. **5**, 37–66 (1990).
14. Railsback, L. B. Contrasting styles of chemical compaction in the Upper Pennsylvanian Dennis Limestone in the Midcontinent region, USA. *J. Sediment. Res*. **63**, 61-72 (1993).
15. Corbett, K., Friedman, M. & Spang, J. Fracture development and mechanical stratigraphy of Austin Chalk, Texas. AAPG Bulletin, **71**, 17-28. (1987).
16. Cardona, A. & Santamarina, J. C. Carbonate rocks: Matrix permeability estimation. AAPG Bulletin, **104**, 131-144. (2020).
17. Fisher, W. L. & Rodda, P. U. Edwards Formation (Lower Cretaceous), Texas: dolomitization in a carbonate platform system. AAPG Bulletin, **53**, 55-72 (1969).

18. Churcher, P. L., French, P. R., Shaw, J. C. & Schramm, L. L. Rock properties of Berea sandstone, Baker dolomite, and Indiana limestone. In SPE International Symposium on Oilfield Chemistry. Society of Petroleum Engineers (1991).
19. Kyser, T. K., James, N. P., & Bone, Y. Alteration of Cenozoic cool-water carbonates to low-Mg calcite in marine waters, Gambier Embayment, South Australia. *J. Sediment. Res*, **68**, 947-955 (1998).
20. Weiner, W. F., & Van Groos, A. K. Petrographic and geochemical study of the formation of chert around the Thornton reef complex, Illinois. *Geol. Soc. Am. Bull.* **87**, 310-318 (1976).
21. Hakiki, F. Electromagnetic Properties of Geomaterials. Ph.D Thesis, King Abdullah University of Science and Technology (2020).
22. Zhang, L., Lu, S., Xiao, D. & Li, B. Pore structure characteristics of tight sandstones in the northern Songliao Basin, China. *Mar. Petrol. Geol*. **88**, 170-180 (2017).
23. Cao, Z., Liu, G., Zhan, H., Li, C., You, Y., Yang, C. & Jiang, H. Pore structure characterization of Chang-7 tight sandstone using MICP combined with N 2 GA techniques and its geological control factors. Scientific reports. **6**, 1-13 (2016).
24. Kuila, U. & Prasad, M. Specific surface area and pore-size distribution in clays and shales. *Geophys. Prospect*. **61**, 341-362 (2013).
25. Suess, E., von Huene, R. et al. Shipboard Scientific Party, Site 680. Proc. ODP, Init. Repts., 112: College Station, TX (Ocean Drilling Program), 249–303 (1988). doi:10.2973/odp.proc.ir.112.111.1988
26. Cragg, B. A., Parkes, R. J., Fry, J. C., Herbert, R. A., Wimpenny, J. W. T. & Getliff, J. M. Bacterial biomass and activity profiles within deep sediment layers. In Suess, E., von Huene, R., et al. Proc. ODP, Sci. Results, 112: College Station, TX (Ocean Drilling Program), 607–619 (1990). doi:10.2973/odp.proc.sr.112.161.1990
27. Suess, E., von Huene, R., et al. Shipboard Scientific Party, Site 681. Proc. ODP, Init. Repts., 112: College Station, TX (Ocean Drilling Program), 305–362 (1988). doi:10.2973/odp.proc.ir.112.112.1988
28. Ingle, J. C., Jr., Suyehiro, K., von Breymann, M. T. et al. Shipboard Scientific Party, Site 798. Proc. ODP, Init. Repts., 128: College Station, TX (Ocean Drilling Program), 121–236 (1990). doi:10.2973/odp.proc.ir.128.105.1990
29. Cragg, B. A., Harvey, S. M., Fry, J. C., Herbert, R. A. & Parkes, R. J. Bacterial biomass and activity in the deep sediment layers of the Japan Sea, Hole 798B. In Pisciotto, K. A., Ingle, J. C., Jr., von Breymann, M. T., Barron, J., et al. Proc. ODP, Sci. Results, 127/128 (Pt. 1): College Station, TX (Ocean Drilling Program), 761–776 (1992). doi:10.2973/odp.proc.sr.127128-1.184.1992
30. Parson, L., Hawkins, J., Allan, J., et al. Shipboard Scientific Party, Site 834. Proc. ODP, Init. Repts., 135: College Station, TX (Ocean Drilling Program), 85–180 (1992). doi:10.2973/odp.proc.ir.135.104.1992
31. Cragg, B. A. Bacterial profiles in deep sediment layers from the Lau Basin (Site 834). In Hawkins, J., Parson, L., Allan, J., et al. Proc. ODP, Sci. Results, 135: College Station, TX (Ocean Drilling Program), 147–150 (1994). doi:10.2973/odp.proc.sr.135.106.1994

32. Mayer, L., Pisias, N., Janecek, T., et al. Shipboard Scientific Party, Site 851. Proc. ODP, Init. Repts., 138: College Station, TX (Ocean Drilling Program), 891–965 (1992). doi:10.2973/odp.proc.ir.138.116.1992

33. Cragg, B. A. & Kemp, A. E. S. Bacterial profiles in deep sediment layers from the eastern equatorial Pacific Ocean, Site 851. In Pisias, N. G., Mayer, L. A., Janecek, T. R., Palmer-Julson, A. & van Andel, T. H. (Eds.), Proc. ODP, Sci. Results, 138: College Station, TX (Ocean Drilling Program), 599–604 (1995). doi:10.2973/odp.proc.sr.138.130.1995

34. Davis, E. E., Mottl, M. J., Fisher, A. T., et al. Shipboard Scientific Party, Site 857. Proc. ODP, Init. Repts., 139: College Station, TX (Ocean Drilling Program), 283–429 (1992). doi:10.2973/odp.proc.ir.139.107.1992

35. Cragg, B. A. & Parkes, R. J. Bacterial profiles in hydrothermally active deep sediment layers from Middle Valley (NE Pacific), Sites 857 and 858. In Mottl, M.J., Davis, E.E., Fisher, A.T. & Slack, J.F. (Eds.), Proc. ODP, Sci. Results, 139: College Station, TX (Ocean Drilling Program), 509–516 (1994). doi:10.2973/odp.proc.sr.139.236.1994

36. Davis, E. E., Mottl, M. J., Fisher, A. T., et al. Shipboard Scientific Party, Site 858. Proc. ODP, Init. Repts., 139: College Station, TX (Ocean Drilling Program), 431–569. (1992). doi:10.2973/odp.proc.ir.139.108.1992

37. Westbrook, G. K., Carson, B., Musgrave, R. J., et al., Shipboard Scientific Party, Site 888. Proc. ODP, Init. Repts., 146 (Pt. 1): College Station, TX (Ocean Drilling Program), 55–125 (1994). doi:10.2973/odp.proc.ir.146-1.007.1994

38. Cragg, B. A., Parkes, R. J., Fry, J. C., Weightman, A. J., Rochelle, P. A., Maxwell, J. R., Kastner, M., Hovland, M., Whiticar, M. J. & Sample, J. C. The impact of fluid and gas venting on bacterial populations and processes in sediments from the Cascadia margin accretionary system (Sites 888–892) and the geochemical consequences. In Carson, B., Westbrook, G. K., Musgrave, R. J. & Suess, E. (Eds.), Proc. ODP, Sci. Results, 146 (Pt. 1): College Station, TX (Ocean Drilling Program), 399–411 (1995). doi:10.2973/odp.proc.sr.146-1.241.1995

39. Westbrook, G. K., Carson, B., Musgrave, R. J., et al. Shipboard Scientific Party, Sites 889 and 890. Proc. ODP, Init. Repts., 146 (Pt. 1): College Station, TX (Ocean Drilling Program), 127–239 (1994). doi:10.2973/odp.proc.ir.146-1.008.1994

40. Westbrook, G. K., Carson, B., Musgrave, R. J. et al. Shipboard Scientific Party, Site 891. Proc. ODP, Init. Repts., 146 (Pt. 1): College Station, TX (Ocean Drilling Program), 241–300 (1994). doi:10.2973/odp.proc.ir.146-1.009.1994

41. Westbrook, G. K., Carson, B. Musgrave, R. J. et al. Shipboard Scientific Party, Site 892. Proc. ODP, Init. Repts., 146 (Pt. 1): College Station, TX (Ocean Drilling Program), 301–378 (1994). doi:10.2973/odp.proc.ir.146-1.010.1994

42. Kennett, J. P., Baldauf, J. G. et al. Shore-based Scientific Party, Site 893. Proc. ODP, Init. Repts., 146 (Pt. 2): College Station, TX (Ocean Drilling Program), 15–50 (1994). doi:10.2973/odp.proc.ir.146-2.024.1994

43. Cragg, B. A., Parkes, R. J., Fry, J. C., Weightman, A. J., Maxwell, J. R., Kastner, M., Hovland, M., Whiticar, M. J., Sample, J. C. & Stein, R. Bacterial profiles in deep sediments of the Santa Barbara Basin, Site 893. In Kennett, J. P., Baldauf, J. G. & Lyle, M. (Eds.),

Proc. ODP, Sci. Results, 146 (Pt. 2): College Station, TX (Ocean Drilling Program), 139–144. (1995). doi:10.2973/odp.proc.sr.146-2.302.1995

44. Flood, R. D., Piper, D. J. W., Klaus, A. et al., Shipboard Scientific Party, Site 934. Proc. ODP, Init. Repts., 155: College Station, TX (Ocean Drilling Program), 241–271. (1995). doi:10.2973/odp.proc.ir.155.110.1995
45. Cragg, B. A., Law, K. M., Cramp, A. & Parkes, R. J. Bacterial profiles in Amazon Fan sediments, Sites 934 and 940. In Flood, R.D., Piper, D.J.W., Klaus, A. & Peterson, L.C. (Eds.), Proc. ODP, Sci. Results, 155: College Station, TX (Ocean Drilling Program), 565–571 (1997). doi:10.2973/odp.proc.sr.155.229.1997
46. Flood, R. D., Piper, D. J. W., Klaus, A., et al. Shipboard Scientific Party, Site 940. Proc. ODP, Init. Repts., 155: College Station, TX (Ocean Drilling Program), 463–501 (1995). doi:10.2973/odp.proc.ir.155.116.1995
47. Emeis, K.-C., Robertson, A. H. F., Richter, C., et al. Shipboard Scientific Party, Site 969. Proc. ODP, Init. Repts., 160: College Station, TX (Ocean Drilling Program), 335–375 (1996). doi:10.2973/odp.proc.ir.160.110.1996
48. Cragg, B. A., Law, K. M., Cramp, A. & Parkes, R. J. The response of bacterial populations to sapropels in deep sediments of the Eastern Mediterranean (Site 969). In Robertson, A. H. F., Emeis, K.-C., Richter, C. & Camerlenghi, A. (Eds.), Proc. ODP, Sci. Results, 160: College Station, TX (Ocean Drilling Program), 303–307 (1998). doi:10.2973/odp.proc.sr.160.069.1998
49. Comas, M. C., Zahn, R., Klaus, A., et al. Shipboard Scientific Party, Site 976. Proc. ODP, Init. Repts., 161: College Station, TX (Ocean Drilling Program), 179–297 (1996). doi:10.2973/odp.proc.ir.161.106.1996
50. Cragg, B. A., Law, K. M., O'Sullivan, G. M. & Parkes, R. J. Bacterial profiles in deep sediments of the Alboran Sea, western Mediterranean, Sites 976–978. In Zahn, R., Comas, M. C. & Klaus, A. (Eds.), Proc. ODP, Sci. Results, 161: College Station, TX (Ocean Drilling Program), 433–438 (1999). doi:10.2973/odp.proc.sr.161.267.1999
51. Comas, M. C., Zahn, R., Klaus, A., et al. Shipboard Scientific Party, Site 977. Proc. ODP, Init. Repts., 161: College Station, TX (Ocean Drilling Program), 299–353 (1996). doi:10.2973/odp.proc.ir.161.107.1996
52. Comas, M. C., Zahn, R., Klaus, A., et al. Shipboard Scientific Party, Site 978. Proc. ODP, Init. Repts., 161: College Station, TX (Ocean Drilling Program), 355–388 (1996). doi:10.2973/odp.proc.ir.161.108.1996
53. Paull, C. K., Matsumoto, R., Wallace, P. J., et al. Shipboard Scientific Party, Site 994. Proc. ODP, Init. Repts., 164: College Station, TX (Ocean Drilling Program), 99–174 (1996). doi:10.2973/odp.proc.ir.164.107.1996
54. Wellsbury, P., Goodman, K., Cragg, B. A. & Parkes, R. J. The geomicrobiology of deep marine sediments from Blake Ridge containing methane hydrate (Sites 994, 995, and 997). In Paull, C. K., Matsumoto, R., Wallace, P. J. & Dillon, W. P. (Eds.), Proc. ODP, Sci. Results, 164: College Station, TX (Ocean Drilling Program), 379–391 (2000). doi:10.2973/odp.proc.sr.164.216.2000

55. Paull, C. K., Matsumoto, R., Wallace, P. J., et al. Shipboard Scientific Party, Site 995. Proc. ODP, Init. Repts., 164: College Station, TX (Ocean Drilling Program), 175–240 (1996). doi:10.2973/odp.proc.ir.164.108.1996
56. Paull, C. K., Matsumoto, R., Wallace, P. J., et al. Shipboard Scientific Party, Site 997. Proc. ODP, Init. Repts., 164: College Station, TX (Ocean Drilling Program), 277–334 (1996). doi:10.2973/odp.proc.ir.164.110.1996
57. Davis, E. E., Fisher, A. T., Firth, J. V., et al. Shipboard Scientific Party, Rough basement transect (Sites 1026 and 1027). Proc. ODP, Init. Repts., 168: College Station, TX (Ocean Drilling Program), 101–160 (1997). doi:10.2973/odp.proc.ir.168.105.1997
58. Mather, I. D. & Parkes, R. J. Bacterial profiles in sediments of the eastern flank of the Juan de Fuca Ridge, Sites 1026 and 1027. In Fisher, A.T., Davis, E. E. & Escutia, C. (Eds.), Proc. ODP, Sci. Results, 168: College Station, TX (Ocean Drilling Program), 161–165 (2000). doi:10.2973/odp.proc.sr.168.023.2000
59. Fouquet, Y., Zierenberg, R. A., Miller, D. J., et al. Shipboard Scientific Party, Middle Valley: Bent Hill area (Site 1035). Proc. ODP, Init. Repts., 169: College Station, TX (Ocean Drilling Program), 35–152 (1998). doi:10.2973/odp.proc.ir.169.103.1998
60. Cragg, B. A., Summit, M. & Parkes, R. J. Bacterial profiles in a sulfide mound (Site 1035) and an area of active fluid venting (Site 1036) in hot hydrothermal sediments from Middle Valley (northeast Pacific). In Zierenberg, R.A., Fouquet, Y., Miller, D. J., & Normark, W.R. (Eds.), Proc. ODP, Sci. Results, 169: College Station, TX (Ocean Drilling Program), 1–18 (2000). doi:10.2973/odp.proc.sr.169.105.2000
61. Fouquet, Y. Zierenberg, R. A. Miller, D. J., et al. Shipboard Scientific Party, Middle Valley: Dead Dog area (Site 1036). Proc. ODP, Init. Repts., 169: College Station, TX (Ocean Drilling Program), 153–203 (1998). doi:10.2973/odp.proc.ir.169.104.1998
62. Bornhold, B., Firth, J. V., et al. Shipboard Scientific Party, Sites 1033 and 1034. Proc. ODP, Init. Repts., 169S: College Station, TX (Ocean Drilling Program), 11–61 (1998). doi:10.2973/odp.proc.ir.169s.102.1998
63. Bird, D. F., Juniper, S. K., Riccardi-Rigault, M., Martineu, P., Prarie, Y. & Calvert, S. E. Subsurface viruses and bacteria in Holocene/Late Pleistocene sediments of Saanich Inlet, BC: ODP Holes 1033B and 1034B, Leg 169S. In Bornhold, B. D., & Kemp, A. E. S. (Eds.), Late Quaternary Sedimentation in Saanich Inlet, British Columbia, Canada–Ocean Drilling Program Leg 169S, Mar. Geol., 174:227–239 (2001). doi:10.1016/S0025-3227(00)00152-3
64. Gersonde, R., Hodell, D. A., Blum, P., et al. Shipboard Scientific Party, Site 1089. Proc. ODP, Init. Repts., 177: College Station, TX (Ocean Drilling Program), 1–97 (1999). doi:10.2973/odp.proc.ir.177.104.1999
65. Wellsbury, P., Mather, I. D. & Parkes, R. J. Bacterial abundances and pore-water acetate concentrations in sediments of the Southern Ocean (Sites 1088 and 1093). In Gersonde, R., Hodell, D. A. & Blum, P. (Eds.), Proc. ODP, Sci. Results, 177: College Station, TX (Ocean Drilling Program), 1–12 (2001). doi:10.2973/odp.proc.sr.177.109.2001
66. Gersonde, R., Hodell, D. A., Blum, P., et al. Shipboard Scientific Party, 1999. Proc. ODP, Init. Repts., 177: College Station, TX (Ocean Drilling Program), 1–101 (Site 1090). doi:10.2973/odp.proc.ir.177.105.1999

67. Taylor, B., Huchon, P., Klaus, A., et al. Shipboard Scientific Party, Site 1108. Proc. ODP, Init. Repts., 180: College Station, TX (Ocean Drilling Program), 1–134 (1999). doi:10.2973/odp.proc.ir.180.105.2000
68. Taylor, B., Huchon, P., Klaus, A., et al. Shipboard Scientific Party, Site 1109. Proc. ODP, Init. Repts., 180: College Station, TX (Ocean Drilling Program), 1–298 (1999). doi:10.2973/odp.proc.ir.180.106.2000
69. Taylor, B., Huchon, P., Klaus, A., et al. Shipboard Scientific Party, Sites 1110–1113. Proc. ODP, Init. Repts., 180: College Station, TX (Ocean Drilling Program), 1–89 (1999). doi:10.2973/odp.proc.ir.180.107.2000
70. Taylor, B., Huchon, P., Klaus, A., et al. Shipboard Scientific Party, Site 1115. Proc. ODP, Init. Repts., 180: College Station, TX (Ocean Drilling Program), 1–226 (1999). doi:10.2973/odp.proc.ir.180.109.2000
71. Taylor, B., Huchon, P., Klaus, A., et al. Shipboard Scientific Party, Site 1118. Proc. ODP, Init. Repts., 180: College Station, TX (Ocean Drilling Program), 1–213 (1999). doi:10.2973/odp.proc.ir.180.112.2000
72. Moore, G. F., Taira, A., Klaus, A., et al. Shipboard Scientific Party, Site 1173. Proc. ODP, Init. Repts., 190: College Station, TX (Ocean Drilling Program), 1–147 (2001). doi:10.2973/odp.proc.ir.190.104.2001
73. Moore, G. F., Taira, A., Klaus, A., et al. Shipboard Scientific Party, Site 1174. Proc. ODP, Init. Repts., 190: College Station, TX (Ocean Drilling Program), 1–149 (2001). doi:10.2973/odp.proc.ir.190.105.2001
74. Moore, G. F., Taira, A., Klaus, A., et al. Shipboard Scientific Party, Site 1175. Proc. ODP, Init. Repts., 190: College Station, TX (Ocean Drilling Program), 1–92 (2001). doi:10.2973/odp.proc.ir.190.106.2001
75. Moore, G. F., Taira, A., Klaus, A., et al. Shipboard Scientific Party, Site 1176. Proc. ODP, Init. Repts., 190: College Station, TX (Ocean Drilling Program), 1–80 (2001). doi:10.2973/odp.proc.ir.190.107.2001
76. Moore, G. F., Taira, A., Klaus, A., et al. Shipboard Scientific Party, Site 1177. Proc. ODP, Init. Repts., 190: College Station, TX (Ocean Drilling Program), 1–91 (2001). doi:10.2973/odp.proc.ir.190.108.2001
77. Moore, G. F., Taira, A., Klaus, A., et al. Shipboard Scientific Party, Site 1178. Proc. ODP, Init. Repts., 190: College Station, TX (Ocean Drilling Program), 1–108. (2001). doi:10.2973/odp.proc.ir.190.109.2001
78. D'Hondt, S. L., Jørgensen, B. B., Miller, D. J., et al. Shipboard Scientific Party, Site 1225. Proc. ODP, Init. Repts., 201: College Station, TX (Ocean Drilling Program), 1–86 (2003). doi:10.2973/odp.proc.ir.201.106.2003
79. D'Hondt, S. L., Jørgensen, B. B., Miller, D. J., et al. Shipboard Scientific Party, Site 1226. Proc. ODP, Init. Repts., 201: College Station, TX (Ocean Drilling Program), 1–96 (2003). doi:10.2973/odp.proc.ir.201.107.2003

80. D'Hondt, S. L., Jørgensen, B. B., Miller, D. J., et al. Shipboard Scientific Party, Site 1227. Proc. ODP, Init. Repts., 201: College Station, TX (Ocean Drilling Program), 1–66 (2003). doi:10.2973/odp.proc.ir.201.108.2003
81. D'Hondt, S. L., Jørgensen, B. B., Miller, D. J., et al. Shipboard Scientific Party, Site 1229. Proc. ODP, Init. Repts., 201: College Station, TX (Ocean Drilling Program), 1–78 (2003). doi:10.2973/odp.proc.ir.201.110.2003
82. D'Hondt, S. L., Jørgensen, B. B., Miller, D. J., et al. Shipboard Scientific Party, Site 1230. Proc. ODP, Init. Repts., 201: College Station, TX (Ocean Drilling Program), 1–107 (2003). doi:10.2973/odp.proc.ir.201.111.2003
83. D'Hondt, S. L., Jørgensen, B. B., Miller, D. J., et al. Shipboard Scientific Party, Site 1231. Proc. ODP, Init. Repts., 201: College Station, TX (Ocean Drilling Program), 1–64 (2003). doi:10.2973/odp.proc.ir.201.112.2003
84. Tréhu, A. M., Bohrmann, G., Rack, F. R., Torres, M. E., et al. Shipboard Scientific Party, Site 1244. Proc. ODP, Init. Repts., 204: College Station, TX (Ocean Drilling Program), 1–132 (2003). doi:10.2973/odp.proc.ir.204.103.2003
85. Inagaki, F., Nunoura, T., Nakagawa, S., Teske, A., Lever, M., Lauer, A., Suzuki, M., Takai, K., Delwiche, M., Colwell, F. S. & Nealson, K. H. Biogeographical distribution and diversity of microbes in methane hydrate-bearing deep marine sediments on the Pacific Ocean Margin. *Proc. Natl. Acad. Sci. U.S.A.* **103**, 2815-2820 (2006).
86. Tréhu, A. M., Bohrmann, G., Rack, F. R., Torres, M. E. et al. Shipboard Scientific Party, Site 1245. Proc. ODP, Init. Repts., 204: College Station, TX (Ocean Drilling Program), 1–131 (2003). doi:10.2973/odp.proc.ir.204.104.2003
87. Tréhu, A. M., Bohrmann, G., Rack, F. R., Torres, M. E. et al. Shipboard Scientific Party, Site 1251. Proc. ODP, Init. Repts., 204: College Station, TX (Ocean Drilling Program), 1–119 (2003). doi:10.2973/odp.proc.ir.204.110.2003
88. Fisher, A. T., Urabe, T., Klaus, A. & the Expedition 301 Scientists, Site U1301. Proc. IODP, 301: College Station TX (Integrated Ocean Drilling Program Management International, Inc.). (2005). doi:10.2204/iodp.proc.301.106.2005
89. Backman, J., Moran, K., McInroy, D. B., Mayer, L. A. & the Expedition 302 Scientists, Sites M0001–M0004. Proc. IODP, 302: Edinburgh (Integrated Ocean Drilling Program Management International, Inc.). (2006). doi:10.2204/iodp.proc.302.104.2006
90. Kallmeyer, J., Smith, D. C., Spivack, A. J. & D'Hondt, S. New cell extraction procedure applied to deep subsurface sediments. *Limnol. Oceanogr-Meth*. **6**, 236-245 (2008).
91. Ferdelman, T. G., Kano, A., Williams, T., Henriet, J.-P. & the Expedition 307 Scientists, Site U1316. Proc. IODP, 307: Washington, DC (Integrated Ocean Drilling Program Management International, Inc.). (2006). doi:10.2204/iodp.proc.307.103.2006
92. Ferdelman, T. G., Kano, A., Williams, T., Henriet, J.-P. & the Expedition 307 Scientists, Site U1317. Proc. IODP, 307: Washington, DC (Integrated Ocean Drilling Program Management International, Inc.). (2006). doi:10.2204/iodp.proc.307.104.2006

93. Ferdelman, T. G., Kano, A., Williams, T., Henriet, J.-P. & the Expedition 307 Scientists, Site U1318. Proc. IODP, 307: Washington, DC (Integrated Ocean Drilling Program Management International, Inc.). (2006). doi:10.2204/iodp.proc.307.105.2006

94. Flemings, P. B., Behrmann, J. H., John, C. M. & the Expedition 308 Scientists, Site U1319. Proc. IODP, 308: College Station TX (Integrated Ocean Drilling Program Management International, Inc.). (2006). doi:10.2204/iodp.proc.308.103.2006

95. Flemings, P. B., Behrmann, J. H., John, C. M. & the Expedition 308 Scientists, Site U1320. Proc. IODP, 308: College Station TX (Integrated Ocean Drilling Program Management International, Inc.). (2006). doi:10.2204/iodp.proc.308.104.2006

96. Flemings, P. B., Behrmann, J. H., John, C. M. & the Expedition 308 Scientists, Site U1322. Proc. IODP, 308: College Station TX (Integrated Ocean Drilling Program Management International, Inc.). (2006). doi:10.2204/iodp.proc.308.106.2006

97. Flemings, P. B., Behrmann, J. H., John, C. M. & the Expedition 308 Scientists, Site U1324. Proc. IODP, 308: College Station TX (Integrated Ocean Drilling Program Management International, Inc.). (2006). doi:10.2204/iodp.proc.308.108.2006

98. Pälike, H., Lyle, M., Nishi, H., Raffi, I., Gamage, K., Klaus, A. & the Expedition 320/321 Scientists, Site U1331. Proc. IODP, 320/321: Tokyo (Integrated Ocean Drilling Program Management International, Inc.). (2010). doi:10.2204/iodp.proc.320321.103.2010

99. Kallmeyer, J. Data report: microbial abundance in subseafloor sediments of the equatorial Pacific Ocean, Expedition 320/321. In Pälike, H., Lyle, M., Nishi, H., Raffi, I., Gamage, K., Klaus, A. & the Expedition 320/321 Scientists, Proc. IODP, 320/321: Tokyo (Integrated Ocean Drilling Program Management International, Inc.). (2013). doi:10.2204/iodp.proc.320321.214.2013

100. Pälike, H., Lyle, M., Nishi, H., Raffi, I., Gamage, K., Klaus, A. & the Expedition 320/321 Scientists, Site U1332. Proc. IODP, 320/321: Tokyo (Integrated Ocean Drilling Program Management International, Inc.). (2010). doi:10.2204/iodp.proc.320321.104.2010

101. Pälike, H., Lyle, M., Nishi, H., Raffi, I., Gamage, K., Klaus, A. & the Expedition 320/321 Scientists, Site U1334. Proc. IODP, 320/321: Tokyo (Integrated Ocean Drilling Program Management International, Inc.). (2010). doi:10.2204/iodp.proc.320321.106.2010

102. Pälike, H., Lyle, M., Nishi, H., Raffi, I., Gamage, K., Klaus, A. & the Expedition 320/321 Scientists, Site U1335. Proc. IODP, 320/321: Tokyo (Integrated Ocean Drilling Program Management International, Inc.). (2010). doi:10.2204/iodp.proc.320321.107.2010

103. Pälike, H., Lyle, M., Nishi, H., Raffi, I., Gamage, K., Klaus, A. & the Expedition 320/321 Scientists, Site U1337. Proc. IODP, 320/321: Tokyo (Integrated Ocean Drilling Program Management International, Inc.). (2010). doi:10.2204/iodp.proc.320321.109.2010

104. Pälike, H., Lyle, M., Nishi, H., Raffi, I., Gamage, K., Klaus, A. & the Expedition 320/321 Scientists, Site U1338. *Proc. IODP*, 320/321: Tokyo (Integrated Ocean Drilling Program Management International, Inc.). (2010). doi:10.2204/iodp.proc.320321.110.2010

105. Takahashi, K., Ravelo, A. C., Alvarez Zarikian, C. A. & the Expedition 323 Scientists, Site U1342. Proc. IODP, 323: Tokyo (Integrated Ocean Drilling Program Management International, Inc.). (2011). doi:10.2204/iodp.proc.323.106.2011

106. Kallmeyer, J., Pockalny, R., Adhikari, R. R., Smith, D. C. & D'Hondt, S. Global distribution of microbial abundance and biomass in subseafloor sediment. *Proc. Natl. Acad. Sci. U. S. A.* **109**. 16213–16216 (2012). doi.org/10.1073/pnas.1203849109

107. Takahashi, K., Ravelo, A. C., Alvarez Zarikian, C. A. & the Expedition 323 Scientists, Site U1343. Proc. IODP, 323: Tokyo (Integrated Ocean Drilling Program Management International, Inc.). (2011). doi:10.2204/iodp.proc.323.107.2011

108. D'Hondt, S., Abrams, L. J., Anderson, R., Dorrance, J., Durbin, A., Ellett, L., Ferdelman, T., Fischer, J., Forschner, S., Fuldauer, R., Goldstein, H., Graham, D., Griffith, W., Halm, H., Harris, R., Harrison, B., Hasiuk, F., Horn, G., Kallmeyer, J., Lever, M., Meyer, J., Morse, L., Moser, C., Murphy, B., Nordhausen, A., Parry, L., Pockalny, R., Puschell, A., Rogers, J., Schrum, H., Smith, D. C., Soffientino, B., Spivack, A. J., Stancin, A., Steinman, M. & Walczak, P. KNOX-02RR: drilling site survey—life in subseafloor sediments of the South Pacific Gyre. In D'Hondt, S., Inagaki, F., Alvarez Zarikian, C. A. & the Expedition 329 Scientists, Proc. IODP, 329: Tokyo (Integrated Ocean Drilling Program Management International, Inc.). (2011). doi:10.2204/iodp.proc.329.112.2011

109. D'Hondt, S., Inagaki, F., Alvarez Zarikian, C. A. & the Expedition 329 Scientists, Site U1365. Proc. IODP, 329: Tokyo (Integrated Ocean Drilling Program Management International, Inc.). (2011). doi:10.2204/iodp.proc.329.103.2011

110. D'Hondt, S., Inagaki, F., Alvarez Zarikian, C. A. & the Expedition 329 Scientists, Site U1366. Proc. IODP, 329: Tokyo (Integrated Ocean Drilling Program Management International, Inc.). (2011). doi:10.2204/iodp.proc.329.104.2011

111. D'Hondt, S., Inagaki, F., Alvarez Zarikian, C. A. & the Expedition 329 Scientists, Site U1367. Proc. IODP, 329: Tokyo (Integrated Ocean Drilling Program Management International, Inc.). (2011). doi:10.2204/iodp.proc.329.105.2011

112. D'Hondt, S., Inagaki, F., Alvarez Zarikian, C. A. & the Expedition 329 Scientists, Site U1368. Proc. IODP, 329: Tokyo (Integrated Ocean Drilling Program Management International, Inc.). (2011). doi:10.2204/iodp.proc.329.106.2011

113. D'Hondt, S., Inagaki, F., Alvarez Zarikian, C. A. & the Expedition 329 Scientists, Site U1369. Proc. IODP, 329: Tokyo (Integrated Ocean Drilling Program Management International, Inc.). (2011). doi:10.2204/iodp.proc.329.107.2011

114. D'Hondt, S., Inagaki, F., Alvarez Zarikian, C. A. & the Expedition 329 Scientists, Site U1370. Proc. IODP, 329: Tokyo (Integrated Ocean Drilling Program Management International, Inc.). (2011). doi:10.2204/iodp.proc.329.108.2011

115. D'Hondt, S., Inagaki, F., Alvarez Zarikian, C. A. & the Expedition 329 Scientists, Site U1371. Proc. IODP, 329: Tokyo (Integrated Ocean Drilling Program Management International, Inc.). (2011). doi:10.2204/iodp.proc.329.109.2011

116. Takai, K., Mottl, M. J., Nielsen, S. H. & the Expedition 331 Scientists, Site C0013. Proc. IODP, 331: Tokyo (Integrated Ocean Drilling Program Management International, Inc.). (2011). doi:10.2204/iodp.proc.331.103.2011

117. Takai, K., Mottl, M. J., Nielsen, S. H. & the Expedition 331 Scientists, Site C0014. Proc. IODP, 331: Tokyo (Integrated Ocean Drilling Program Management International, Inc.). (2011). doi:10.2204/iodp.proc.331.104.2011

118. Takai, K., Mottl, M. J., Nielsen, S. H. & the Expedition 331 Scientists, Site C0015. Proc. IODP, 331: Tokyo (Integrated Ocean Drilling Program Management International, Inc.). (2011). doi:10.2204/iodp.proc.331.105.2011

119. Takai, K., Mottl, M. J., Nielsen, S. H. & the Expedition 331 Scientists, Site C0017. Proc. IODP, 331: Tokyo (Integrated Ocean Drilling Program Management International, Inc.). (2011). doi:10.2204/iodp.proc.331.107.2011

120. Hirano, S., Ogawa, Y. & Kawamura, K. Deformation of unlithified sediments in an early stage of the compaction process deduced from microtextures and magnetic fabrics: ODP Leg 174B, Hole 1074A. In Becker, K. & Malone, M.J. (Eds.), Proc. ODP, Sci. Results, 174B: College Station, TX (Ocean Drilling Program), 1–13 (2001). doi:10.2973/odp.proc.sr.174b.131.2001

121. Edwards, K. J., Bach, W., Klaus, A. & the Expedition 336 Scientists, Site U1382. Proc. IODP, 336: Tokyo (Integrated Ocean Drilling Program Management International, Inc.). (2012). doi:10.2204/iodp.proc.336.104.2012

122. Edwards, K. J., Bach, W., Klaus, A. & the Expedition 336 Scientists, Site U1383. Proc. IODP, 336: Tokyo (Integrated Ocean Drilling Program Management International, Inc.). (2012). doi:10.2204/iodp.proc.336.105.2012

123. Breuker, A. & Schippers, A. Data report: total cell counts and qPCR abundance of Archaea and Bacteria in shallow subsurface marine sediments of North Pond: gravity cores collected during site survey cruise prior to IODP Expedition 336. In Edwards, K. J., Bach, W., Klaus, A. & the Expedition 336 Scientists, Proc. IODP, 336: Tokyo (Integrated Ocean Drilling Program Management International, Inc.). (2013). doi:10.2204/iodp.proc.336.201.2013

124. Inagaki, F., Hinrichs, K.-U., Kubo, Y. & the Expedition 337 Scientists, Site C0020. Proc. IODP, 337: Tokyo (Integrated Ocean Drilling Program Management International, Inc.). (2013). doi:10.2204/iodp.proc.337.103.2013

125. Andrén, T., Jørgensen, B. B., Cotterill, C., Green, S., Andrén, E., Ash, J., Bauersachs, T., Cragg, B., Fanget, A.-S., Fehr, A., Granoszewski, W., Groeneveld, J., Hardisty, D., Herrero-Bervera, E., Hyttinen, O., Jensen, J.B., Johnson, S., Kenzler, M., Kotilainen, A., Kotthoff, U., Marshall, I. P. G., Martin, E., Obrochta, S., Passchier, S., Quintana Krupinski, N., Riedinger, N., Slomp, C., Snowball, I., Stepanova, A., Strano, S., Torti, A., Warnock, J., Xiao, N. & Zhang, R. Site M0059. In Andrén, T., Jørgensen, B. B., Cotterill, C., Green, S. & the Expedition 347 Scientists, Proc. IODP, 347: College Station, TX (Integrated Ocean Drilling Program). (2015). doi:10.2204/iodp.proc.347.103.2015

126. Andrén, T., Jørgensen, B. B., Cotterill, C., Green, S., Andrén, E., Ash, J., Bauersachs, T., Cragg, B., Fanget, A.-S., Fehr, A., Granoszewski, W., Groeneveld, J., Hardisty, D., Herrero-Bervera, E., Hyttinen, O., Jensen, J.B., Johnson, S., Kenzler, M., Kotilainen, A., Kotthoff, U., Marshall, I. P. G., Martin, E., Obrochta, S., Passchier, S., Quintana Krupinski, N., Riedinger, N., Slomp, C., Snowball, I., Stepanova, A., Strano, S., Torti, A., Warnock, J., Xiao, N. & Zhang, R. Site M0060. In Andrén, T., Jørgensen, B. B., Cotterill, C., Green, S. & the Expedition 347 Scientists, Proc. IODP, 347: College Station, TX (Integrated Ocean Drilling Program). (2015). doi:10.2204/iodp.proc.347.104.2015

127. Andrén, T., Jørgensen, B. B., Cotterill, C., Green, S., Andrén, E., Ash, J., Bauersachs, T., Cragg, B., Fanget, A.-S., Fehr, A., Granoszewski, W., Groeneveld, J., Hardisty, D., Herrero-

Bervera, E., Hyttinen, O., Jensen, J.B., Johnson, S., Kenzler, M., Kotilainen, A., Kotthoff, U., Marshall, I. P. G., Martin, E., Obrochta, S., Passchier, S., Quintana Krupinski, N., Riedinger, N., Slomp, C., Snowball, I., Stepanova, A., Strano, S., Torti, A., Warnock, J., Xiao, N. & Zhang, R. Site M0061. In Andrén, T., Jørgensen, B. B., Cotterill, C., Green, S. & the Expedition 347 Scientists, Proc. IODP, 347: College Station, TX (Integrated Ocean Drilling Program). (2015). doi:10.2204/iodp.proc.347.105.2015

128. Andrén, T., Jørgensen, B. B., Cotterill, C., Green, S., Andrén, E., Ash, J., Bauersachs, T., Cragg, B., Fanget, A.-S., Fehr, A., Granoszewski, W., Groeneveld, J., Hardisty, D., Herrero-Bervera, E., Hyttinen, O., Jensen, J.B., Johnson, S., Kenzler, M., Kotilainen, A., Kotthoff, U., Marshall, I. P. G., Martin, E., Obrochta, S., Passchier, S., Quintana Krupinski, N., Riedinger, N., Slomp, C., Snowball, I., Stepanova, A., Strano, S., Torti, A., Warnock, J., Xiao, N. & Zhang, R. Site M0063. In Andrén, T., Jørgensen, B. B., Cotterill, C., Green, S. & the Expedition 347 Scientists, Proc. IODP, 347: College Station, TX (Integrated Ocean Drilling Program). (2015). doi:10.2204/iodp.proc.347.107.2015

129. Andrén, T., Jørgensen, B. B., Cotterill, C., Green, S., Andrén, E., Ash, J., Bauersachs, T., Cragg, B., Fanget, A.-S., Fehr, A., Granoszewski, W., Groeneveld, J., Hardisty, D., Herrero-Bervera, E., Hyttinen, O., Jensen, J.B., Johnson, S., Kenzler, M., Kotilainen, A., Kotthoff, U., Marshall, I. P. G., Martin, E., Obrochta, S., Passchier, S., Quintana Krupinski, N., Riedinger, N., Slomp, C., Snowball, I., Stepanova, A., Strano, S., Torti, A., Warnock, J., Xiao, N. & Zhang, R. Site M0065. In Andrén, T., Jørgensen, B. B., Cotterill, C., Green, S., and the Expedition 347 Scientists, Proc. IODP, 347: College Station, TX (Integrated Ocean Drilling Program). (2015). doi:10.2204/iodp.proc.347.109.2015

130. Inagaki, F., Suzuki, M., Takai, K., Oida, H., Sakamoto, T., Aoki, K., Nealson, K. H. & Horikoshi, K. Microbial communities associated with geological horizons in coastal subseafloor sediments from the Sea of Okhotsk. *Appl. Environ. Microbiol*. **69**, 7224-7235 (2003).

131. Breuker, A., Köweker, G., Blazejak, A. & Schippers, A. The deep biosphere in terrestrial sediments in the Chesapeake Bay area, Virginia, USA. *Front. Microbiol.* **2**, 156 (2011).

132. Roussel, E. G., Sauvadet, A. L., Chaduteau, C., Fouquet, Y., Charlou, J. L., Prieur, D. & Cambon Bonavita, M. A. Archaeal communities associated with shallow to deep subseafloor sediments of the New Caledonia Basin. *Environ. Microbiol*. **11**, 2446-2462. (2009).

133. Wilms, R., Sass, H., Köpke, B., Cypionka, H. & Engelen, B. Methane and sulfate profiles within the subsurface of a tidal flat are reflected by the distribution of sulfate-reducing bacteria and methanogenic archaea. *FEMS Microbiol. Ecol*. **59**, 611-621 (2007).

134. Schippers, A., Köweker, G., Höft, C. & Teichert, B. M. Quantification of microbial communities in forearc sediment basins off Sumatra. *Geomicrobiol. J*. **27**. 170-182 (2010).

135. Chong, S. H. & Santamarina, J. C. Soil compressibility models for a wide stress range. *J. Geotech. Geoenviron*. **142**, 06016003 (2016).

136. Salva Ramirez, M. Advanced sediment characterization. Ph.D Thesis, King Abdullah University of Science and Technology (2020).

137. Parkes, R. J., Cragg, B., Roussel, E., Webster, G., Weightman, A. & Sass, H. A review of prokaryotic populations and processes in sub-seafloor sediments, including biosphere: geosphere interactions. *Mar. Geol*. **352**, 409-425 (2014).
